# Author Correction: Like-minded sources on Facebook are prevalent but not polarizing

**DOI:** 10.1038/s41586-023-06795-x

**Published:** 2023-11-01

**Authors:** Brendan Nyhan, Jaime Settle, Emily Thorson, Magdalena Wojcieszak, Pablo Barberá, Annie Y. Chen, Hunt Allcott, Taylor Brown, Adriana Crespo-Tenorio, Drew Dimmery, Deen Freelon, Matthew Gentzkow, Sandra González-Bailón, Andrew M. Guess, Edward Kennedy, Young Mie Kim, David Lazer, Neil Malhotra, Devra Moehler, Jennifer Pan, Daniel Robert Thomas, Rebekah Tromble, Carlos Velasco Rivera, Arjun Wilkins, Beixian Xiong, Chad Kiewiet de Jonge, Annie Franco, Winter Mason, Natalie Jomini Stroud, Joshua A. Tucker

**Affiliations:** 1 Department of Government, Dartmouth College, Hanover, NH USA; 2 Department of Government and Data Science, William and Mary, Williamsburg, VA USA; 3 Maxwell School of Citizenship and Public Affairs, Syracuse University, Syracuse, NY USA; 4 Department of Communication, University of California, Davis, CA USA; 5 Amsterdam School of Communication Research, University of Amsterdam, Amsterdam, The Netherlands; 6 Meta, Menlo Park, CA USA; 7 CUNY Institute for State and Local Governance, New York, NY USA; 8 Environmental and Energy Policy Analysis Center, Stanford University, Stanford, CA USA; 9 Annenberg School for Communication, University of Pennsylvania, Philadelphia, PA USA; 10 Department of Economics, Stanford University, Stanford, CA USA; 11 Department of Politics, Princeton University, Princeton, NJ USA; 12 School of Public and International Affairs, Princeton University, Princeton, NJ USA; 13 Department of Statistics and Data Science, Carnegie Mellon University, Pittsburgh, PA USA; 14 School of Journalism and Mass Communication, University of Wisconsin-Madison, Madison, WI USA; 15 Network Science Institute, Northeastern University, Boston, MA USA; 16 Graduate School of Business, Stanford University, Stanford, CA USA; 17 Department of Communication, Stanford University, Stanford, CA USA; 18 School of Media and Public Affairs, The George Washington University, Washington, DC USA; 19 Institute for Data, Democracy, and Politics, The George Washington University, Washington, DC USA; 20 Moody College of Communication, University of Texas at Austin, Austin, TX USA; 21 Center for Media Engagement, University of Texas at Austin, Austin, TX USA; 22 Wilf Family Department of Politics, New York University, New York, NY USA; 23 Center for Social Media and Politics, New York University, New York, NY USA; 24 Present Address: Research Network Data Science, University of Vienna, Vienna, Austria

**Keywords:** Politics, Communication

Correction to: *Nature* 10.1038/s41586-023-06297-w Published online 27 July 2023

In the version of this article initially published, the variable described in Supplementary Table 36 as measuring Facebook “strikes” for violations of content policies against “Coordinated Inauthentic Behavior” (CIB) is inaccurate and does not reflect enforcement of the actual CIB policy, which is not a content-level policy. We therefore removed this row from Supplementary Table 36.

