# Supplementary material for: Like-minded sources on Facebook are prevalent but not polarizing
Source: Nature. 2023 Jul 27;620(7972):137–44. doi: 10.1038/s41586-023-06297-w (PMC10396953; doi:10.1038/s41586-023-06297-w)
Supplement: Supplementary file 1 — This file contains Supplementary Methods (including more details about the experimental implementation and classifiers used), Supplementary Tables and Figures (including descriptive statistics and analyses in separate sections), and Supplementary Notes (including the pre-analysis plan and the survey questionnaires). [file 41586_2023_6297_MOESM1_ESM.pdf]

nature portfolio

https://doi.org/10.1038/s41586-023-06297-w

Supplementary information

Like-minded sources on Facebook are prevalent but not polarizing

In the format provided by the
authors and unedited

nature portfolio

https://doi.org/10.1038/s41586-023-06297-w

Supplementary information

Like-minded sources on Facebook are prevalent but not polarizing

In the format provided by the
authors and unedited

Supplemental Information

Like-minded sources on Facebook are prevalent but not polarizing

Brendan Nyhan, Jaime Settle, Emily Thorson, Magdalena Wojcieszak, Pablo Barberá, Annie Y. Chen, Hunt Allcott, Taylor Brown, Adriana Crespo-Tenorio, Drew Dimmery, Deen Freelon, Matthew Gentzkow, Sandra González-Bailón, Andrew M. Guess, Edward Kennedy, Young Mie Kim, David Lazer, Neil Malhotra, Devra Moehler, Jennifer Pan, Carlos Velasco Rivera, Daniel Robert Thomas, Rebekah Tromble, Arjun Wilkins, Beixian Xiong, Chad Kiewiet de Jonge, Annie Franco, Winter Mason, Natalie Jomini Stroud, Joshua A. Tucker

Correspondence to: nyhan@dartmouth.edu

Contents

1 Supplementary Methods . . . S4
1.1 Implementation . . . S4
1.2 Ethics and harm minimization . . . S5
1.3 Classifiers . . . S8
1.4 Outcome measures . . . S9
1.4.1 Outcomes for preregistered hypotheses and research questions . . . S9
1.4.2 Outcomes for preregistered research questions . . . S13
1.5 Analytical approach . . . S17
1.5.1 Estimation of treatment effects: Main specification . . . S18
1.5.2 Weights: PATE vs. SATE . . . S18
1.5.3 Standard errors and p-values . . . S18
1.5.4 Adjustment for multiple comparisons . . . S19
2 Supplementary Tables and Figures: Descriptive Statistics . . . S21
2.1 Sample . . . S21
2.1.1 Sample characteristics . . . S21
2.1.2 Balance and attrition bias . . . S25
2.1.3 Deleted accounts and study withdrawals . . . S28
2.2 Exposure . . . S29
2.3 Sources and characteristics . . . S48
2.4 Behavior . . . S56
3 Supplementary Tables and Figures: Analyses . . . S61
3.1 Treatment effects: Exposure . . . S61

3.2 Treatment effects: Behavior S73
3.3 Treatment effects on outcomes for primary hypotheses (PATE/SATE) S77
3.4 Treatment effects on outcomes for research questions S78
3.5 Treatment effects on auxiliary outcome measures S79
3.6 Passive and active engagement component measures S82
3.7 Additional analyses S84
3.8 Wave-specific analyses S87
3.9 Treatment effects: Subgroup heterogeneity S89
3.9.1 Preregistered HTE estimation approach S90
3.9.2 HTE analysis approach: Deviations S93
3.9.3 HTE analysis for primary hypotheses S94
3.9.4 HTE analysis for research questions S111
3.9.5 HTE analysis for exploratory moderators S148
3.10 Exploratory analysis: Robustness check for (0.4, 0.6) exclusion S155
3.11 Exploratory analysis: Treatment effects on self-reported media use . . S159
3.12 Exploratory analysis: Equivalence bounds for treatment effect estimates S160
4 Supplementary Notes S168
4.1 Factor loadings of survey scales S168
4.2 Coding of on-platform behavior S172
4.3 Classification and categorization methods S175
4.3.1 Meta classifiers and categorization methods S175
4.3.2 Other classifiers and categorization methods S178
4.4 Passive tracking data S182
4.5 Sampling, strata definitions, randomization, and power analyses . . . S187
4.5.1 Sampling approach S187
4.5.2 Sampling frames and strata definitions S188
4.5.3 Sampling probabilities and target distributions S188
4.5.4 Power calculations S192
4.5.5 AmeriSpeak sample S193
4.6 Participant recruitment and consent S195
4.6.1 Recruitment language S195
4.6.2 Data collection timeline S197
4.6.3 Randomization S198
4.6.4 Response rates S199
4.7 Survey weights and PATE estimation S201
4.8 Meta/academic collaboration overview S203
4.9 Additional ethics considerations S209
4.9.1 Ethics and individual participants S209
4.9.2 Re-identification risk S209
4.9.3 Ethics and experiments during an election S210
4.9.4 Aggregated platform data S210

4.9.5 Professional ethics advice S211
4.10 Deviations S212
4.11 Survey questionnaire S220
4.12 References S380

1 Supplementary Methods

This section provides additional details about the design of the experiment, which was part of a broader set of experimental and observational studies conducted in collaboration between academics and Meta, the company formerly known as Facebook (see Section 4.8). In this Materials and Methods section, we provide information specific to our treatment intervention. Information that pertains to multiple studies in the collaboration such as details on sampling, randomization, and weights is provided in subsequent sections of this SI, which contain text adapted from a set of supplementary materials that shared across studies in the collaboration.

1.1 Implementation

Participants consisted of Facebook users age 18 and over who agreed to participate in a study of social media and politics and completed both baseline survey waves. They were recruited via survey invitations placed at the top of their feeds and remunerated for their participation (for additional details on sampling, power calculations, recruitment and consent, see Section 4.5 of the SI).

In this study, users who consented to participate were randomized into two experimental conditions: a control condition where no changes were made to their Facebook Feed or a treatment condition where content shared by friends, groups, and Pages predicted to be ideologically similar to the user was downranked in their Feed. Participants were also randomized to other treatment conditions as part of the other studies in the collaboration; the results of those studies will be reported in separate papers.

Ranking is the process Facebook uses to organize all the content users see, with the content deemed "the most relevant" at the top. Ranking has three elements:

1. **Inventory**: the collection of all posts that could be ranked for a given user. The eligible inventory includes any non-deleted post shared by a friend, Group, or Page that the user is connected to and was made since the user's last login. To make sure unseen posts are also reconsidered, posts that a user has not seen yet but that were ranked for the user in previous sessions are eligible again for the user to see. If any posts a user has already seen have triggered an interesting conversation among friends, the user may be eligible to see the post again.

2. **Posts**: once the inventory is identified, the algorithm scores each post in the inventory using multitask neural nets, which process data points such as type of post, embeddings (i.e., feature representations generated by deep learning models), and past exposures and engagements.

3. **A relevancy score** for each post, which is assigned based on multiple predictions that are specific to each user. First, integrity processes are applied to every post. These are designed to determine which integrity detection measures (such as removal or demotion)

need to be applied to the content selected for ranking, if any. A model is then run to select approximately 500 of the most relevant posts for a given user that are eligible for ranking. Each post is then scored independently and all 500 eligible posts are ordered by score. Finally, contextual features, such as content-type diversity rules, are added to shuffle the order in which posts are displayed in order to ensure that the Feed is relatively diverse. More information on the ranking algorithm can be found at https://engineering.fb.com/2021/01/26/ml-applications/news-feed-ranking/.

Our demotion was implemented by multiplying the relevancy score assigned to posts created by like-minded sources by a known factor, which determines the strength of the demotion. The intervention affected the content that respondents in the treatment group were exposed to from their friends, the groups of which they were a member, and the Pages that they followed. Ads and in-feed recommendations to content from groups of which they are not a member, and Pages that they do not follow were not affected by this intervention. The Feed of users who were assigned to the control condition did not change as a result of the experiment. For our experiment, after exploring different possible strengths, we selected the strongest demotion we tested, which corresponded to a multiplication factor of 0.05. The decision to implement this type of demotion, as opposed to an intervention in which all content from like-minded sources is hidden (i.e., a multiplication factor equal to 0), was driven by our intention to strike a balance between an intervention whose effects would be observable and one that would not result in high levels of attrition. In practice, a demotion using a multiplication factor of 0.05 does not lead to a 95% reduction in exposure to the content affected by the demotion because some users do not have other posts in inventory that could be shown to them with a relevancy score higher than that of the demoted content. In other words, the final position in which posts from like-minded sources were displayed to each user in the treatment group depended on their inventory. For example, users whose friends, groups, and Pages were primarily like-minded did not have to scroll down as far before they encountered like–minded content compared to those who had more diverse inventory. In our intervention, we observed a reduction in views from like-minded sources of around 33% (see Table 33). Additional details on how the effect of this demotion varied on characteristics such as pre-treatment exposure to like-minded sources are available in Supplementary Information, section 3.1.

1.2 Ethics and harm minimization

As with each intervention conducted as part of this study, all participants provided informed consent before taking part (see Section 4.6 for recruitment and consent materials). Participants were given the option to withdraw from the study while the experiment was ongoing (i.e., participants in treatment group could revert to a default feed without the demotion implemented in this study) as well as to withdraw their data (even if they participated in the full study) at any time up until their survey responses were disconnected from any identifying information (a step that took place in February 2023). External researchers could only access de-identified data when conducting analyses. The entire study was reviewed and approved by the NORC

IRB. Academic researchers worked with their respective university IRBs to ensure compliance with human subjects research regulations in analyzing data collected by NORC and Meta and authoring papers based on those findings. The research team also received ethical guidance from the independent firm Ethical Resolve to inform study designs. See the Supplementary Information, section 4.9 for additional discussion of ethical and privacy considerations. The lead authors with final control rights retain final discretion over everything reported in this paper. Meta did not have the right to pre-publication approval.

The study was designed to minimize the number of respondents who take part from any given state or district while providing adequate statistical power to test our hypotheses. Given the nature of the interventions and the context in which the study takes place, we took the following additional steps to mitigate any potential negative consequences. First, no content was entirely removed from any participant's feed; a participant would simply have to spend more time scrolling through their feed to find content from like-minded sources. Second, posts from the Pages of elected officials and candidates for public office at the federal, state, and local level were excluded from this treatment and thus were not downranked for any participant. However, reshares of politicians' posts by a user's friends or a Page that the participant follows could have been downranked if such friends/Pages were like-minded with respect to the participant.

In addition, we implemented a stopping rule, inspired by clinical trials, which stated that we would terminate the intervention before the election if we detected it was generating changes in specific variables related to individual welfare that were much larger than expected. The stopping rule was pre-registered at the Open Science Foundation `https://osf.io/upkns/` and is summarized below:

Specifically, we would have stopped the treatment and re-assigned users to the same feed experience as the control if any of the following effects were detected:

- If the treatment reduced turnout intention by significantly more than five percentage points (relative to control)
- If the treatment reduced registration rates by significantly more than five percentage points (relative to control)
- If the treatment increased exposure to content from untrustworthy sources (as a proportion of Feed content) by significantly more than 10 percentage points (relative to control)
- If the treatment increases exposure to misinformation content (as a proportion of Feed content) by significantly more than 10 percentage points (relative to control)

The control group baselines were as follows:

- Self-report intent to turn out (wave 3): 91%
- Self-report as registered voter (wave 3): 95%

- Pre-treatment share of views of content from untrustworthy sources[1]: 2.02%
- Pre-treatment share of views of misinformation on Feed[2]: 0.02%

Using a sample of 6,233 people in the treatment group and 13,890 in the control group who had completed wave 3 of the study as of October 23, 2020 and had activity on Facebook during the 28 days prior, we estimated the unweighted average treatment effects reported in Table 1. None of the stopping conditions listed above were met and we thus did not terminate the study.

Table 1: Treatment effect estimates for stopping rule analysis

	ATE (95% CI)
Voter turnout	-0.114 (-0.959, 0.731)
Voter registration	0.158 (-0.321, 0.636)
Untrustworthy source exposure	-0.147 (-0.205, -0.09)
Misinformation exposure	-0.002 (-0.002, -0.001)
N	20,123

Models estimated to assess conditions of stopping rules designed to prevent harms to study participants. Separate OLS models fit without sample weights. Each regression controlled for the pre-treatment outcome measure (except for turnout intention), whether the respondent lived in a battleground state, their total number of friends on Facebook, self-reported party ID, and race. Outcome measures are self-reported intent to vote, self-reported voter registration, share of views of content from untrustworthy sources, and share of views of misinformation on Feed.

[1]In the pre-registered stopping rule, "untrustworthy content", i.e. content from untrustworthy sources, consisted of original or reshared posts by Pages, in groups, or from web domains accruing two or more lifetime "strikes" for misinformation under Meta's Misinformation Repeat Offender program. While our main metric of exposure to "untrustworthy sources" in this study is estimated differently due to data retention limitations (see Section 4.3.2), here the construction of this metric matches our pre-registered definition because the data required to compute it was still available at the time. The metric is defined as the proportion of the total number of Feed views that were of content from untrustworthy sources aggregated over a 28-day period. The pre-treatment period was August 27–September 23, 2020 and the post-treatment period was September 25–October 22, 2020. The list of untrustworthy actors was defined as of October 22, 2020.

[2]Misinformation is defined as content rated as "false" by third-party fact-checkers or copies of such content as determined by text, image, and video matching algorithms. The metric is defined as the proportion of Feed views of misinformation over a 28-day period. The pre-treatment period was August 27–September 23, 2020 and the post-treatment period was September 25–October 22, 2020. The set of posts rated as misinformation was defined as of October 22, 2020.

1.3 Classifiers

This section describes the internal and external classifiers that were used as part of the project. For detailed descriptions of all classifiers and categorization methods used in this study and empirical measures of their performance, see Section 4.3.

We identified the content to be downranked for a given user with an approach that utilized an ideology classifier. First, prior to the start of the treatment, we estimated each user's political views with an internal Facebook classifier that predicts ideology. The classifier had 95 percent coverage of monthly active US users 18+. Predictions were made at the user level and range from 0 to 1, liberal to conservative. We used a threshold of 0.5, classifying those above or equal to 0.5 as conservative and those below 0.5 as liberal. We employed the mean ideology score for estimates generated in the 28 days prior to the start of the experiment. Data from 25 users for whom no classifier prediction was available were excluded from the analyses. Our 0.5 liberal/conservative threshold to classify participants in the experiment slightly differs from the standard thresholds used by Facebook to classify users (see Section 4.3 for more information). We chose this threshold to maximize coverage among participants. For friends, we differed from the standard Facebook thresholds to also use 0.4 (liberal) and 0.6 (conservative).

We also used this classifier to identify the ideological leanings of a user's friends as well as the ideology of the audience of the Pages they followed and the groups of which they were a member. For groups and Pages, we followed the standard cut-off points used by Facebook of 0.4 (liberal) and 0.6 (conservative). For Pages and groups, we computed audience ideology as the average estimated ideology of users engaging with these entities (e.g., those who shared content from Pages or groups or were a member of a Group).

Content by friends who were identified as left-leaning (predicted probability of .4 or below) or by Pages or groups with an audience identified as left-leaning (average audience ideology of .4 or below) was downranked for users whom we determined to be left-leaning (estimated ideology below 0.5). Content by friends who were identified as right-leaning (predicted probability of .6 or above) or by Pages or groups with an audience ideology identified as right-leaning (average audience ideology of .6 or above) was downranked for users whom we determined to be right-leaning (estimated ideology above 0.5). We did not alter the ranking of content from friends who were estimated to be neither like-minded nor cross-cutting or from Pages/groups with neither like-minded nor cross-cutting estimated audience ideology (between 0.4 and 0.6). For reshares on which estimates of ideology were not aligned (e.g., a post by a Page with a liberal audience that was shared by a conservative user), we used the predicted (audience) ideology of the content resharer.

In our analyses, we also relied on internal Meta classifiers as well as external classifiers created for this project, all of which are detailed in Section 4.3. From the internal Meta classifiers, we used the following:

- binary *'civic' classifier* to capture content in posts (links, photos, videos, text) as related to politics (government, elections, politicians, activism, etc) or social issues (major issues that affect a large group of people, such as the economy, inequality, racism, education,

immigration, human rights, the environment, etc.).

- binary *news classifier* for posts with a link or video that were about current events, timely information, and that followed journalistic standards, such as citing sources and containing a byline.

- *topic classifier* that categorizes content along 26 different broad topics (Animals & Pets, Books & Literature, Business, Finance & Economics, Crime & Tragedy, Education & Learning, Fashion & Style, Children & Parenting, Fitness & Workouts, Food & Drink, Games, Puzzles & Play, Health & Medical, History & Philosophy, Holidays & Celebrations, Home & Garden, Music & Audio, Performing Arts, Politics, Relationships, Friends & Family, Religion & Spirituality, Science & Tech, Social Issues, Sports, Travel & Leisure Activities, TV & Movies, Vehicles & Transportation, Visual Arts, Architecture & Crafts).

From the external classifiers, we used the following:

- *content with slur words classifier* which identifies content containing at least one term sourced from Hatebase and the Racial Slur Database and attempts to reduce false positives introduced by the inclusion of terms with ambiguous meaning.

- *incivility classifier* to capture uncivil content (*54*).

We also used four non-classifier-based content categorizations that are also described in Section 4.3. We relied on Facebook content policies to categorize the following:

- *problematic content* that violated one or more of Facebook enforcement policies (e.g., Coordinating Harm, Hate Speech, etc.)

- *misinformation repeat offender* content — domains, Pages, and groups on Facebook with 3+ misinformation strikes in the last 90 days

- *content from untrustworthy sources* — posts by Pages and in groups with 2+ lifetime misinformation strikes or containing links to domains with 2+ lifetime misinformation strikes

- *misinformation* — content rated “False” by a third-party fact-checker or post that matches a post rated “False” by a third-party fact checker

1.4 Outcome measures

1.4.1 Outcomes for preregistered hypotheses and research questions

This section lists our hypotheses and research questions, the survey items used to measure the outcome variables along with survey variable names (for specific question wording and response

options see Section 1.4), and any potential deviations from the measurement (for the list of and justification for all the deviations, see Section 4.10). The factor loadings described below are presented in Section 4.1.
H1: Decreased exposure to content shared by like-minded friends, Pages, and groups decreases affective polarization.

- *Pre-registered measurement of construct:* Average of three standardized measures, each created as the difference between the party a respondent was predicted to prefer and the other party: 1) difference in feeling thermometer scores between people who support the party the respondent preferred and people who support the other party (survey variable name: FT-PEOPC, FT-PEOPD); 2) difference in feeling thermometer scores between people running for office as the party the respondent preferred and people running for office from the other party (Wave 4), and difference in feeling thermometer scores between people who ran for office from the party the respondent preferred and people who ran for office from the other party (Wave 5) (survey variable name: FT-PEOPE, FT-PEOPF); 3) difference in perceptions of how smart people are who support the party the respondent preferred and people who support the other party (survey variable name: DEMSMART, REPSMART).
- *Implementation notes*: None
- *Deviation from pre-registered measurement*: None

H2: Decreased exposure to content shared by like-minded friends, Pages, and groups decreases partisan and ideological extremity.

- *Pre-registered measurement of construct:* Two separate measures: 1) partisan extremity using folded seven-point partisanship scale (survey variable names: PID, PIDSTRENGTH-D, PIDSTRENGTH-R, PIDLEAN); 2) ideological extremity using folded seven-point ideology scale (survey variable name: IDEO1)
- *Implementation notes*: See deviations.
- *Deviation from pre-registered measurement*: Party identification was omitted from the post-treatment waves, and so the measure only includes ideological extremity.

H3: Decreased exposure to content shared by like-minded friends, Pages, and groups decreases ideologically consistent issue positions and group evaluations.

- *Pre-registered measurement of construct:* Two composite scales using exploratory factor analysis with varimax rotation to select items from each of the two lists below. Those that did not clearly load on the same underlying dimension (i.e., the first principal component) were pre-registered to be analyzed separately. The final scales were pre-registered to be the average of the standardized measures included.

  - Scale 1 - Policy issues: immigration policy (IMMIG), health care policy (HEALTH), unemployment/tax policy (UNEMPLOY), Covid-related policy (COVID), foreign policy (FOREIGN), funding for police departments (POLICE). For each issue, we defined the "ideologically consistent" side as the position preferred by more people in the control group who had the same inferred ideology as the respondent (i.e., higher/lower mean), with the scale being the average of the standardized measures.
  - Scale 2 - Race: feeling thermometer toward Black Lives Matter (FT-PEOPLEGROUPS-I); perceptions as to the relative treatment of black people versus white people in dealing with the police(BLACKWHITE-A), when voting in elections (BLACKWHITE-B), when seeking medical treatment(BLACKWHITE-C), in hiring, pay and promotions (BLACKWHITE-D) using a five-point scale)

- *Implementation notes*: None
- *Deviation from pre-registered measurement*: None

H4: Decreased exposure to content shared by like-minded friends, Pages, and groups decreases ideologically consistent vote choice and candidate evaluations.

- *Pre-registered measurement of construct:* A composite scale using exploratory factor analysis with varimax rotation to select items from the list below. Those that did not clearly load on the same underlying dimension (i.e., the first principal component) were pre-registered to be analyzed separately. The final scale was pre-registered to be the average of the standardized measures included.
  - Presidential vote choice (-1 = support candidate from uncongenial party, 0 = other/DK, 1= support candidate from congenial party; survey variable name VOTE-POST)
  - Senate/House/Governor vote choice (-1 = support candidate from uncongenial party, 0 = other/DK, 1 = support candidate from congenial party; survey variable names VOTESENATE, VOTESENATE2, VOTEHOUSE, VOTEGOV)
  - Feeling thermometer toward presidential candidate from congenial party as defined using inferred ideology (0-100, FT-PEOPA, FT-PEOPB)
  - Feeling thermometer toward presidential candidate from uncongenial party as defined using inferred ideology (100-FT, FT-PEOPA, FT-PEOPB)
- *Implementation notes*: Senate and Governor vote choice was missing for more than 15% of respondents, so both measures were analyzed separately (see "Auxiliary outcome measures").
- *Deviation from pre-registered measurement*: Some people did not complete the vote choice question in W4 but did complete it in W5. In these cases, we used their vote choice response from W5. We use only the feeling thermometers from W4, since vote

choice is measured in Wave 4 for the substantial majority of respondents and we wanted to maintain temporal consistency within the measure. The Wave 5 feeling thermometers are included in the auxiliary analyses.

H5: Decreased exposure to content shared by like-minded friends, Pages, and groups decreases partisan-congenial beliefs about election misconduct and outcomes, and partisan-congenial views toward the electoral system and respect for electoral norms.

- *Pre-registered measurement of construct:* For each of the sets of three or more questions below, we pre-registered constructing a composite scale using exploratory factor analysis with varimax rotation to select items in each list. Those that did not clearly load on the same underlying dimension (i.e., the first principal component) were pre-registered to be analyzed separately. The scale was defined as the average of the standardized measures included. Note that for these items, we code each differently depending on whether the respondent is identified (based on the Facebook classifier) as a conservative or liberal.
  - Party-congenial beliefs:
    * Which candidate won the 2020 election (ELECTWIN): Joe Biden (2 if Democrat, 0 if Republican, Donald Trump (0 if Democrat, 2 if Republican), or Not yet determined (1)
    * How often registered voters were illegally prevented from voting (IRREG2020A): Often (4 if Democrat, 1 if Republican), Sometimes (3 if Democrat, 2 if Republican), Rarely (2 if Democrat, 3 if Republican), Never (1 if Democrat, 4 if Republican
    * How often people voted illegally (IRREG2020B): Often (1 if Democrat, 4 if Republican), Sometimes (2 if Democrat, 3 if Republican), Rarely (3 if Democrat, 2 if Republican), Never (4 if Democrat, 1 if Republican);
    * asked only of people who did not answer "Never" to IRREG2020A Whether people illegally prevented from voting changed the outcome of the presidential election (PREVENTEFFECT2020): Yes (4 if Democrat, 1 if Republican), No (2 if Democrat, 3 if Republican), Not sure (3 if Democrat, 2 if Republican), [never on IRREG2020=1 if Democrat, 4 if Republican];
    * asked only of people who did not answer "Never" to IRREG2020B Whether people illegally voting changed the outcome of the presidential election (ILLEGALVOTEEFFECT2020): Yes (4 if Republican, 1 if Democrat), No (2 if Republican, 3 if Democrat), Not sure (3 if Republican, 2 if Democrat), [never on IRREG2020=1 if Republican, 4 if Democrat].
  - Confidence in elections:
    * Confidence in the officials who oversee elections (CONFOFFICIALS): None (1 if Democrat, 5 if Republican), A little (2 if Democrat, 4 if Republican), A

moderate amount (3 if Democrat, 3 if Republican), A lot (4 if Democrat, 2 if Republican), A great deal (5 if Democrat, 1 if Republican);

    * How accurately the votes were counted in the 2020 general election (COUNTACCURATE): Not at all accurately (1 if Democrat, 5 if Republican), Not very accurately (2 if Democrat, 4 if Republican), Moderately accurately (3 if Democrat, 3 if Republican), Very accurately (4 if Democrat, 2 if Republican), Completely accurately (5 if Democrat, 1 if Republican);
    * How much do you trust that votes are counted accurately when people mail in their ballots? (MAILACCURATE): A great deal (5 if Democrat, 1 if Republican), A lot (4 if Democrat, 2 if Republican), A moderate amount (3 if Democrat, 3 if Republican), A little (2 if Democrat, 4 if Republican), Not at all (1 if Democrat, 5 if Republican).

  - Respect for election norms:
    * Whether Donald Trump should or should not concede the election to Joe Biden (TRUMPCONCEDE): Definitely should concede (4 if Democrat, 1 if Republican), Probably should concede (3 if Democrat, 2 if Republican), Probably should not concede (2 if Democrat, 3 if Republican), Definitely should not concede (1 if Democrat, 4 if Republican).
  - *Implementation notes*: The belief that registered voters were prevented from voting and that this affected election outcomes did not load with the other items and so were analyzed separately (see "Auxiliary outcome measures").
  - *Deviation from pre-registered measurement*: We used inferred ideology to determine congeniality, rather than self-reported partisanship. This maintains consistency with the rest of the pre-registered analyses, for which we use inferred ideology rather than self-reported partisanship.

1.4.2 Outcomes for preregistered research questions

RQ1: How does decreased exposure to content shared by ideologically like-minded friends, groups, and Pages affect online and self-reported offline participation?

- *Pre-registered measurement of construct:* Four separate measures:
  - Validated voter turnout from survey vendor: 0 = no validated vote in 2020 general election found in voter file data, 1 = validated vote found for 2020 general election.
  - Self-reported turnout: "I am sure I voted" (1) vs. did not vote (0: "I did not vote"; "I thought about voting this time, but didn't"; "I usually vote, but didn't this time," survey variable name TURNOUT)
  - Self-reported participation: Sum of 6 binary measures tapping different aspects of political engagement (variable name POLPART)

- On-platform political participation: For the nine on-platform political participation measures, we pre-registered to take the natural log of the ratio of the frequency of that metric to the frequency of views of civic content (to account for the skewness of these behaviors and for the rates of these behaviors relative to exposure). The composite scale was to be created using exploratory factor analysis with varimax rotation to select the items, with those that did not clearly load on the same underlying dimension (i.e., the first principal component) pre-registered to be analyzed separately. The final scale was to be constructed as the average of the standardized measures included:
    - ∗ civic content engagement (click, like, comment, share, or reaction on a post labelled civic by the civic classifier),
    - ∗ civic events (indication of interest in going to a civic event, indication of going to a civic event, or creation of a civic event),
    - ∗ engagement with Voter Hub (click, like, comment, share, or reaction),
    - ∗ engagement with Town Hall (click, like, comment, share, or reaction),
    - ∗ sharing that you voted with others using "Share You Voted" feature, clicking on or sharing of a petition,
    - ∗ donating to civic causes on the Facebook platform,
    - ∗ enabling the constituent badge,
    - ∗ engagement with politicians and candidates running for office (i.e., reacting/liking, commenting, resharing of posts, tags, mentions, or contacting a politician using Facebook Messenger)

- *Implementation notes*:

- *Deviation from pre-registered measurement*: There was a typo in the initial description of how the measure should be constructed (it should be "the ratio of the natural log" rather than "the natural log of the ratio"). In addition, rather than analyzing only those actions that loaded on the first dimension, we opted to use exploratory factor analysis with varimax rotation to select items, then analyzed the factor scores of the resulting three-dimensional solution. We included the first dimension as our primary analysis, and then the second and third as auxiliary analyses. Finally, the following pre-registered on-platform participation measures were not available: Voter Hub likes, comments, shares, and reactions were not logged; the Town Hall product did not allow for likes, comments, shares, or reactions; and the "Share Your Vote" feature was not used in the 2020 election. Several additional participation measures were added: views on Voter Hub, and follows and contacts on Town Hall.

RQ2: How does decreased exposure to content shared by ideologically like-minded friends, groups, and Pages affect their perceptions of whether they've seen political conversations, the

nature of the conversations they see on Facebook, and the partisan composition of their friends on the platform?

- *Pre-registered measurement of construct:* Four measures:
  - Two items analyzed separately (survey measure NETDIVFB-GROUP): The reported number of Facebook friends who are (a) congenial party, (b) uncongenial party; both based on inferred ideology: Democrats/Republicans (5 = all or nearly all, 1 = none or almost none)
  - A binary measure asking about exposure to political conversations on Facebook in the past week (survey measure FBPOLCON).
  - Whether the political conversations seen on Facebook in the last week were "Respectful" and "informative" (five-point scale, survey variable name FBPOLDES). These two items were pre-registered to be a scale as the average of the standardized measures if the Pearson's correlation between was greater than 0.6; if not, pre-registered to be analyzed separately. These outcomes were pre-registered to be assessed if the results are null for effects on self-reports of seeing political conversation due to the risk of post-treatment bias.
- *Implementation notes*: The correlation between "respectful" and "informative" was greater than .6 and so the two variables were analyzed together.
- *Deviation from pre-registered measurement*: None.

RQ3: How does decreased exposure to content shared by like-minded friends, Pages, and groups affect perceived polarization?

- *Pre-registered measurement of construct:* A composite scale using exploratory factor analysis with varimax rotation to select items below. Those that do not clearly load on the same underlying dimension (i.e., the first principal component) were pre-registered to be analyzed separately. The final scale was pre-registered to be the average of the standardized measure included. Rating the three individuals and groups? (seven-point scale; survey variable name IDEOLOGY-GROUP)
- Absolute difference in perceived ideology between people who support Democrats (1-7) and people who support Republicans (1-7)
- Absolute difference in perceived ideology between Democrats running for office (1-7) and Republicans running for office (1-7)
- Absolute difference in perceived ideology between people the respondent sees on Facebook who support Democrats (1-7) and people the respondent sees on Facebook who support Republicans (1-7)

- *Implementation notes*: None.
- *Deviation from pre-registered measurement*: None.

RQ4: Does decreased exposure to content shared by like-minded friends, Pages, and groups decrease own-party affinity or out-party antipathy?

- *Pre-registered measurement of construct:* Composite scales using exploratory factor analysis with varimax rotation to select items below. Those that do not clearly load on the same underlying dimension (i.e., the first principal component) were pre-registered to be analyzed separately. The final scale was pre-registered as the average of the standardized measures included.
- Party the respondent prefers: Feeling thermometer scores for people (a) who support the congenial party and (b) running for office from the congenial party (0-100), survey variable name FTPEOPLEGROUPS; (c) Perceptions of how smart people are who support the congenial party, five-point scale (DEMSIART, REPSIART)
- Party that respondent does not prefer: Feeling thermometer scores for people who (a) support the uncongenial party and (b) running for office from the uncongenial party (0-100), survey variable name FTPEOPLEGROUPS; (c) Perceptions of how smart people are who support the uncongenial party, five-point scale (DEMSIART, REPSIART)
- *Implementation notes*: The two "smart" measures were not asked in Wave 5 and so were not included in the scale (see "Auxiliary Analyses" section).
- *Deviation from pre-registered measurement*: None.

RQ5: Does decreased exposure to content shared by like-minded friends, Pages, and groups affect the perceived accuracy of false claims about current events, especially those that are pro-attitudinal?

- *Pre-registered measurement of construct:* Mean belief in false claims as well as the difference between mean belief in true claims and mean belief in false claims for all claims and for two pro-attitudinal subsets for liberal and conservative participants, pre-registered to be standardized at the respondent level, four-point scale (MISINFO):
- Wave 4: questions about currently circulating fake news topics, Conservative, Liberal, COVID-related, and also false statements by Trump and by Biden. Wave 4 also included true liberal-congenial statement, true conservative-congenial statement, and true COVID statements. Pro-attitudinal subsets for liberal and conservative participants were to be created using participants' inferred ideology (survey variable name: MISINFO)
- Wave 5: Six false statements related to the election, three true but plausibly false statements (survey variable name: MISINFO).

- *Implementation notes*: Because different false claims were included on the Wave 4 and Wave 5 surveys, these were analyzed separately.
- *Deviation from pre-registered measurement*: The Wave 5 misinformation battery as pre-registered inadvertently omitted the false, liberal-congenial item "Church bells rang in Paris to celebrate Joe Biden's victory." It was included in the scale.

RQ6: Does decreased exposure to content shared by like-minded friends, Pages, and groups affect perceptions of democratic performance?

- *Pre-registered measurement of construct:* Average of standardized responses to six statements as to whether U.S. meets a given standard (USDEMOC).
- *Implementation notes*: None.
- *Deviation from pre-registered measurement*: None.

RQ7: Does decreased exposure to content shared by like-minded friends, Pages, and groups affect the ideological extremity of the web domains that people visit?

- *Pre-registered measurement of construct:* Non-survey based measure, applies only to respondents with behavioral data from online browsers: mean audience ideology of domains visited by respondents during the treatment period (based on the Facebook audience ideology classifier) weighted by the number of visits to each domain (i.e., frequency weights). The final measure is the absolute value of the difference between this value and 0.5.
- *Implementation notes*: None.
- *Deviation from pre-registered measurement*: The PAP did not specify whether we would restrict this analysis only to those who completed the survey. Thus, we only used web tracking data from respondents who completed Waves 3, 4, or 5 of the survey to maintain consistency with the Facebook log data measures, for which we also use only people who completed one of those waves.

1.5 Analytical approach

This section describes the pre-registered models and analysis plan.

1.5.1 Estimation of treatment effects: Main specification

The pre-registration stated that we would estimate treatment effects for outcome variables that are measured in multiple survey waves separately by wave. However, prior to viewing any results we registered a deviation that we would combine items measured in multiple waves into a single measure that takes the average across waves (i.e., mean response among non-missing data for waves 4 and 5). This decision was made because the events of the election continued after Election Day (as did our treatment), so we expected our treatment to continue to have meaningful effects on exposure to political content throughout that period. However, we also include estimates separately by wave in Section 11.

We report a baseline model using OLS regression with variables selected via lasso included as controls (X_i) as well as stratum indicators:

$$Y_i = \alpha + \tau * \text{Treatment}_i + \beta_1 * X_i + \beta_2 * \text{StratumDummies} + \epsilon_i$$

To select covariates, we used lasso (with default options in `cv.glmnet`: 10 folds, seed = 2020) following this procedure:

For the lasso model, include pre-treatment DVs (if available) and survey-based metrics from wave 1 and wave 2: gender, age, race/ethnicity (non-Hispanic white, Hispanic, non-Hispanic black, AAPI, Other), ideology, 7-point party ID, turnout in 2016, news consumption [network TV, average of cable, online websites, average of social media (FB, IG, Tw, YT), newspapers], political interest, sum of political participation, and sum of digital literacy. Stratum indicators and treatment assignment were not included in this model. The model was computed on the full sample (treatment and control units). If one or more levels (e.g., "Northeast") were selected from a factor variable, we included only the selected level(s) in the model. Finally, the pre-registration did not specify whether variables that do not load in a post-treatment scale should be included in the pre-treatment scale used as a control. We registered a deviation (prior to seeing the results) that those variables should be excluded.

1.5.2 Weights: PATE vs. SATE

For each treatment effect estimate, we used two estimands on a combination of subsamples. We designated the Population Average Treatment Effect (PATE) as the primary estimand reported in the main text, which was computed using weights. Additionally, in Section 3.3, we report our estimates of Sample Average Treatment Effects (SATE) for the full sample.

All descriptive statistics based on the survey data were computed using the same set of weights to recover the population-level descriptive statistics. For further details on how the weights were constructed, see Section 4.7.

1.5.3 Standard errors and p-values

We used two-sided tests with $p < 0.05$ as our measure of statistical significance for all the tests. Regression analyses used HC2 robust standard errors.

1.5.4 Adjustment for multiple comparisons

In the pre-registration, we designated all outcome measures in the hypotheses and research questions as primary outcomes for the purposes of multiple comparisons testing. However, prior to viewing any results we registered a deviation that we would treat all RQ tests as secondary outcomes. Our reasoning was that the RQs substantially inflated the number of primary tests because many of the RQs had separate outcome measures and we had weaker theoretical expectations for the RQs than for the hypotheses.

In the pre-registration (`https://osf.io/3sjy2`), we indicated that all items that were excluded from preregistered indices or scales were considered to be primary outcomes. However, prior to viewing any results we registered a deviation that we would instead report all items that were excluded from preregistered indices or scales as auxiliary tests reported only in an appendix with unadjusted p-values. Our reasoning was that if these items did not load with the other items, it might be because they are measured with error or were not good measures of the underlying construct, we did not have pre-registered theoretical expectations for individual outcome measures that do not load with the other items in the scale, and these individual outcome measures are less important in the context of the project.

We implemented a sharpened FDR adjustment (*55*) with code adapted from (*56*) with four groups of variables:

- K1 primary outcomes (hypotheses and research questions - scales and outcome measures excluded from scales and analyzed separately)
  - Sharpened FDR-adjusted p-values with these K1 outcomes
- K2 secondary outcomes (individual outcome measures in scales)
  - Sharpened FDR-adjusted p-values with K1+K2 outcomes
  - These must be clearly labeled as secondary outcomes
  - If analyzed, any single wave-specific outcome measure that is part of an index or part of an average across waves will be considered to be a secondary outcome unless otherwise specified in the PAP. All such individual outcome measures within indices or measures that are repeated over waves should be included in the set of K2 outcomes if any analysis of such measures is conducted.
- L1 primary tests of moderation/subgroups/heterogeneity
  - If these tests are specified in relation to specific hypotheses, this implies sharpened FDR-adjusted p-values with L1 tests
  - For PAPs with hypotheses related to heterogeneity across groups, those tests should be included as part of the K1 primary outcomes

- When testing the subgroup effects individually, as opposed to heterogeneity in treatment effects, the number of tests should be equivalent to the number of effects (i.e., p-values) being estimated
- If a list of moderators is provided to test across all hypotheses, this implies controlling FDR for L1 * K1 hypothesis tests (unless otherwise specified).

Auxiliary tests (e.g., substitution, first stage effects on usage) are not adjusted (generally these are variables for which statistical significance should not matter).

2 Supplementary Tables and Figures: Descriptive Statistics

2.1 Sample

In this section, we describe characteristics of our sample, including their demographics, time on Facebook, selection and attrition, and randomization.

2.1.1 Sample characteristics

Extended Data Table 5 compares demographic characteristics of survey participants (both unweighted as well as well as weighted to match characteristics of active Facebook users; see Section 4.7 for more details) as well as the Amerispeak sample of Facebook users, and the AmeriSpeak sample of the U.S. population more generally. See Section 4.5 for additional details on the AmeriSpeak sample.

Table 2 shows the distribution and characteristics of our sample's Facebook usage compared to the set of Facebook monthly active users age 18 and above that were part of the sampling frame (denoted as "US MAP" in the table). Aggregated usage levels are measured in the subset of U.S. adults who accessed Facebook at least once in the 30 days preceding August 17, 2020 (see Section 4.9.4 for details). During the third and fourth quarters of 2020, which encompasses this interval as well as the study period for the experiment reported below, Facebook had 231 million users who accessed it every month in the U.S. The tables below display the standardized time that monthly active users age 18 and above spent on Facebook (i.e., the average number of standard deviations of time spent that a respondent is above or below the running monthly average time spent for monthly active users), the number of days over the last 28 (as of September 23, 2020) that a user logged on the platform at least once (L28), their predicted ideology score, the number of days since they created their Facebook account, their inventory size (total number of posts created by their friends, Pages they follow, or groups they are members of), and the number of friends they have. Unless otherwise noted, all metrics are reported as of September 23, 2020. The column denoted by N indicates either the number of respondents or the proportion of adult monthly active users with a value on each metric. (Ideology predictions are not available for all U.S. users — see SI 1.3.)

Table 2: Activity on Facebook among sample compared to US monthly active users

Metric	Sample	p2.5	median	p97.5	mean	SD	N
Standardized time spent on FB	Respondents	-0.6	0.3	2.67	0.49	0.88	23377
	US MAP				0	1	100%
L28 as of 2020-09-23	Respondents	22	28	28	27.48	2.2	23377
	US MAP	2	28	28	23.4	7.96	100%
Predicted ideology score	Respondents	0.03	0.41	0.98	0.45	0.36	23377
	US MAP	0.04	0.61	0.98	0.57	0.3	100%
Days since FB account creation	Respondents	502	4263	5657	4011.06	1184.79	23361
	US MAP	135	3635	5247	3049.29	1538.3	100%
Inventory size	Respondents	159	2585	20708.8	4561.2	6354	23377
	US MAP	0	1119	15359	2757.11	5750.88	100%
Friend count as of 2020-09-23	Respondents	37	384	2505.95	578.19	666.04	23363
	US MAP	1	273	2751	494.85	697.14	100%

Table 3 compares the Facebook inferred ideology classifier to self-reported partisanship, ideology, and Trump approval. Figure 1 summarizes this analysis visually by showing how the binary predictions from our classifier (which we used during our intervention to determine the ideological alignment between study participants and sources they are exposed to — see Supplementary Information, section 4.2) correspond with binary aggregations of the same set of self-reported political characteristics from the survey. Figure 2 displays a similar comparison but instead disaggregates self-reported ideology and party identification into three categories and the predicted ideology score into ten different bins of width 0.10. Finally, Extended Data Figure 1 shows the distribution of the predicted ideology scores for the same three self-reported political characteristics. These analyses confirm that the ideology classifier is able to reliably distinguish liberals from conservatives and that it is well-calibrated. Additional details about the

ideology classifier and its performance can be found in Supplementary Information, section 1.3 and more information about how we use it to estimate the political alignment of sources is available in Supplementary Information, section 4.2.

Table 3: Distribution of Facebook ideology predictions in sample, by self-reported political characteristics

Variable	Category	% <0.50	% >=0.50	N
Party ID.	Strong Democrat	90.2	9.8	5875
Party ID.	Not very strong Democrat	77.7	22.3	3206
Party ID.	Independent, but closer to Democrat	80.6	19.4	3555
Party ID.	Independent	48.0	52.0	2897
Party ID.	Independent, but closer to Republican	16.7	83.3	2349
Party ID.	Not very strong Republican	23.5	76.5	2427
Party ID.	Strong Republican	7.0	93.0	3068
Ideology	Very liberal	92.8	7.2	3875
Ideology	Somewhat liberal	84.3	15.7	5746
Ideology	Middle of the road	50.5	49.5	7818
Ideology	Somewhat conservative	16.5	83.5	4125
Ideology	Very conservative	8.3	91.7	1809
Trump approval	Strongly disapprove	82.9	17.1	12850
Trump approval	Somewhat disapprove	51.6	48.4	2119
Trump approval	Neither approve nor disapprove	40.0	60.0	1261
Trump approval	Somewhat approve	19.4	80.6	3487
Trump approval	Strongly approve	7.9	92.1	3657

Figure 1: Confusion matrices comparing predicted ideology with self-reported ideology, party identification, and Trump approval

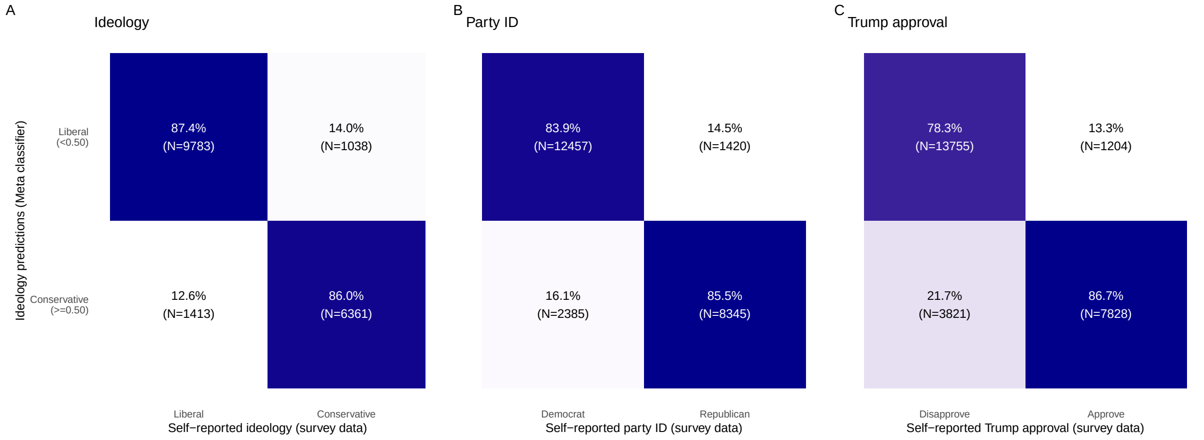

Confusion matrices comparing binary ideology predictions using Meta's classifier for Facebook (see Supplementary Information, section 1.3 for more details) with self-reported ideology, party identification, and Trump approval from surveys (Waves 1 and 2 prior to treatment). The percentages indicate column proportions; the percentages in the diagonal cells thus correspond to the recall statistic in a binary prediction task. Numbers in parentheses indicate the number of panelists included in each cell. The totals across matrices may not match due to missing values in the survey data.

Figure 2: Distribution of self-reported ideology, party identification, and Trump approval by predicted ideology

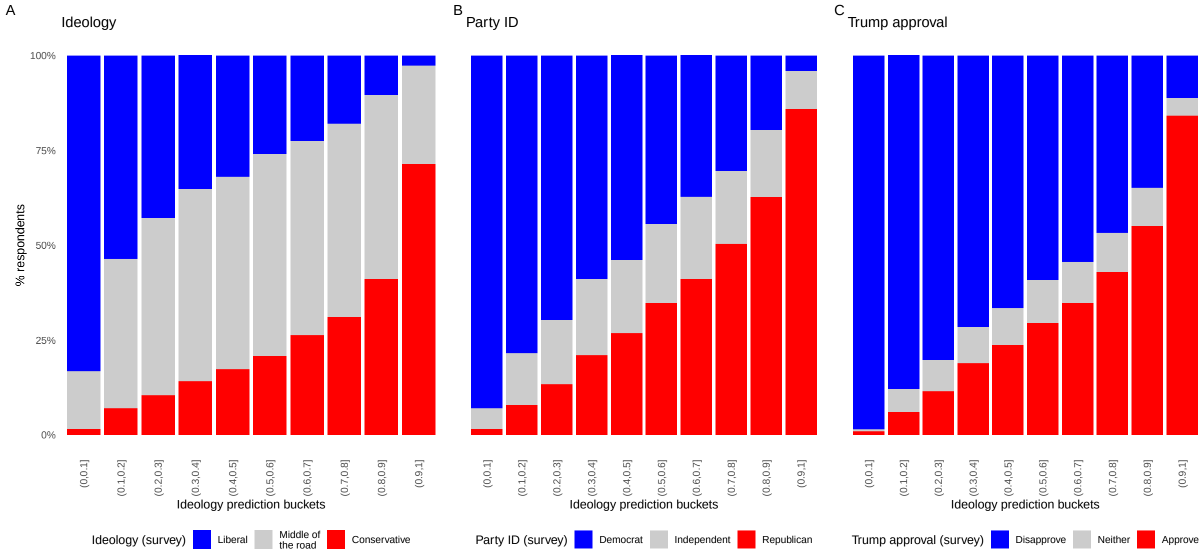

Each bar displays the distribution of respondents' answers to questions about their political characteristics in the survey by bin of predicted ideology. The bins are defined by the predicted ideology score (see Supplementary Information, section 1.3) and have a width of 0.10 on that scale.

Table 4 reports the proportion of participants in the treatment and control groups with one or more friends in the study, five or more friends in the study, and ten or more friends in the study as well as the mean number of friends in the study. (These statistics, unlike those reported elsewhere in the paper, include all participants, not just those who completed at least one post-treatment wave.) The data are computed using the Facebook friend graph as of January 2022.

Table 4: Number of friends in study

Condition	N	p(1+ friends)	p(5+ friends)	p(10+ friends)	Mean friends
Treatment group	8968	0.442	0.024	0.003	0.8
Control group	20072	0.444	0.027	0.004	0.9

2.1.2 Balance and attrition bias

This section examines randomization into the treatment and control condition, as well as whether attrition from the study varied by treatment status.

Table 5 compares participants in the treatment condition to those in the control group on numerous observable characteristics using permutation tests. These calculations include respondents who left the study prior to completing a post-treatment wave, but do not include those who withdrew from the study or deleted their accounts (see the following section for more details on withdrawals and account deletions).

Table 6 shows attrition by treatment status and quartile of pre-treatment exposure to content from like-minded sources in Wave 4 or 5.

Table 5: Distribution of observable characteristics by condition

	Control	Treatment	Diff.	p(diff!=0)
Battleground state	0.35	0.35	0.00	0.93
Not battleground state	0.65	0.65	-0.00	0.93
Low number of friends (first tercile)	0.33	0.33	0.00	0.89
Medium number of friends (second tercile)	0.33	0.34	-0.00	0.87
High number of friends (third tercile)	0.34	0.34	0.00	0.98
Non-white	0.28	0.28	-0.00	0.99
White	0.72	0.72	0.00	0.99
Democrat	0.52	0.52	0.00	0.99
Republican	0.35	0.35	-0.00	0.95
Independent	0.13	0.13	0.00	0.95
Age 18–29	0.19	0.19	-0.00	0.57
Age 30–44	0.42	0.42	-0.00	0.83
Age 45–65	0.33	0.32	0.00	0.53
Age 66+	0.07	0.07	0.00	0.91
Female gender	0.56	0.56	-0.00	0.85
Male gender	0.43	0.43	0.00	0.94
Other gender	0.01	0.01	0.00	0.49
College degree	0.48	0.48	0.01	0.29
No college degree	0.52	0.52	-0.01	0.29
Black, non-Hispanic	0.07	0.07	0.00	0.53
White, non-Hispanic	0.72	0.72	0.00	0.99
Hispanic	0.12	0.13	-0.01	0.16
Other	0.08	0.08	0.00	0.25
Low income (first tercile)	0.34	0.35	-0.01	0.02
Medium income (second tercile)	0.34	0.34	0.00	0.53
High income (third tercile)	0.32	0.31	0.01	0.08

The last column reports the unadjusted p-value for a two-sided t-test for the null hypothesis of no difference between treatment and control groups on each metric.

Table 6: Attrition rate by wave, treatment status, and quartile of pre-treatment exposure to content from like-minded sources

Wave	Treatment	Exposure quartile	Attrition rate	Sample size
4	0	1	0.174	4842
4	0	2	0.173	4894
4	0	3	0.177	4924
4	0	4	0.170	4883
4	1	1	0.163	2226
4	1	2	0.176	2174
4	1	3	0.168	2143
4	1	4	0.183	2185
5	0	1	0.273	4842
5	0	2	0.277	4894
5	0	3	0.277	4924
5	0	4	0.283	4883
5	1	1	0.266	2226
5	1	2	0.279	2174
5	1	3	0.272	2143
5	1	4	0.289	2185

2.1.3 Deleted accounts and study withdrawals

In total, 75,318 participants were randomized into one of the experimental conditions within the collaboration. Of these, 8 (0.01%) withdrew from the study after completing a post-treatment wave, and 1,369 (1.8%) deleted or deactivated their Facebook account between data collection and when the link between participant survey data and Facebook activity data was destroyed. Data from these participants are not included in the analyses in this paper.

2.2 Exposure

In this section, we present descriptive statistics for on-platform exposure metrics measured for monthly active Facebook users (which we define as the subset of U.S. adults who accessed Facebook at least once in the 30 days preceding August 17, 2020) and for the treatment and control groups in the field experiment. These statistics are measured during what we refer to as the pre-treatment period (the 90 days prior to the field experiment; June 26–September 22, 2020) or the treatment period (September 24–December 22, 2020 [the field experiment ended on December 23, 2020 but we only include full days in measuring these statistics]). In the paper, we analyze data from the population of monthly active Facebook users separately from the results of our field experiment. However, in the tables below, we often report data on both study participants and Facebook monthly (and, in some cases, daily) active users to allow the reader to compare our participants to the broader population of adult Facebook users in the U.S. as well as compare the control and treatment groups.

Unless otherwise noted, these tables report the distribution of user-level metrics. For example, the metric "% of like-minded friends" reported in Table 7, we first computed the proportion of like-minded friends for each participant or user and then reported the relevant statistics in the table. The average reported in the table therefore corresponds to the mean of the distribution of user-level proportions of like-minded friends.

Each table employs listwise deletion for the respondent sample so quantities can be easily compared within the table. As a result, the exact sample sizes vary by table depending on the prevalence of missing data. For the U.S. monthly active person results ("MAP"), we report the percentage of the population for which a value can be computed for each metric. Users are thus excluded if the denominator used to compute the metric is zero (e.g., in the first metric below if they do not have any friends).

Table 7 shows the total percentage of friends in the control group and treatment group that are like-minded and cross-cutting as of Sept. 22, 2020 (prior to treatment).

Table 7: Percentage of friends that are like-minded and cross-cutting

Metric	Group	p2.5	p50	p97.5	Avg.	SD	N	p(diff!=0)
% like-minded friends	Control	0	46.6	78.9	45.5	19.2	16049	–
	Treatment	0	46	78.1	45.2	19.2	7172	p=0.52
	US MAP	0	40.6	81.2	39	24.6	98%	–
% cross-cutting friends	Control	0	22.3	60.7	24.9	16.2	16049	–
	Treatment	0	22.4	59.9	24.9	15.9	7172	p=0.82
	US MAP	0	15.6	58	18.8	16.7	98%	–

156 observations (0.67%) dropped by listwise deletion. The last column reports the unadjusted p-value from a two-sided test of the hypothesis of no difference between treatment and control groups on each metric, computed using the baseline OLS model (see Section S1.5.1).

Table 8 shows the percentage of Pages and groups joined (prior to treatment) in the control

and treatment groups that are like-minded and cross-cutting.

Table 8: Percentage of Pages/groups followed that are like-minded or cross-cutting

Metric	Group	p2.5	p50	p97.5	Avg.	SD	N	p(diff!=0)
% Pages followed that are like-minded	Control	4.9	39.6	85	41.1	22.2	15933	–
	Treatment	4.8	39.1	85	40.8	22	7127	p=0.54
	US MAP	0	40.8	100	41.9	27.7	93.6%	–
% Pages followed that are cross-cutting	Control	0	10.4	53.9	15.1	14.6	15933	–
	Treatment	0	10.7	54.1	15.2	14.5	7127	p=0.75
	US MAP	0	7.1	75	15	20	93.6%	–
% groups joined that are like-minded	Control	0	41	93.8	42.4	28.2	15933	–
	Treatment	0	41.3	93.5	42.6	28.2	7127	p=0.34
	US MAP	0	41.7	100	43.2	33.7	89.6%	–
% groups joined that are cross-cutting	Control	0	7.7	71.4	16.5	20.5	15933	–
	Treatment	0	7.7	71.8	16.2	20.2	7127	p=0.36
	US MAP	0	1.1	82	13.3	22.2	89.6%	–

317 observations (1.36%) dropped by listwise deletion. The last column reports the unadjusted p-value from a two-sided test of the hypothesis of no difference between treatment and control groups on each metric, computed using the baseline OLS model (see Section S1.5.1).

Extended Data Table 4 shows the percentage of total content exposure that is like-minded or cross-cutting among the treatment and control groups for both the pre-treatment and treatment periods.

Table 9 shows the percentage of civic content exposure among the treatment and control groups that is like-minded or cross-cutting for both the pre-treatment and treatment periods.

Table 9: Percentage of civic content exposure that is like-minded or cross-cutting

Metric	Group	p2.5	p50	p97.5	Avg.	SD	N	p(diff!=0)
% like-minded civic exposure (pre)	Control	9.1	68	96.2	62.9	24.8	15992	–
	Treatment	8.4	67.4	96	62.5	24.9	7155	p=0.73
	US MAP	0	55.3	96.8	51.4	30.3	94.3%	–
% cross-cutting civic exposure (pre)	Control	0.2	12.7	70.3	19.1	19.1	15992	–
	Treatment	0.3	13	69.8	19.6	19.2	7155	p=0.18
	US MAP	0	12.5	78.9	20.5	22	94.3%	–
% like-minded civic exposure	Control	9.2	68.4	96.3	63.2	24.9	15992	–
	Treatment	4.6	46.4	92.5	47.3	25.5	7155	p=0.00
	US MAP	0	55.7	97.4	52.7	29.6	92.7%	–
% cross-cutting civic exposure	Control	0.2	12.4	71	19	19.2	15992	–
	Treatment	0.7	20.8	78	26.6	21.8	7155	p=0.00
	US MAP	0	14.1	77.7	21.2	21.6	92.7%	–

230 observations (0.98%) dropped by listwise deletion. The last column reports the unadjusted p-value from a two-sided test of the hypothesis of no difference between treatment and control groups on each metric, computed using the baseline OLS model (see Section S1.5.1).

Table 10 shows the percentage of news content that is like-minded or cross-cutting, for both the treatment and control groups, prior to the treatment period.

Table 10: Percentage of news content exposure that is like-minded or cross-cutting

Metric	Group	p2.5	p50	p97.5	Avg.	SD	N	p(diff!=0)
% like-minded news exposure (pre)	Control	6.9	56.8	94.7	55	25.3	15961	–
	Treatment	7	56.5	94.7	54.9	25.1	7136	p=0.68
	US MAP	0	47.1	95.2	46	29	93.2%	–
% cross-cutting news exposure (pre)	Control	0.2	11.6	63	17.3	17.1	15961	–
	Treatment	0.3	11.9	63.7	17.5	17.1	7136	p=0.66
	US MAP	0	10.9	75.5	18.2	20.5	93.2%	–
% like-minded news exposure	Control	6	55.8	95.2	54.3	25.7	15961	–
	Treatment	3.7	33.6	88.2	37.8	24	7136	p=0.00
	US MAP	0	45.9	96.3	46	28.5	91.2%	–
% cross-cutting news exposure	Control	0	11.2	64.4	17.1	17.4	15961	–
	Treatment	0.5	17	71.5	22.6	19.4	7136	p=0.00
	US MAP	0	11.5	74.8	18.4	20.1	91.2%	–

280 observations (1.2%) dropped by listwise deletion. The last column reports the unadjusted p-value from a two-sided test of the hypothesis of no difference between treatment and control groups on each metric, computed using the baseline OLS model (see Section S1.5.1).

Extended Data Figure 2 provides the pre-treatment distribution of exposure to sources that were like-minded, cross-cutting, or neither for all content (top row), civic content (middle row), and news content (bottom row) for both study participants, almost all of whom use Facebook every day (see Table 2), and U.S. adults who logged into Facebook every day in the 30 days preceding August 17, 2020. Extended Data Table 2 reports relevant statistics about these distributions for both groups. For instance, the median study participant received 55% of their content exposure from like-minded sources and 16.3% among cross-cutting sources versus 53% and 14.0%, respectively, among daily Facebook users. Exposure to 75% or more of content from like-minded sources was 20.4% and 21.1% among study participants and daily users, respectively.

Figure 3 presents the distribution of pre-treatment exposure by source type among study participants and U.S. adults who logged into Facebook at least once in the month prior to August 17, 2020. The spikes in the distributions of exposure to content from different types of sources at 0% and 100% for monthly active users are likely attributable to two major factors. First, some U.S.-based users are connected largely to non-U.S. people, groups, and Pages who do not have ideology scores. Second, since our usage threshold for inclusion in this analysis is relatively low, a subset of the users here may have been exposed to a small number of posts during this period (in some cases possibly just posts from a single group or Page or very few). Extended Data Table 1 reports relevant statistics about these distributions for both groups. Exposure to content from like-minded sources is somewhat lower among monthly users compared to daily users — the medians are 53% versus 50%, respectively.

Table 11 shows the percentage of civic content and news content exposure for study participants in the treatment and control groups as well as for monthly active users in both the pre-treatment and treatment periods.

Table 12 shows the percentage of like-minded and cross-cutting views of civic and news content for the treatment and control groups prior to the treatment period.

The next set of tables presents the mean, median, and the distribution of each of the 26 topic categories to which participants in the treatment and control groups were exposed, for content from like-minded and cross-cutting sources, both prior to and during the treatment periods. For example, of all the content from like-minded that participants in the control group saw in the pre-treatment period, an average of 4.1% of it was classified as being about "animals or pets" (see Table 13). Section 1.3 provides more details on the classifiers used in this study.

Figure 3: Pre-treatment exposure to Facebook Feed content by source type: Study participants and monthly Facebook users

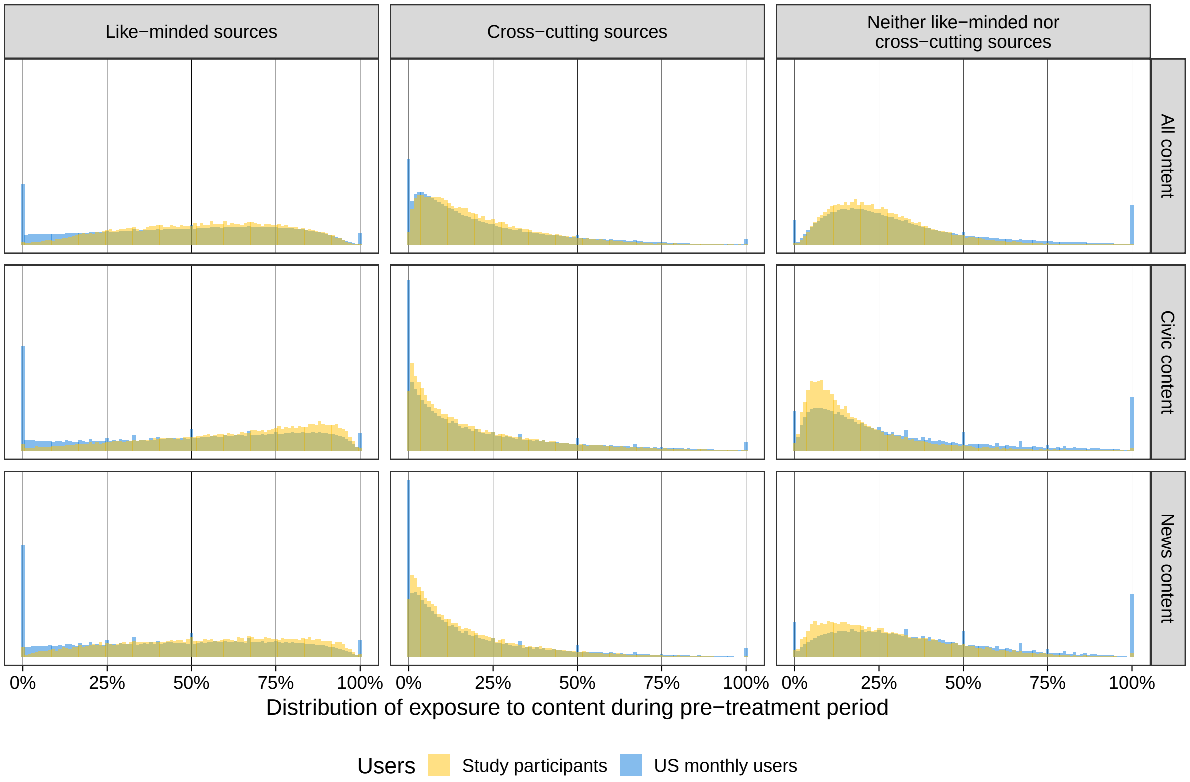

Distribution of Facebook Feed exposure to content from like-minded sources (left column), cross-cutting sources (center column), and those that fall into neither category (right column). Estimates presented for all content (top row) and for content classified as civic (i.e., political; center row) and news (bottom row). Source and content classifications were created using internal Facebook classifiers (see Supplementary Information, section 1.3). The graph includes the distribution of exposure for both study participants and the Facebook population of users age 18+ who logged into Facebook at least once in the month prior to August 17, 2020, when the study sampling frame was constructed.

Table 11: Exposure to civic and news content pre- and post-treatment by condition

Metric	Group	p2.5	p50	p97.5	Avg.	SD	N	p(diff!=0)
% civic content (pre-treatment)	Control	1.99	10.94	40.15	13.75	10.1	16046	–
	Treatment	1.97	10.83	40.01	13.61	10.15	7180	p=0.79
	US MAP	0	6.93	33.33	9.37	9.03	97.8%	–
% civic content (treatment)	Control	1.6	10.71	39.63	13.47	10.25	16046	–
	Treatment	1.76	10.31	37.52	12.79	9.47	7180	p=0.00
	US MAP	0	5.91	32.96	8.54	8.94	96.9%	–
% news content (pre-treatment)	Control	1.45	11.58	41.37	14.07	10.39	16046	–
	Treatment	1.46	11.32	40.46	13.92	10.57	7180	p=0.61
	US MAP	0	6.68	35.16	9.52	9.76	97.8%	–
% news content (treatment)	Control	0.78	8.22	36.31	10.85	9.41	16046	–
	Treatment	1.04	8.82	35.3	11.25	9.27	7180	p=0.00
	US MAP	0	4.39	30.03	7.18	8.44	96.9%	–

151 observations (0.65%) dropped by listwise deletion. The last column reports the unadjusted p-value from a two-sided test of the hypothesis of no difference between treatment and control groups on each metric, computed using the baseline OLS model (see Section S1.5.1).

Table 12: Pre-treatment exposure to civic and news content on Facebook

Metric	Group	p2.5	p50	p97.5	Avg.	SD	N	p(diff!=0)
% like-minded views on civic content (pre-treatment)	Control	1.69	13.25	50.52	16.92	13.11	16034	–
	Treatment	1.62	13.19	50.81	16.77	13.11	7164	p=0.96
% cross-cutting views on civic content (pre-treatment)	Control	0.84	7.93	34.8	10.4	8.96	16034	–
	Treatment	0.9	7.94	34.41	10.38	8.95	7164	p=0.80
% like-minded views on news content (pre-treatment)	Control	1.05	11.45	47.67	14.88	12.28	16034	–
	Treatment	1.13	11.19	47.29	14.89	12.56	7164	p=0.53
% cross-cutting views on news content (pre-treatment)	Control	0.39	7.67	41.64	10.83	10.64	16034	–
	Treatment	0.45	7.64	39.85	10.67	10.35	7164	p=0.39

179 observations (0.77%) dropped by listwise deletion. The last column reports the unadjusted p-value from a two-sided test of the hypothesis of no difference between treatment and control groups on each metric, computed using the baseline OLS model (see Section S1.5.1).

Table 13: Distribution of topics in content from like-minded sources to which respondents were exposed during pre-treatment period

Metric	Group	p2.5	p50	p97.5	Avg.	SD	N	p(diff!=0)
Animals & Pets	Control	0.5	3.2	14.3	4.1	4	16055	–
	Treatment	0.5	3.2	13.9	4.1	4.2	7176	p=0.59
Books & Literature	Control	0	0.4	2.1	0.5	0.8	16055	–
	Treatment	0	0.4	2.2	0.6	0.9	7176	p=0.23
Business, Finance & Economics	Control	0.3	1.3	3.7	1.5	1.1	16055	–
	Treatment	0.2	1.3	4	1.5	1.1	7176	p=0.07
Crime & Tragedy	Control	1.5	5.6	14.1	6.1	3.2	16055	–
	Treatment	1.4	5.6	13.8	6.1	3.2	7176	p=0.62
Education & Learning	Control	0.6	2.4	9.5	3.1	2.4	16055	–
	Treatment	0.6	2.4	9.7	3.1	2.7	7176	p=0.23
Fashion & Style	Control	0.6	2.7	10.9	3.5	3.1	16055	–
	Treatment	0.6	2.8	10.7	3.6	3	7176	p=0.96
Children & Parenting	Control	1.3	5.2	16.4	6.2	4.1	16055	–
	Treatment	1.4	5.4	15.7	6.2	4	7176	p=0.79
Fitness & Workouts	Control	0	0.4	2.2	0.6	0.9	16055	–
	Treatment	0	0.4	2.2	0.6	1.1	7176	p=0.74
Food & Drink	Control	0.8	3.5	10.7	4.1	2.9	16055	–
	Treatment	0.8	3.5	10.6	4	2.8	7176	p=0.67
Games, Puzzles & Play	Control	0.2	1	6	1.5	2.2	16055	–
	Treatment	0.2	1	6.1	1.5	2.1	7176	p=0.86
Health & Medical	Control	1.9	6	13.9	6.5	3.2	16055	–
	Treatment	1.9	6	14.3	6.6	3.5	7176	p=0.07
History & Philosophy	Control	0	0.2	1.2	0.3	0.5	16055	–
	Treatment	0	0.2	1.3	0.3	0.5	7176	p=0.82
Holidays & Celebrations	Control	0.4	1.8	6.3	2.2	1.9	16055	–
	Treatment	0.4	1.8	6.3	2.2	2.2	7176	p=0.80
Home & Garden	Control	0.7	3	12.1	3.9	3.3	16055	–
	Treatment	0.7	3.1	12	3.9	3.2	7176	p=0.75
Music & Audio	Control	0.5	2.2	9.1	2.9	2.7	16055	–
	Treatment	0.6	2.2	9.9	2.9	3.1	7176	p=0.23
Performing Arts	Control	0	0.3	1.4	0.4	0.5	16055	–
	Treatment	0	0.3	1.4	0.4	0.5	7176	p=0.70
Politics	Control	0.6	8.9	41.7	12.3	11.1	16055	–
	Treatment	0.7	8.8	41.3	12.1	11	7176	p=0.80
Relationships, Friends & Family	Control	0.9	4.2	17.8	5.5	4.5	16055	–
	Treatment	0.9	4.3	17.5	5.5	4.4	7176	p=0.43
Religion & Spirituality	Control	0.3	1.6	12.1	2.7	3.7	16055	–
	Treatment	0.3	1.7	11.9	2.8	3.8	7176	p=0.20
Science & Tech	Control	0.6	1.8	5.7	2.1	1.6	16055	–
	Treatment	0.5	1.8	5.7	2.1	1.6	7176	p=0.35
Social Issues	Control	0.8	6.7	22.6	8.1	5.8	16055	–
	Treatment	0.8	6.5	22.8	8.1	6	7176	p=0.58
Sports	Control	0	0.8	7.7	1.5	2.5	16055	–
	Treatment	0	0.8	7.5	1.5	2.6	7176	p=0.96
Travel & Leisure Activities	Control	0.4	1.9	6.9	2.3	1.9	16055	–
	Treatment	0.4	1.9	6.8	2.3	1.9	7176	p=0.56
TV & Movies	Control	1.1	4.3	14.4	5.2	3.7	16055	–
	Treatment	1.1	4.2	14.6	5.2	3.6	7176	p=0.44
Vehicles & Transportation	Control	0.9	2.6	14.7	3.8	4.3	16055	–
	Treatment	0.9	2.7	13.7	3.7	3.9	7176	p=0.22
Visual Arts, Architecture & Crafts	Control	0.9	2.8	9	3.4	2.4	16055	–
	Treatment	0.8	2.9	8.8	3.4	2.3	7176	p=0.46

146 observations (0.62%) dropped by listwise deletion. The last column reports the unadjusted p-value from a two-sided test of the hypothesis of no difference between treatment and control groups on each metric, computed using the baseline OLS model (see Section S1.5.1).

Table 14: Distribution of topics in content from cross-cutting sources to which respondents were exposed during pre-treatment period

Metric	Group	p2.5	p50	p97.5	Avg.	SD	N	p(diff!=0)
Animals & Pets	Control	0.3	3.9	20.4	5.3	5.8	16047	–
	Treatment	0.2	3.9	20.5	5.2	5.4	7172	p=0.35
Books & Literature	Control	0	0.3	2.1	0.5	0.9	16047	–
	Treatment	0	0.3	2.1	0.5	0.9	7172	p=0.81
Business, Finance & Economics	Control	0	1.1	4.1	1.4	1.4	16047	–
	Treatment	0	1.1	4.2	1.4	1.2	7172	p=0.52
Crime & Tragedy	Control	0.4	4.6	14	5.3	3.6	16047	–
	Treatment	0.3	4.6	13.1	5.2	3.4	7172	p=0.50
Education & Learning	Control	0	2.4	9.9	3.1	2.8	16047	–
	Treatment	0	2.4	10.1	3.1	2.9	7172	p=0.65
Fashion & Style	Control	0	2.9	11.3	3.6	3.3	16047	–
	Treatment	0	2.9	11.7	3.6	3.3	7172	p=0.98
Children & Parenting	Control	1	6.5	19.2	7.4	4.8	16047	–
	Treatment	1.1	6.5	18.9	7.4	4.6	7172	p=0.96
Fitness & Workouts	Control	0	0.5	2.9	0.7	1.5	16047	–
	Treatment	0	0.5	2.8	0.7	1.4	7172	p=0.74
Food & Drink	Control	0.2	4	13.6	4.7	3.8	16047	–
	Treatment	0.2	4.1	13	4.7	3.9	7172	p=0.84
Games, Puzzles & Play	Control	0	1.1	5.8	1.6	2.6	16047	–
	Treatment	0	1.1	6.8	1.7	2.8	7172	p=0.10
Health & Medical	Control	0.9	5.3	14.7	5.9	3.7	16047	–
	Treatment	1	5.3	15	6	3.8	7172	p=0.44
History & Philosophy	Control	0	0.2	1.7	0.3	0.9	16047	–
	Treatment	0	0.2	1.7	0.3	0.9	7172	p=0.93
Holidays & Celebrations	Control	0.2	2.3	8.5	2.8	2.8	16047	–
	Treatment	0.2	2.3	8.5	2.8	2.5	7172	p=0.26
Home & Garden	Control	0.2	3.5	14.6	4.4	3.9	16047	–
	Treatment	0.1	3.4	14.9	4.3	3.9	7172	p=0.55
Music & Audio	Control	0	2.2	9.9	2.9	3.3	16047	–
	Treatment	0	2.1	11	2.9	3.6	7172	p=0.36
Performing Arts	Control	0	0.2	1.5	0.4	0.6	16047	–
	Treatment	0	0.3	1.5	0.4	0.8	7172	p=0.68
Politics	Control	0	4.6	25.4	6.7	7	16047	–
	Treatment	0	4.6	25	6.6	7	7172	p=0.97
Relationships, Friends & Family	Control	0.5	5	18.3	6.1	4.6	16047	–
	Treatment	0.5	5	17.9	6.1	4.6	7172	p=0.56
Religion & Spirituality	Control	0	1.6	11.9	2.5	4	16047	–
	Treatment	0	1.5	12.4	2.6	4.4	7172	p=0.20
Science & Tech	Control	0	1.8	6.7	2.1	2.2	16047	–
	Treatment	0	1.8	6.9	2.2	2.1	7172	p=0.74
Social Issues	Control	0	4.3	17.4	5.4	4.6	16047	–
	Treatment	0.2	4.3	17.2	5.4	4.5	7172	p=0.77
Sports	Control	0	0.9	10	1.8	3.3	16047	–
	Treatment	0	0.9	10.1	1.8	3.6	7172	p=0.83
Travel & Leisure Activities	Control	0	2.3	9.1	2.9	2.7	16047	–
	Treatment	0	2.3	8.8	2.9	2.6	7172	p=0.61
TV & Movies	Control	0	4	16.2	5.1	4.3	16047	–
	Treatment	0	4.1	15.8	5.1	4.5	7172	p=0.68
Vehicles & Transportation	Control	0.2	3.1	13.9	4	4.5	16047	–
	Treatment	0.2	3.1	12.9	3.9	4	7172	p=0.04
Visual Arts, Architecture & Crafts	Control	0	2.9	10.4	3.5	3	16047	–
	Treatment	0	3	10.9	3.6	3.4	7172	p=0.08

158 observations (0.68%) dropped by listwise deletion. The last column reports the unadjusted p-value from a two-sided test of the hypothesis of no difference between treatment and control groups on each metric, computed using the baseline OLS model (see Section S1.5.1).

Table 15: Distribution of topics in content from like-minded sources to which respondents were exposed during study period

Metric	Group	p2.5	p50	p97.5	Avg.	SD	N	p(diff!=0)
Animals & Pets	Control	0.6	3.6	16.2	4.6	4.6	16033	–
	Treatment	0.4	3.7	14.8	4.5	4.2	7168	p=0.30
Books & Literature	Control	0	0.5	2.8	0.7	1	16033	–
	Treatment	0	0.5	2.7	0.7	1.3	7168	p=0.28
Business, Finance & Economics	Control	0.2	1.2	4	1.4	1.2	16033	–
	Treatment	0	1.2	4.4	1.5	1.7	7168	p=0.01
Crime & Tragedy	Control	0.6	2.7	8.4	3.1	2.2	16033	–
	Treatment	0.5	2.7	8	3.1	2	7168	p=0.21
Education & Learning	Control	0.3	1.6	6.6	2	1.9	16033	–
	Treatment	0	1.6	6.5	2	2.3	7168	p=0.74
Fashion & Style	Control	0.7	3.1	12.6	4.1	3.6	16033	–
	Treatment	0.5	3.2	12.1	4.1	3.6	7168	p=0.37
Children & Parenting	Control	1.7	6.6	20.6	7.8	5	16033	–
	Treatment	1.6	6.9	20.8	7.9	5.1	7168	p=0.02
Fitness & Workouts	Control	0	0.4	2.1	0.6	0.9	16033	–
	Treatment	0	0.4	2.4	0.6	1.1	7168	p=0.11
Food & Drink	Control	0.9	3.8	12.8	4.6	3.7	16033	–
	Treatment	0.6	3.8	11.6	4.4	3.4	7168	p=0.00
Games, Puzzles & Play	Control	0.2	1.2	6.8	1.7	2.5	16033	–
	Treatment	0	1.2	6.7	1.7	2.4	7168	p=0.35
Health & Medical	Control	1.6	5.5	14.3	6.2	3.5	16033	–
	Treatment	1.6	5.5	15	6.3	3.9	7168	p=0.00
History & Philosophy	Control	0	0.2	1.6	0.3	0.8	16033	–
	Treatment	0	0.2	1.5	0.3	0.6	7168	p=0.02
Holidays & Celebrations	Control	1.6	5.2	13.9	5.9	3.6	16033	–
	Treatment	1.4	5.4	14.2	6.1	3.7	7168	p=0.01
Home & Garden	Control	1	3.9	15.8	5	4.1	16033	–
	Treatment	0.8	3.8	14.2	4.7	3.6	7168	p=0.00
Music & Audio	Control	0.5	2.3	10.6	3.1	3.3	16033	–
	Treatment	0.3	2.5	11.1	3.3	3.4	7168	p=0.00
Performing Arts	Control	0	0.2	1.4	0.3	0.6	16033	–
	Treatment	0	0.3	1.5	0.4	0.8	7168	p=0.00
Politics	Control	0.5	8.8	45.6	12.7	12.2	16033	–
	Treatment	0.7	9.5	46	13.4	12.3	7168	p=0.00
Relationships, Friends & Family	Control	1.3	5.8	21.4	7.2	5.3	16033	–
	Treatment	1.2	5.9	21.3	7.2	5.5	7168	p=0.70
Religion & Spirituality	Control	0.2	1.6	13.4	2.9	4.3	16033	–
	Treatment	0	1.6	12.2	2.8	3.9	7168	p=0.03
Science & Tech	Control	0.5	2.1	7.7	2.6	2.3	16033	–
	Treatment	0.3	2.1	7.6	2.5	2.2	7168	p=0.01
Social Issues	Control	0.7	7.3	31.1	9.8	8.3	16033	–
	Treatment	0.9	7.6	31.5	10.1	8.4	7168	p=0.00
Sports	Control	0	0.7	9.6	1.6	3.2	16033	–
	Treatment	0	0.8	9	1.6	3	7168	p=0.88
Travel & Leisure Activities	Control	0.3	1.7	6.8	2.1	2.2	16033	–
	Treatment	0.1	1.7	6.6	2.1	2	7168	p=0.38
TV & Movies	Control	1.1	4.7	16.2	5.7	4.1	16033	–
	Treatment	0.8	4.6	15.6	5.6	3.9	7168	p=0.00
Vehicles & Transportation	Control	0.7	2.3	16.8	3.8	5.3	16033	–
	Treatment	0.5	2.4	14.9	3.6	4.5	7168	p=0.01
Visual Arts, Architecture & Crafts	Control	1.2	4.1	12	4.7	3	16033	–
	Treatment	1	3.9	10.8	4.5	3.3	7168	p=0.00

176 observations (0.75%) dropped by listwise deletion. The last column reports the unadjusted p-value from a two-sided test of the hypothesis of no difference between treatment and control groups on each metric, computed using the baseline OLS model (see Section S1.5.1).

Table 16: Distribution of topics in content from cross-cutting sources to which respondents were exposed during study period

Metric	Group	p2.5	p50	p97.5	Avg.	SD	N	p(diff!=0)
Animals & Pets	Control	0	4.2	22.8	5.8	6.4	16005	–
	Treatment	0.5	4.2	22.2	5.7	6.1	7166	p=0.35
Books & Literature	Control	0	0.4	2.9	0.7	1.3	16005	–
	Treatment	0	0.5	2.9	0.7	1.3	7166	p=0.06
Business, Finance & Economics	Control	0	1	4.2	1.3	1.8	16005	–
	Treatment	0	1	4.1	1.3	1.3	7166	p=0.49
Crime & Tragedy	Control	0	2.2	9.7	2.8	2.9	16005	–
	Treatment	0	2.3	10.2	2.9	2.8	7166	p=0.00
Education & Learning	Control	0	1.5	6.8	2	2.4	16005	–
	Treatment	0	1.5	6.8	2	2.3	7166	p=0.43
Fashion & Style	Control	0	3.3	13.4	4.2	3.8	16005	–
	Treatment	0.4	3.3	13.1	4.1	3.6	7166	p=0.20
Children & Parenting	Control	1.4	8.2	23.7	9.4	6	16005	–
	Treatment	1.6	7.9	21.3	8.8	5.3	7166	p=0.00
Fitness & Workouts	Control	0	0.4	2.9	0.7	1.3	16005	–
	Treatment	0	0.4	2.7	0.7	1.7	7166	p=0.48
Food & Drink	Control	0	4.3	16.1	5.2	4.7	16005	–
	Treatment	0.5	4.3	16.7	5.3	4.5	7166	p=0.21
Games, Puzzles & Play	Control	0	1.3	7.3	1.9	3.2	16005	–
	Treatment	0	1.3	7.7	1.9	3.1	7166	p=0.28
Health & Medical	Control	0.7	4.7	14.9	5.5	4	16005	–
	Treatment	1	4.7	14.3	5.4	3.7	7166	p=0.31
History & Philosophy	Control	0	0.1	2.2	0.4	1	16005	–
	Treatment	0	0.2	2.1	0.4	1.2	7166	p=0.07
Holidays & Celebrations	Control	1.4	6.8	17.5	7.4	4.4	16005	–
	Treatment	1.6	6.4	15.4	7	3.8	7166	p=0.00
Home & Garden	Control	0.3	4.4	18.7	5.6	5	16005	–
	Treatment	0.6	4.3	19	5.6	5	7166	p=0.70
Music & Audio	Control	0	2.2	11.8	3.1	3.8	16005	–
	Treatment	0.2	2.4	12.2	3.3	3.8	7166	p=0.01
Performing Arts	Control	0	0.2	1.4	0.3	0.8	16005	–
	Treatment	0	0.2	1.5	0.4	0.9	7166	p=0.00
Politics	Control	0	4.4	27.7	6.7	7.7	16005	–
	Treatment	0.2	4.7	28	7.1	7.7	7166	p=0.00
Relationships, Friends & Family	Control	0.9	6.9	22.6	8	5.6	16005	–
	Treatment	1	6.6	21	7.7	5.2	7166	p=0.00
Religion & Spirituality	Control	0	1.5	13.4	2.7	4.4	16005	–
	Treatment	0	1.6	14.1	2.9	4.9	7166	p=0.00
Science & Tech	Control	0	2	8.7	2.6	3	16005	–
	Treatment	0	2	9.3	2.6	3	7166	p=0.27
Social Issues	Control	0	4.2	22.4	5.9	6.1	16005	–
	Treatment	0.5	4.5	22.6	6.3	6	7166	p=0.00
Sports	Control	0	0.9	11.7	2	4	16005	–
	Treatment	0	0.9	11.6	2	4	7166	p=0.51
Travel & Leisure Activities	Control	0	2	9.2	2.6	2.9	16005	–
	Treatment	0	2	8.8	2.6	2.5	7166	p=0.09
TV & Movies	Control	0	4.4	17.8	5.6	4.8	16005	–
	Treatment	0.7	4.5	17.6	5.7	4.8	7166	p=0.11
Vehicles & Transportation	Control	0	2.7	14.5	3.8	5.3	16005	–
	Treatment	0.4	2.7	14	3.8	4.8	7166	p=0.39
Visual Arts, Architecture & Crafts	Control	0.4	4.2	14.3	5	3.9	16005	–
	Treatment	0.8	4.3	15.1	5.2	4.2	7166	p=0.00

206 observations (0.88%) dropped by listwise deletion. The last column reports the unadjusted p-value from a two-sided test of the hypothesis of no difference between treatment and control groups on each metric, computed using the baseline OLS model (see Section S1.5.1).

Table 17: Percentage of content exposure (by topic) that is from like-minded sources during pre-treatment period

Metric	Group	p2.5	p50	p97.5	Avg.	SD	N	p(diff!=0)
Animals & Pets	Control	7.6	49.4	91.7	49.6	23.5	15115	–
	Treatment	7.7	49	91.3	49.2	23.4	6765	p=0.50
	US MAP	2.9	47.8	100	48	26.3	87.4%	–
Books & Literature	Control	3.7	56.1	100	54.5	26.6	15115	–
	Treatment	4.6	55.9	98.5	54.3	26.4	6765	p=0.90
	US MAP	2.4	56.2	100	55.3	28.8	72.9%	–
Business, Finance & Economics	Control	7.4	55.6	94	54	24.5	15115	–
	Treatment	7.4	55.6	93.8	53.8	24.6	6765	p=0.89
	US MAP	1.8	54.5	100	52.7	27.7	82.3%	–
Crime & Tragedy	Control	10.5	63.1	95.7	59.9	24.5	15115	–
	Treatment	10.3	62.8	95.4	59.6	24.3	6765	p=0.95
	US MAP	1.4	60.5	100	56.4	28.7	88.2%	–
Education & Learning	Control	9.9	58.1	93.4	56.1	23.6	15115	–
	Treatment	9.9	57.7	93	55.8	23.5	6765	p=0.76
	US MAP	2.2	54.6	100	52.8	27	85.6%	–
Fashion & Style	Control	6.8	52.8	93	51.9	24.7	15115	–
	Treatment	6.8	52.5	92.9	51.5	24.7	6765	p=0.51
	US MAP	0.8	51.1	100	49.9	28.2	87.8%	–
Children & Parenting	Control	11.2	53.5	91.2	52.9	22.3	15115	–
	Treatment	10.6	53.6	90.5	52.6	22.1	6765	p=0.81
	US MAP	2.6	52.3	95.4	50.8	26.1	90.1%	–
Fitness & Workouts	Control	4.4	50	95.3	50.1	25.6	15115	–
	Treatment	3.9	50	95	49.7	25.6	6765	p=0.45
	US MAP	1.7	50	100	50.5	27.9	79.3%	–
Food & Drink	Control	6.9	50.4	92.2	50.2	24.3	15115	–
	Treatment	6.2	49.9	91.8	49.6	24.4	6765	p=0.18
	US MAP	1.5	50	100	49.3	27.7	87.7%	–
Games, Puzzles & Play	Control	4.8	49.1	93.8	49.1	25.6	15115	–
	Treatment	3.9	48.5	93.3	48.7	25.7	6765	p=0.57
	US MAP	1.1	50	100	50.1	28.5	83.1%	–
Health & Medical	Control	9.8	57.7	92.9	55.8	23.4	15115	–
	Treatment	9.7	57.2	92.5	55.3	23.4	6765	p=0.50
	US MAP	1.2	51.8	94.2	50.1	26.5	88.8%	–
History & Philosophy	Control	0	56.5	100	54.6	28.9	15115	–
	Treatment	0	55.6	100	54.1	29	6765	p=0.47
	US MAP	2.9	61.9	100	59	30.5	68.3%	–
Holidays & Celebrations	Control	11.4	54.5	91.1	53.8	22.3	15115	–
	Treatment	11.1	55	90.7	53.5	22.3	6765	p=0.64
	US MAP	3.1	54.9	100	53	26.7	88.1%	–
Home & Garden	Control	5.5	51.7	93.6	50.8	25.8	15115	–
	Treatment	5.3	51.7	93.3	50.5	25.9	6765	p=0.67
	US MAP	1.9	52	100	51	28.7	87.4%	–
Music & Audio	Control	8.8	56.6	93.2	55.2	23.4	15115	–
	Treatment	9.3	56.3	92.9	54.8	23.4	6765	p=0.42
	US MAP	0.9	53.4	100	51.4	27.6	86.6%	–
Performing Arts	Control	3.1	56.7	100	55.2	25.9	15115	–
	Treatment	1.1	55.9	100	54.8	26.1	6765	p=0.56
	US MAP	0	51.9	100	51.7	28.3	76.3%	–
Politics	Control	10.2	73.3	97.5	66.8	24.9	15115	–
	Treatment	9.8	72.7	97.4	66.5	24.8	6765	p=0.84
	US MAP	0.7	65.7	100	59.5	29.4	86.6%	–
Relationships, Friends & Family	Control	10.8	55.3	92.2	54.4	22.6	15115	–
	Treatment	10.1	54.7	91.5	53.8	22.5	6765	p=0.20
	US MAP	1.6	52.8	97.5	51	26.9	89.9%	–
Religion & Spirituality	Control	8.4	63	96.5	59.5	25.4	15115	–
	Treatment	8.7	63.2	96.2	59.4	25.2	6765	p=0.70

	US MAP	1.5	60	100	56.4	30.1	86.6%	–
Science & Tech	Control	8.1	52.9	92.8	52.2	23.8	15115	–
	Treatment	8	52.5	92.2	51.7	23.6	6765	p=0.31
	US MAP	1.6	51.9	100	51.1	27.3	84.5%	–
Social Issues	Control	10.2	66.7	95.8	62.1	24.3	15115	–
	Treatment	9.7	65.8	95.5	61.7	24.2	6765	p=0.66
	US MAP	0.8	56.3	96.4	53.2	28	87%	–
Sports	Control	4.6	50	95.9	49.9	27	15115	–
	Treatment	4.8	50	95.2	49.9	26.7	6765	p=0.59
	US MAP	1.9	50	100	50.2	29.2	82.6%	–
Travel & Leisure Activities	Control	9	51.3	91	51.1	22.8	15115	–
	Treatment	8.9	50.9	90.2	50.5	22.5	6765	p=0.17
	US MAP	2.6	51.7	100	51	26.3	86.8%	–
TV & Movies	Control	8.3	53	92.8	52.4	23.6	15115	–
	Treatment	8.6	52.3	92.5	51.9	23.4	6765	p=0.60
	US MAP	1	50	100	49.3	27.2	86.9%	–
Vehicles & Transportation	Control	6.9	54.7	94.1	53.2	25.5	15115	–
	Treatment	6.3	54.5	93.6	52.8	25.5	6765	p=0.48
	US MAP	2	55.2	100	53.1	28.8	87.9%	–
Visual Arts, Architecture & Crafts	Control	8.8	53.5	92.7	52.7	23.6	15115	–
	Treatment	8.2	52.9	91.9	52.1	23.5	6765	p=0.27
	US MAP	1.7	50	100	49.6	27.1	86.9%	–

1497 observations (6.4%) dropped by listwise deletion. The last column reports the unadjusted p-value from a two-sided test of the hypothesis of no difference between treatment and control groups on each metric, computed using the baseline OLS model (see Section S1.5.1).

Table 18: Percentage of content exposure (by topic) that is from cross-cutting sources during pre-treatment period

Metric	Group	p2.5	p50	p97.5	Avg.	SD	N	p(diff!=0)
Animals & Pets	Control	0.9	17.7	67.8	22.4	18	15115	–
	Treatment	0.9	18.1	67.1	22.8	18	6765	p=0.19
	US MAP	0.1	16.9	78.6	22.9	20.7	84.9%	–
Books & Literature	Control	0	14.3	74.1	20.7	20.4	15115	–
	Treatment	0	14.6	72.5	20.7	20.1	6765	p=0.84
	US MAP	0	20.5	100	28.5	25.6	63.2%	–
Business, Finance & Economics	Control	0	14	66.3	19.4	17.8	15115	–
	Treatment	0	14.3	66.1	19.6	17.8	6765	p=0.66
	US MAP	0	15.8	85.7	23.4	22.6	76.9%	–
Crime & Tragedy	Control	0.5	15.3	71.9	21.4	19.6	15115	–
	Treatment	0.5	15.7	70.4	21.7	19.4	6765	p=0.40
	US MAP	0	15.3	84.8	23.5	23.3	85.6%	–
Education & Learning	Control	0.5	16.5	67.7	21.5	18.2	15115	–
	Treatment	0.6	16.9	67.7	21.7	18.1	6765	p=0.56
	US MAP	0	17.4	82	24.1	21.9	82.1%	–
Fashion & Style	Control	0.4	15.6	69	21.1	18.6	15115	–
	Treatment	0.5	15.6	69.4	21.2	18.7	6765	p=0.80
	US MAP	0	15.3	83.3	22.8	22.4	85.3%	–
Children & Parenting	Control	1.3	19.5	65.6	23.4	17.5	15115	–
	Treatment	1.4	19.6	65.1	23.6	17.5	6765	p=0.56
	US MAP	0.4	17.8	76.6	23.8	20.7	88.2%	–
Fitness & Workouts	Control	0	16.2	71.4	21.8	19.8	15115	–
	Treatment	0	16	71.4	21.6	19.6	6765	p=0.50
	US MAP	0	17.2	94.5	24.8	23.4	72.8%	–
Food & Drink	Control	0.7	16.6	68	21.8	18.3	15115	–
	Treatment	0.8	16.8	68.3	21.8	18.2	6765	p=0.86
	US MAP	0.2	15.6	82.2	22.9	22	85.1%	–
Games, Puzzles & Play	Control	0	14.7	69.2	20.4	18.8	15115	–
	Treatment	0	14.7	70.2	20.6	19	6765	p=0.54
	US MAP	0	15.6	86.9	23.1	22.7	77.9%	–
Health & Medical	Control	0.6	14.9	64.4	19.7	17.1	15115	–
	Treatment	0.7	15.3	63.3	20.1	17.2	6765	p=0.22
	US MAP	0	15.6	75	21.9	20.3	86.6%	–
History & Philosophy	Control	0	12.5	82.7	20.3	22.5	15115	–
	Treatment	0	13	81.8	20.8	22.6	6765	p=0.23
	US MAP	0	20	100	29.3	27.9	55.5%	–
Holidays & Celebrations	Control	1.3	20.8	67.5	24.9	18.2	15115	–
	Treatment	1.4	20.6	68.2	25	18.2	6765	p=0.73
	US MAP	0.4	18.7	80.3	25	21.6	85.4%	–
Home & Garden	Control	0.5	16	74.9	22.4	20.3	15115	–
	Treatment	0.6	16.2	74	22.5	20.1	6765	p=0.94
	US MAP	0.1	14.9	84.3	22.7	22.5	84.4%	–
Music & Audio	Control	0.5	16.3	66.7	21.2	18.1	15115	–
	Treatment	0.6	16.6	67.2	21.3	18	6765	p=0.79
	US MAP	0	16.7	84.4	23.9	22.7	83.8%	–
Performing Arts	Control	0	16.7	74.3	22	20.6	15115	–
	Treatment	0	16.5	75	22.2	20.8	6765	p=0.75
	US MAP	0	20	100	27.2	24.6	70.2%	–
Politics	Control	0.2	11.9	74.4	19.3	20.3	15115	–
	Treatment	0.2	12.3	74.1	19.8	20.4	6765	p=0.15
	US MAP	0	15	88.7	24.1	24.8	83.6%	–
Relationships, Friends & Family	Control	0.9	19.7	66.5	23.6	18	15115	–
	Treatment	1	19.7	67	23.7	18	6765	p=0.86
	US MAP	0.1	18.2	80	24.4	21.6	88%	–
Religion & Spirituality	Control	0	17	76.8	23.5	21.3	15115	–
	Treatment	0	16.7	75.2	23.3	21	6765	p=0.40

	US MAP	0	15.4	88.5	24	24.1	82.3%	–
Science & Tech	Control	0.3	15.2	65.2	20.1	17.4	15115	–
	Treatment	0.4	15.5	63.9	20.2	17.2	6765	p=0.65
	US MAP	0	15.9	83.3	22.9	21.8	80.2%	–
Social Issues	Control	0.3	13	69.2	19.3	18.8	15115	–
	Treatment	0.3	13.7	68.7	19.8	18.8	6765	p=0.14
	US MAP	0	15.1	80	22.4	21.9	84.5%	–
Sports	Control	0	15	75	21.9	20.8	15115	–
	Treatment	0	15.3	73.7	22.2	20.9	6765	p=0.35
	US MAP	0	13.6	87.5	22.2	23.1	77.1%	–
Travel & Leisure Activities	Control	0.9	18.7	67.5	23.1	17.9	15115	–
	Treatment	1	18.9	66	23.4	17.8	6765	p=0.27
	US MAP	0	18.2	80	24.3	21.3	83.7%	–
TV & Movies	Control	0.5	14.9	62.9	19.6	16.9	15115	–
	Treatment	0.5	15.4	63.1	19.8	16.9	6765	p=0.43
	US MAP	0	15.8	83.3	23	22.2	84.5%	–
Vehicles & Transportation	Control	0.6	16.7	75.3	23.3	20.7	15115	–
	Treatment	0.8	16.9	74.1	23.4	20.5	6765	p=0.71
	US MAP	0	15	83.3	23	22.7	85%	–
Visual Arts, Architecture & Crafts	Control	0.5	16.1	66.1	21	17.8	15115	–
	Treatment	0.6	16.4	65.6	21.3	17.7	6765	p=0.44
	US MAP	0	16.3	81.8	22.9	21.6	84.1%	–

1497 observations (6.4%) dropped by listwise deletion. The last column reports the unadjusted p-value from a two-sided test of the hypothesis of no difference between treatment and control groups on each metric, computed using the baseline OLS model (see Section S1.5.1).

Table 19: Percentage of content exposure (by topic) that is from like-minded sources during study period

Metric	Group	p2.5	p50	p97.5	Avg.	SD	N	p(diff!=0)
Animals & Pets	Control	7.3	49.9	91.9	49.9	23.8	14877	–
	Treatment	3.6	29.6	82.9	33.9	21.9	6653	p=0.00
	US MAP	3.5	47.8	100	48.5	26.4	87.1%	–
Books & Literature	Control	4.5	54.2	96.9	53.1	25.9	14877	–
	Treatment	0	31.2	88.2	35.4	24.3	6653	p=0.00
	US MAP	2.3	52.7	100	53	28.8	74.8%	–
Business, Finance & Economics	Control	7.1	55.1	94.7	53.5	24.9	14877	–
	Treatment	3.3	34.5	87.5	38	23.8	6653	p=0.00
	US MAP	2.2	52.8	100	52.2	27.9	81.8%	–
Crime & Tragedy	Control	7.9	59.3	95.4	56.8	25.3	14877	–
	Treatment	3.8	36.4	90.6	39.8	24.7	6653	p=0.00
	US MAP	1.6	55.8	100	54.3	28.6	86%	–
Education & Learning	Control	8.8	56.4	93.6	54.8	24.1	14877	–
	Treatment	4.1	35.6	86.5	38.8	23	6653	p=0.00
	US MAP	2.7	53.1	100	52.4	27.4	83.9%	–
Fashion & Style	Control	6.6	51.4	92.6	50.9	24.7	14877	–
	Treatment	3.5	30.7	84.1	35	22.6	6653	p=0.00
	US MAP	1.2	49.3	100	48.8	27.7	88%	–
Children & Parenting	Control	11.1	53.1	90.8	52.5	22.2	14877	–
	Treatment	5.7	33.9	82.5	37.2	21	6653	p=0.00
	US MAP	3.5	50.9	95.3	50.5	25.7	90.5%	–
Fitness & Workouts	Control	3.8	49.6	95.5	49.3	26	14877	–
	Treatment	0	29.6	87.2	34.2	24	6653	p=0.00
	US MAP	1.7	48.6	100	49	27.8	78.4%	–
Food & Drink	Control	6.3	50.4	92.6	50.1	24.5	14877	–
	Treatment	3.1	28.7	82.3	33.1	22.1	6653	p=0.00
	US MAP	1.9	49.2	100	48.9	27.7	87.4%	–
Games, Puzzles & Play	Control	4.2	48	93.8	48.5	26	14877	–
	Treatment	1.7	27.2	84.4	32.5	23.2	6653	p=0.00
	US MAP	1.6	50	100	49.7	28.7	83%	–
Health & Medical	Control	9.6	57.5	93.3	55.5	23.7	14877	–
	Treatment	5	35.9	86.8	39.3	22.9	6653	p=0.00
	US MAP	2.2	51.4	95.4	50.6	26.4	88.2%	–
History & Philosophy	Control	0	55.1	100	53.9	29.5	14877	–
	Treatment	0	30.6	100	36.5	28.6	6653	p=0.00
	US MAP	2.9	60	100	58.5	30.7	66.9%	–
Holidays & Celebrations	Control	11.9	54.6	90.8	53.6	22.1	14877	–
	Treatment	6.4	35.9	82.9	38.5	21.1	6653	p=0.00
	US MAP	3.9	53.4	96.9	52.3	26.2	90.3%	–
Home & Garden	Control	5.4	51	93.4	50.3	26	14877	–
	Treatment	2.8	28.8	87.7	34.1	23.9	6653	p=0.00
	US MAP	2.3	50	100	50.4	28.6	87.7%	–
Music & Audio	Control	9.3	56.3	93.1	55.2	23.2	14877	–
	Treatment	4.6	36	83.7	39	22.1	6653	p=0.00
	US MAP	1.4	52.7	100	51.4	27.5	86.1%	–
Performing Arts	Control	0	54.8	100	54	26.7	14877	–
	Treatment	0	36.4	91.8	39.1	25.4	6653	p=0.00
	US MAP	0	50	100	50.8	28.3	74.8%	–
Politics	Control	9.9	73.6	97.6	67.1	25	14877	–
	Treatment	5.5	52.9	95	52	26.7	6653	p=0.00
	US MAP	1.6	65.1	100	60.1	28.9	85.8%	–
Relationships, Friends & Family	Control	11.5	55.7	92.1	54.6	22.4	14877	–
	Treatment	5.9	36.2	84.9	39.1	21.8	6653	p=0.00
	US MAP	2.3	52	96.5	51	26.5	90.5%	–
Religion & Spirituality	Control	8.1	63.4	97	59.6	25.8	14877	–
	Treatment	3.2	40.4	93.4	43.2	26.2	6653	p=0.00

	US MAP	2.2	59.2	100	56.4	30.2	86.2%	–
Science & Tech	Control	7.7	52.3	92.7	51.7	24.1	14877	–
	Treatment	3.9	30.4	84.2	34.6	22.2	6653	p=0.00
	US MAP	2.3	50.5	100	50.8	27.5	84.5%	–
Social Issues	Control	9.6	67.8	96.1	62.8	24.8	14877	–
	Treatment	5.1	45.5	92.1	46.9	25.4	6653	p=0.00
	US MAP	1.1	56.6	97.7	54.3	27.9	86.6%	–
Sports	Control	3.3	47.6	95.5	48.1	27.1	14877	–
	Treatment	0.6	27.8	87.1	33.2	24.3	6653	p=0.00
	US MAP	1.7	48.4	100	49.2	29	81.8%	–
Travel & Leisure Activities	Control	7.8	50.3	91.3	50.2	23.2	14877	–
	Treatment	3.8	30	81.4	33.9	21.2	6653	p=0.00
	US MAP	2.9	50	100	50.3	26.4	85.6%	–
TV & Movies	Control	7.9	52.6	92.3	52	23.5	14877	–
	Treatment	4	30.8	81	34.5	21.2	6653	p=0.00
	US MAP	1.5	48.8	100	48.5	26.6	86.5%	–
Vehicles & Transportation	Control	5.9	53.1	94.6	52.1	26.2	14877	–
	Treatment	2.9	31.6	90	36.5	24.7	6653	p=0.00
	US MAP	2.5	52.6	100	52.4	29	87.1%	–
Visual Arts, Architecture & Crafts	Control	8.4	52.5	92	51.8	23.5	14877	–
	Treatment	3.9	30.2	81.7	34.3	21.6	6653	p=0.00
	US MAP	2.3	49.2	100	49.1	26.8	87.7%	–

1847 observations (7.9%) dropped by listwise deletion. The last column reports the unadjusted p-value from a two-sided test of the hypothesis of no difference between treatment and control groups on each metric, computed using the baseline OLS model (see Section S1.5.1).

Table 20: Percentage of content exposure (by topic) that is from cross-cutting sources during study period

Metric	Group	p2.5	p50	p97.5	Avg.	SD	N	p(diff!=0)
Animals & Pets	Control	0.8	17.4	68.1	22.1	18.1	14877	–
	Treatment	1.7	25.2	75	29.2	20.3	6653	p=0.00
	US MAP	0.3	17.2	76.1	22.7	20.2	84.2%	–
Books & Literature	Control	0	14.3	71.4	20	19.6	14877	–
	Treatment	0	22.2	80.5	27.3	22.2	6653	p=0.00
	US MAP	0	18.6	100	26	24.3	66.1%	–
Business, Finance & Economics	Control	0	13.6	66.5	19.1	18	14877	–
	Treatment	0.4	20.2	73.5	25.4	20.3	6653	p=0.00
	US MAP	0	15.8	83.3	22.7	21.8	75.7%	–
Crime & Tragedy	Control	0.2	14.3	71.5	20.8	19.6	14877	–
	Treatment	0.7	22.6	77.9	28	21.9	6653	p=0.00
	US MAP	0	15.4	82.4	22.6	22.1	82.4%	–
Education & Learning	Control	0.2	15.9	68.2	21.1	18.3	14877	–
	Treatment	1.1	24.2	75.1	28.4	20.4	6653	p=0.00
	US MAP	0.3	17.5	83.3	23.8	21.7	79.5%	–
Fashion & Style	Control	0.5	15.4	69.6	20.9	18.6	14877	–
	Treatment	1.2	22.2	75	27	20.3	6653	p=0.00
	US MAP	0	15.6	79.1	22.3	21.2	85.5%	–
Children & Parenting	Control	1.4	19.3	65.2	23.3	17.3	14877	–
	Treatment	2.8	27.4	72.8	30.5	19.2	6653	p=0.00
	US MAP	0.7	18.2	73.8	23.5	19.7	88.7%	–
Fitness & Workouts	Control	0	15.4	71.6	20.9	19.6	14877	–
	Treatment	0	21.3	79	26.6	21.9	6653	p=0.00
	US MAP	0	17.1	91.8	24	22.6	71.2%	–
Food & Drink	Control	0.6	16.1	68.8	21.5	18.5	14877	–
	Treatment	1.2	23.8	76.1	28.2	20.7	6653	p=0.00
	US MAP	0.2	15.7	80	22.4	21.2	84.5%	–
Games, Puzzles & Play	Control	0	14	70	20	18.9	14877	–
	Treatment	0.4	20.8	77.4	26.1	21.2	6653	p=0.00
	US MAP	0	15.4	84.2	22.4	21.9	77.5%	–
Health & Medical	Control	0.6	14.5	64.6	19.4	17.2	14877	–
	Treatment	1.2	21.8	71	26.1	19.5	6653	p=0.00
	US MAP	0.3	16.5	73.9	22.2	19.9	85.9%	–
History & Philosophy	Control	0	12.5	84	20.6	23.2	14877	–
	Treatment	0	21.2	93.7	28.5	26.3	6653	p=0.00
	US MAP	0	20	100	28.9	27.3	53.4%	–
Holidays & Celebrations	Control	1.8	20.2	68	24.4	17.9	14877	–
	Treatment	3.2	28.9	74.8	32.1	19.5	6653	p=0.00
	US MAP	0.7	18.5	75	24	20.1	88.2%	–
Home & Garden	Control	0.6	15.7	75.8	22.3	20.4	14877	–
	Treatment	1	23.8	80	29.1	22.2	6653	p=0.00
	US MAP	0.2	14.8	80	22	21.5	84.6%	–
Music & Audio	Control	0.5	16.3	66.8	21.2	18	14877	–
	Treatment	1.5	24.2	73.6	28.1	20	6653	p=0.00
	US MAP	0	16.9	82.5	23.6	22	83.2%	–
Performing Arts	Control	0	15.4	75	21.5	21	14877	–
	Treatment	0	22.4	84.2	28	23.3	6653	p=0.00
	US MAP	0	19.4	100	26	23.7	67.9%	–
Politics	Control	0.2	11.3	75.5	19.1	20.5	14877	–
	Treatment	0.5	20	81.8	26.8	23.2	6653	p=0.00
	US MAP	0	16.4	88.9	24.5	24.2	82.4%	–
Relationships, Friends & Family	Control	1	19.5	66.3	23.5	17.7	14877	–
	Treatment	2.1	28.1	74.1	31.1	19.8	6653	p=0.00
	US MAP	0.3	18.4	75.4	23.8	20.4	88.7%	–
Religion & Spirituality	Control	0	16.8	77.5	23.7	21.7	14877	–
	Treatment	0.6	27.8	84.2	32.5	24.1	6653	p=0.00

	US MAP	0	15.5	85.9	23.6	23.5	81.3%	–
Science & Tech	Control	0.3	14.4	65.5	19.6	17.5	14877	–
	Treatment	1	22	72.6	26.2	19.8	6653	p=0.00
	US MAP	0	15.4	80	22	21.1	80%	–
Social Issues	Control	0.3	12	69.7	18.6	19	14877	–
	Treatment	0.6	20.1	77.3	26	21.6	6653	p=0.00
	US MAP	0	16.1	79.5	22.9	21.7	84.1%	–
Sports	Control	0	15.8	77.1	22.7	21.5	14877	–
	Treatment	0	23.1	83.3	28.7	23.7	6653	p=0.00
	US MAP	0	15	86.3	22.7	22.8	75.8%	–
Travel & Leisure Activities	Control	0.5	17.8	67.5	22.4	18	14877	–
	Treatment	1.6	25.5	74.3	29.3	20	6653	p=0.00
	US MAP	0	17.9	78.1	23.5	20.7	81.9%	–
TV & Movies	Control	0.5	14.5	62.7	19.1	16.7	14877	–
	Treatment	1.3	21.3	70.5	25.7	19.1	6653	p=0.00
	US MAP	0	16	79.5	22.3	21	83.9%	–
Vehicles & Transportation	Control	0.5	16.1	76	23	21	14877	–
	Treatment	1	24.4	80.9	30	23	6653	p=0.00
	US MAP	0	14.9	80.9	22.2	21.9	83.5%	–
Visual Arts, Architecture & Crafts	Control	0.7	16	66.5	20.9	17.7	14877	–
	Treatment	1.4	24.4	74.2	28.4	20.1	6653	p=0.00
	US MAP	0	16.3	77.7	22.3	20.5	85.2%	–

1847 observations (7.9%) dropped by listwise deletion. The last column reports the unadjusted p-value from a two-sided test of the hypothesis of no difference between treatment and control groups on each metric, computed using the baseline OLS model (see Section S1.5.1).

2.3 Sources and characteristics

The tables below offer detailed information on the sources (friends, Pages, and groups) of like-minded and cross-cutting exposure among the treatment group, the control group, and US monthly active users and the specific characteristics of these exposures. Each table employs listwise deletion so quantities can be easily compared within the table. As a result, the exact sample sizes vary by table depending on the prevalence of missing data.

Table 21 presents the percentage of content exposure that came from like-minded versus cross-cutting sources — friends, Pages, and groups — during the pre-treatment period among the treatment and control groups.

Table 21: Percentage of content exposure (by source) that is from like-minded or cross-cutting sources during pre-treatment period

Metric	Group	p2.5	p50	p97.5	Avg.	SD	N	p(diff!=0)
% like-minded exposure: friends	Control	12.6	62.8	93.7	60.2	21.9	15835	–
	Treatment	12.6	62.2	93.1	59.8	21.8	7077	p=0.44
	US MAP	0	55.3	95.7	51.7	28.2	96.4%	–
% like-minded exposure: Pages	Control	2.2	41.9	92.9	43.7	26.2	15835	–
	Treatment	2.7	41.2	92.1	43.4	26	7077	p=0.73
	US MAP	0	31.4	92.7	36	28.2	93.6%	–
% like-minded exposure: groups	Control	0	45.9	100	47.1	34	15835	–
	Treatment	0	44.3	100	46.7	34.3	7077	p=0.46
	US MAP	0	46.7	100	47.6	39	85.6%	–
% cross-cutting exposure: friends	Control	1.3	21.5	69.1	25.3	18.7	15835	–
	Treatment	1.2	21.6	68.1	25.5	18.5	7077	p=0.59
	US MAP	0	18.2	78	24.1	21.4	96.4%	–
% cross-cutting exposure: Pages	Control	0	7.7	57.9	13.5	15.6	15835	–
	Treatment	0	7.9	56.1	13.6	15.5	7077	p=0.50
	US MAP	0	5.8	69.5	12.9	18	93.6%	–
% cross-cutting exposure: groups	Control	0	4	88.8	16.2	24.3	15835	–
	Treatment	0	4	87.3	16.1	24	7077	p=0.62
	US MAP	0	0.3	99.6	14.3	26.6	85.6%	–

465 observations (1.99%) dropped by listwise deletion. The last column reports the unadjusted p-value from a two-sided test of the hypothesis of no difference between treatment and control groups on each metric, computed using the baseline OLS model (see Section S1.5.1).

Table 22 presents the percentage of content exposure that came from like-minded versus cross-cutting sources — friends, Pages, and groups — during the study period among the treatment and control groups.

Table 22: Percentage of content exposure (by source) that is from like-minded or cross-cutting sources during study period

Metric	Group	p2.5	p50	p97.5	Avg.	SD	N	p(diff!=0)
% like-minded exposure: friends	Control	13.4	63.1	93.8	60.5	21.8	15786	–
	Treatment	7.4	45.1	88.8	46.4	22.8	7107	p=0.00
	US MAP	0	54.5	95.6	51.9	27.5	95.7%	–
% like-minded exposure: Pages	Control	2.3	44	93.3	45.2	26.1	15786	–
	Treatment	1.2	20.2	79.7	26.4	21.4	7107	p=0.00
	US MAP	0	34.7	94.7	38.5	28.1	92.1%	–
% like-minded exposure: groups	Control	0	43.7	100	45.9	33.5	15786	–
	Treatment	0	18.4	99.6	30.8	31.2	7107	p=0.00
	US MAP	0	42.6	100	46.2	38.2	85.5%	–
% cross-cutting exposure: friends	Control	1.4	21.4	69.5	25.3	18.6	15786	–
	Treatment	2.4	32.8	77.1	34.9	21	7107	p=0.00
	US MAP	0	18.8	74.7	23.9	20.4	95.7%	–
% cross-cutting exposure: Pages	Control	0	8.2	58.6	13.9	15.8	15786	–
	Treatment	0.2	12.2	63.1	17.8	17.1	7107	p=0.00
	US MAP	0	6.5	65.7	13.5	17.7	92.1%	–
% cross-cutting exposure: groups	Control	0	3.9	88.5	16	23.9	15786	–
	Treatment	0	7.8	89.9	19.9	25.4	7107	p=0.00
	US MAP	0	0.3	96.4	13.5	24.8	85.5%	–

484 observations (2.07%) dropped by listwise deletion. The last column reports the unadjusted p-value from a two-sided test of the hypothesis of no difference between treatment and control groups on each metric, computed using the baseline OLS model (see Section S1.5.1).

Table 23 shows the percentage of content exposure from like-minded versus cross-cutting sources that originated from reshared versus non-reshared posts during the pre-treatment period among the treatment and control groups.

Table 23: Percentage of content exposure (by content origin) that is from like-minded or cross-cutting sources during pre-treatment period

Metric	Group	p2.5	p50	p97.5	Avg.	SD	N	p(diff!=0)
% like-minded exposure: reshared posts	Control	10.8	62.4	94.8	59.7	23.3	16065	–
	Treatment	10.2	61.8	94.6	59.1	23.3	7176	p=0.26
	US MAP	0	55.9	97.9	52.3	29.4	95.4%	–
% like-minded exposure: non-reshare posts	Control	9	52	90.7	51.1	23	16065	–
	Treatment	8.8	51.7	89.9	50.7	22.9	7176	p=0.40
	US MAP	0	48.1	92	46.6	26.8	97.6%	–
% cross-cutting exposure: reshared posts	Control	0.6	18	69.6	23	19	16065	–
	Treatment	0.6	18.4	69.8	23.3	19	7176	p=0.36
	US MAP	0	15.4	80.7	22.5	22.1	95.4%	–
% cross-cutting exposure: non-reshare posts	Control	1.2	14.8	62.3	19.7	16.4	16065	–
	Treatment	1.3	15.3	60.6	19.7	16.1	7176	p=0.98
	US MAP	0	13.8	71.3	20	19.4	97.6%	–

136 observations (0.58%) dropped by listwise deletion. The last column reports the unadjusted p-value from a two-sided test of the hypothesis of no difference between treatment and control groups on each metric, computed using the baseline OLS model (see Section S1.5.1).

Table 24 shows the percentage of content exposure from like-minded versus cross-cutting sources that originated from reshared versus non-reshared posts during the study period among the treatment and control groups.

Table 25 shows the percentage of content exposure from like-minded versus cross-cutting users, Pages, and group that originated from reshared versus non-reshared posts during the pre-treatment period among the treatment and control groups.

Table 24: Percentage of content exposure (by content origin) that is from like-minded or cross-cutting sources during study period

Metric	Group	p2.5	p50	p97.5	Avg.	SD	N	p(diff!=0)
% like-minded exposure: reshared posts	Control	10.8	62.2	95	59.4	23.3	16024	–
	Treatment	4.7	40.7	88.7	42.8	23.5	7167	p=0.00
	US MAP	0	54.4	97.8	52.1	28.8	94.5%	–
% like-minded exposure: non-reshare posts	Control	9.6	52.3	90.6	51.5	22.7	16024	–
	Treatment	5.1	30.3	79.9	34	20.5	7167	p=0.00
	US MAP	0	47.5	92.7	47.1	26.4	96.7%	–
% cross-cutting exposure: reshared posts	Control	0.6	18.1	70.3	23.1	19.1	16024	–
	Treatment	1.5	29.5	78.5	32.6	21.7	7167	p=0.00
	US MAP	0	16	76.7	22.2	21.1	94.5%	–
% cross-cutting exposure: non-reshare posts	Control	1.2	15.2	62.5	19.8	16.4	16024	–
	Treatment	2	22.4	68.7	26	18.2	7167	p=0.00
	US MAP	0	14.4	67.4	19.8	18.3	96.7%	–

186 observations (0.8%) dropped by listwise deletion. The last column reports the unadjusted p-value from a two-sided test of the hypothesis of no difference between treatment and control groups on each metric, computed using the baseline OLS model (see Section S1.5.1).

Table 25: Percentage of content exposure (by content origin and source) that is from like-minded or cross-cutting sources during pre-treatment period

Metric	Group	p2.5	p50	p97.5	Avg.	SD	N	p(diff!=0)
% like-minded: reshared user posts	Control	11.6	65.2	95.7	61.8	23.1	15119	–
	Treatment	11.1	64.2	95.1	61.3	23.1	6746	p=0.45
	US MAP	0	57.2	98.5	53	29.8	94.5%	–
% like-minded: non-reshare user posts	Control	14.4	61.1	92.7	59	21.1	15119	–
	Treatment	13.4	60.7	92	58.6	21.1	6746	p=0.59
	US MAP	0	53.9	94.8	50.8	27.3	96.1%	–
% cross-cutting: reshared user posts	Control	0.7	19.9	71.4	24.7	19.7	15119	–
	Treatment	0.7	20.3	71.2	24.9	19.6	6746	p=0.61
	US MAP	0	16.6	83	23.7	22.8	94.5%	–
% cross-cutting: non-reshare user posts	Control	1.7	22.6	67.6	25.9	18	15119	–
	Treatment	1.7	22.6	66.9	26.1	17.9	6746	p=0.64
	US MAP	0	19.4	77.1	24.7	21	96.1%	–
% like-minded: reshared Page posts	Control	0	51.6	100	50.6	32.3	15119	–
	Treatment	0	51.3	100	50.2	32.2	6746	p=0.74
	US MAP	0	48.2	100	47.5	36.5	78.6%	–
% like-minded: non-reshare Page posts	Control	3.4	42	92.6	43.9	25.7	15119	–
	Treatment	3.8	41.6	92	43.7	25.5	6746	p=0.86
	US MAP	0	30.7	92.5	35.5	28	93.5%	–
% cross-cutting: reshared Page posts	Control	0	4.7	79.2	14.4	21.2	15119	–
	Treatment	0	5	78	14.7	21.2	6746	p=0.36
	US MAP	0	2	99	15	25.7	78.6%	–
% cross-cutting: non-reshare Page posts	Control	0.1	7.9	57.6	13.6	15.3	15119	–
	Treatment	0	8	55.8	13.6	15.1	6746	p=0.86
	US MAP	0	5.8	68.8	12.8	17.8	93.5%	–
% like-minded: reshared group posts	Control	0	52.7	100	51.1	35.2	15119	–
	Treatment	0	52.4	100	50.7	35.5	6746	p=0.76
	US MAP	0	50	100	49.3	39.8	80.2%	–
% like-minded: non-reshare group posts	Control	0	44.3	100	46.2	33.9	15119	–
	Treatment	0	42.9	100	45.7	34.1	6746	p=0.55
	US MAP	0	45.8	100	47.4	39.2	84.6%	–
% cross-cutting: reshared group posts	Control	0	3.1	91.9	15.6	24.6	15119	–
	Treatment	0	2.9	90.4	15.5	24.4	6746	p=0.69
	US MAP	0	0.1	100	14.1	27	80.2%	–
% cross-cutting: non-reshare group posts	Control	0	4.1	88.6	16.5	24.4	15119	–
	Treatment	0	4.1	86.8	16.2	23.9	6746	p=0.40
	US MAP	0	0.2	100	14.3	26.7	84.6%	–

1512 observations (6.47%) dropped by listwise deletion. The last column reports the unadjusted p-value from a two-sided test of the hypothesis of no difference between treatment and control groups on each metric, computed using the baseline OLS model (see Section S1.5.1).

Table 26 shows the percentage of content exposure from like-minded versus cross-cutting users, Pages, and group that originated from reshared versus non-reshared posts during the study period among the treatment and control groups.

Table 26: Percentage of content exposure (by content origin and source) that is from like-minded or cross-cutting sources during study period

Metric	Group	p2.5	p50	p97.5	Avg.	SD	N	p(diff!=0)
% like-minded: reshared user posts	Control	11.8	65.1	95.8	61.8	23.2	14871	–
	Treatment	5.7	46	91.5	47.1	24.4	6823	p=0.00
	US MAP	0	56	98.4	53	29.2	93.5%	–
% like-minded: non-reshare user posts	Control	14.6	61.8	92.8	59.7	21.1	14871	–
	Treatment	8.8	46	87.4	46.9	21.8	6823	p=0.00
	US MAP	0	53.6	94.8	51.3	26.9	95.4%	–
% cross-cutting: reshared user posts	Control	0.7	19.9	72.3	24.7	19.7	14871	–
	Treatment	1.5	32.2	80.7	34.8	22.6	6823	p=0.00
	US MAP	0	17.2	79.6	23.5	21.9	93.5%	–
% cross-cutting: non-reshare user posts	Control	1.6	22.3	67.6	25.8	18.1	14871	–
	Treatment	2.8	32.7	74.7	34.5	20.1	6823	p=0.00
	US MAP	0	19.8	74	24.4	20.1	95.4%	–
% like-minded: reshared Page posts	Control	0	51.6	100	50.8	32.1	14871	–
	Treatment	0	17.9	94.7	27.3	27.3	6823	p=0.00
	US MAP	0	46.8	100	47.4	35.8	76.6%	–
% like-minded: non-reshare Page posts	Control	3.6	44.2	92.8	45.5	25.6	14871	–
	Treatment	1.6	20.3	78.9	26.4	21.1	6823	p=0.00
	US MAP	0	34.2	94.5	38.2	28	92%	–
% cross-cutting: reshared Page posts	Control	0	4.7	77.3	14.4	21.2	14871	–
	Treatment	0	10.6	85.4	19.9	23.7	6823	p=0.00
	US MAP	0	2.1	96.1	14.6	24.4	76.6%	–
% cross-cutting: non-reshare Page posts	Control	0	8.3	56.9	13.8	15.3	14871	–
	Treatment	0.5	12.4	62.1	17.8	16.8	6823	p=0.00
	US MAP	0	6.5	65.6	13.4	17.6	92%	–
% like-minded: reshared group posts	Control	0	50.6	100	50	34.8	14871	–
	Treatment	0	24.8	100	35.5	33.1	6823	p=0.00
	US MAP	0	46.3	100	47.9	39	79.9%	–
% like-minded: non-reshare group posts	Control	0	42.8	100	45.4	33.4	14871	–
	Treatment	0	18	99.6	30.4	31.3	6823	p=0.00
	US MAP	0	42	100	46.1	38.4	84.6%	–
% cross-cutting: reshared group posts	Control	0	3	90.4	15.4	24.2	14871	–
	Treatment	0	6.9	92.4	19.8	26.5	6823	p=0.00
	US MAP	0	0	99.5	13.3	25.2	79.9%	–
% cross-cutting: non-reshare group posts	Control	0	4	87.1	16	23.8	14871	–
	Treatment	0	7.8	89.7	19.9	25.6	6823	p=0.00
	US MAP	0	0.2	96.9	13.5	25	84.6%	–

1683 observations (7.2%) dropped by listwise deletion. The last column reports the unadjusted p-value from a two-sided test of the hypothesis of no difference between treatment and control groups on each metric, computed using the baseline OLS model (see Section S1.5.1).

Table 27 shows the percentage of exposure to content that was classified as uncivil from like-minded versus cross-cutting sources during the pre-treatment period among the treatment and control groups.

Table 27: Percentage of content exposure to like-minded and cross-cutting sources that is uncivil during pre-treatment period

Metric	Group	p2.5	p50	p97.5	Avg.	SD	N	p(diff!=0)
% uncivil: like-minded (all)	Control	0.7	3.7	10.8	4.2	2.6	16032	–
	Treatment	0.7	3.6	10.7	4.2	2.6	7161	p=0.84
	US MAP	0	2.7	13.8	3.6	4.1	93.9%	–
% uncivil: cross-cutting (all)	Control	0	3.1	10.8	3.7	2.9	16032	–
	Treatment	0	3.1	10.7	3.7	3	7161	p=0.31
	US MAP	0	2.7	14.4	3.6	4.5	92.8%	–
% uncivil: other (all)	Control	0	1.7	9	2.4	2.6	16032	–
	Treatment	0	1.7	8.9	2.4	2.5	7161	p=0.31
	US MAP	0	1.2	12.7	2.4	3.9	96.1%	–

184 observations (0.79%) dropped by listwise deletion. The last column reports the unadjusted p-value from a two-sided test of the hypothesis of no difference between treatment and control groups on each metric, computed using the baseline OLS model (see Section S1.5.1).

Table 28 shows the percentage of exposure to content that was classified as uncivil from like-minded versus cross-cutting sources during the study period among the treatment and control groups.

Table 28: Percentage of content exposure to like-minded and cross-cutting sources that is uncivil during study period

Metric	Group	p2.5	p50	p97.5	Avg.	SD	N	p(diff!=0)
% uncivil: like-minded sources	Control	0.5	3.1	9.6	3.6	2.5	15985	–
	Treatment	0.3	3.1	9.8	3.6	2.6	7156	p=0.08
	US MAP	0	2.2	12.4	3	4	93.7%	–
% uncivil: cross-cutting sources	Control	0	2.5	9.7	3.1	2.7	15985	–
	Treatment	0	2.4	8.7	2.9	2.8	7156	p=0.00
	US MAP	0	2.2	13.2	3.1	4.4	92.5%	–
% uncivil: other sources	Control	0	1.6	8.2	2.2	2.2	15985	–
	Treatment	0	1.4	6.7	1.9	1.8	7156	p=0.00
	US MAP	0	1.1	11.7	2.1	3.8	95.2%	–

236 observations (1.01%) dropped by listwise deletion. The last column reports the unadjusted p-value from a two-sided test of the hypothesis of no difference between treatment and control groups on each metric, computed using the baseline OLS model (see Section S1.5.1).

2.4 Behavior

The tables below report users' engagement with different kinds of like-minded and cross-cutting content before and during the treatment period among both the treatment and control groups, as well as Facebook U.S. monthly active users. Each table employs listwise deletion so quantities can be easily compared within the table. As a result, the exact sample sizes vary by table depending on the prevalence of missing data.

Table 29 shows the percentage of total comments that were uncivil, made by users in the treatment and control group, on content that was like-minded, cross-cutting, and neither like-minded nor cross-cutting. The data are from the pre-treatment period.

Table 29: Percentage of comments on posts by like-minded and cross-cutting sources that are uncivil during pre-treatment period

Metric	Group	p2.5	p50	p97.5	Avg.	SD	N	p(diff!=0)
% uncivil comments: like-minded posts	Control	0	1.9	19	4	6	12005	–
	Treatment	0	1.8	18.8	3.9	6.3	5327	p=0.58
	US MAP	0	0	21.4	2.8	7.7	65.5%	–
% uncivil comments: cross-cutting posts	Control	0	0	22.2	3.6	7.3	12005	–
	Treatment	0	0	22.5	3.5	7.6	5327	p=0.58
	US MAP	0	0	25	2.8	8.9	57%	–
% uncivil comments: other posts	Control	0	0	25	3.4	8	12005	–
	Treatment	0	0	22.8	3.4	7.9	5327	p=0.70
	US MAP	0	0	25	2.6	8.8	59.5%	–

6045 observations (25.86%) dropped by listwise deletion. The last column reports the unadjusted p-value from a two-sided test of the hypothesis of no difference between treatment and control groups on each metric, computed using the baseline OLS model (see Section S1.5.1).

Table 30 shows the percentage of total comments that were uncivil, made by users in the treatment and control group, on content that was like-minded, cross-cutting, and neither like-minded nor cross-cutting. The data are from during the treatment period.

Table 31 shows pre-treatment rates of engagement (conditional on exposure) for the control and treatment groups, with like-minded, cross-cutting, and other content. Engagement includes clicking anywhere in the post (e.g., a link click, "see more," profile click), reacting (e.g., Like, Love, Sad), commenting, and resharing.

Table 30: Percentage of comments on posts by like-minded and cross-cutting sources that are uncivil during study period

Metric	Group	p2.5	p50	p97.5	Avg.	SD	N	p(diff!=0)
% uncivil comments: like-minded posts	Control	0	1.7	18.8	3.8	6	11699	–
	Treatment	0	1	18.8	3.6	6.3	5205	p=0.07
	US MAP	0	0	21.4	2.7	7.8	65%	–
% uncivil comments: cross-cutting posts	Control	0	0	21.4	3.4	7.7	11699	–
	Treatment	0	0	22.2	3.5	7.4	5205	p=0.44
	US MAP	0	0	25	2.6	8.8	56.3%	–
% uncivil comments: other posts	Control	0	0	25	3.4	8.5	11699	–
	Treatment	0	0	25	3.7	8.8	5205	p=0.11
	US MAP	0	0	25	2.5	8.9	58.4%	–

6473 observations (27.69%) dropped by listwise deletion. The last column reports the unadjusted p-value from a two-sided test of the hypothesis of no difference between treatment and control groups on each metric, computed using the baseline OLS model (see Section S1.5.1).

Table 31: Engagement rates (conditional on exposure) with posts by like-minded and cross-cutting sources during pre-treatment period

Metric	Group	p2.5	p50	p97.5	Avg.	SD	N	p(diff!=0)
Click rate: like-minded content	Control	2.2	7.9	22.6	9.1	5.6	14712	–
	Treatment	2.1	7.9	22.1	9.1	5.4	6552	p=0.90
	US MAP	0	6.3	22.9	7.7	13.3	93.9%	–
Click rate: cross-cutting content	Control	2	8.2	24.5	9.5	6.1	14712	–
	Treatment	2	8.2	24.1	9.5	6.1	6552	p=0.67
	US MAP	0	6.4	24.6	7.9	9.5	92.8%	–
Click rate: other content	Control	1.8	7.6	22.3	8.8	5.6	14712	–
	Treatment	1.8	7.8	22.2	8.8	5.5	6552	p=0.59
	US MAP	0	5.9	21.9	7.3	11.9	96.1%	–
Reaction rate: like-minded content	Control	0.1	3.7	38.6	7.8	10.4	14712	–
	Treatment	0.1	3.9	39.4	8	10.8	6552	p=0.04
	US MAP	0	1.7	34	5.6	90.6	93.9%	–
Reaction rate: cross-cutting content	Control	0	3.5	37.8	7.5	11.2	14712	–
	Treatment	0	3.7	38.2	7.7	11.4	6552	p=0.17
	US MAP	0	1.6	34.8	5.5	13.8	92.8%	–
Reaction rate: other content	Control	0	2.6	37.2	6.6	10	14712	–
	Treatment	0	2.7	37.5	6.9	10.4	6552	p=0.05
	US MAP	0	1.1	32.8	4.9	24.3	96.1%	–
Comment rate: like-minded content	Control	0	0.5	9.1	1.5	4.1	14712	–
	Treatment	0	0.5	8.8	1.4	2.9	6552	p=0.13
	US MAP	0	0.1	8.3	1.2	18.5	93.9%	–
Comment rate: cross-cutting content	Control	0	0.5	11.9	2.4	57.7	14712	–
	Treatment	0	0.5	11.3	1.9	9	6552	p=0.33
	US MAP	0	0.1	9.5	1.3	16.8	92.8%	–
Comment rate: other content	Control	0	0.8	24.3	4	22	14712	–
	Treatment	0	0.8	21.7	3.6	21.7	6552	p=0.27
	US MAP	0	0.1	7.5	1	15.7	96.1%	–
Reshare rate: like-minded content	Control	0	0.1	3.7	0.6	7.1	14712	–
	Treatment	0	0.1	4.3	0.5	1.9	6552	p=0.69
	US MAP	0	0	2	0.3	21.7	96.3%	–
Reshare rate: cross-cutting content	Control	0	0	4.3	0.6	6.1	14712	–
	Treatment	0	0	4.1	0.6	3.6	6552	p=0.52
	US MAP	0	0	2.2	0.3	3.2	95.5%	–
Reshare rate: other content	Control	0	0.2	13.5	1.9	14.1	14712	–
	Treatment	0	0.2	14.6	1.9	7.5	6552	p=0.64
	US MAP	0	0	5.9	0.8	26.1	99%	–

2113 observations (9.04%) dropped by listwise deletion. The last column reports the unadjusted p-value from a two-sided test of the hypothesis of no difference between treatment and control groups on each metric, computed using the baseline OLS model (see Section S1.5.1).

Table 32 shows rates of engagement (conditional on exposure) for the control and treatment groups, with like-minded, cross-cutting, and other content. Engagement includes clicking anywhere in the post (e.g., a link click, "see more," profile click), reacting (e.g., Like, Love, Sad), commenting, and resharing. These data are from during the treatment period.

Table 32: Engagement rates (conditional on exposure) with posts by like-minded and cross-cutting sources during study period

Metric	Group	p2.5	p50	p97.5	Avg.	SD	N	p(diff!=0)
Click rate: like-minded content	Control	2	7	20.3	8	5.2	14548	–
	Treatment	2	7.5	20	8.4	5.2	6472	p=0.00
	US MAP	0	5.7	19.8	6.9	88.2	93.7%	–
Click rate: cross-cutting content	Control	1.8	7.2	21.5	8.3	5.6	14548	–
	Treatment	1.7	6.5	18.4	7.4	4.8	6472	p=0.00
	US MAP	0	5.7	20.7	6.9	26	92.5%	–
Click rate: other content	Control	1.7	6.8	20.1	7.8	5.1	14548	–
	Treatment	1.6	6.3	17.8	7.1	4.6	6472	p=0.00
	US MAP	0	5.3	19.2	6.5	20.2	95.2%	–
Reaction rate: like-minded content	Control	0.1	3.7	35.8	7.5	10.3	14548	–
	Treatment	0.1	4.4	36.8	8.1	10.3	6472	p=0.00
	US MAP	0	1.6	32	5.7	339.4	93.7%	–
Reaction rate: cross-cutting content	Control	0	3.4	34.2	7.1	9.7	14548	–
	Treatment	0	3	30.9	6.2	8.5	6472	p=0.00
	US MAP	0	1.5	32	5.1	18.2	92.5%	–
Reaction rate: other content	Control	0	2.5	33	6.1	11.2	14548	–
	Treatment	0	2.1	30	5.4	8.4	6472	p=0.00
	US MAP	0	1.1	30.2	4.6	16.6	95.2%	–
Comment rate: like-minded content	Control	0	0.4	7.7	1.3	2.7	14548	–
	Treatment	0	0.5	8.9	1.5	3.8	6472	p=0.00
	US MAP	0	0.1	7.6	1.1	20.2	93.7%	–
Comment rate: cross-cutting content	Control	0	0.5	10	2.3	63.5	14548	–
	Treatment	0	0.4	6.9	1.1	2.7	6472	p=0.04
	US MAP	0	0.1	8.5	1.2	14.9	92.5%	–
Comment rate: other content	Control	0	0.7	21	3.7	46.2	14548	–
	Treatment	0	0.5	11.5	1.9	10.4	6472	p=0.00
	US MAP	0	0.1	6.9	0.9	6.9	95.2%	–
Reshare rate: like-minded content	Control	0	0.1	3.8	0.5	1.9	14548	–
	Treatment	0	0.1	6.2	0.8	4.7	6472	p=0.00
	US MAP	0	0	2.2	0.3	50.5	95.9%	–
Reshare rate: cross-cutting content	Control	0	0	4.3	0.6	4.4	14548	–
	Treatment	0	0	2.8	0.4	1.6	6472	p=0.00
	US MAP	0	0	2.6	0.4	7.3	95%	–
Reshare rate: other content	Control	0	0.1	5.3	0.8	5.2	14548	–
	Treatment	0	0.1	4.7	0.6	2.5	6472	p=0.00
	US MAP	0	0	2.9	0.4	34.8	98%	–

2357 observations (10.08%) dropped by listwise deletion. The last column reports the unadjusted p-value from a two-sided test of the hypothesis of no difference between treatment and control groups on each metric, computed using the baseline OLS model (see Section S1.5.1).

3 Supplementary Tables and Figures: Analyses

3.1 Treatment effects: Exposure

This section explores the effect of the treatment on exposure to different types of content.

Extended Data Figure 3, which complements Figure 2 in the main text of the manuscript, displays the average day-level share of respondent views of content from cross-cutting sources by treatment group for the period of July 1–December 23, 2020. W1–W5 indicate Waves 1–5 of the survey; shading indicates wave duration. The treatment increased exposure to content from cross-cutting sources relative to the pre-treatment period. The magnitude of this increase was not equivalent to the decrease in exposure to like-minded sources because exposure to content from sources that were neither like-minded nor cross-cutting also increased. However, exposure to cross-cutting sources remained lower than exposure to like-minded sources throughout the entire study period.

Figure 4 shows the distribution of exposure, during the treatment period, to content from like-minded and cross-cutting sources for both the treatment and control groups.

Figure 4: Distribution of exposure by source type and treatment status

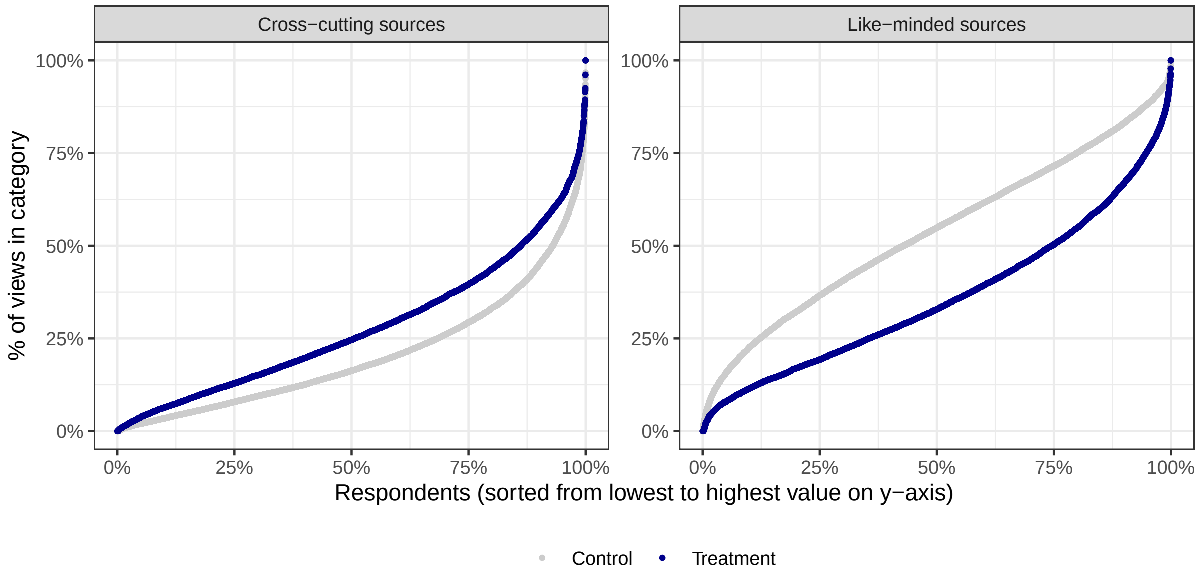

Figure 5 shows how the distribution of participants' Feeds changed between the pre-treatment and treatment periods for both like-minded and cross-cutting sources.

Figure 5: Distribution of change in exposure levels by source type and treatment status

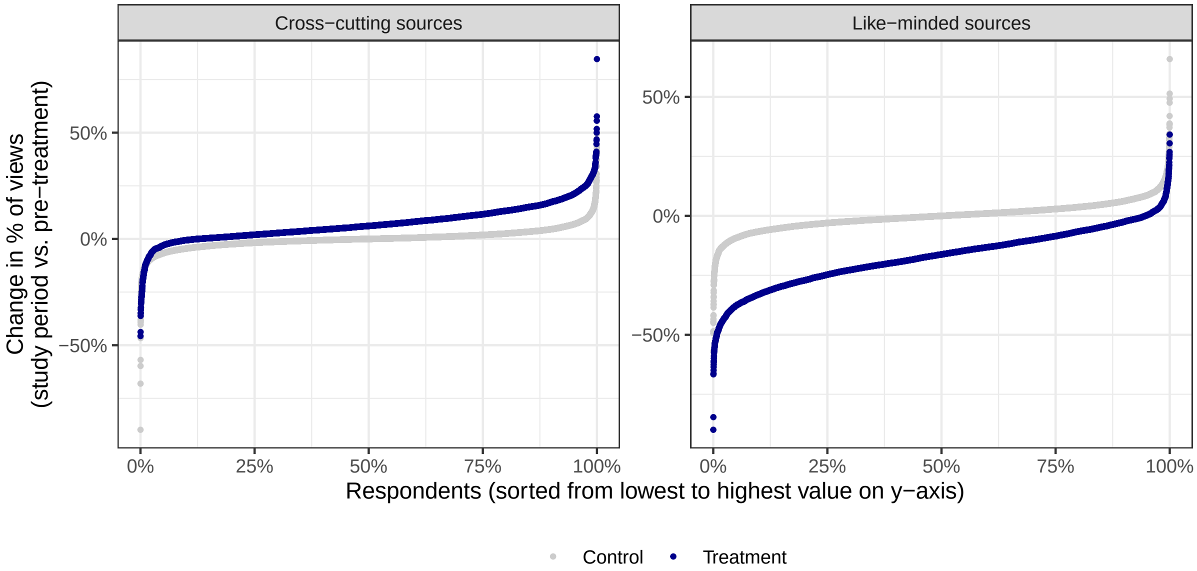

Figure 6 shows the number of participants in the treatment and the control group and pre-treatment exposure to content from like-minded sources. The x-axis shows the percentage of pre-treatment exposure to like-minded content. The y-axis shows the number of total participants (in both treatment and control) who fall into each of those buckets (note that the control group is much larger than the treatment group).

Figure 6: Number of respondents by treatment status and pre-treatment exposure to content from like-minded sources

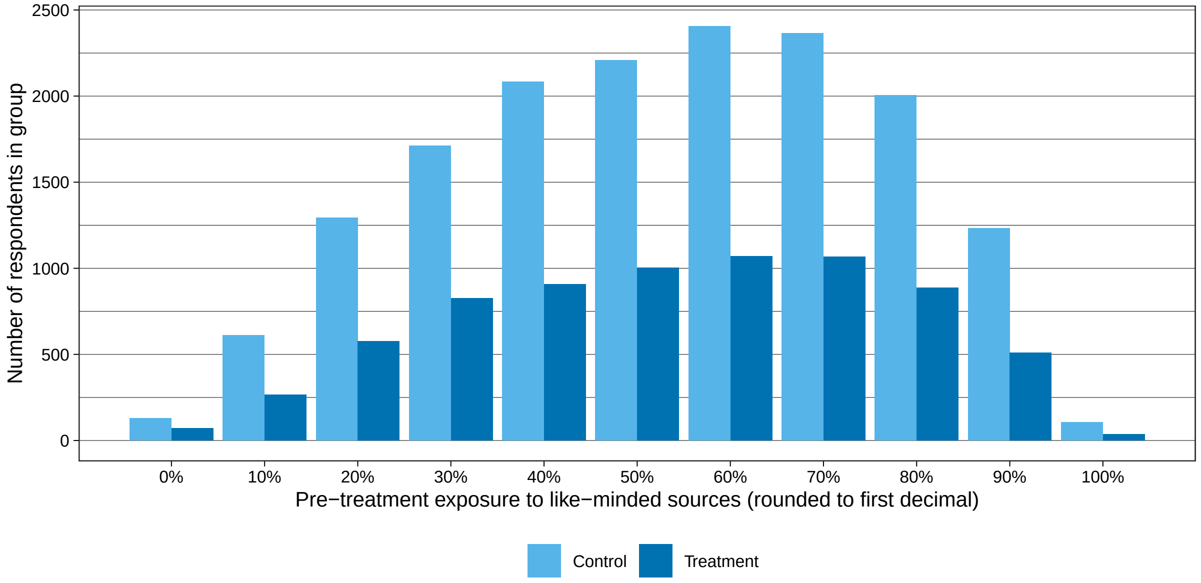

Figures 7 and 8 show how exposure to content from cross-cutting and like-minded sources changed among the treatment and the control group dependent on participants' pre-treatment exposure to content from like-minded sources.

Figure 7: Distribution of change in exposure by pre-treatment exposure to content from like-minded sources

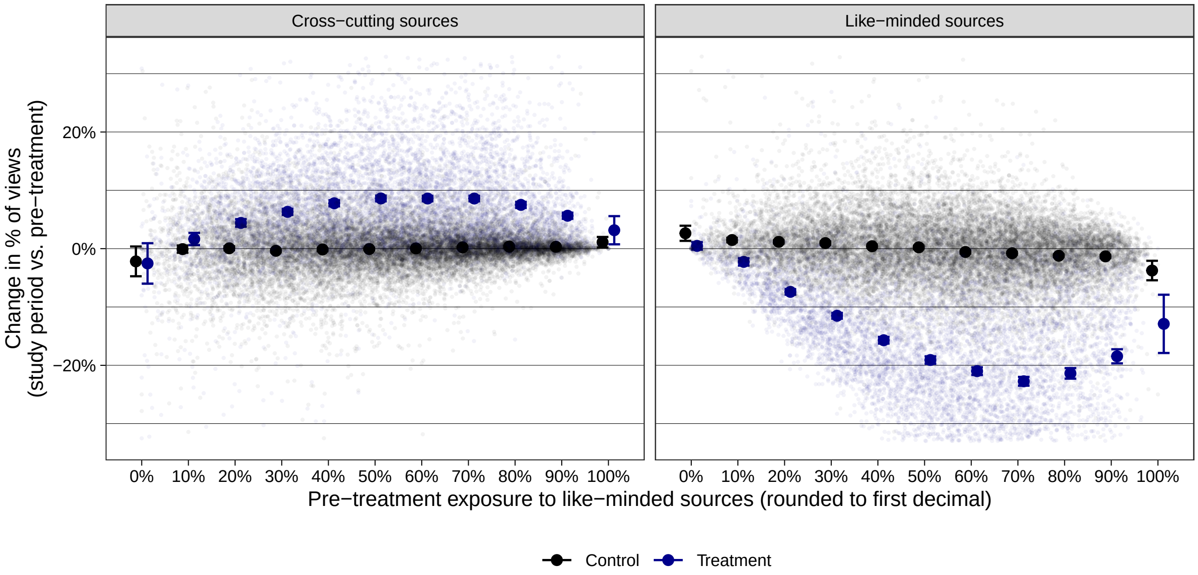

Exposure measured using Feed behavior data from participants during the study period. Error bars indicate 95% confidence intervals. Each point represents a participant in the study. 814 data points (1.7% of the sample) had values above 33% or below -33% and are excluded from the figure in order to facilitate interpretation.

Figure 8: Distribution of change in exposure by pre-treatment exposure to content from cross-cutting sources

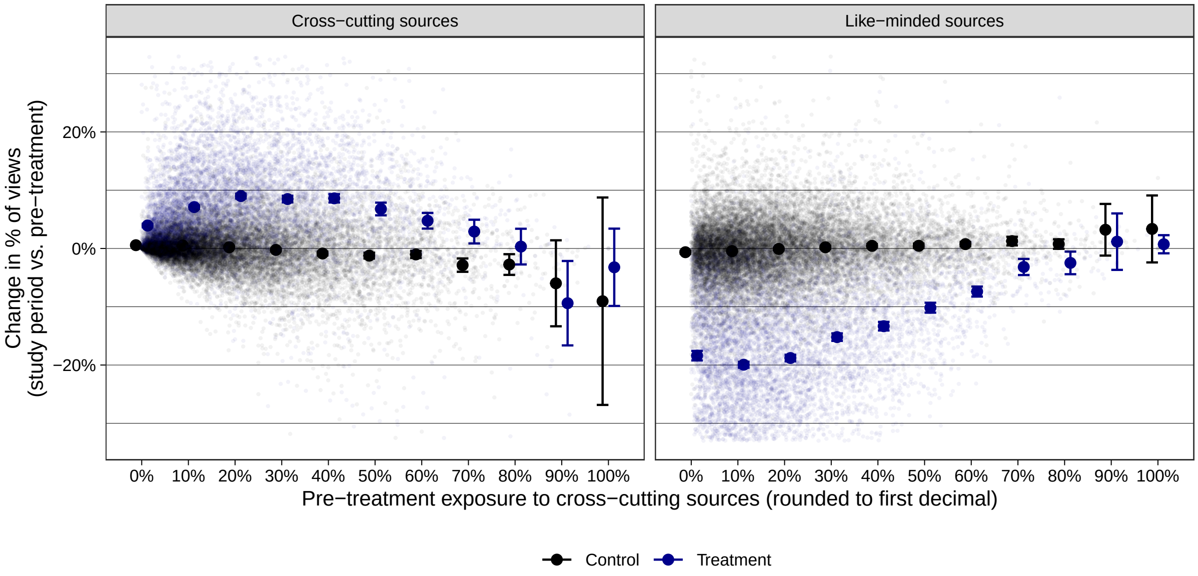

Exposure measured using Feed behavior data from participants during the study period. Error bars indicate 95% confidence intervals. Each point represents a participant in the study. 814 data points (1.7% of the sample) had values above 33% or below -33% and are excluded from the figure in order to facilitate interpretation.

Table 33 shows exposure (in both treatment and control group) to content from sources that are like-minded, cross-cutting, and neither like-minded nor cross-cutting. It also shows the change in exposure to content for which there was insufficient information available about the source to classify it: for example, a source with a non-U.S. audience (since the ideology classifier is only applied to U.S. users). In other analyses, this "no ideology" content is combined with "neither like-minded or cross-cutting" unless otherwise specified.

Table 33: Exposure to like-minded and cross-cutting content on Facebook

Metric	Control	Treatment	Diff	p(diff!=0)
% like-minded content	53.7	36.2	-17.5	p=0.00
% cross-cutting content	20.7	27.9	7.3	p=0.00
% other content	22.6	31.5	8.9	p=0.00
% no ideology	3.1	4.4	1.3	p=0.00

N=23228. 149 observations (0.64%) dropped by listwise deletion. The last column reports the unadjusted p-value from a two-sided test of the hypothesis of no difference between treatment and control groups on each metric, computed using the baseline OLS model (see Section S1.5.1).

Table 34 shows the average ideology of links to news domains participants saw on Facebook (weighted by views) and the average audience ideology of news domains participants visited (weighted by number of pageviews) during the treatment period. Facebook exposure was measured using Feed behavior data from participants during the study period. Web visits data were measured using web tracking data from the subset of study participants who opted in and completed Waves 3, 4, and/or 5 of the survey.

Table 34: Average audience ideology of domains visited during the treatment period

Metric	Control	Treatment	Diff	p(diff!=0)
Avg. audience ideology - FB exposure	0.524	0.524	0.000	p=0.61
Avg. audience ideology - web visits	0.493	0.495	0.002	p=0.71

N=3397. 19980 observations (85.47%) dropped by listwise deletion. The last column reports the unadjusted p-value from a two-sided test of the hypothesis of no difference between treatment and control groups on each metric, computed using the baseline OLS model (see Section S1.5.1).

Table 35 shows the percentage of exposure to content classified as civic that includes links to news domains, content from misinformation repeat offenders, content classified as uncivil, and content classified as containing one or more slur words.

Table 35: Exposure to different types of content on Facebook

Metric	Control	Treatment	Diff	p(diff!=0)
% civic content	13.470	12.787	-0.683	p=0.00
% content with news links	10.206	10.527	0.322	p=0.00
% content from misinfo. ROs	0.757	0.549	-0.208	p=0.00
% content classified as uncivil	3.151	2.813	-0.338	p=0.00
% content with slur words	0.034	0.030	-0.004	p=0.00

N=23228. 149 observations (0.64%) dropped by listwise deletion. The last column reports the unadjusted p-value from a two-sided test of the hypothesis of no difference between treatment and control groups on each metric, computed using the baseline OLS model (see Section S1.5.1).

Table 36 reports the percentage of views of posts that meet both of the following criteria by treatment group:[3]

- They include links to domains or posts from Pages or groups that have had one or more lifetime strikes for content that violates Facebook's Community Standards for the category in question.
- The domain, Page, or group has two or more lifetime strikes overall across all categories.

Table 36: Percentage of views on sources with 2+ strikes overall and 1+ strikes for...

Metric	Control	Treatment	Diff	p(diff!=0)
Violence and Incitement	0.38	0.32	-0.05	p=0.01
Misinformation (including harmful health misinformation)	0.13	0.12	-0.02	p=0.12
Dangerous Individuals and Organizations	0.35	0.37	0.02	p=0.40
Voter Suppression and Fraud	0.07	0.06	-0.01	p=0.42
Other types of Coordinating Harm	0.02	0.03	0.00	p=0.35
Bullying and Harassment	0.45	0.44	-0.01	p=0.69
Content Spam	1.58	1.65	0.06	p=0.02
Hate Speech	0.57	0.50	-0.06	p=0.01

N=23228. 149 observations (0.64%) dropped by listwise deletion. The last column reports the unadjusted p-value from a two-sided test of the hypothesis of no difference between treatment and control groups on each metric, computed using the baseline OLS model (see Section S1.5.1).

Table 37 shows the percentage of views on content from 26 distinct types of Facebook's content categories among the treatment and the control group.

[3]We applied this dual threshold rather than our initial pre-registered threshold of two or more strikes on each category because content is removed after only one strike for some policy violations (e.g., Dangerous Individuals and Organizations) so no entity would meet the two-strike threshold. For additional details on how strikes are applied and counted, see https://transparency.fb.com/enforcement/taking-action/counting-strikes/.

Table 37: Exposure to content about different topic categories (% of views)

Metric	Control	Treatment	Diff	p(diff!=0)
Animals & Pets	5.24	5.45	0.21	p=0.00
Books & Literature	0.78	0.79	0.01	p=0.40
Business, Finance & Economics	1.51	1.55	0.04	p=0.03
Crime & Tragedy	3.02	3.07	0.05	p=0.06
Education & Learning	2.06	2.03	-0.03	p=0.31
Fashion & Style	4.46	4.53	0.07	p=0.22
Children & Parenting	7.94	7.83	-0.11	p=0.02
Fitness & Workouts	0.67	0.76	0.08	p=0.00
Food & Drink	5.08	5.37	0.29	p=0.00
Games, Puzzles & Play	2.31	2.56	0.26	p=0.00
Health & Medical	6.05	6.08	0.03	p=0.24
History & Philosophy	0.36	0.38	0.02	p=0.09
Holidays & Celebrations	5.83	5.65	-0.18	p=0.00
Home & Garden	5.61	5.63	0.01	p=0.90
Music & Audio	3.03	3.19	0.16	p=0.00
Performing Arts	0.32	0.35	0.03	p=0.00
Politics	9.62	8.96	-0.66	p=0.00
Relationships, Friends & Family	7.02	6.88	-0.14	p=0.00
Religion & Spirituality	2.63	2.58	-0.05	p=0.22
Science & Tech	2.80	2.89	0.09	p=0.00
Social Issues	7.96	7.61	-0.35	p=0.00
Sports	2.06	2.22	0.15	p=0.00
Travel & Leisure Activities	2.31	2.41	0.09	p=0.00
TV & Movies	6.09	6.29	0.20	p=0.00
Vehicles & Transportation	3.96	3.85	-0.10	p=0.08
Visual Arts, Architecture & Crafts	4.99	5.12	0.13	p=0.00

N=23228. 149 observations (0.64%) dropped by listwise deletion. The last column reports the unadjusted p-value from a two-sided test of the hypothesis of no difference between treatment and control groups on each metric, computed using the baseline OLS model (see Section S1.5.1).

Table 38 shows the percentage of views on content classified as civic about the 26 distinct types of Facebook's content categories among the treatment and the control group.

Table 39 shows the percentage of views from strong ties versus weak ties among the treatment and the control group. Strong ties are defined (for each user) as the top 20% users or the top 100 friends (whichever is smallest) with whom he/she had most interactions.

Table 38: Exposure to civic content about different topic categories (% of views of civic content)

Metric	Control	Treatment	Diff	p(diff!=0)
Animals & Pets	0.61	0.70	0.09	p=0.00
Books & Literature	0.19	0.19	-0.00	p=0.92
Business, Finance & Economics	3.20	3.43	0.23	p=0.00
Crime & Tragedy	8.22	8.46	0.23	p=0.00
Education & Learning	3.22	3.29	0.07	p=0.13
Fashion & Style	0.57	0.57	0.00	p=0.87
Children & Parenting	3.17	3.17	-0.00	p=0.66
Fitness & Workouts	0.14	0.18	0.03	p=0.12
Food & Drink	0.35	0.38	0.03	p=0.00
Games, Puzzles & Play	0.22	0.23	0.01	p=0.26
Health & Medical	24.42	25.14	0.72	p=0.00
History & Philosophy	0.39	0.42	0.03	p=0.04
Holidays & Celebrations	1.36	1.42	0.06	p=0.00
Home & Garden	0.72	0.76	0.04	p=0.01
Music & Audio	0.80	0.84	0.04	p=0.10
Performing Arts	0.10	0.11	0.01	p=0.17
Politics	62.49	62.24	-0.26	p=0.56
Relationships, Friends & Family	0.72	0.71	-0.01	p=0.51
Religion & Spirituality	1.43	1.36	-0.08	p=0.01
Science & Tech	1.61	1.72	0.10	p=0.00
Social Issues	57.62	57.86	0.24	p=0.07
Sports	0.78	0.86	0.08	p=0.00
Travel & Leisure Activities	0.97	1.05	0.08	p=0.00
TV & Movies	2.26	2.31	0.05	p=0.17
Vehicles & Transportation	1.57	1.59	0.02	p=0.30
Visual Arts, Architecture & Crafts	0.99	0.94	-0.05	p=0.00

N=23165. 212 observations (0.91%) dropped by listwise deletion. The last column reports the unadjusted p-value from a two-sided test of the hypothesis of no difference between treatment and control groups on each metric, computed using the baseline OLS model (see Section S1.5.1).

Table 39: Exposure to content from strong ties (% of views of user content)

Metric	Control	Treatment	Diff	p(diff!=0)
% content from strong ties	50.90	47.52	-3.38	p=0.00

N=23191. 186 observations (0.8%) dropped by listwise deletion. The last column reports the unadjusted p-value from a two-sided test of the hypothesis of no difference between treatment and control groups on each metric, computed using the baseline OLS model (see Section S1.5.1).

3.2 Treatment effects: Behavior

This section describes the effects of our treatment on on-platform behavior.

Table 40 shows the effects of our treatment on how participants used the platform in terms of the days they were active on the platform, the time spent on the platform per day, and how many on-platform views they had during the study period.

Table 40: Effects of treatment on platform usage

Metric	Group	p2.5	p50	p97.5	Avg.	SD	N	p(diff!=0)
Days active during study period	Control	47	90	90	86.2	11.6	16075	–
	Treatment	44.7	90	90	85.9	12	7187	p=0.07
Standardized time spent per day	Control	-0.6	0.3	2.7	0.5	0.9	16075	–
	Treatment	-0.6	0.3	2.6	0.5	0.9	7187	p=0.07
Count of views per day	Control	5.7	203.1	922.8	267	237.7	16075	–
	Treatment	5.3	189.3	913.5	254.9	234.7	7187	p=0.00
Count of posts per day	Control	0	0.5	10.1	1.4	2.7	16075	–
	Treatment	0	0.5	9.5	1.4	2.5	7187	p=0.14
Count of clicks per day	Control	0.5	13.1	61.6	17.5	15.8	16075	–
	Treatment	0.4	11.9	58.3	16.1	15.1	7187	p=0.00
Count of reactions per day	Control	0	5.1	69.2	12.1	17.7	16075	–
	Treatment	0	4.5	59.1	10.8	16.1	7187	p=0.00
Count of comments per day	Control	0	0.8	11.8	2	3.1	16075	–
	Treatment	0	0.7	10.5	1.8	2.8	7187	p=0.00
Count of reshares per day	Control	0	0.1	7.4	0.8	2	16075	–
	Treatment	0	0.1	6.4	0.8	1.9	7187	p=0.06

115 observations (0.49%) dropped by listwise deletion. The last column reports the unadjusted p-value from a two-sided test of the hypothesis of no difference between treatment and control groups on each metric, computed using the baseline OLS model (see Section S1.5.1).

Table 41 shows the effects of the treatment on rates of engagement (i.e., engagement conditional on exposure) with content from like-minded sources. Engagement includes clicking anywhere in the post (e.g., a link click, "see more," profile click), reacting (e.g., Like, Love, Sad), commenting, and resharing.

Table 42 shows the effects of the treatment on rates of engagement (i.e., engagement conditional on exposure) with cross-cutting content. Engagement includes clicking anywhere in the post (e.g., a link click, "see more," profile click), reacting (e.g., Like, Love, Sad), commenting, and resharing.

Table 41: Effects of treatment on engagement with content from like-minded sources, conditional on exposure

Metric	Group	p2.5	p50	p97.5	Avg.	SD	N	p(diff!=0)
Click rate	Control	1.8	6.8	20.4	8	12.8	16033	–
	Treatment	1.8	7.3	20	8.3	5.4	7168	p=0.06
Reaction rate	Control	0	3.4	35.2	7.2	10.4	16033	–
	Treatment	0	3.9	36.5	7.6	10.4	7168	p=0.00
Comment rate	Control	0	0.4	7.5	1.2	2.9	16033	–
	Treatment	0	0.4	8.4	1.4	3.7	7168	p=0.00
Reshare rate	Control	0	0.1	3.7	0.5	1.9	16033	–
	Treatment	0	0.1	6	0.8	4.7	7168	p=0.00

176 observations (0.75%) dropped by listwise deletion. The last column reports the unadjusted p-value from a two-sided test of the hypothesis of no difference between treatment and control groups on each metric, computed using the baseline OLS model (see Section S1.5.1).

Table 42: Effects of treatment on engagement with content from cross-cutting sources, conditional on exposure

Metric	Group	p2.5	p50	p97.5	Avg.	SD	N	p(diff!=0)
Click rate	Control	1.5	7	21.3	8.4	30.2	16005	–
	Treatment	1.5	6.4	18.5	7.3	4.8	7166	p=0.00
Reaction rate	Control	0	3.1	33.7	6.7	10.1	16005	–
	Treatment	0	2.6	30.4	5.8	8.3	7166	p=0.00
Comment rate	Control	0	0.4	9.5	2.1	60.6	16005	–
	Treatment	0	0.3	6.7	1.1	2.8	7166	p=0.03
Reshare rate	Control	0	0	4.1	0.6	4.2	16005	–
	Treatment	0	0	2.8	0.4	1.5	7166	p=0.00

206 observations (0.88%) dropped by listwise deletion. The last column reports the unadjusted p-value from a two-sided test of the hypothesis of no difference between treatment and control groups on each metric, computed using the baseline OLS model (see Section S1.5.1).

Table 43 shows the effects of the treatment on the production of and engagement with civic content among the treatment and the control group. Production includes posting and engagement includes clicking anywhere in the post (e.g., a link click, "see more," profile click), reacting (e.g., Like, Love, Sad), commenting, and resharing.

Table 43: Effects of treatment on production and engagement with civic content

Metric	Group	p2.5	p50	p97.5	Avg.	SD	N	p(diff!=0)
% of posts created that are civic	Control	0	7.4	66.7	14.6	18.4	15207	–
	Treatment	0	7	64.1	13.8	18	6790	p=0.02
% of clicks on civic content	Control	1.3	13.3	47.7	16.4	12.6	15207	–
	Treatment	1.5	13.1	47.8	16.1	12.2	6790	p=0.32
Reaction rate on civic content	Control	0	2.3	31.8	5.9	10.3	15207	–
	Treatment	0	2.2	28.3	5.3	7.9	6790	p=0.00
Comment rate on civic content	Control	0	0.3	9.9	1.6	7.4	15207	–
	Treatment	0	0.3	8	1.3	3.1	6790	p=0.00
Reshare rate on civic content	Control	0	0.1	5.1	0.8	6.7	15207	–
	Treatment	0	0	4.8	0.7	4.1	6790	p=0.18

1380 observations (5.9%) dropped by listwise deletion. The last column reports the unadjusted p-value from a two-sided test of the hypothesis of no difference between treatment and control groups on each metric, computed using the baseline OLS model (see Section S1.5.1).

Table 44 shows the effects of the treatment on the count of negative actions taken by users against content from like-minded or cross-cutting friends, Pages, or groups (e.g., hiding a post) or the friends, Pages, and groups themselves (e.g., snoozing, blocking, unfriending, unfollowing).

Table 45 shows the effects of the treatment on rates of engagement with content produced by misinformation repeat offenders (see definition in Section 1.3 above). Engagement includes clicking anywhere in the post (e.g., a link click, "see more," profile click), reacting (e.g., Like, Love, Sad), commenting, and resharing.

Table 44: Effects of treatment on count of negative actions on content, users, and entities

Metric	Group	p2.5	p50	p97.5	Avg.	SD	N	p(diff!=0)
Content from like-minded sources	Control	0	0	3	0.2	0.8	16075	–
	Treatment	0	0	2	0.2	0.8	7187	p=0.01
Like-minded users	Control	0	0	21	2.2	6.6	16075	–
	Treatment	0	0	18	2.1	6.5	7187	p=0.26
Like-minded Pages and groups	Control	0	0	16	1.8	4.7	16075	–
	Treatment	0	0	15	1.6	4.5	7187	p=0.00
Content from cross-cutting sources	Control	0	0	3	0.2	0.8	16075	–
	Treatment	0	0	3	0.3	0.9	7187	p=0.00
Cross-cutting users	Control	0	0	20	2.2	6.2	16075	–
	Treatment	0	0	22	2.4	6.4	7187	p=0.08
Cross-cutting Pages and groups	Control	0	0	9	0.9	2.6	16075	–
	Treatment	0	0	9	1	2.7	7187	p=0.01

115 observations (0.49%) dropped by listwise deletion. The last column reports the unadjusted p-value from a two-sided test of the hypothesis of no difference between treatment and control groups on each metric, computed using the baseline OLS model (see Section S1.5.1).

Table 45: Effects of treatment on engagement with content from misinformation repeat offenders

Metric	Group	p2.5	p50	p97.5	Avg.	SD	N	p(diff!=0)
Click rate	Control	0	0	37.5	5.3	12.9	15114	–
	Treatment	0	0	37.5	5.2	17.5	6758	p=0.43
Reaction rate	Control	0	0	37.5	5.3	12.9	15114	–
	Treatment	0	0	37.5	5.2	17.5	6758	p=0.43
Comment rate	Control	0	0	9.3	1	4.8	15114	–
	Treatment	0	0	10	1	6.2	6758	p=0.54
Reshare rate	Control	0	0	8.1	1	10.8	15114	–
	Treatment	0	0	8.3	1.2	29.4	6758	p=0.60

1505 observations (6.44%) dropped by listwise deletion. The last column reports the unadjusted p-value from a two-sided test of the hypothesis of no difference between treatment and control groups on each metric, computed using the baseline OLS model (see Section S1.5.1).

3.3 Treatment effects on outcomes for primary hypotheses (PATE/SATE)

As per the pre-registration, we examined the effects of the treatment for outcome variables specified in our primary hypotheses. Extended Data Figure 4 shows the results of the treatment.

Table 46: Treatment effects on outcomes for primary hypotheses

Hypothesis	Est	ATE	95% CI	SE	N	p	Adj. p
H1: Affective polarization	PATE	-0.004	[-0.029, 0.022]	0.013	22127	0.785	1.000
	SATE	-0.017	[-0.031, -0.003]	0.007	22127	0.021	0.198
H2: Ideological extremity	PATE	-0.004	[-0.036, 0.028]	0.016	21154	0.815	1.000
	SATE	-0.012	[-0.031, 0.006]	0.010	21154	0.197	1.000
H3a: Consistency of issue positions	PATE	0.024	[-0.027, 0.075]	0.026	21159	0.353	1.000
	SATE	-0.003	[-0.030, 0.024]	0.014	21159	0.844	1.000
H3b: Consistency of racial group evaluations	PATE	-0.007	[-0.030, 0.015]	0.012	21162	0.524	1.000
	SATE	0.000	[-0.012, 0.012]	0.006	21162	0.987	1.000
H4a: Consistency of vote choice / cand. evals	PATE	0.056	[0.001, 0.110]	0.028	21158	0.044	0.552
	SATE	0.005	[-0.022, 0.033]	0.014	21158	0.717	1.000
H5a: Party-congenial beliefs	PATE	0.027	[-0.028, 0.082]	0.028	20442	0.333	1.000
	SATE	-0.002	[-0.031, 0.026]	0.015	20442	0.874	1.000
H5b: Confidence in elections	PATE	0.009	[-0.044, 0.062]	0.027	20442	0.744	1.000
	SATE	-0.009	[-0.037, 0.020]	0.014	20442	0.555	1.000
H5c: Respect for election norms	PATE	0.019	[-0.036, 0.073]	0.028	20390	0.502	1.000
	SATE	-0.014	[-0.042, 0.014]	0.014	20390	0.313	1.000

Estimates based on baseline OLS model (see Section S1.5.1). The last two columns report unadjusted and adjusted p-values from a two-sided t-test of the null hypothesis that each coefficient equals zero.

3.4 Treatment effects on outcomes for research questions

As per the pre-registration, we examined the effects of the treatment for outcome variables specified in our research questions. Extended Data Figure 5 shows the results of the treatment.

Table 47: Treatment effects on outcomes for research questions

Hypothesis	Est	ATE	95% CI	SE	N	p	Adj. p
RQ1a: Validated turnout	PATE	-0.051	[-0.131, 0.029]	0.041	10330	0.210	1.000
	SATE	-0.030	[-0.072, 0.011]	0.021	10330	0.151	0.469
RQ1b: Self-reported turnout	PATE	-0.014	[-0.067, 0.039]	0.027	22139	0.607	1.000
	SATE	-0.021	[-0.047, 0.005]	0.013	22139	0.114	0.371
RQ1c: Self-reported participation	PATE	0.000	[-0.032, 0.033]	0.016	21162	0.985	1.000
	SATE	-0.010	[-0.031, 0.010]	0.010	21162	0.326	0.721
RQ1d: On-platform political participation (Dim 1)	PATE	-0.003	[-0.015, 0.010]	0.006	23377	0.669	1.000
	SATE	-0.020	[-0.037, -0.003]	0.009	23377	0.024	0.122
RQ2a: perceived % of congenial friends	PATE	-0.006	[-0.053, 0.040]	0.024	21071	0.791	1.000
	SATE	-0.030	[-0.058, -0.003]	0.014	21071	0.032	0.122
RQ2b: perceived % of uncongenial friends	PATE	0.005	[-0.048, 0.058]	0.027	21070	0.848	1.000
	SATE	0.036	[0.007, 0.065]	0.015	21070	0.015	0.122
RQ2c: Seen pol. conv. on FB	PATE	0.018	[-0.043, 0.079]	0.031	21355	0.561	1.000
	SATE	-0.009	[-0.038, 0.020]	0.015	21355	0.531	0.895
RQ2d: Pol. conv. on FB were respectful	PATE	-0.015	[-0.065, 0.035]	0.026	19061	0.561	1.000
	SATE	-0.033	[-0.063, -0.003]	0.015	19061	0.031	0.122
RQ2e: Pol. conv. on FB were informative	PATE	-0.038	[-0.085, 0.010]	0.024	19063	0.122	1.000
	SATE	-0.047	[-0.076, -0.017]	0.015	19063	0.002	0.024
RQ3: Perceived polarization	PATE	-0.005	[-0.044, 0.035]	0.020	21157	0.815	1.000
	SATE	0.003	[-0.019, 0.025]	0.011	21157	0.767	1.000
RQ4a: In-party affinity	PATE	-0.006	[-0.036, 0.025]	0.015	22136	0.713	1.000
	SATE	-0.035	[-0.052, -0.017]	0.009	22136	0.000	0.003
RQ4c: Out-party antipathy	PATE	-0.009	[-0.040, 0.022]	0.016	22130	0.574	1.000
	SATE	-0.009	[-0.027, 0.009]	0.009	22130	0.348	0.721
RQ5a: Belief in false claims	PATE	-0.012	[-0.049, 0.026]	0.019	22136	0.539	1.000
	SATE	-0.000	[-0.022, 0.022]	0.011	22136	0.983	1.000
RQ5b: Belief in pro-attitudinal false claims	PATE	0.007	[-0.034, 0.047]	0.021	22135	0.742	1.000
	SATE	-0.010	[-0.035, 0.015]	0.013	22135	0.417	0.776
RQ5c: Accurate knowledge of current events	PATE	0.000	[-0.037, 0.038]	0.019	19414	0.996	1.000
	SATE	-0.009	[-0.031, 0.013]	0.011	19414	0.426	0.776
RQ5d: Accurate pro-attitudinal knowledge of events	PATE	-0.025	[-0.071, 0.021]	0.024	19398	0.293	1.000
	SATE	-0.012	[-0.039, 0.014]	0.013	19398	0.349	0.721
RQ6: Perceptions of democratic performance	PATE	-0.009	[-0.048, 0.030]	0.020	22138	0.662	1.000
	SATE	-0.007	[-0.031, 0.016]	0.012	22138	0.529	0.895
RQ7: Ideological extremity of web visits	PATE	-0.028	[-0.141, 0.084]	0.057	3432	0.623	1.000
	SATE	-0.031	[-0.096, 0.034]	0.033	3432	0.349	0.721

Estimates based on baseline OLS model (see Section S1.5.1). The last two columns report unadjusted and adjusted p-values from a two-sided t-test of the null hypothesis that each coefficient equals zero.

3.5 Treatment effects on auxiliary outcome measures

For a number of our outcome measures, the pre-registration specified that we would construct a composite scale using exploratory factor analysis with varimax rotation to select items, and that those that did not clearly load on the same underlying dimension would be analyzed separately. When conducting analyses, we included each item in the scale only if its factor analysis loading was at least .7 and missingness was less than .15. In this section, we report all items that were excluded from preregistered indices or scales because they did not load (see Section 4.1 for factor loadings). Figure 10 analyzes these as auxiliary tests with unadjusted p-values. Note that this is a deviation from the pre-registration, which originally stated that "All items that are excluded from preregistered indices or scales are considered to be primary outcomes." The reason for this deviation (as explained in the deviation document) is that we do not have pre-registered theoretical expectations for individual outcome measures that do not load with the other items in the scale: indeed, the fact that these items did not load with the other items suggests that either they were measured with error or did not capture the underlying construct.

The list of measures included is below, along with the hypothesis or research question each was originally a part of and the reason it was excluded from the scale.

- H4: Senate vote choice consistent with ideology (missingness above .15)
- H4: Governor vote choice consistent with ideology (missingness above .15)
- H4: Presidential candidate evaluations consistent with ideology (Wave 5, since only Wave 4 was included in the final measure)
- H5: Belief that registered voters were prevented from voting (did not load with other measures)
- H5: Belief that the fact registered voters were prevented from voting changed the election outcome (did not load with other measures)
- RQ1: Items that loaded above .7 on the second dimension of on-platform participation: shares, comments, petition shares, reshares of politicians' content.
- RQ1: Items that loaded above .7 on the third dimension of on-platform participation: clicks and contacts on Town Hall.
- RQ4: Perceptions of how smart people are who support the congenial party (was not asked in Wave 5)
- RQ4: Perception of how smart people are who support the non-congenial party (was not asked in Wave 5)

Figure 9 shows the treatment effects on the outcomes that were classified as auxiliary. Table 48 shows the estimates from 9 in tabular format.

Figure 9: Treatment effects on auxiliary outcomes

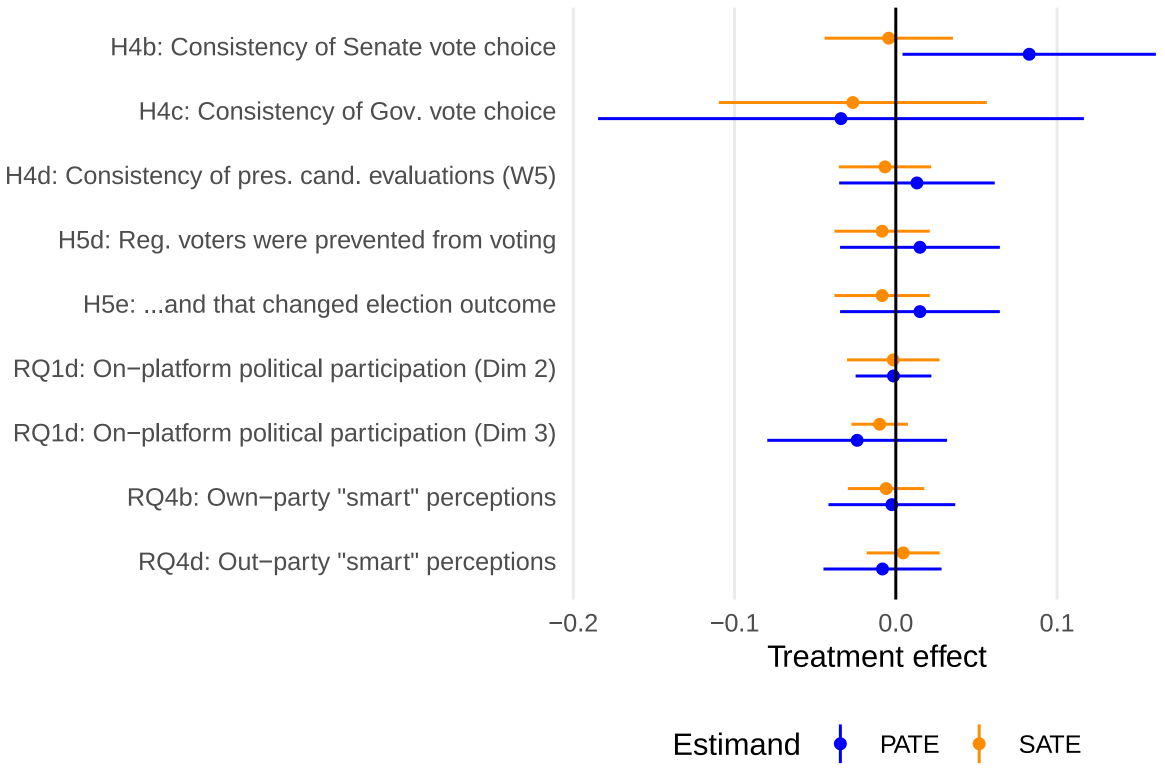

Average treatment effects of reducing exposure to like-minded sources in the Facebook Feed from September 24–December 23, 2020. The figure shows OLS estimates of sample average treatment effects (SATE) as well as population average treatment effect (PATE) using survey weights and HC2 robust standard errors. Engagement outcome measures were measured using Feed behavior by participants. Survey outcome measures are standardized scales averaged across surveys conducted November 4–18, 2020 and/or December 9–23, 2020, unless indicated otherwise. Sample size and p-values for each estimate are reported in Table 48.

Table 48: Treatment effects on auxiliary outcomes

Hypothesis	Est	ATE	95% CI	SE	N	p
H4b: Consistency of Senate vote choice	PATE	0.083	[0.004, 0.161]	0.040	10686	0.039
	SATE	-0.004	[-0.044, 0.035]	0.020	10686	0.829
H4c: Consistency of Gov. vote choice	PATE	-0.034	[-0.185, 0.117]	0.077	2689	0.658
	SATE	-0.027	[-0.110, 0.056]	0.042	2689	0.529
H4d: Consistency of pres. cand. evaluations (W5)	PATE	0.013	[-0.035, 0.061]	0.025	20380	0.594
	SATE	-0.007	[-0.035, 0.022]	0.015	20380	0.646
H5d: Reg. voters were prevented from voting	PATE	0.015	[-0.035, 0.065]	0.025	20441	0.554
	SATE	-0.008	[-0.038, 0.021]	0.015	20441	0.573
H5e: ...and that changed election outcome	PATE	0.015	[-0.035, 0.065]	0.025	20441	0.554
	SATE	-0.008	[-0.038, 0.021]	0.015	20441	0.573
RQ1d: On-platform political participation (Dim 2)	PATE	-0.001	[-0.025, 0.022]	0.012	23377	0.905
	SATE	-0.002	[-0.030, 0.027]	0.015	23377	0.915
RQ1d: On-platform political participation (Dim 3)	PATE	-0.024	[-0.080, 0.032]	0.028	23377	0.399
	SATE	-0.010	[-0.028, 0.008]	0.009	23377	0.263
RQ4b: Own-party ”smart” perceptions	PATE	-0.002	[-0.042, 0.037]	0.020	21143	0.903
	SATE	-0.006	[-0.030, 0.018]	0.012	21143	0.618
RQ4d: Out-party ”smart” perceptions	PATE	-0.008	[-0.045, 0.028]	0.019	21145	0.659
	SATE	0.005	[-0.018, 0.027]	0.012	21145	0.692

Estimates based on baseline OLS model (see Section S1.5.1). The last two columns report unadjusted and adjusted p-values from a two-sided t-test of the null hypothesis that each coefficient equals zero.

3.6 Passive and active engagement component measures

As described in Section 4.2, clicks are counted in our analysis whenever a respondent clicked on any component of a post, which includes clicks to add a like or reaction. As a result, metrics that aggregate clicks with other engagement types, such as the passive engagement metrics that we report in Figure 3 of the main text, contain some inherent measurement error. To address this concern, Table 49 reports treatment effect estimates for the passive engagement metrics reported in the main text that are disaggregated into their two main components: reactions (which includes likes) and clicks. Similarly, we are not able to disaggregate reshare actions between the Feed and other surfaces. As a result, our measure combining comments and reshares creates a potential inconsistency because we measure comments in Feed but reshares across all surfaces. To demonstrate that this combination is not a problem for the analysis, Table 49 also disaggregates these two engagement types. In all cases, the patterns we find for our aggregated passive and active engagement metrics hold when they are split into their two main subcomponents.

Table 49: Treatment effects on total passive engagement and passive engagement rate, disaggregated by engagement type

Outcome measure	Estimand	ATE	95% CI	SE	N	p
Panel B: Total engagement						
Clicks on posts from like-minded sources	SATE	-0.27	[-0.293, -0.246]	0.01	23377	0.00
Likes+reactions on posts from like-minded sources	SATE	-0.17	[-0.194, -0.150]	0.01	23377	0.00
Comments on posts from like-minded sources	SATE	-0.12	[-0.145, -0.098]	0.01	23377	0.00
Reshares of posts from like-minded sources	SATE	-0.07	[-0.097, -0.052]	0.01	23377	0.00
Clicks on civic posts from like-minded sources	SATE	-0.16	[-0.185, -0.137]	0.01	23377	0.00
Likes+reactions on civic posts from like-minded sources	SATE	-0.10	[-0.121, -0.074]	0.01	23377	0.00
Comments on civic posts from like-minded sources	SATE	-0.06	[-0.087, -0.028]	0.02	23377	0.00
Reshares of civic posts from like-minded sources	SATE	-0.04	[-0.068, -0.015]	0.01	23377	0.00
Clicks on posts from cross-cutting sources	SATE	0.11	[0.084, 0.141]	0.01	23377	0.00
Likes+reactions on posts from cross-cutting sources	SATE	0.08	[0.053, 0.110]	0.01	23377	0.00
Comments on posts from cross-cutting sources	SATE	0.04	[0.006, 0.065]	0.02	23377	0.02
Reshares of posts from cross-cutting sources	SATE	0.03	[-0.005, 0.063]	0.02	23377	0.09
Clicks on content from misinfo. repeat offenders	SATE	-0.08	[-0.108, -0.053]	0.01	23377	0.00
Likes+reactions on posts from misinfo. repeat offenders	SATE	-0.05	[-0.075, -0.016]	0.02	23377	0.00
Comments on posts from misinfo. repeat offenders	SATE	-0.00	[-0.047, 0.039]	0.02	23377	0.86
Reshares of posts from misinfo. repeat offenders	SATE	-0.03	[-0.051, -0.003]	0.01	23377	0.03
Panel C: Engagement rate						
Clicks on posts from like-minded sources	SATE	0.02	[-0.000, 0.036]	0.01	23316	0.05
Likes+reactions on posts from like-minded sources	SATE	0.05	[0.024, 0.078]	0.01	23316	0.00
Comments on posts from like-minded sources	SATE	0.07	[0.034, 0.101]	0.02	23316	0.00
Reshares of posts from like-minded sources	SATE	0.15	[0.096, 0.210]	0.03	23316	0.00
Clicks on civic posts from like-minded sources	SATE	0.01	[-0.013, 0.039]	0.01	23148	0.31
Likes+reactions on civic posts from like-minded sources	SATE	-0.02	[-0.040, 0.009]	0.01	23148	0.22
Commments on civic posts from like-minded sources	SATE	-0.01	[-0.031, 0.009]	0.01	23148	0.29
Reshares of civic posts from like-minded sources	SATE	-0.00	[-0.023, 0.021]	0.01	23148	0.93
Clicks on posts from cross-cutting sources	SATE	-0.04	[-0.052, -0.020]	0.01	23286	0.00
Likes+reactions on posts from cross-cutting sources	SATE	-0.09	[-0.111, -0.064]	0.01	23286	0.00
Comments on posts from cross-cutting sources	SATE	-0.02	[-0.032, -0.001]	0.01	23286	0.03
Reshares of posts from cross-cutting sources	SATE	-0.05	[-0.070, -0.036]	0.01	23286	0.00
Clicks on posts from misinfo. repeat offenders	SATE	-0.01	[-0.040, 0.014]	0.01	21979	0.34
Likes+reactions on posts from misinfo. repeat offenders	SATE	-0.01	[-0.050, 0.022]	0.02	21979	0.45
Comments on posts from misinfo. repeat offenders	SATE	0.01	[-0.024, 0.044]	0.02	21979	0.57
Reshares of posts from misinfo. repeat offenders	SATE	0.02	[-0.048, 0.087]	0.03	21979	0.58

Estimates based on baseline OLS model (see Section S1.5.1). The last columns reports unadjusted p-values from a two-sided t-test of the null hypothesis that each coefficient equals zero.

3.7 Additional analyses

As per the pre-registration, Figure 10 examines the effects of the treatment on a range of other outcomes, all measured with survey data. Unless otherwise noted, data are from Wave 5 of the survey. Below is a brief description of how each outcome was operationalized. See the pre-registration and survey instrument for more details and specific question wording.

- Issue polarization: an index of standardized responses to six issue opinion questions re-signed so that on each question higher values are closer to the congenial-party mean and lower values are closer to the non-congenial-party mean (congenial with respect to the respondent's self-reported party identification). The issues included questions on immigration, health care, unemployment, COVID, foreign policy, and police funding (survey items IMMIG, HEALTH, UNEMPLOY, COVID, POLICE).
- Election knowledge: the proportion correct on an six-question battery of candidate policy positions.
- News knowledge: the proportion correct on a seven-question battery assessing knowledge about events currently in the news (survey item SPECKNOWPO).
- #MeToo feeling thermometer: a 1-100 feeling thermometer about the #MeToo movement (FTPEOPLEGROUPS).
- Agree/disagree with statement that "Most women interpret innocent remarks or acts as being sexist" (SEXISI12).
- Agree/disagree with statement that "Recent allegations of sexual harassment and assault reflect widespread problems in society." (SEXISI12).
- Satisfaction with Facebook: 1–7 scale from "completely dissatisfied" to "completely satisfied" (FBSAT)
- Frequency of feeling anxious over the last four weeks (1–5 scale from "never" to "all the time") (EMOT).
- Frequency of feeling depressed over the last four weeks (1–5 scale from "never" to "all the time") (EMOT).

Table 50 shows the results of Figure 10 in tabular format.

Figure 10: Treatment effects on additional outcomes

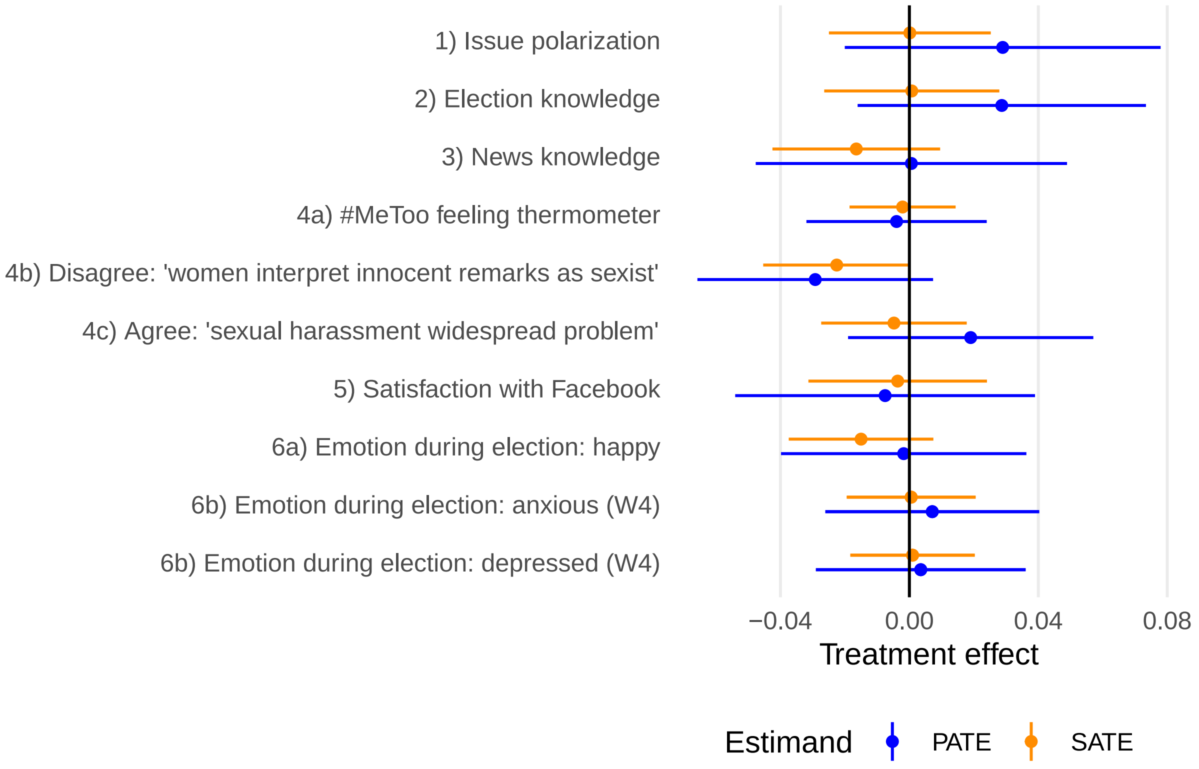

Average treatment effects of reducing exposure to like-minded sources in the Facebook Feed from September 24–December 23, 2020. The figure shows OLS estimates of sample average treatment effects (SATE) as well as population average treatment effect (PATE) using survey weights and HC2 robust standard errors. Survey outcome measures are standardized scales averaged across surveys conducted November 4–18, 2020 and/or December 9–23, 2020, unless indicated otherwise. Sample size and p-values for each estimate are reported in Table 50.

Table 50: Treatment effects on additional outcomes

Hypothesis	Est	ATE	95% CI	SE	N	p
1) Issue polarization	PATE	0.029	[-0.020, 0.078]	0.025	21161	0.247
	SATE	0.000	[-0.025, 0.025]	0.013	21161	0.991
2) Election knowledge	PATE	0.029	[-0.016, 0.073]	0.023	21162	0.209
	SATE	0.001	[-0.026, 0.028]	0.014	21162	0.957
3) News knowledge	PATE	0.001	[-0.048, 0.049]	0.025	21162	0.980
	SATE	-0.016	[-0.042, 0.010]	0.013	21162	0.215
4a) #MeToo feeling thermometer	PATE	-0.004	[-0.032, 0.024]	0.014	21127	0.780
	SATE	-0.002	[-0.019, 0.014]	0.008	21127	0.801
4b) Disagree: 'women interpret innocent remarks as sexist'	PATE	-0.029	[-0.066, 0.007]	0.019	21155	0.117
	SATE	-0.023	[-0.045, 0.000]	0.012	21155	0.053
4c) Agree: 'sexual harassment widespread problem'	PATE	0.019	[-0.019, 0.057]	0.019	21156	0.327
	SATE	-0.005	[-0.027, 0.018]	0.012	21156	0.678
5) Satisfaction with Facebook	PATE	-0.008	[-0.054, 0.039]	0.024	20356	0.751
	SATE	-0.004	[-0.031, 0.024]	0.014	20356	0.797
6a) Emotion during election: happy	PATE	-0.002	[-0.040, 0.036]	0.019	22141	0.928
	SATE	-0.015	[-0.037, 0.007]	0.011	22141	0.190
6b) Emotion during election: depressed (W4)	PATE	0.004	[-0.029, 0.036]	0.017	22142	0.832
	SATE	0.001	[-0.018, 0.020]	0.010	22142	0.921
6b) Emotion during election: depressed (W4)	PATE	0.004	[-0.029, 0.036]	0.017	22142	0.832
	SATE	0.001	[-0.018, 0.020]	0.010	22142	0.921
6b) Emotion during election: anxious (W4)	PATE	0.007	[-0.026, 0.040]	0.017	22142	0.675
	SATE	0.001	[-0.019, 0.021]	0.010	22142	0.956
6b) Emotion during election: anxious (W4)	PATE	0.007	[-0.026, 0.040]	0.017	22142	0.675
	SATE	0.001	[-0.019, 0.021]	0.010	22142	0.956

Estimates based on baseline OLS model (see Section S1.5.1). The last two columns report unadjusted and adjusted p-values from a two-sided t-test of the null hypothesis that each coefficient equals zero.

3.8 Wave-specific analyses

As per the pre-registration, we also examined the effects of the treatment for outcome variables that were measured in multiple survey waves separately by wave. Note that not all outcome variables were measured in multiple survey waves. Figure 11 shows the results of the treatment for those that were.

Figure 11: Treatment effects by survey wave

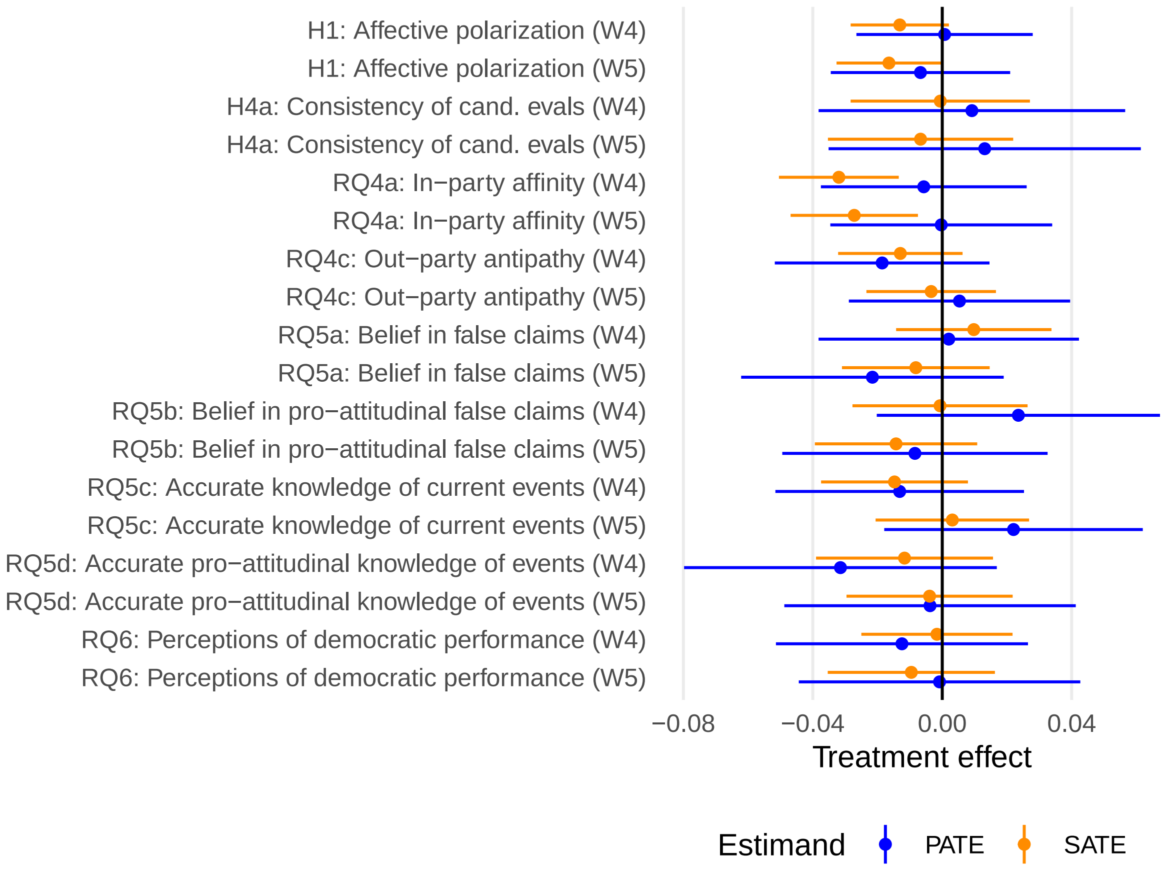

Average treatment effects of reducing exposure to like-minded sources in the Facebook Feed from September 24–December 23, 2020. The figure shows OLS estimates of sample average treatment effects (SATE) as well as population average treatment effect (PATE) using survey weights and HC2 robust standard errors. Sample size and p-values for each estimate are reported in Table 51.

Table 51 shows the results of 11 in tabular format.

Table 51: Treatment effects by survey wave

Hypothesis	Est	ATE	95% CI	SE	N	p
H1: Affective polarization (W4)	PATE	0.001	[-0.027, 0.028]	0.014	21100	0.959
	SATE	-0.013	[-0.028, 0.002]	0.008	21100	0.090
H1: Affective polarization (W5)	PATE	-0.007	[-0.034, 0.021]	0.014	20408	0.634
	SATE	-0.016	[-0.033, -0.000]	0.008	20408	0.046
H4a: Consistency of cand. evals (W4)	PATE	0.009	[-0.038, 0.057]	0.024	21077	0.705
	SATE	-0.001	[-0.028, 0.027]	0.014	21077	0.965
H4a: Consistency of cand. evals (W5)	PATE	0.013	[-0.035, 0.061]	0.025	20380	0.594
	SATE	-0.007	[-0.035, 0.022]	0.015	20380	0.646
RQ4a: In-party affinity (W4)	PATE	-0.006	[-0.038, 0.026]	0.016	21143	0.725
	SATE	-0.032	[-0.050, -0.013]	0.009	21143	0.001
RQ4a: In-party affinity (W5)	PATE	-0.000	[-0.035, 0.034]	0.017	20430	0.986
	SATE	-0.027	[-0.047, -0.008]	0.010	20430	0.007
RQ4c: Out-party antipathy (W4)	PATE	-0.019	[-0.052, 0.015]	0.017	21107	0.272
	SATE	-0.013	[-0.032, 0.006]	0.010	21107	0.187
RQ4c: Out-party antipathy (W5)	PATE	0.005	[-0.029, 0.040]	0.017	20413	0.760
	SATE	-0.003	[-0.023, 0.017]	0.010	20413	0.737
RQ5a: Belief in false claims (W4)	PATE	0.002	[-0.038, 0.042]	0.021	21159	0.922
	SATE	0.010	[-0.014, 0.034]	0.012	21159	0.426
RQ5a: Belief in false claims (W5)	PATE	-0.022	[-0.062, 0.019]	0.021	20397	0.297
	SATE	-0.008	[-0.031, 0.015]	0.012	20397	0.483
RQ5b: Belief in pro-attitudinal false claims (W4)	PATE	0.024	[-0.020, 0.067]	0.022	21154	0.292
	SATE	-0.001	[-0.028, 0.026]	0.014	21154	0.960
RQ5b: Belief in pro-attitudinal false claims (W5)	PATE	-0.008	[-0.049, 0.033]	0.021	20397	0.686
	SATE	-0.014	[-0.039, 0.011]	0.013	20397	0.265
RQ5c: Accurate knowledge of current events (W4)	PATE	-0.013	[-0.052, 0.025]	0.020	21157	0.502
	SATE	-0.015	[-0.037, 0.008]	0.012	21157	0.203
RQ5c: Accurate knowledge of current events (W5)	PATE	0.022	[-0.018, 0.062]	0.020	20392	0.280
	SATE	0.003	[-0.021, 0.027]	0.012	20392	0.797
RQ5d: Accurate pro-attitudinal knowledge of events (W4)	PATE	-0.031	[-0.080, 0.017]	0.025	21151	0.202
	SATE	-0.012	[-0.039, 0.016]	0.014	21151	0.403
RQ5d: Accurate pro-attitudinal knowledge of events (W5)	PATE	-0.004	[-0.049, 0.041]	0.023	20378	0.869
	SATE	-0.004	[-0.030, 0.022]	0.013	20378	0.765
RQ6: Perceptions of democratic performance (W4)	PATE	-0.012	[-0.051, 0.026]	0.020	21159	0.531
	SATE	-0.002	[-0.025, 0.022]	0.012	21159	0.890
RQ6: Perceptions of democratic performance (W5)	PATE	-0.001	[-0.044, 0.043]	0.022	20400	0.970
	SATE	-0.010	[-0.035, 0.016]	0.013	20400	0.468

Estimates based on baseline OLS model (see Section S1.5.1). The last two columns report unadjusted and adjusted p-values from a two-sided t-test of the null hypothesis that each coefficient equals zero.

3.9 Treatment effects: Subgroup heterogeneity

Moderators

The preregistered list of primary moderators we test is listed below (we designate no secondary moderators). As discussed below, we deviate from our preregistration to treat the continuous/quasi-continuous variables as discrete because the software implementation of our estimation approach required discrete moderators in order to accommodate survey weights. We use indicators for quintiles for each moderator except as specified below.

- Political ideology (IDEO1)
  - Binary indicator for estimated ideology $\geq .5$
- Strength of political ideology (IDEO1)
  - Quintiles of ideology classifier score folded at .5 (i.e., the absolute value of the classifier value minus .5)
- Political sophistication
  - Sum of number of correct answers on political knowledge scale (KNOWHOUSE, KNOWSENATE) (0–2) and whether respondent correctly places Republicans running for office to the right of Democrats running for office (IDEOLOGY-GROUP) (0/1)
- Digital literacy
  - Three-point scale created from digital literacy scale (DIGLITERACY-TERM) where items (Viral, PDF, Selfie, Wiki, Hashtag, Emoji, Privacy settings) are measured on a five-point scale from 5=full understanding to 1=no understanding; 50% of the sample reported highest possible value so we include indicators for respondents in the 0–25th percentile, 25th–50th percentile, and 50th–100th percentile
- Prior levels/indicators of like-minded exposure during pre-treatment period (June 26–September 23, 2020)
  - Quintiles of proportion of views of content from like-minded sources
  - Quintiles of total views of content from like-minded sources
  - Quintiles of mean friends, Pages, and groups they follow that are classified as like-minded (average of proportions of friends, Pages, and groups)
- Prior levels of political content exposure during pre-treatment period (June 26–September 23, 2020)
  - Quintiles of proportion "civic" views (as measured by Facebook classifier)

3.9.1 Preregistered HTE estimation approach

The approach that we preregistered for estimating heterogeneous effects was as follows (https://osf.io/3sjy2). We first describe the preregistered approach and then document any deviations that were required.

We use the DR-Learner (*57–59*) to estimate effect heterogeneity across moderators specified in each individual pre-analysis plan. The DR-Learner is a nonparametric two-stage regression procedure that has been proven to be statistically optimal under weak conditions (*59*) and under essentially no conditions in an experiment. It reduces to the well-known semiparametric efficient augmented inverse-probability-weighted (AIPW) estimator in the case of discrete moderators (*60–64*).

In summary, the DR-Learner first constructs a doubly robust pseudo-outcome (*59, 65*) and then regresses this pseudo-outcome on moderators of interest; cross-fit sample splitting is used to prevent overfitting. There are thus three main pre-specification choices: how to estimate the pseudo-outcome, how to estimate the second-stage regression of the pseudo-outcome, and how to do sample splitting. The general approach is described in Kennedy (*59*); the definition of the pseudo-outcome is:

$$\hat{\varphi}(Z) = \frac{A - \hat{\pi}(X)}{\hat{\pi}(X)\{1 - \hat{\pi}(X)\}}\{Y - \hat{\mu}_A(X)\} + \hat{\mu}_1(X) - \hat{\mu}_0(X)$$

where X denotes covariates, A a binary treatment, Y the outcome of interest, $\widehat{\pi}(x)$ an estimate of the propensity score $P(A = 1 \mid X = x)$, and $\widehat{\mu}_a(x)$ a regression prediction of $E(Y \mid X = x, A = a)$. The pseudo-outcome mimics the difference in counterfactual outcomes $(Y^1 - Y^0)$ that would be observed under treatment versus control in the sense that the true pseudo-outcome $\varphi(Z)$ has conditional expectation exactly equal to the CATE: $\tau(x) = \mu_1(x) - \mu_0(x)$.

The doubly robust pseudo-outcome in the DR-Learner is built from two ingredients: propensity scores and regression predictions. In this experiment, the propensity scores are simply the known randomization probabilities. The regression predictions will be built using the superlearner package in R via a cross-validated ensemble of learners including linear regression, lasso with two-way interactions, random forests, boosting, and GAM.

Second stage DR-learner: "Effect models"
This second stage model will sometimes be referred to in this document as an "effect" model in contrast to the "outcome models" discussed above.

There are three different models we use:

1. **Effect model (1)**: Within-group averages will be used when the moderators are discrete and low-dimensional (five or fewer levels); this is equivalent to the classic AIPW estimator (*60–64*).

2. **Effect model (2)**: We will use local polynomial regression for single continuous moderators (*66*).

3. **Effect model (3)**: We will determine the most parsimonious, accurate model to use based on cross-validation using the one standard-error rule for multiple mixed moderators (*67*). Models considered will be linear regression, lasso with two-way interactions, GAMs, random forests, and boosted regression trees (listed in order of increasing complexity). Hyper-parameters will be tuned within each model class, then the simplest model within one standard-error of the best will be used (linear regression, lasso and GAMs will be ordered in complexity based on their number of non-zero parameters).

We will use 12-fold cross-validation for tuning parameter selection, both for regression predictions and second-stage pseudo-outcome regression, with 12-fold cross-fitting to separate building of regression predictions from the second-stage regression and second-stage model selection. This ensures efficient use of the data as each observation will separately contribute to regressions in both first and second stages.

Reporting of results

We will provide two types of estimates of effects of moderators:

- Marginal Moderation Effects (MCATE)
  - These correspond to the standard notion of a CATE (sometimes referred to as the Group Average Treatment Effect). In other words, it provides the average effect for all users with a particular value of a covariate.
  - If two potential moderators are strongly correlated, both will appear to have a strong marginal moderation effect.

  $$\text{MCATE}(X_k) = \int \tau(X)\, dP(X_{-k} \mid X_k)$$

  where X_{-k} denotes all covariates other than X_k so $dP(X_{-k} \mid X_k)$ denotes the conditional density of these covariates given X_k.

- Partial Moderation Effects (PCATE)
  - Under linearity, these correspond to the regression coefficients in a linear model predicting the treatment effect.
  - If two potential moderators are strongly correlated, one or both moderators could appear to have a strong marginal moderation effect, depending on which one best explains the observed heterogeneity.

  $$\text{PCATE}(X_k) = \int \tau(X)\, dP(X_{-k})$$

  This corresponds to the effects reported by the margins package in R, sometimes referred to as the "average marginal effect" or partial dependence (*68*). This sum will be computed using monte carlo.

Results on pre-specified moderators

- For all PCATEs, effect model (3) will be used (as described above in the section on the second stage DR-learner).
- For all MCATEs, the appropriate effect model will be chosen based on whether the hypothesis of interest is low-cardinality (model 1) or not (model 2).
- Plots will be generated for slices along the effect model in one dimension.

Results on unspecified moderators

- The first step will be to fit the effect model (3) as described above.
- We will then calculate feature importances. For all models, we will use the VIMP method for non-parametric estimation of variable importance (*69*). We will adjust p-values for these tests using a Benjamini-Hochberg correction as in the SOP, but which incorporates all accessible moderators (i.e., all available pre-treatment variables including ones not currently specified as moderators). This will not modify the corrections proposed for primary and secondary moderators in the "Adjustment for multiple comparisons" section.
- We will surface the features which reject the null of this test. We will plot slices (of the PCATE) of each of these features.
- Additional plots of slices along other moderators will be considered exploratory and non-definitive.
- These unspecified moderators may be the focus of a separate paper or not reported at all.

Hypothesis testing of moderation

- If individual research plans call for hypothesis tests against the null hypothesis that a variable is not a moderator, we will use the variable importance (defined above).
- Multiple comparisons corrections will be used according to the procedure detailed in the "Adjustment for multiple comparisons" section (i.e., with separate FDR control for primary and secondary moderators).

Model diagnostics

- Propensity scores are known, so no diagnostics are necessary.
- Outcome models:
  - We will examine common error metrics such as cross-validated mean squared-error (as primary error metric) along with other proper scoring rules.

- We will show the ensembling weights (and standard errors) for the SuperLearner model for each response surface.
- We will present plots of Regression ROC curves to demonstrate the performance of the regression more broadly (*70*).

- Effect models:
  - Cross-validated mean squared-error for each model considered
  - Regression ROC curves
  - For each effect model (3), we will plot the average predicted effects from the effect model against the difference-in-means effect:
    - Binned by the predicted effects from the effect model
    - Binned by the pre-specified low-dimensional moderators

3.9.2 HTE analysis approach: Deviations

Our preregistered approach to estimating heterogeneous treatment effects faced two implementation problems:

- The functions we use to compute the smoothed ATEs for continuous moderators do not accept survey weights.
- We wished to avoid the problem of false positives in conditional average treatment effect calculations (*71*). However, our approach to implementing our multiple comparison correction was to adjust the significance of every treatment effect estimate at different values of the moderator, which is likely too conservative.

We therefore deviate from our preregistration to treat our continuous moderators as discrete, converting them into quintiles where possible.[4] This change allows us to implement weights and a more conventional approach to the multiple comparisons correction. Our HTE estimation approach is thus the one described in model (1) above, which is equivalent to the augmented inverse-probability-weighted (AIPW) estimator.

We also deviate from our preregistered approach to analyzing variable importance (VIMP) because the fully non-parametric formulations we specified in the pre-analysis plan entail unacceptably large amounts of variance. We therefore instead use the "linear" measure of VIMP, which simply tests the difference in mean squared error between a model incorporating all covariates and one that leaves out a particular moderator. In contrast to the non-parametric version, this approach requires assuming that the linear model is the correct specification of the CATE.

[4]Approximately half of our sample reported the highest possible value on the digital literacy scale, so the sample was instead split into three parts on that measure: the 0–25th percentile, 25th–50th percentile, and the $> 50^{\text{th}}$ percentile. As preregistered, our ideology measure is split into liberal and conservative and the political sophistication measure is split by the number of correct answers from 0–3.

Finally, to economize on space, the preregistered HTE diagnostics listed below are not reported in this appendix but will instead be made available as part of the public replication materials we make available when this study is published:

- Outcome models:
  - We will examine common error metrics such as cross-validated mean squared-error (as primary error metric) along with other proper scoring rules.
  - We will present plots of Regression ROC curves to demonstrate the performance of the regression more broadly (*70*).
- Effect models:
  - Cross-validated mean squared-error for each model considered
  - Regression ROC curves
  - For each effect model (3), we will plot the average predicted effects from the effect model against the difference-in-means effect:
    * Binned by the predicted effects from the effect model
    * Binned by the pre-specified low-dimensional moderators

3.9.3 HTE analysis for primary hypotheses

Figures 12–27 visualize the results of the HTE analysis for our primary hypotheses. We first report the ensemble weights used to create regression predictions for each dependent variable. We then depict the estimated HTE results for that dependent variable for each of eight preregistered moderators: political ideology, strength of ideology, political sophistication, digital literacy, percent like-minded views, total like-minded views, percent like-minded network, and percent civic views. A clear pattern of null results is evident. All figures plot MCATE estimates by quintile. Graphs report two-tailed t-statistic for the test of whether the subgroup treatment effect is different from zero (adjusted using the approach described in Section 1.5.4).

Figure 12: Ensemble weights for model of heterogeneous treatment effects of reducing exposure to like-minded sources on affective polarization

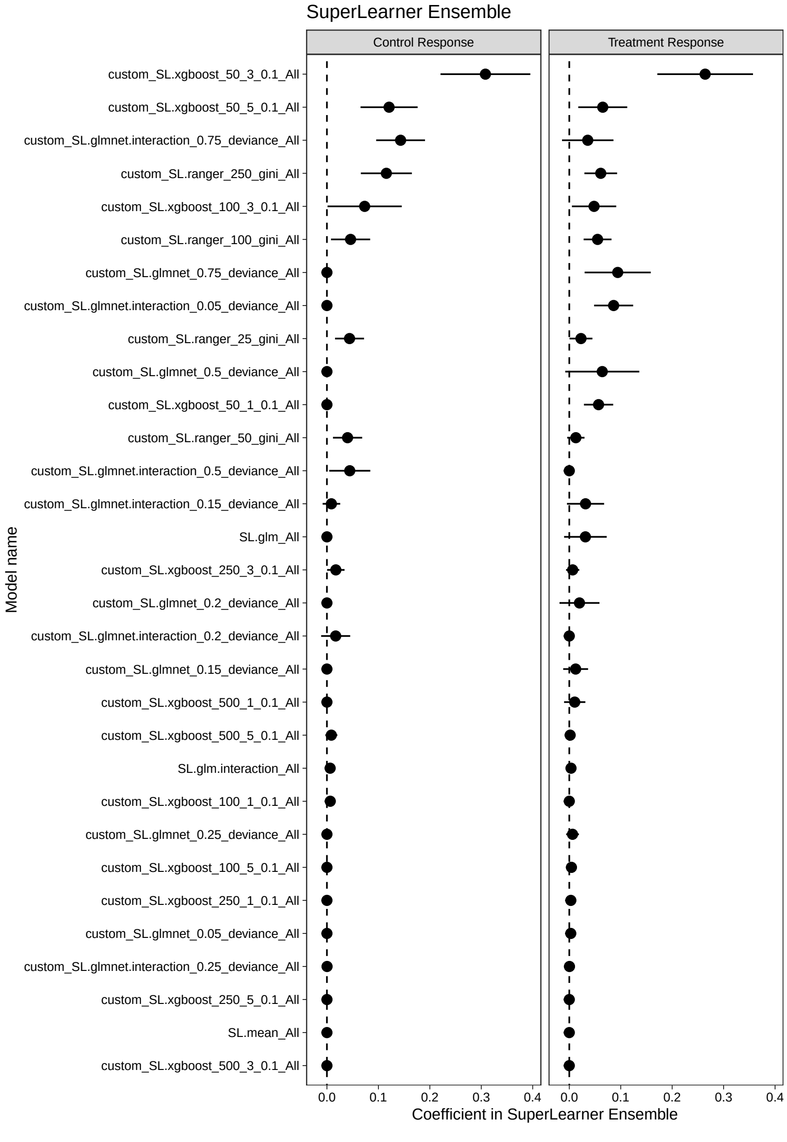

Ensemble weights (including 95% confidence intervals) estimated using the DR-Learner approach (*57–59*) for models testing for heterogeneous treatment effects of reducing exposure to like-minded sources in the Facebook Feed from September 24–December 23, 2020 on affective polarization across several preregistered moderators. N = 22,127.

Figure 13: Heterogeneous treatment effects of reducing exposure to like-minded sources on affective polarization

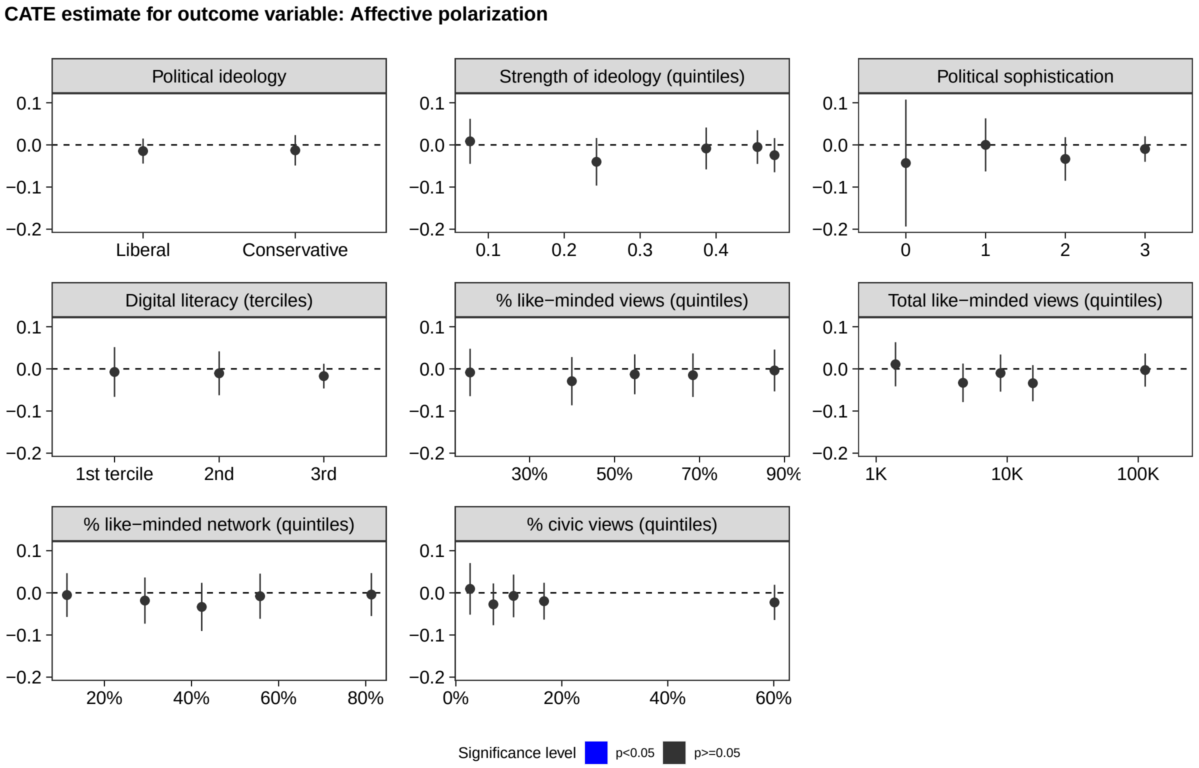

Conditional average treatment effects (including 95% confidence intervals) of reducing exposure to like-minded sources in the Facebook Feed from September 24–December 23, 2020 on affective polarization across several preregistered moderators. Estimates were constructed using the DR-Learner approach (*57–59*). Tests of significance consider the two-tailed t-statistic for the test of whether the subgroup treatment effect is different from zero after controlling the false discovery rate (*55*). Political ideology and sophistication were treated as discrete variables; other moderators were treated as continuous. Survey outcome measures are standardized scales averaged across surveys conducted November 4–18, 2020 and/or December 9–23, 2020. N = 22,127. After adjusting for multiple comparisons, all p-values equal 1.

Figure 14: Ensemble weights for model of heterogeneous treatment effects of reducing exposure to like-minded sources on ideological extremity

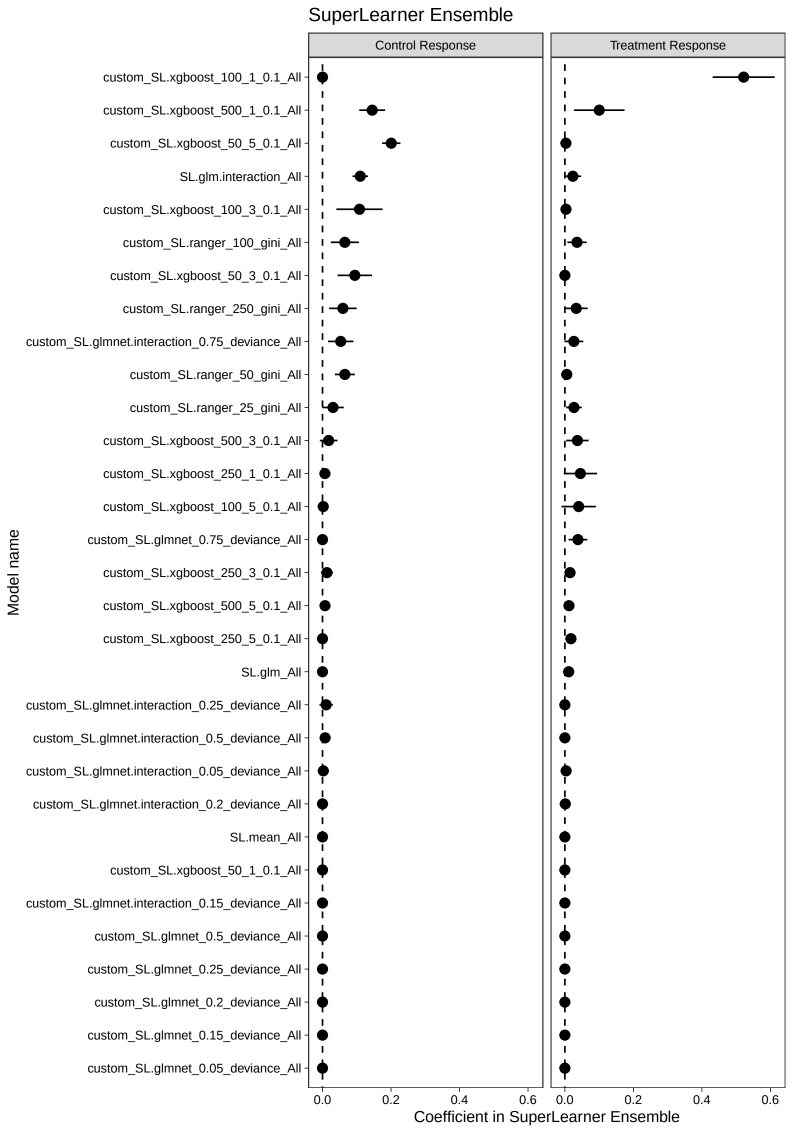

Ensemble weights (including 95% confidence intervals) estimated using the DR-Learner approach (*57–59*) for models testing for heterogeneous treatment effects of reducing exposure to like-minded sources in the Facebook Feed from September 24–December 23, 2020 on ideological extremity across several preregistered moderators. N = 22,154.

Figure 15: Heterogeneous treatment effects of reducing exposure to like-minded sources on ideological extremity

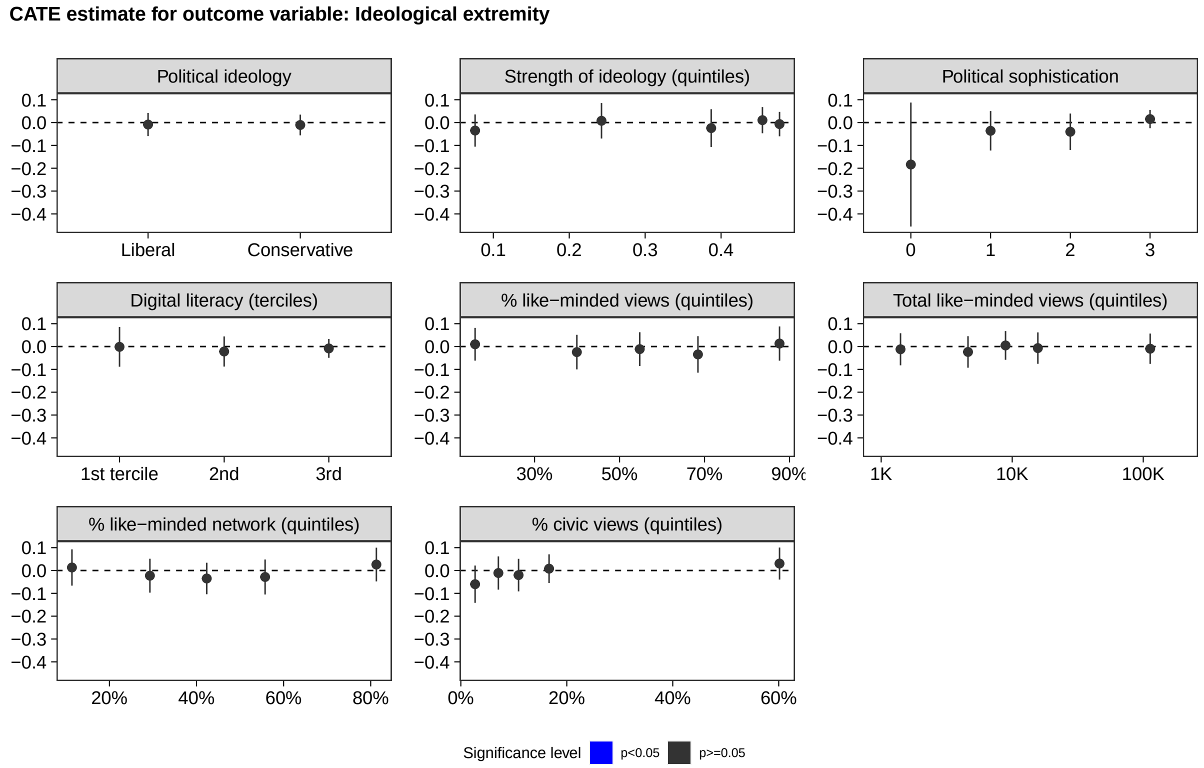

Conditional average treatment effects (including 95% confidence intervals) of reducing exposure to like-minded sources in the Facebook Feed from September 24–December 23, 2020 on ideological extremity across several preregistered moderators. Estimates were constructed using the DR-Learner approach (*57–59*). Tests of significance consider the two-tailed t-statistic for the test of whether the subgroup treatment effect is different from zero after controlling the false discovery rate (*55*). Political ideology and sophistication were treated as discrete variables; other moderators were treated as continuous. Survey outcome measures are standardized scales averaged across surveys conducted November 4–18, 2020 and/or December 9–23, 2020. N = 22,154. After adjusting for multiple comparisons, all p-values equal 1.

Figure 16: Ensemble weights for model of heterogeneous treatment effects of reducing exposure to like-minded sources on policy consistency

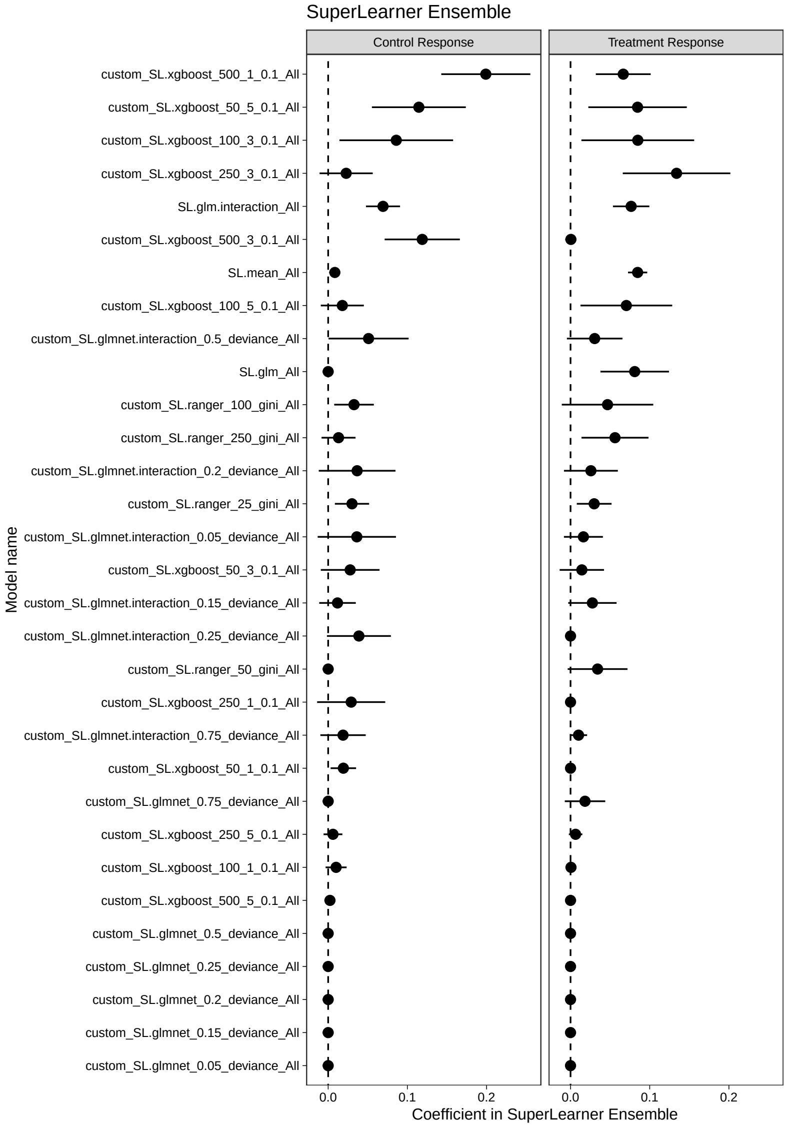

Ensemble weights (including 95% confidence intervals) estimated using the DR-Learner approach (*57–59*) for models testing for heterogeneous treatment effects of reducing exposure to like-minded sources in the Facebook Feed from September 24–December 23, 2020 on policy consistency across several preregistered moderators. N = 22,159.

Figure 17: Heterogeneous treatment effects of reducing exposure to like-minded sources on policy consistency

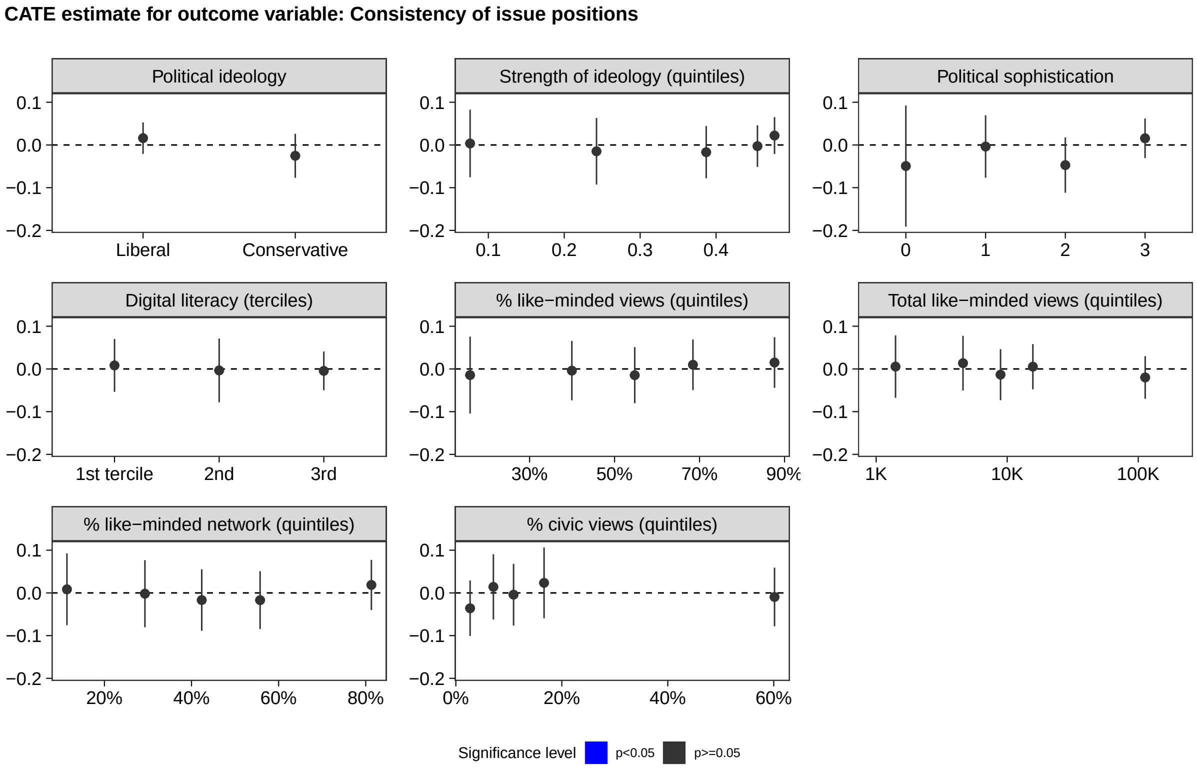

Conditional average treatment effects (including 95% confidence intervals) of reducing exposure to like-minded sources in the Facebook Feed from September 24–December 23, 2020 on policy consistency across several preregistered moderators. Estimates were constructed using the DR-Learner approach (*57–59*). Tests of significance consider the two-tailed t-statistic for the test of whether the subgroup treatment effect is different from zero after controlling the false discovery rate (*55*). Political ideology and sophistication were treated as discrete variables; other moderators were treated as continuous. Survey outcome measures are standardized scales averaged across surveys conducted November 4–18, 2020 and/or December 9–23, 2020. N $=$ 22,159. After adjusting for multiple comparisons, all p-values equal 1.

Figure 18: Ensemble weights for model of heterogeneous treatment effects of reducing exposure to like-minded sources on racial attitudes

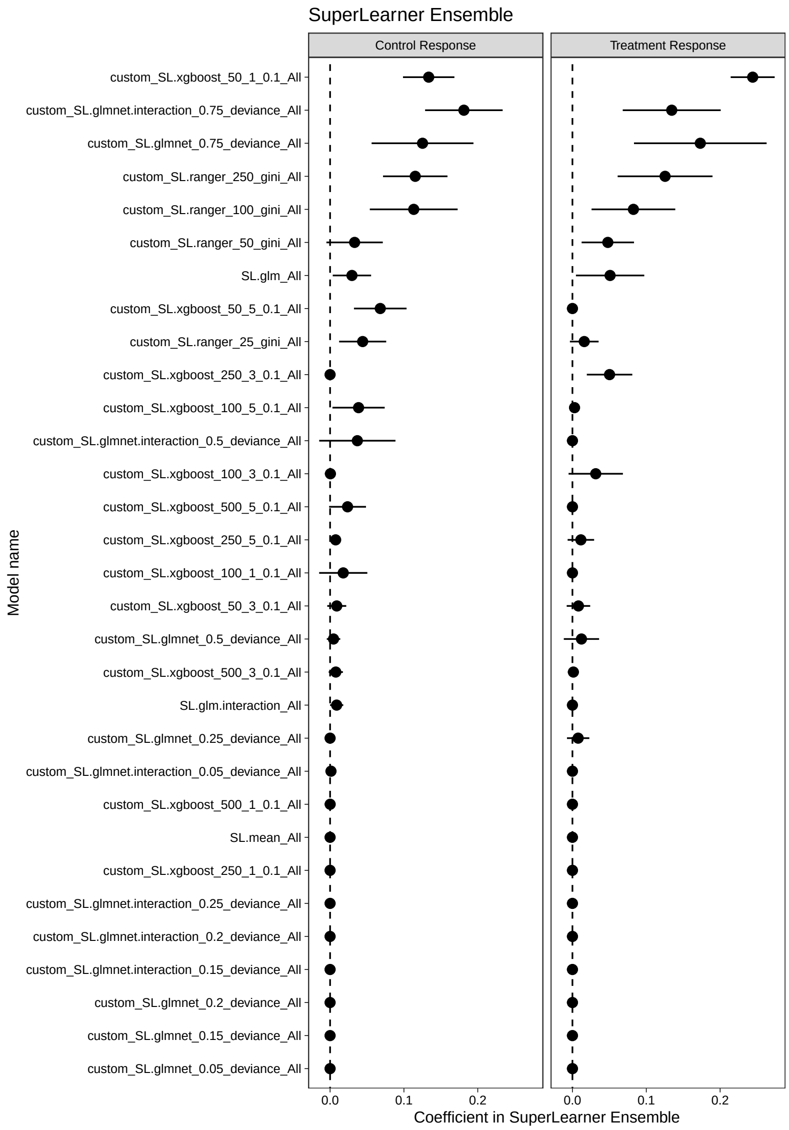

Ensemble weights (including 95% confidence intervals) estimated using the DR-Learner approach (*57–59*) for models testing for heterogeneous treatment effects of reducing exposure to like-minded sources in the Facebook Feed from September 24–December 23, 2020 on racial attitudes across several preregistered moderators. N = 22,162.

Figure 19: Heterogeneous treatment effects of reducing exposure to like-minded sources on racial attitudes

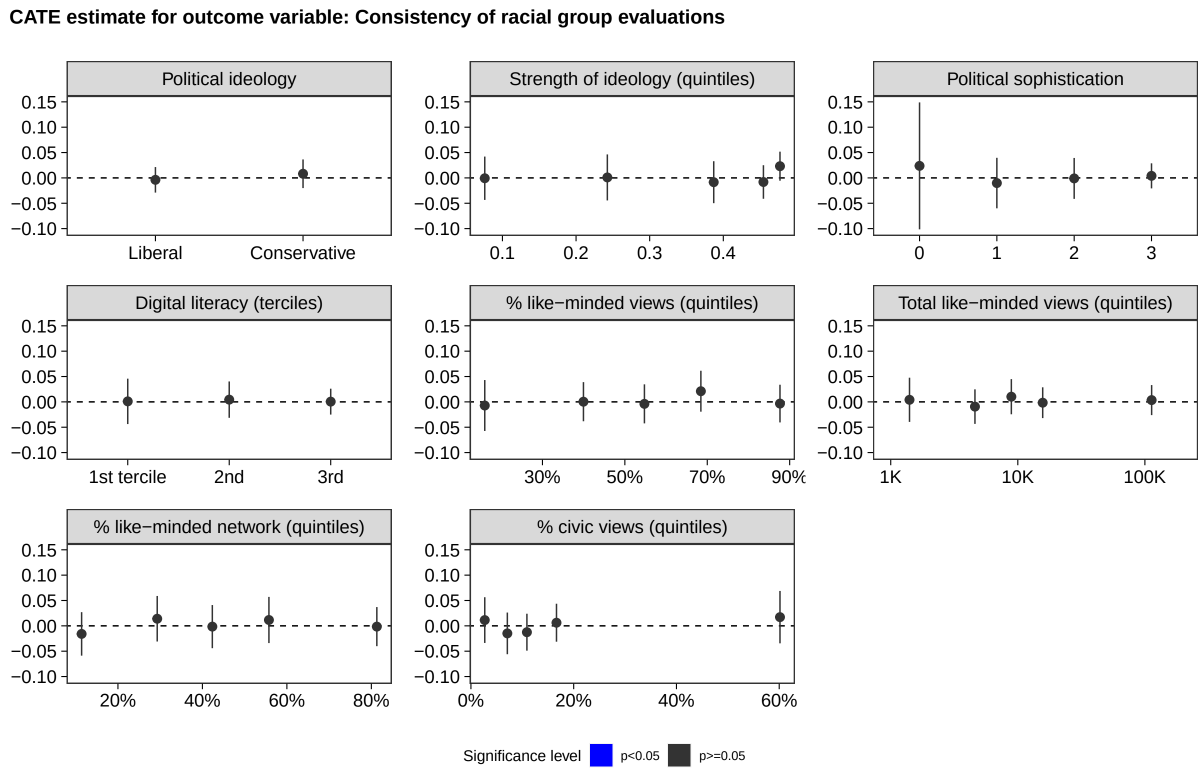

Conditional average treatment effects (including 95% confidence intervals) of reducing exposure to like-minded sources in the Facebook Feed from September 24–December 23, 2020 on racial attitudes across several preregistered moderators. Estimates were constructed using the DR-Learner approach (*57–59*). Tests of significance consider the two-tailed t-statistic for the test of whether the subgroup treatment effect is different from zero after controlling the false discovery rate (*55*). Political ideology and sophistication were treated as discrete variables; other moderators were treated as continuous. Survey outcome measures are standardized scales averaged across surveys conducted November 4–18, 2020 and/or December 9–23, 2020. N $=$ 22,162. After adjusting for multiple comparisons, all p-values equal 1.

Figure 20: Ensemble weights for model of heterogeneous treatment effects of reducing exposure to like-minded sources on consistency of vote choice and candidate evaluations

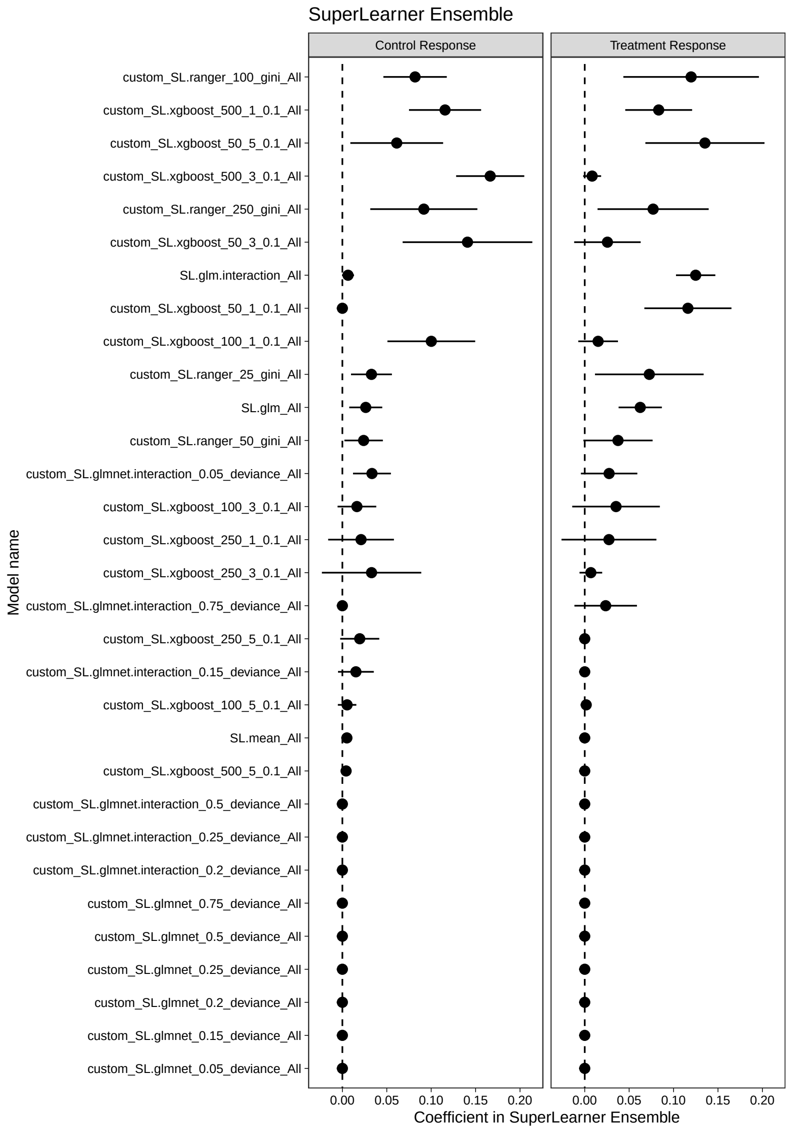

Ensemble weights (including 95% confidence intervals) estimated using the DR-Learner approach (*57–59*) for models testing for heterogeneous treatment effects of reducing exposure to like-minded sources in the Facebook Feed from September 24–December 23, 2020 on consistency of vote choice and candidate evaluations across several preregistered moderators. N = 22158.

Figure 21: Heterogeneous treatment effects of reducing exposure to like-minded sources on vote choice and candidate evaluations

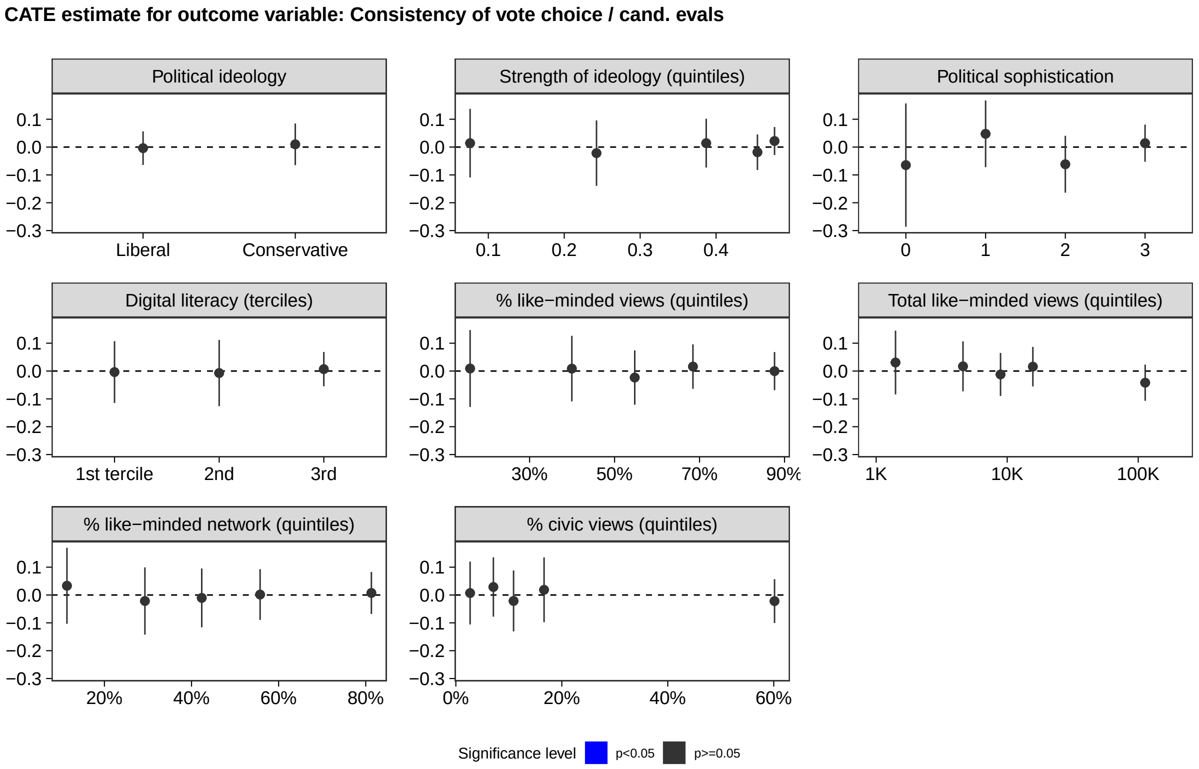

Conditional average treatment effects (including 95% confidence intervals) of reducing exposure to like-minded sources in the Facebook Feed from September 24–December 23, 2020 on vote choice and candidate evaluations across several preregistered moderators. Estimates were constructed using the DR-Learner approach (*57–59*). Tests of significance consider the two-tailed t-statistic for the test of whether the subgroup treatment effect is different from zero after controlling the false discovery rate (*55*). Political ideology and sophistication were treated as discrete variables; other moderators were treated as continuous. Survey outcome measures are standardized scales averaged across surveys conducted November 4–18, 2020 and/or December 9–23, 2020. N = 21,158. After adjusting for multiple comparisons, all p-values equal 1.

Figure 22: Ensemble weights for model of heterogeneous treatment effects of reducing exposure to like-minded sources on party-congenial beliefs

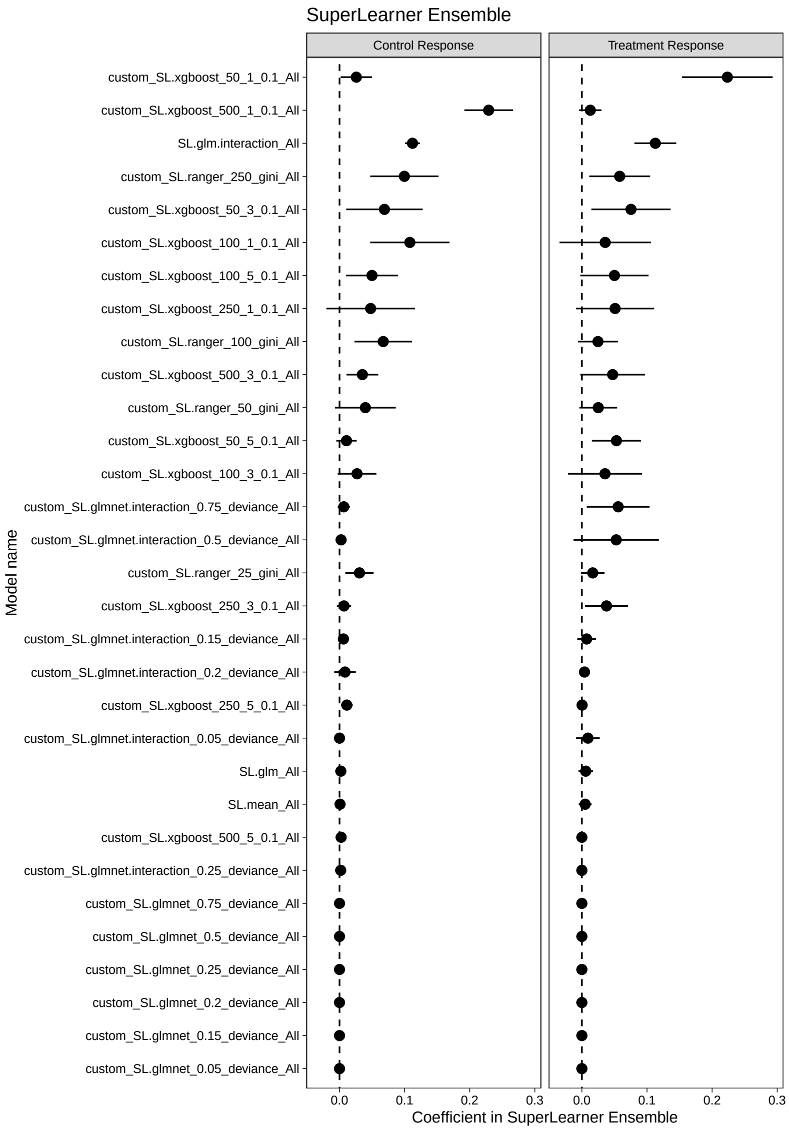

Ensemble weights (including 95% confidence intervals) estimated using the DR-Learner approach (*57–59*) for models testing for heterogeneous treatment effects of reducing exposure to like-minded sources in the Facebook Feed from September 24–December 23, 2020 on party-congenial beliefs across several preregistered moderators. N = 20,442.

Figure 23: Heterogeneous treatment effects of reducing exposure to like-minded sources on party-congenial beliefs

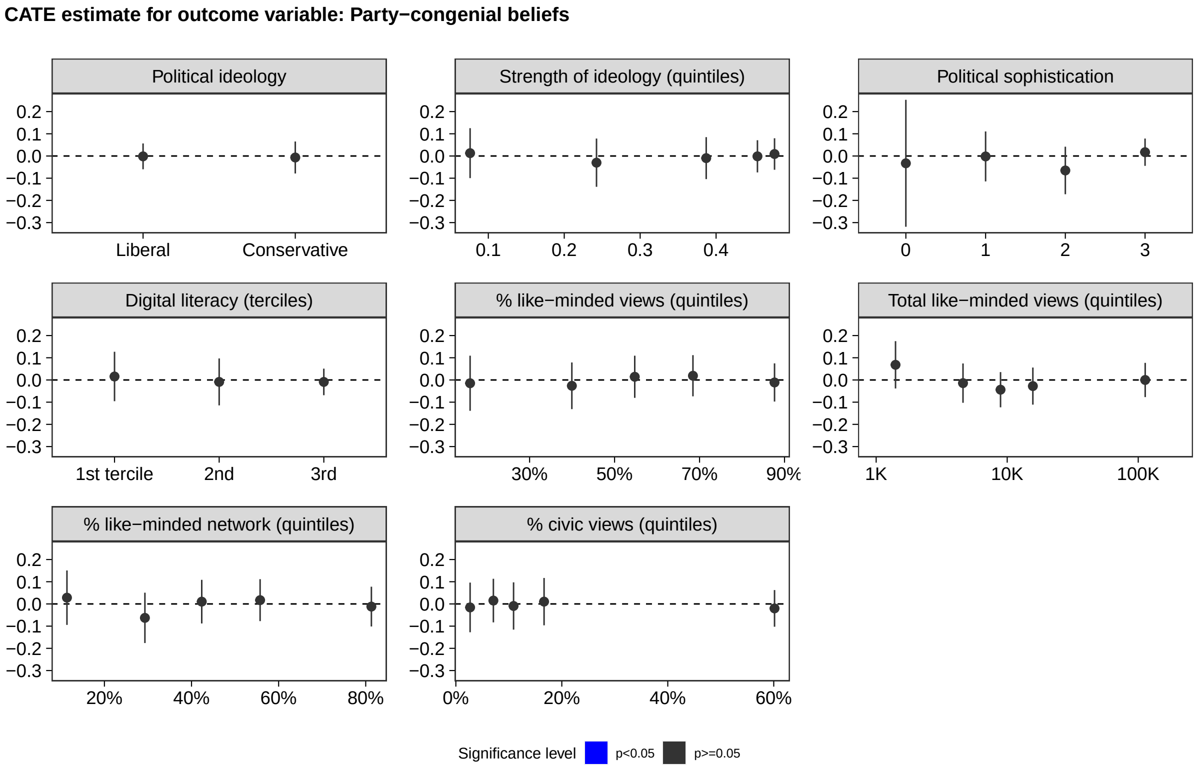

Conditional average treatment effects (including 95% confidence intervals) of reducing exposure to like-minded sources in the Facebook Feed from September 24–December 23, 2020 on party-congenial beliefs across several preregistered moderators. Estimates were constructed using the DR-Learner approach (*57–59*). Tests of significance consider the two-tailed t-statistic for the test of whether the subgroup treatment effect is different from zero after controlling the false discovery rate (*55*). Political ideology and sophistication were treated as discrete variables; other moderators were treated as continuous. Survey outcome measures are standardized scales averaged across surveys conducted November 4–18, 2020 and/or December 9–23, 2020. N $=$ 20,442. After adjusting for multiple comparisons, all p-values equal 1.

Figure 24: Ensemble weights for model of heterogeneous treatment effects of reducing exposure to like-minded sources on election confidence

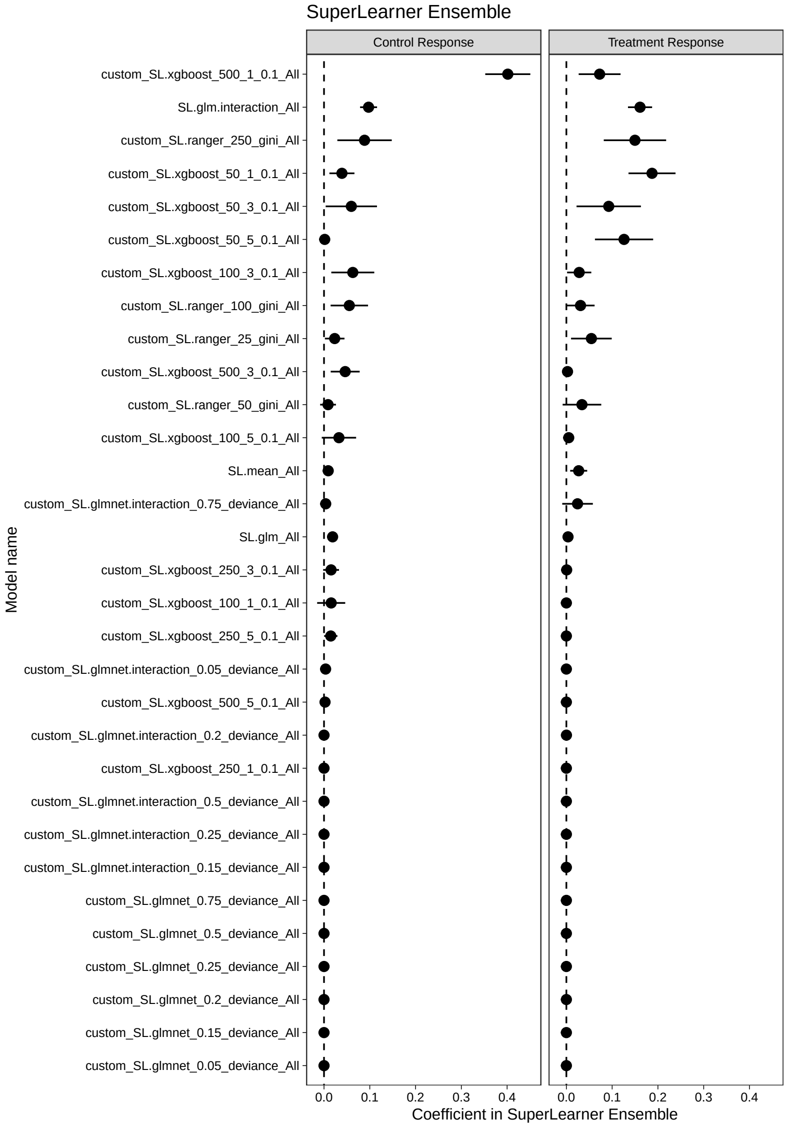

Ensemble weights (including 95% confidence intervals) estimated using the DR-Learner approach (*57–59*) for models testing for heterogeneous treatment effects of reducing exposure to like-minded sources in the Facebook Feed from September 24–December 23, 2020 on election confidence across several preregistered moderators. N = 20,442.

Figure 25: Heterogeneous treatment effects of reducing exposure to like-minded sources on election confidence

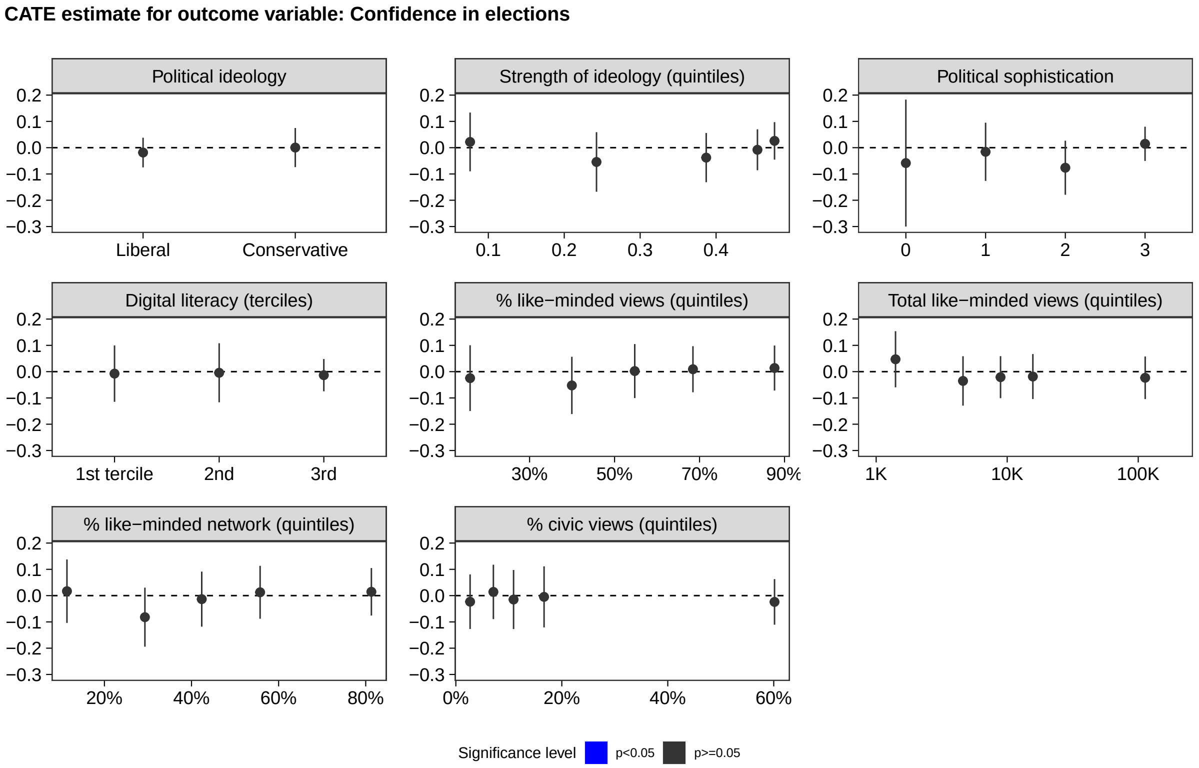

Conditional average treatment effects (including 95% confidence intervals) of reducing exposure to like-minded sources in the Facebook Feed from September 24–December 23, 2020 on electionconfidence across several preregistered moderators. Estimates were constructed using the DR-Learner approach (*57–59*). Tests of significance consider the two-tailed t-statistic for the test of whether the subgroup treatment effect is different from zero after controlling the false discovery rate (*55*). Political ideology and sophistication were treated as discrete variables; other moderators were treated as continuous. Survey outcome measures are standardized scales averaged across surveys conducted November 4–18, 2020 and/or December 9–23, 2020. N = 20,442. After adjusting for multiple comparisons, all p-values equal 1.

Figure 26: Ensemble weights for model of heterogeneous treatment effects of reducing exposure to like-minded sources on respect for election norms

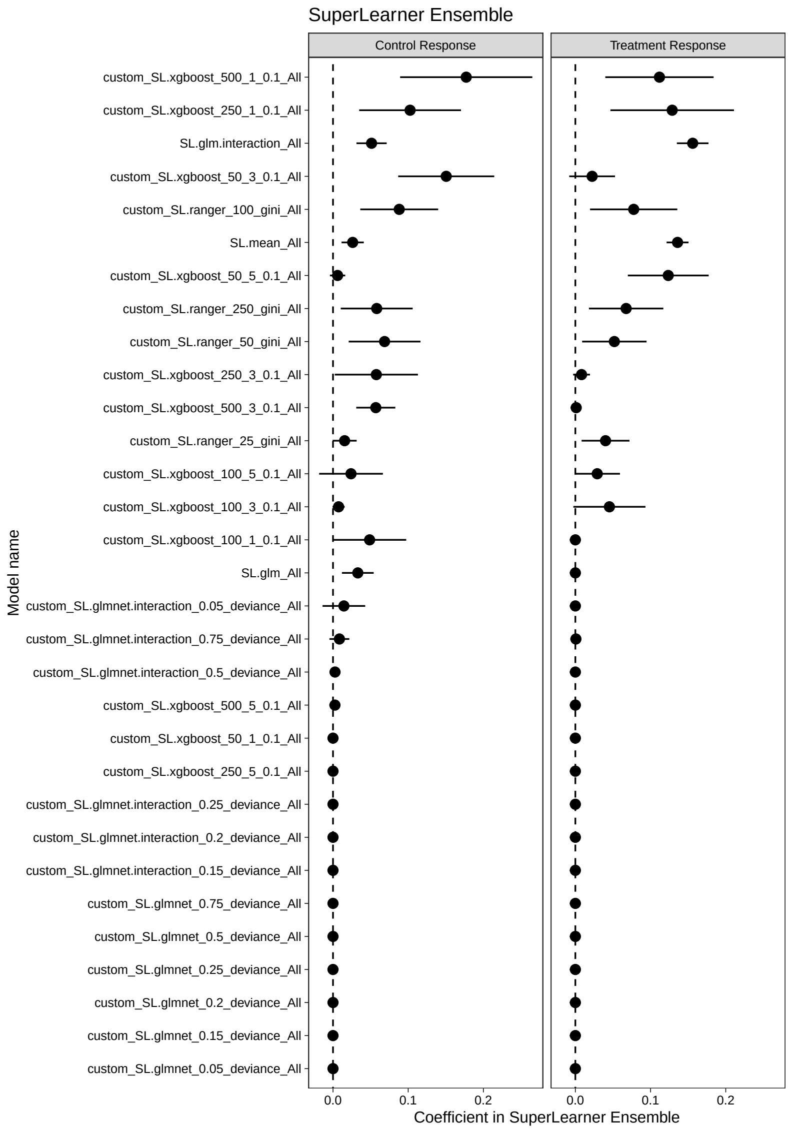

Ensemble weights (including 95% confidence intervals) estimated using the DR-Learner approach (*57–59*) for models testing for heterogeneous treatment effects of reducing exposure to like-minded sources in the Facebook Feed from September 24–December 23, 2020 on respect for election norms across several preregistered moderators. N = 20,390.

Figure 27: Heterogeneous treatment effects of reducing exposure to like-minded sources on respect for election norms

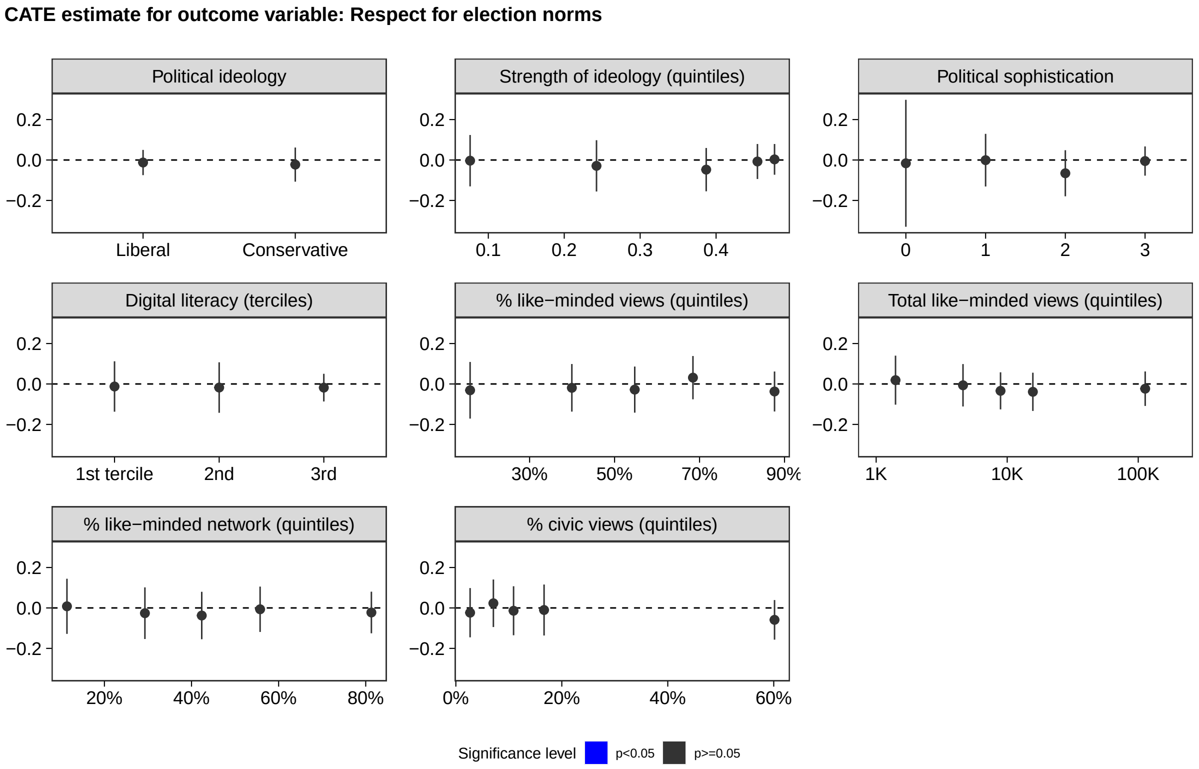

Conditional average treatment effects (including 95% confidence intervals) of reducing exposure to like-minded sources in the Facebook Feed from September 24–December 23, 2020 on respect for election norms across several preregistered moderators. Estimates were constructed using the DR-Learner approach (*57–59*). Tests of significance consider the two-tailed t-statistic for the test of whether the subgroup treatment effect is different from zero after controlling the false discovery rate (*55*). Political ideology and sophistication were treated as discrete variables; other moderators were treated as continuous. Survey outcome measures are standardized scales averaged across surveys conducted November 4–18, 2020 and/or December 9–23, 2020. N $=$ 20,390. After adjusting for multiple comparisons, all p-values equal 1.

3.9.4 HTE analysis for research questions

Figures 28–61 present the results of the HTE analysis for our research questions. We first report the ensemble weights used to create regression predictions for each dependent variable. We then depict the estimated HTE results for that dependent variable for each of eight pre-registered moderators: political ideology, strength of ideology, political sophistication, digital literacy, percent like-minded views, total like-minded views, percent like-minded network, and percent civic views. A clear pattern of null results is evident for all dependent variables except on-platform political participation. All figures plot MCATE estimates by quintile. Graphs report two-tailed t-statistic for the test of whether the subgroup treatment effect is different from zero (adjusted using the approach described in Section 1.5.4).

Figure 28: Ensemble weights for model of heterogeneous treatment effects of reducing exposure to like-minded sources on self-reported turnout

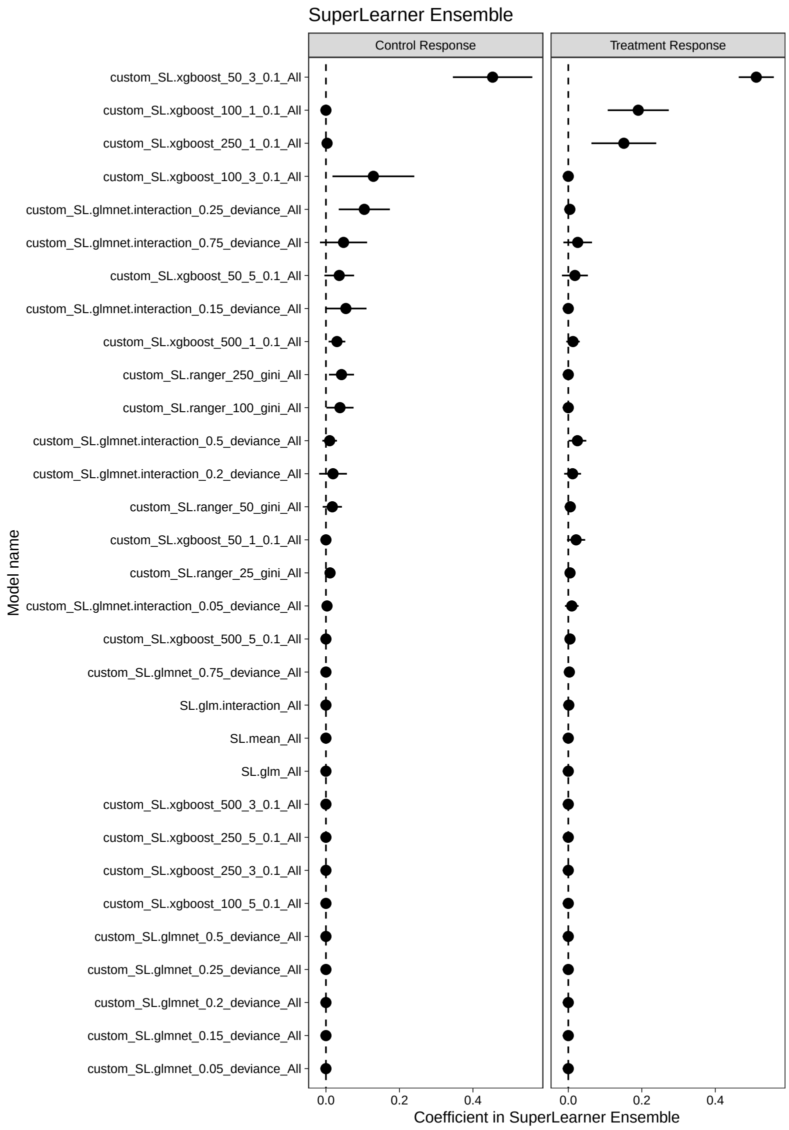

Ensemble weights (including 95% confidence intervals) estimated using the DR-Learner approach (*57–59*) for models testing for heterogeneous treatment effects of reducing exposure to like-minded sources in the Facebook Feed from September 24–December 23, 2020 on self-reported turnout across several preregistered moderators. N = 22,139.

Figure 29: Heterogeneous treatment effects of reducing exposure to like-minded sources on self-reported turnout

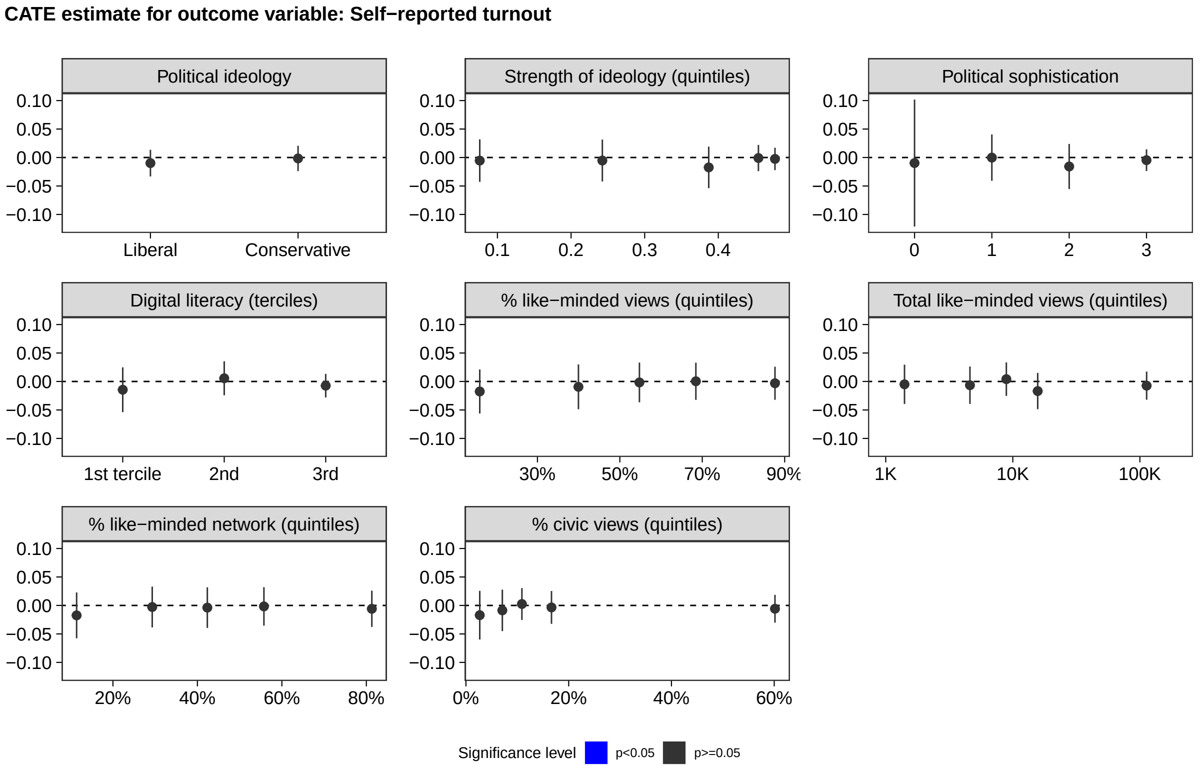

Conditional average treatment effects (including 95% confidence intervals) of reducing exposure to like-minded sources in the Facebook Feed from September 24–December 23, 2020 on self-reported turnout across several preregistered moderators. Estimates were constructed using the DR-Learner approach (*57–59*). Tests of significance consider the two-tailed t-statistic for the test of whether the subgroup treatment effect is different from zero after controlling the false discovery rate (*55*). Political ideology and sophistication were treated as discrete variables; other moderators were treated as continuous. Survey outcome measures are standardized scales averaged across surveys conducted November 4–18, 2020 and/or December 9–23, 2020. N = 22,139. After adjusting for multiple comparisons, all p-values equal 1.

Figure 30: Ensemble weights for model of heterogeneous treatment effects of reducing exposure to like-minded sources on self-reported participation

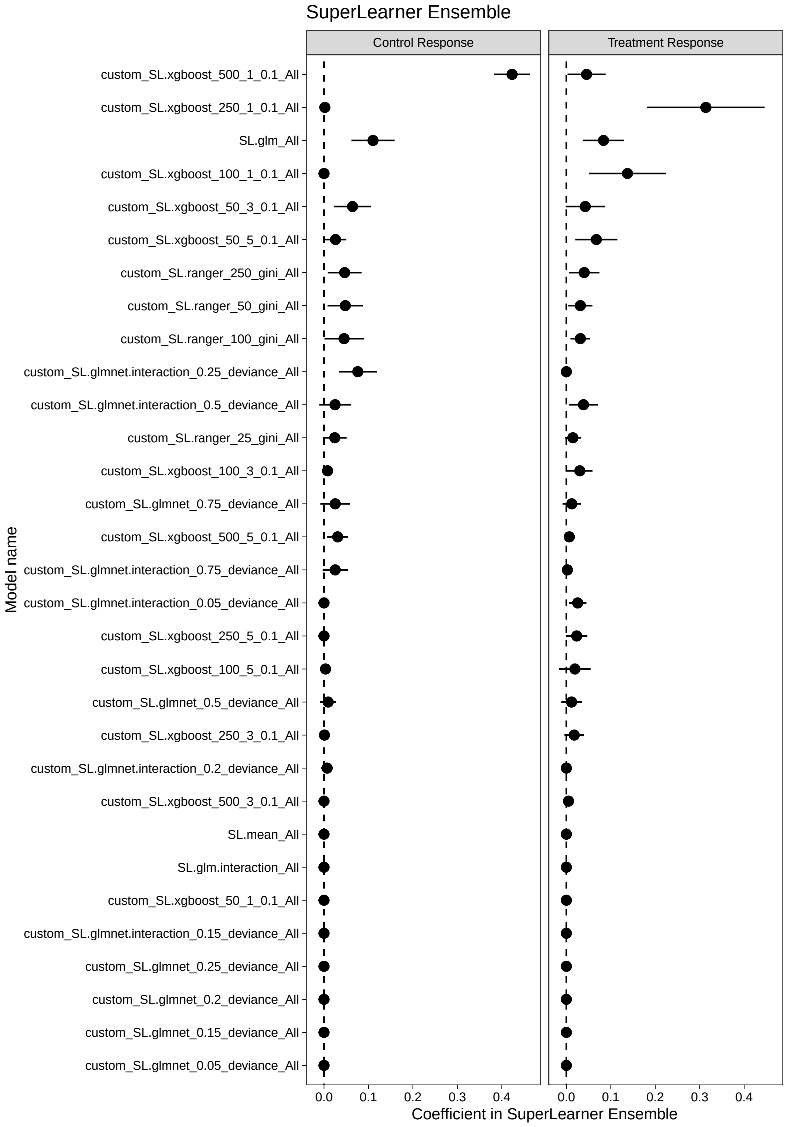

Ensemble weights (including 95% confidence intervals) estimated using the DR-Learner approach (*57*–*59*) for models testing for heterogeneous treatment effects of reducing exposure to like-minded sources in the Facebook Feed from September 24–December 23, 2020 on self-reported participation across several preregistered moderators. N = 22,162

Figure 31: Heterogeneous treatment effects of reducing exposure to like-minded sources on self-reported participation

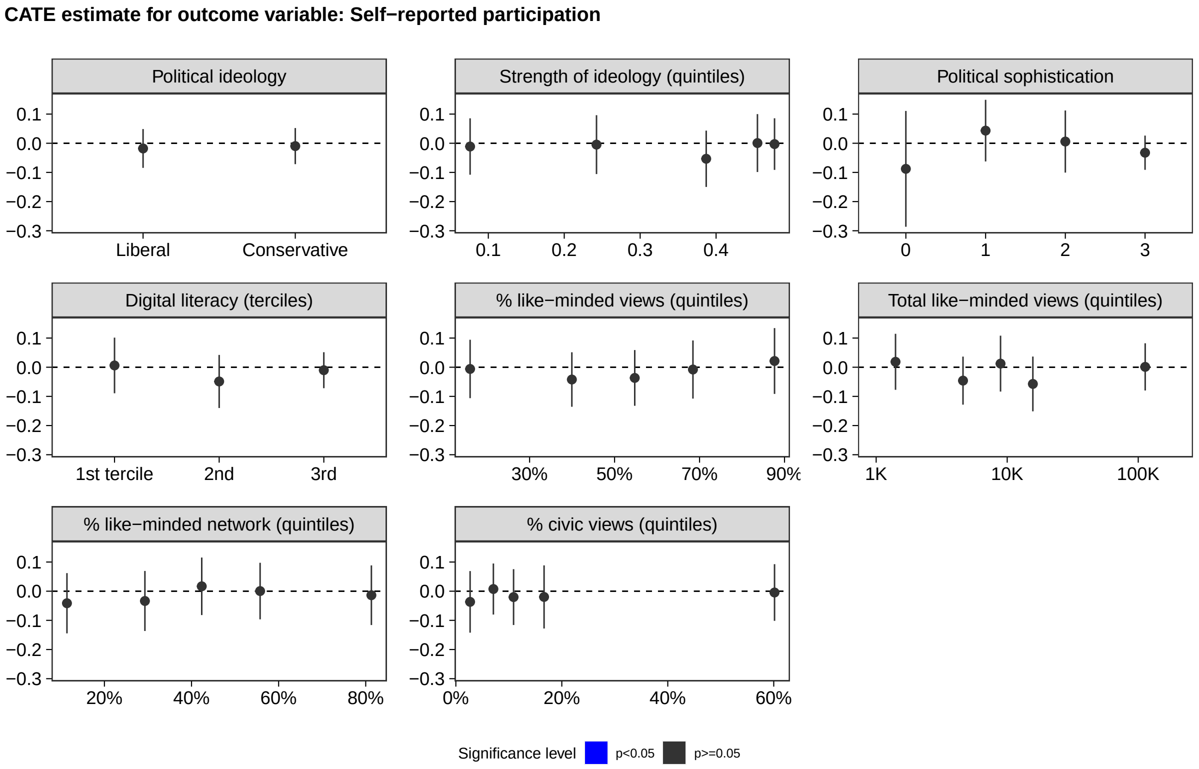

Conditional average treatment effects (including 95% confidence intervals) of reducing exposure to like-minded sources in the Facebook Feed from September 24–December 23, 2020 on self-reported participation across several preregistered moderators. Estimates were constructed using the DR-Learner approach (*57–59*). Tests of significance consider the two-tailed t-statistic for the test of whether the subgroup treatment effect is different from zero after controlling the false discovery rate (*55*). Political ideology and sophistication were treated as discrete variables; other moderators were treated as continuous. Survey outcome measures are standardized scales averaged across surveys conducted November 4–18, 2020 and/or December 9–23, 2020. N = 22,162. After adjusting for multiple comparisons, all p-values equal 1.

Figure 32: Ensemble weights for model of heterogeneous treatment effects of reducing exposure to like-minded sources on political participation on Facebook

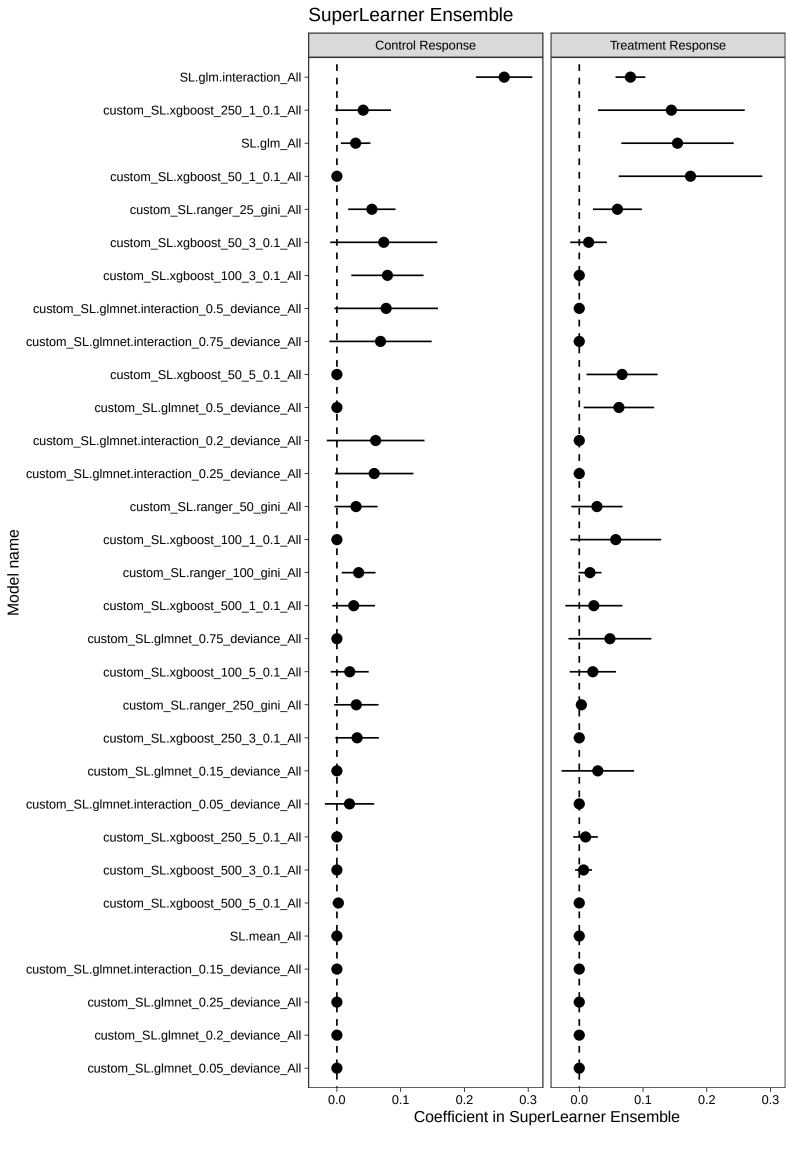

Ensemble weights (including 95% confidence intervals) estimated using the DR-Learner approach (*57–59*) for models testing for heterogeneous treatment effects of reducing exposure to like-minded sources in the Facebook Feed from September 24–December 23, 2020 on political participation on Facebook across several preregistered moderators. N = 22,143.

Figure 33: Heterogeneous treatment effects of reducing exposure to like-minded sources on political participation on Facebook

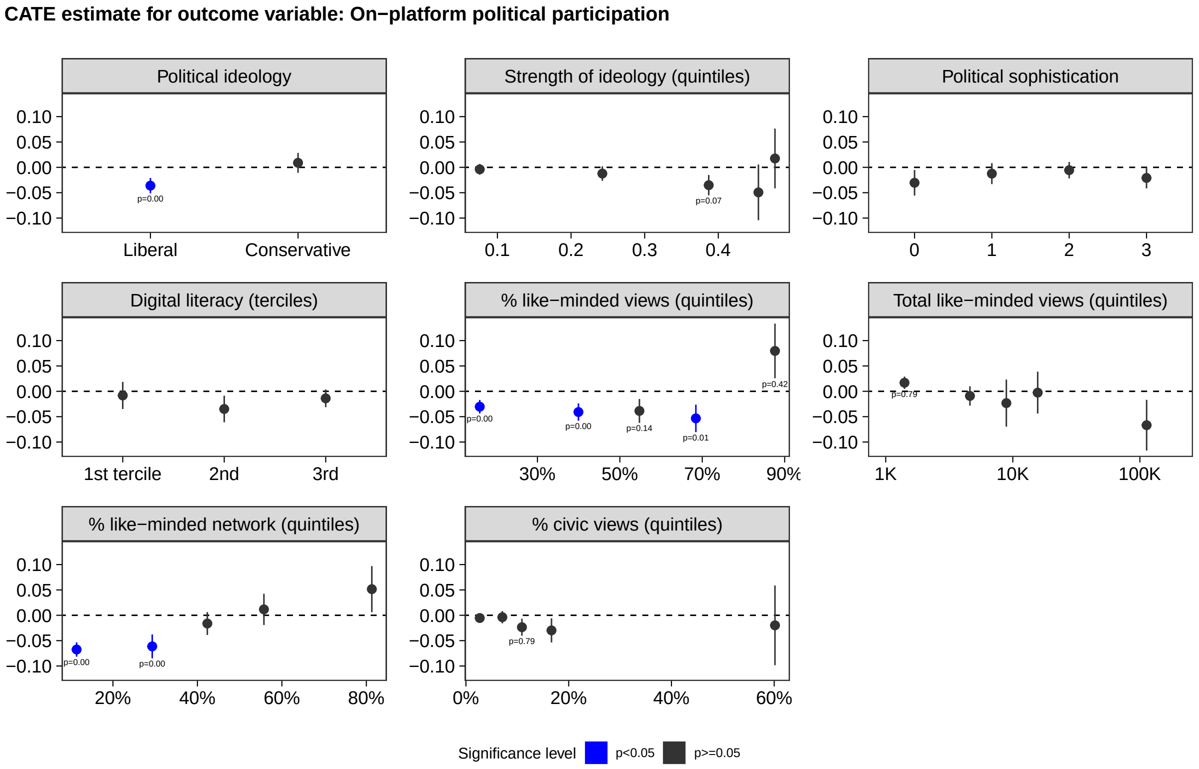

Conditional average treatment effects (including 95% confidence intervals) of reducing exposure to like-minded sources in the Facebook Feed from September 24–December 23, 2020 on political participation on Facebook across several preregistered moderators. Estimates were constructed using the DR-Learner approach (*57–59*). Tests of significance consider the two-tailed t-statistic for the test of whether the subgroup treatment effect is different from zero after controlling the false discovery rate (*55*). Political ideology and sophistication were treated as discrete variables; other moderators were treated as continuous. Survey outcome measures are standardized scales averaged across surveys conducted November 4–18, 2020 and/or December 9–23, 2020. N $=$ 22,143. Unless otherwise reported in the figure, all p-values equal 1 after adjusting for multiple comparisons.

Figure 34: Ensemble weights for model of heterogeneous treatment effects of reducing exposure to like-minded sources on perceived percentage of Facebook friends who are like-minded

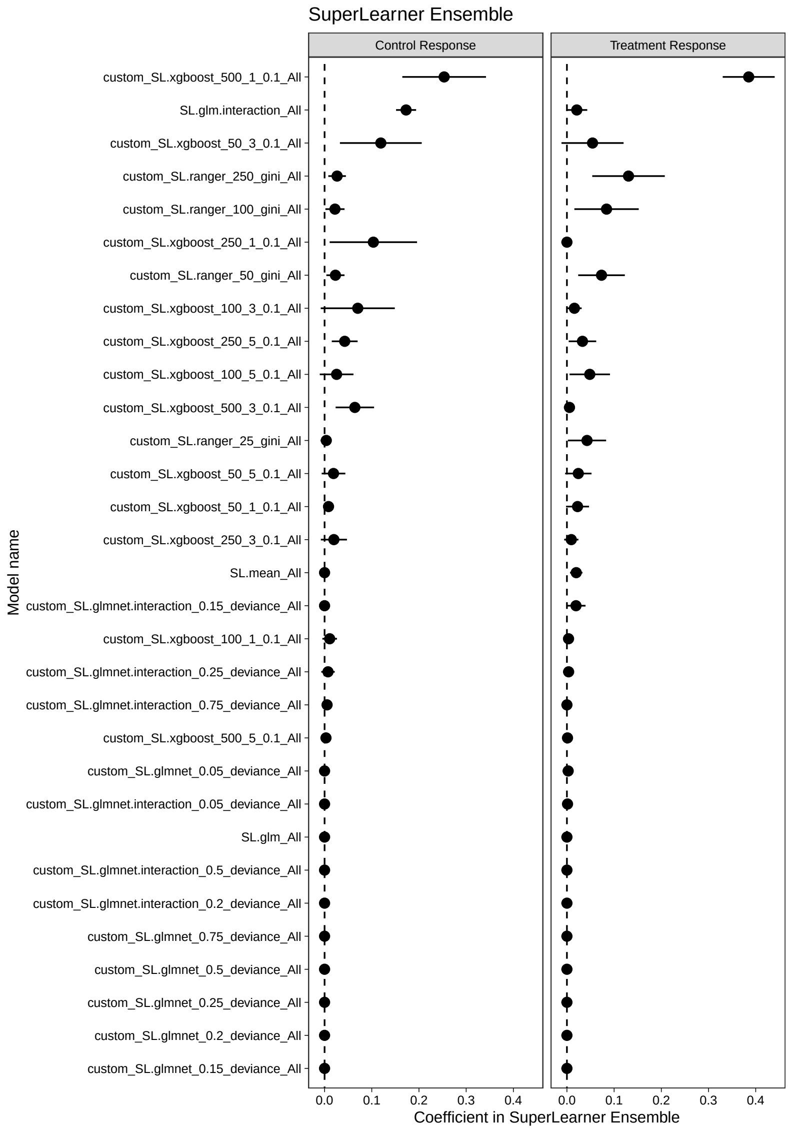

Ensemble weights (including 95% confidence intervals) estimated using the DR-Learner approach (*57*–*59*) for models testing for heterogeneous treatment effects of reducing exposure to like-minded sources in the Facebook Feed from September 24–December 23, 2020 on perceived percentage of Facebook friends who are like-minded across several preregistered moderators. N = 21,071.

Figure 35: Heterogeneous treatment effects of reducing exposure to like-minded sources on perceived percentage of Facebook friends who are like-minded

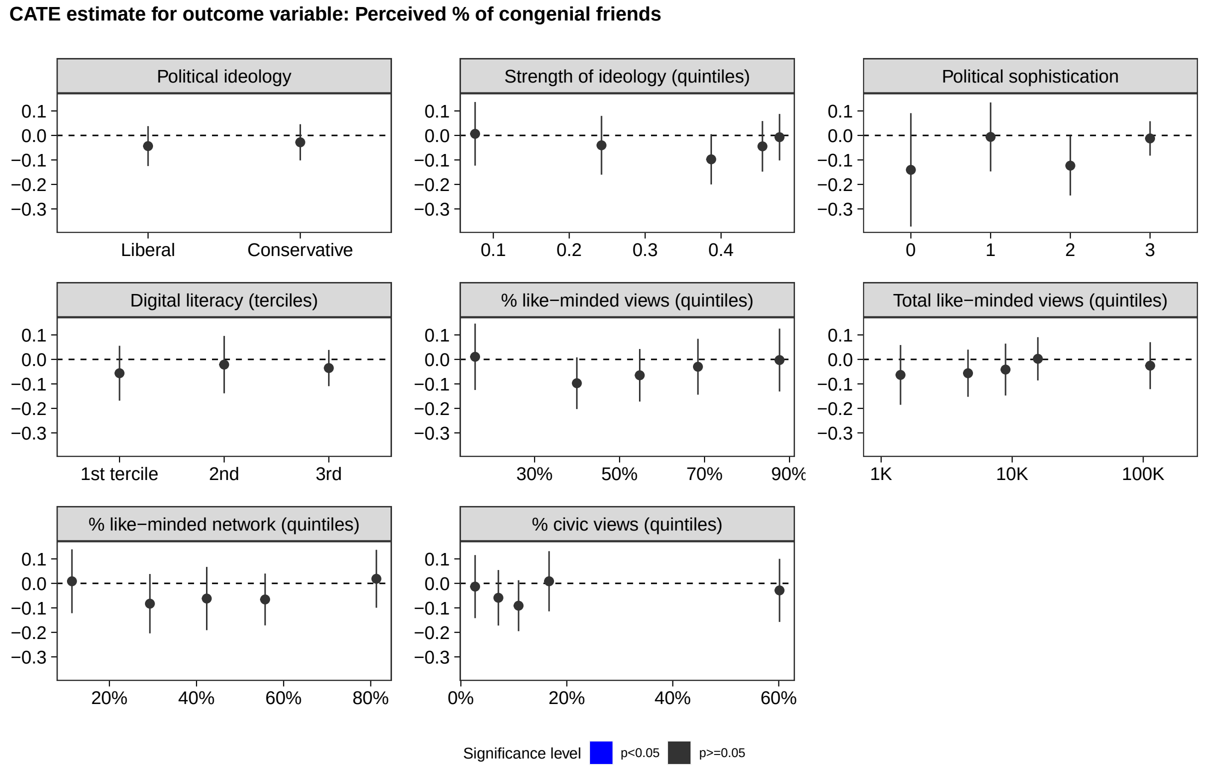

Conditional average treatment effects (including 95% confidence intervals) of reducing exposure to like-minded sources in the Facebook Feed from September 24–December 23, 2020 on the perceived percentage of Facebook friends who are like-minded across several preregistered moderators. Estimates were constructed using the DR-Learner approach (*57–59*). Tests of significance consider the two-tailed t-statistic for the test of whether the subgroup treatment effect is different from zero after controlling the false discovery rate (*55*). Political ideology and sophistication were treated as discrete variables; other moderators were treated as continuous. Survey outcome measures are standardized scales averaged across surveys conducted November 4–18, 2020 and/or December 9–23, 2020. N = 21,071. After adjusting for multiple comparisons, all p-values equal 1.

Figure 36: Ensemble weights for model of heterogeneous treatment effects of reducing exposure to like-minded sources on perceived percentage of Facebook friends with differing political views

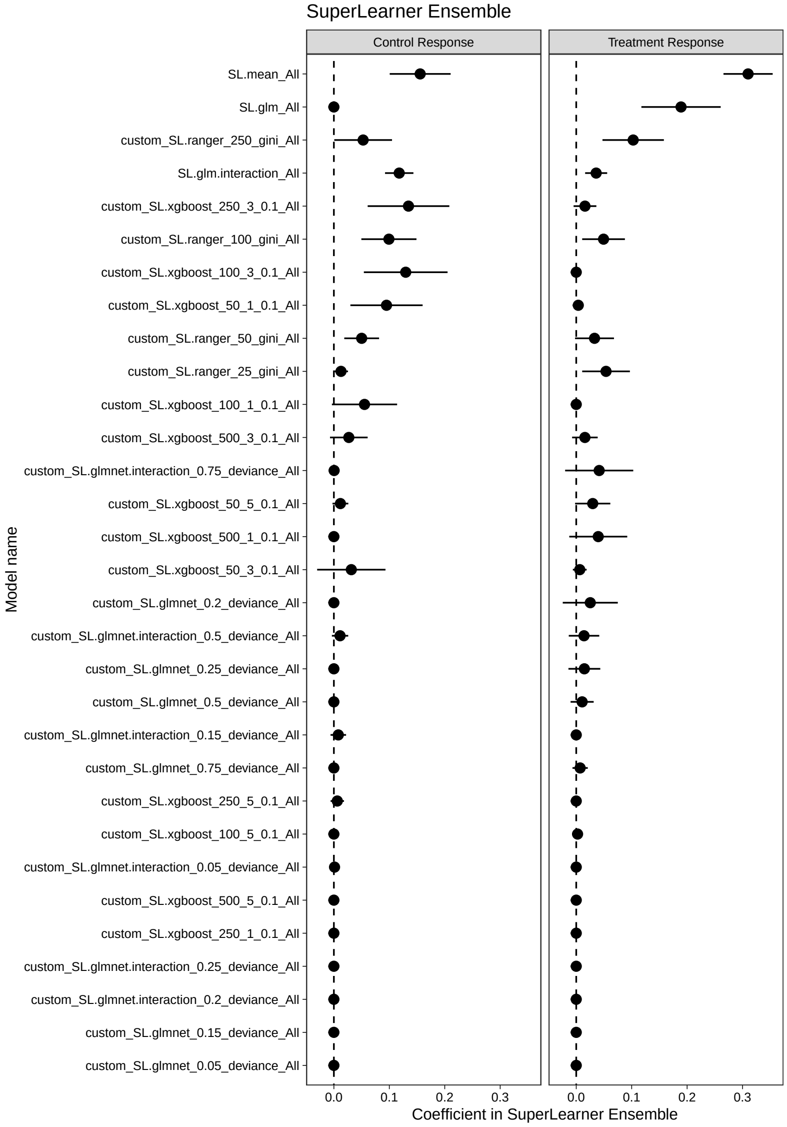

Ensemble weights (including 95% confidence intervals) estimated using the DR-Learner approach (*57–59*) for models testing for heterogeneous treatment effects of reducing exposure to like-minded sources in the Facebook Feed from September 24–December 23, 2020 on perceived percentage of Facebook friends with differing political views across several preregistered moderators. N = 21,070.

Figure 37: Heterogeneous treatment effects of reducing exposure to like-minded sources on perceived percentage of Facebook friends with differing political views

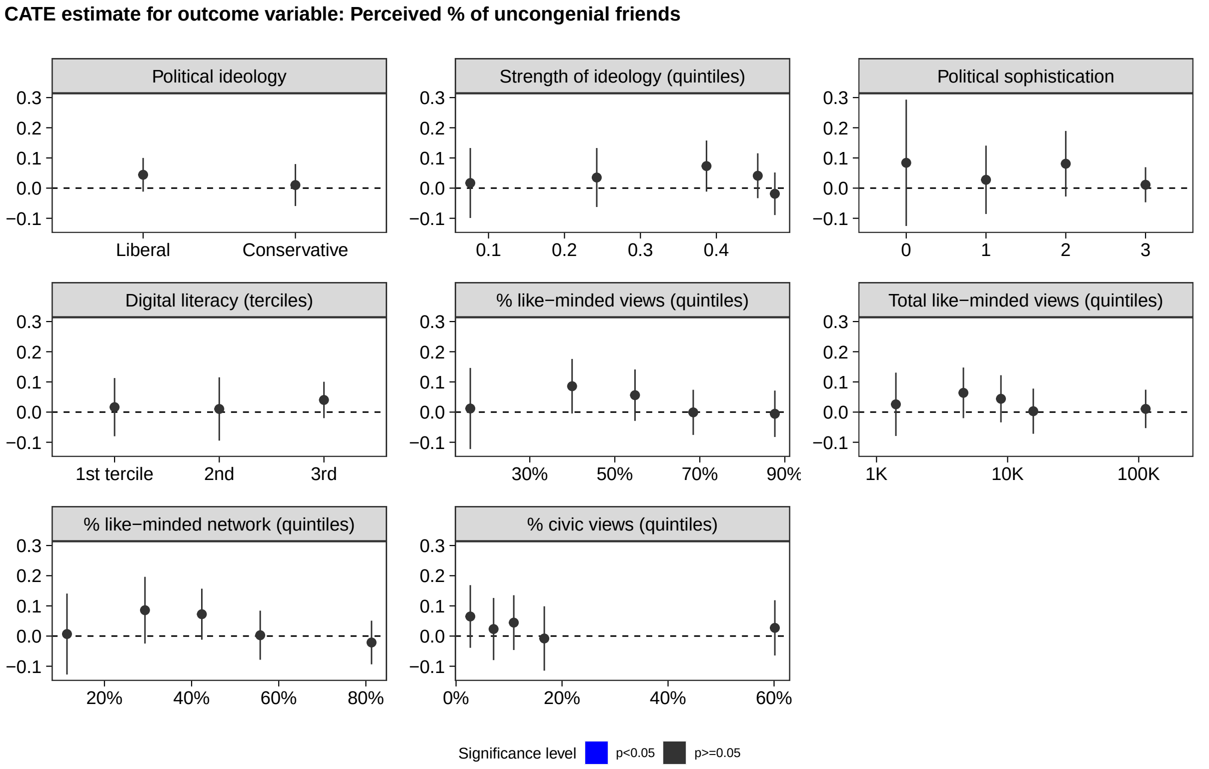

Conditional average treatment effects (including 95% confidence intervals) of reducing exposure to like-minded sources in the Facebook Feed from September 24–December 23, 2020 on the perceived percentage of Facebook friends with differing political views across several preregistered moderators. Estimates were constructed using the DR-Learner approach (*57–59*). Tests of significance consider the two-tailed t-statistic for the test of whether the subgroup treatment effect is different from zero after controlling the false discovery rate (*55*). Political ideology and sophistication were treated as discrete variables; other moderators were treated as continuous. Survey outcome measures are standardized scales averaged across surveys conducted November 4–18, 2020 and/or December 9–23, 2020. N $=$ 21,070. After adjusting for multiple comparisons, all p-values equal 1.

Figure 38: Ensemble weights for model of heterogeneous treatment effects of reducing exposure to like-minded sources on reported frequency of seeing political conversations on Facebook

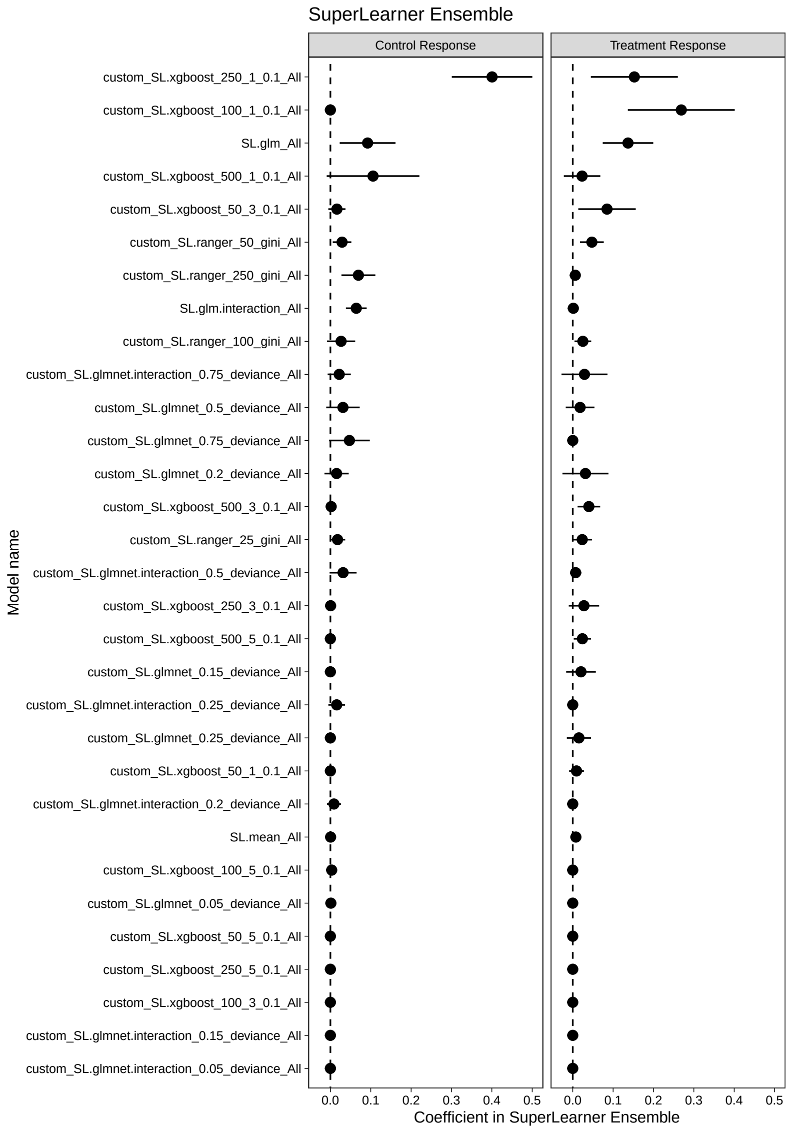

Ensemble weights (including 95% confidence intervals) estimated using the DR-Learner approach (*57–59*) for models testing for heterogeneous treatment effects of reducing exposure to like-minded sources in the Facebook Feed from September 24–December 23, 2020 on reported frequency of seeing political conversations on Facebook across several preregistered moderators. N = 21,355.

Figure 39: Heterogeneous treatment effects of reducing exposure to like-minded sources on reported frequency of seeing political conversations on Facebook

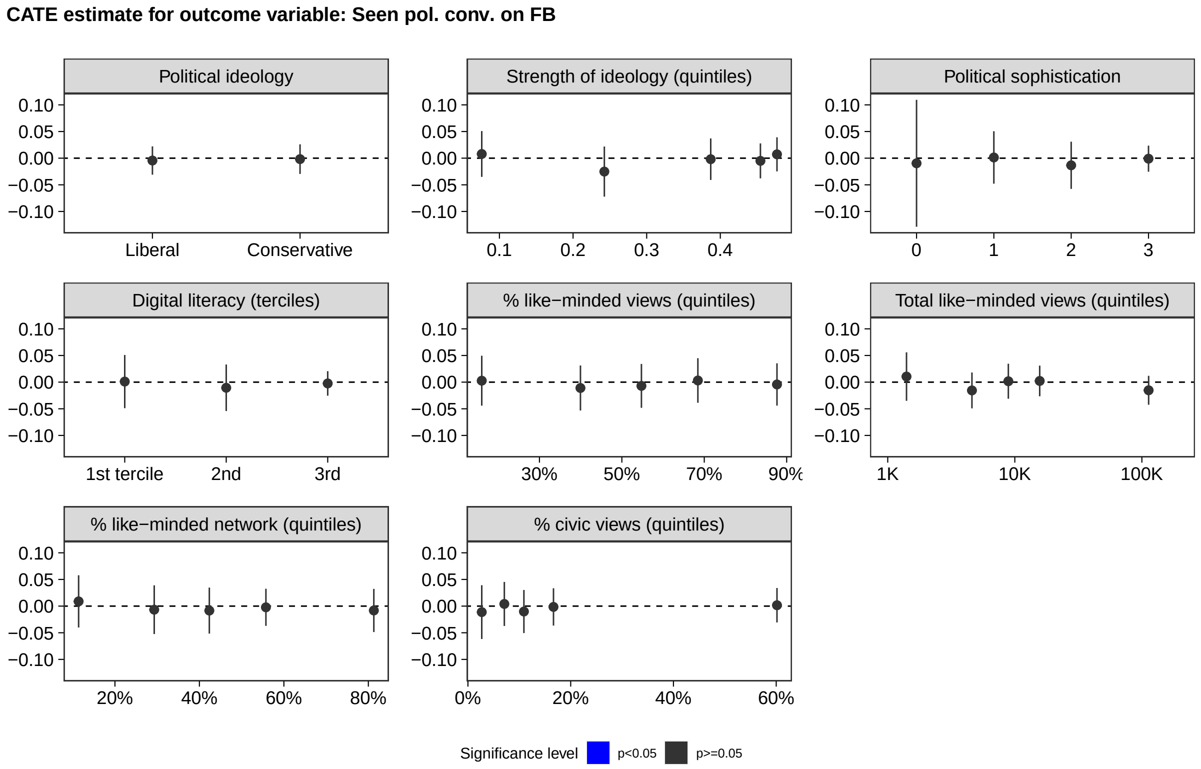

Conditional average treatment effects (including 95% confidence intervals) of reducing exposure to like-minded sources in the Facebook Feed from September 24–December 23, 2020 on reported frequency of seeing political conversations across several preregistered moderators. Estimates were constructed using the DR-Learner approach (*57–59*). Tests of significance consider the two-tailed t-statistic for the test of whether the subgroup treatment effect is different from zero after controlling the false discovery rate (*55*). Political ideology and sophistication were treated as discrete variables; other moderators were treated as continuous. Survey outcome measures are standardized scales averaged across surveys conducted November 4–18, 2020 and/or December 9–23, 2020. N = 21,355. After adjusting for multiple comparisons, all p-values equal 1.

Figure 40: Ensemble weights for model of heterogeneous treatment effects of reducing exposure to like-minded sources on perceived respectfulness of political conversations on Facebook

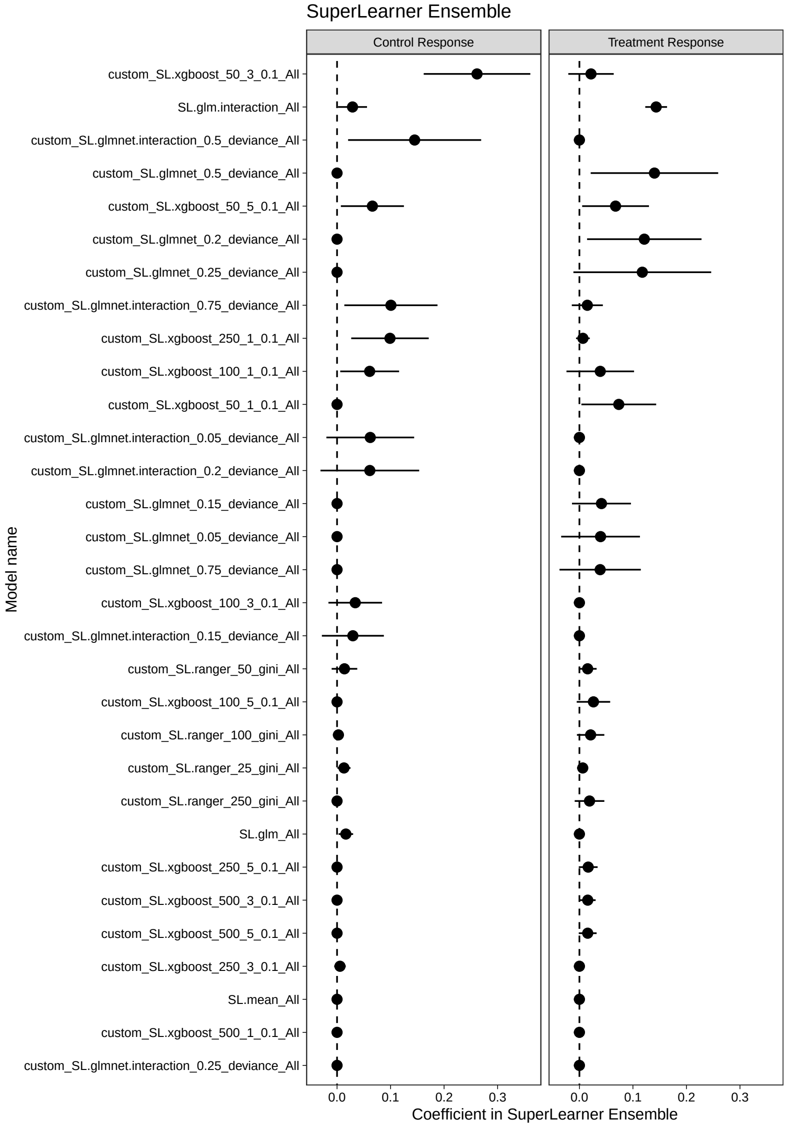

Ensemble weights (including 95% confidence intervals) estimated using the DR-Learner approach (*57–59*) for models testing for heterogeneous treatment effects of reducing exposure to like-minded sources in the Facebook Feed from September 24–December 23, 2020 on perceived respectfulness of political conversations on Facebook across several preregistered moderators. N = 19,061.

Figure 41: Heterogeneous treatment effects of reducing exposure to like-minded sources on perceived respectfulness of political conversations on Facebook

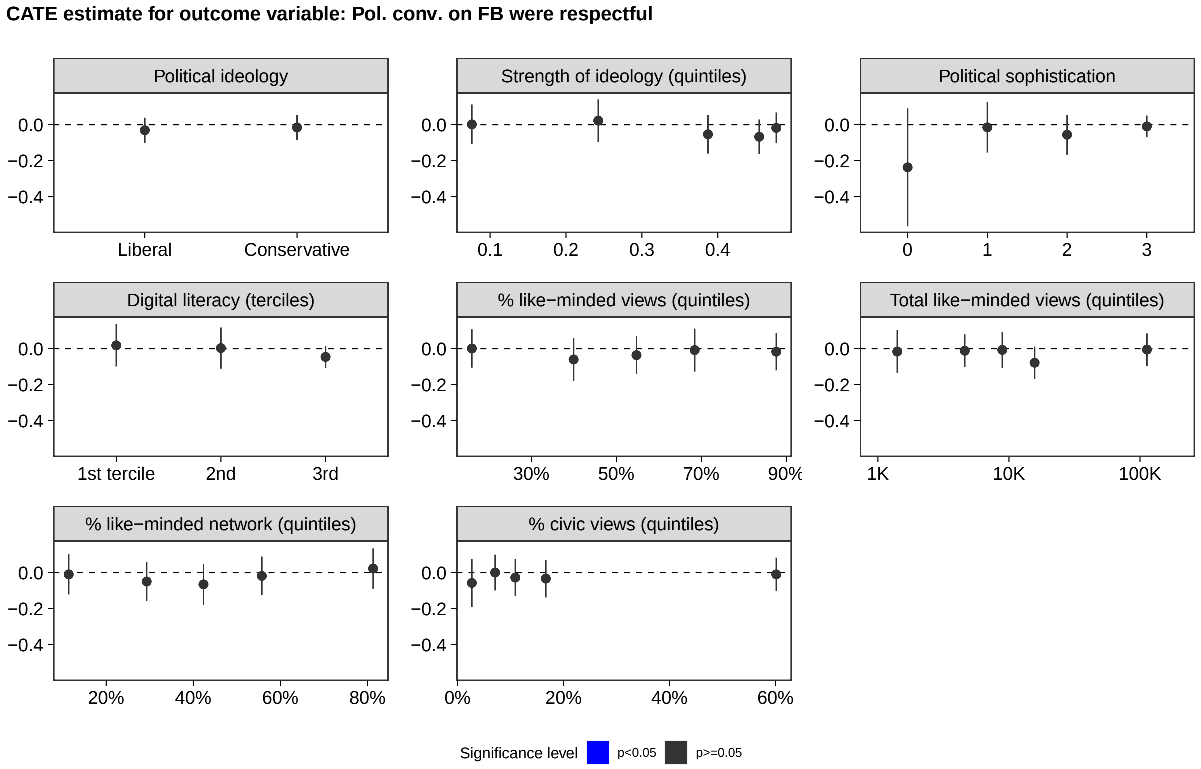

Conditional average treatment effects (including 95% confidence intervals) of reducing exposure to like-minded sources in the Facebook Feed from September 24–December 23, 2020 on perceived respectfulness of political conversations on Facebook across several preregistered moderators. Estimates were constructed using the DR-Learner approach (*57–59*). Tests of significance consider the two-tailed t-statistic for the test of whether the subgroup treatment effect is different from zero after controlling the false discovery rate (*55*). Political ideology and sophistication were treated as discrete variables; other moderators were treated as continuous. Survey outcome measures are standardized scales averaged across surveys conducted November 4–18, 2020 and/or December 9–23, 2020. N = 19,061. After adjusting for multiple comparisons, all p-values equal 1.

Figure 42: Ensemble weights for model of heterogeneous treatment effects of reducing exposure to like-minded sources on perceived informativeness of political conversations on Facebook

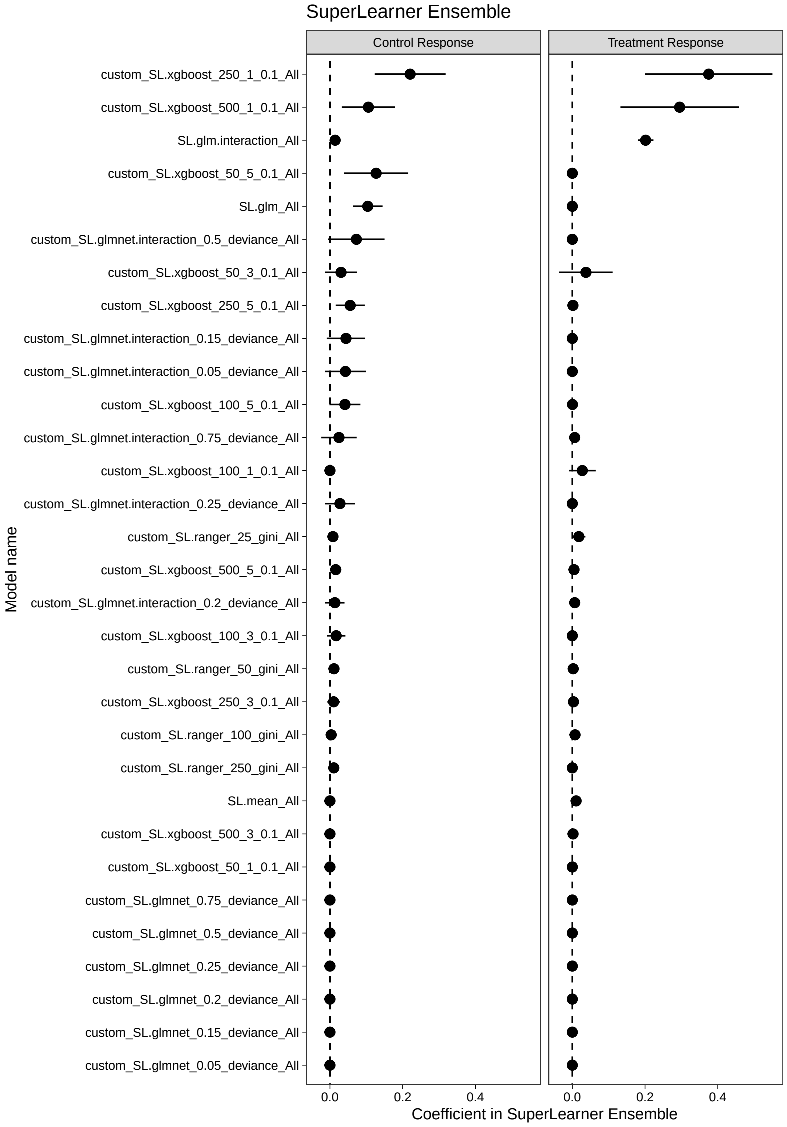

Ensemble weights (including 95% confidence intervals) estimated using the DR-Learner approach (*57–59*) for models testing for heterogeneous treatment effects of reducing exposure to like-minded sources in the Facebook Feed from September 24–December 23, 2020 on perceived informativeness of political conversations on Facebook across several preregistered moderators. N = 19,063.

Figure 43: Heterogeneous treatment effects of reducing exposure to like-minded sources on perceived informativeness of political conversations on Facebook

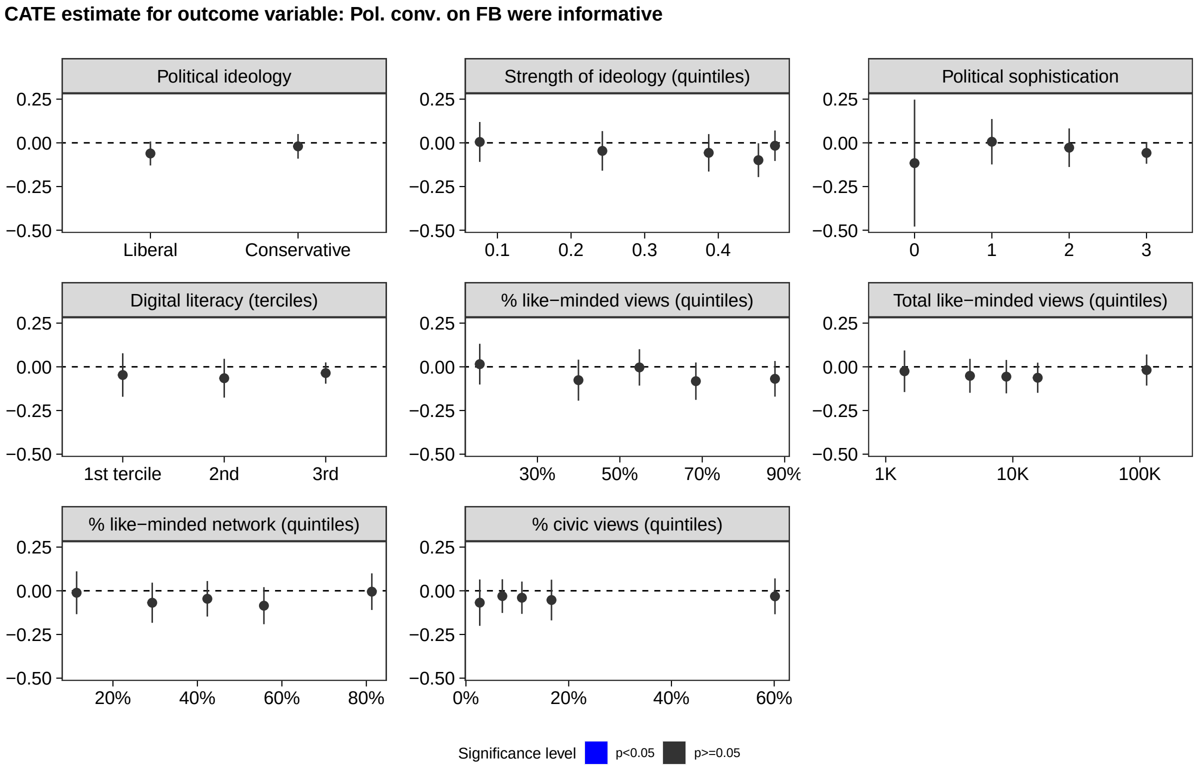

Conditional average treatment effects (including 95% confidence intervals) of reducing exposure to like-minded sources in the Facebook Feed from September 24–December 23, 2020 on perceived informativeness of political conversations on Facebook across several preregistered moderators. Estimates were constructed using the DR-Learner approach (*57–59*). Tests of significance consider the two-tailed t-statistic for the test of whether the subgroup treatment effect is different from zero after controlling the false discovery rate (*55*). Political ideology and sophistication were treated as discrete variables; other moderators were treated as continuous. Survey outcome measures are standardized scales averaged across surveys conducted November 4–18, 2020 and/or December 9–23, 2020. N $=$ 19,063. After adjusting for multiple comparisons, all p-values equal 1.

Figure 44: Ensemble weights for model of heterogeneous treatment effects of reducing exposure to like-minded sources on perceived polarization

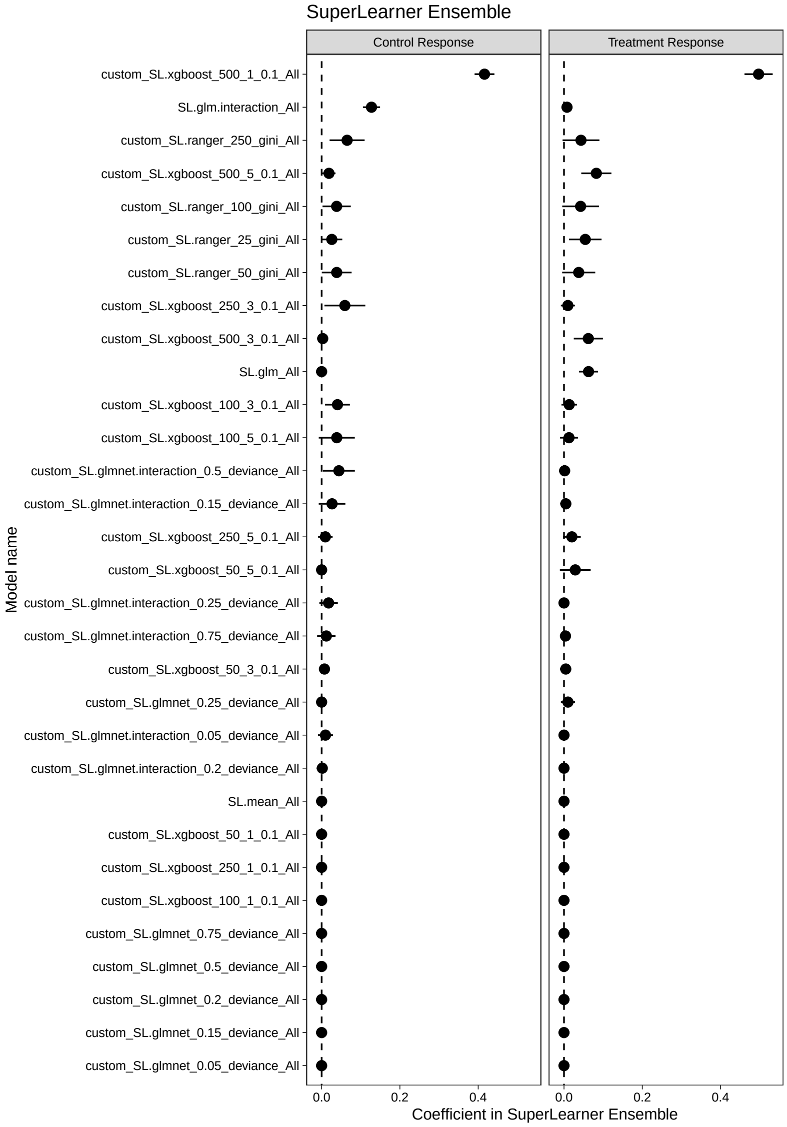

Ensemble weights (including 95% confidence intervals) estimated using the DR-Learner approach (*57–59*) for models testing for heterogeneous treatment effects of reducing exposure to like-minded sources in the Facebook Feed from September 24–December 23, 2020 on perceived polarization across several preregistered moderators. N = 21,157.

Figure 45: Heterogeneous treatment effects of reducing exposure to like-minded sources on perceived polarization

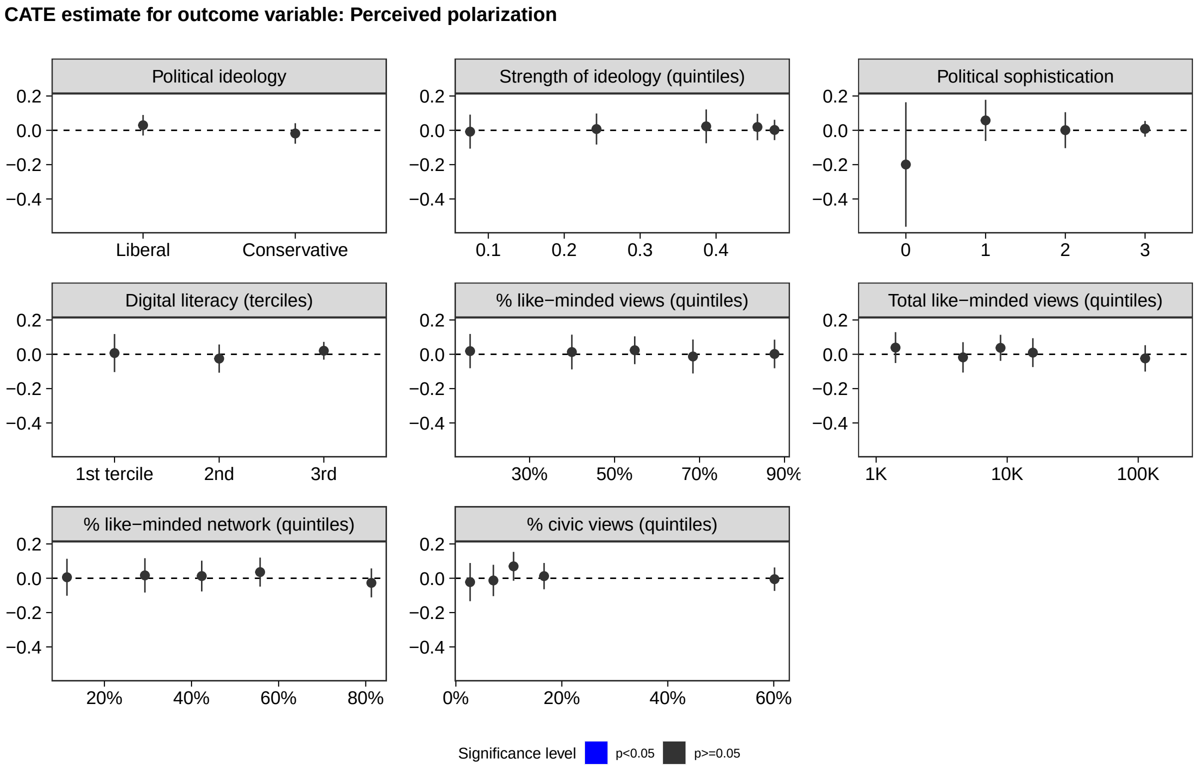

Conditional average treatment effects (including 95% confidence intervals) of reducing exposure to like-minded sources in the Facebook Feed from September 24–December 23, 2020 on perceived political polarization across several preregistered moderators. Estimates were constructed using the DR-Learner approach (*57–59*). Tests of significance consider the two-tailed t-statistic for the test of whether the subgroup treatment effect is different from zero after controlling the false discovery rate (*55*). Political ideology and sophistication were treated as discrete variables; other moderators were treated as continuous. Survey outcome measures are standardized scales averaged across surveys conducted November 4–18, 2020 and/or December 9–23, 2020. N $=$ 21,157. After adjusting for multiple comparisons, all p-values equal 1.

Figure 46: Ensemble weights for model of heterogeneous treatment effects of reducing exposure to like-minded sources on own-party affinity

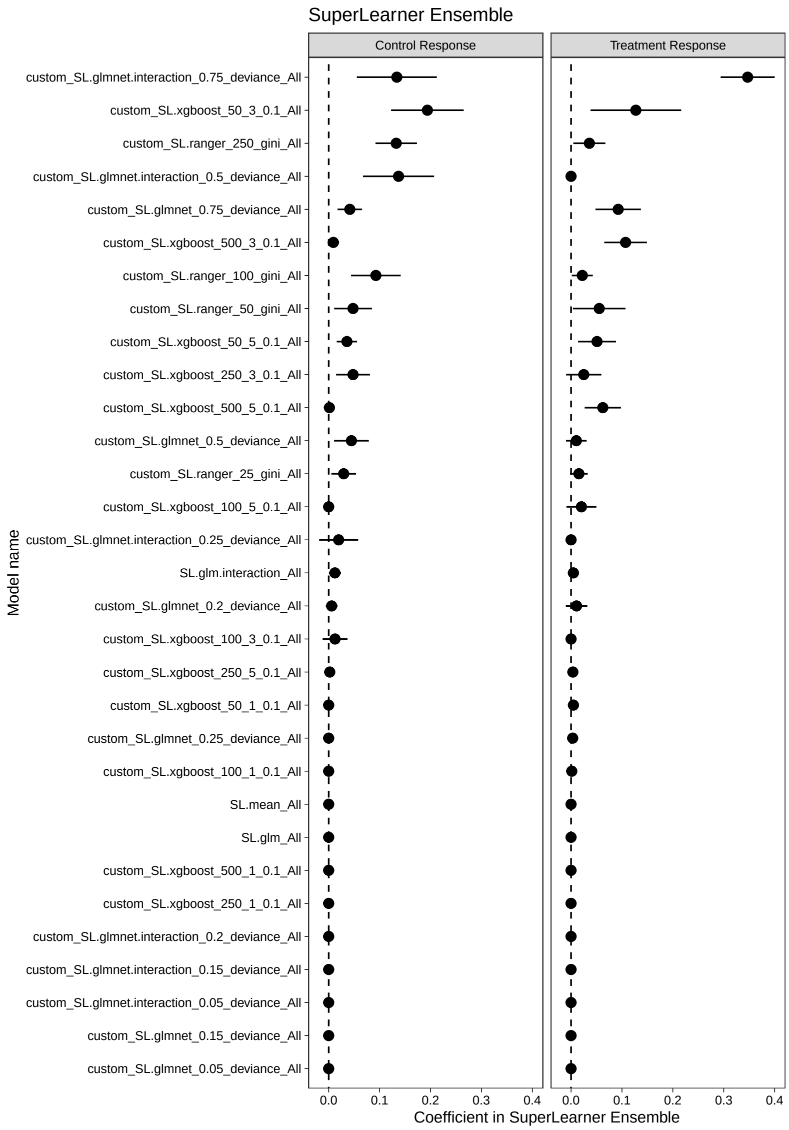

Ensemble weights (including 95% confidence intervals) estimated using the DR-Learner approach (*57–59*) for models testing for heterogeneous treatment effects of reducing exposure to like-minded sources in the Facebook Feed from September 24–December 23, 2020 on own-party affinity across several preregistered moderators. N = 22,136.

Figure 47: Heterogeneous treatment effects of reducing exposure to like-minded sources on own-party affinity

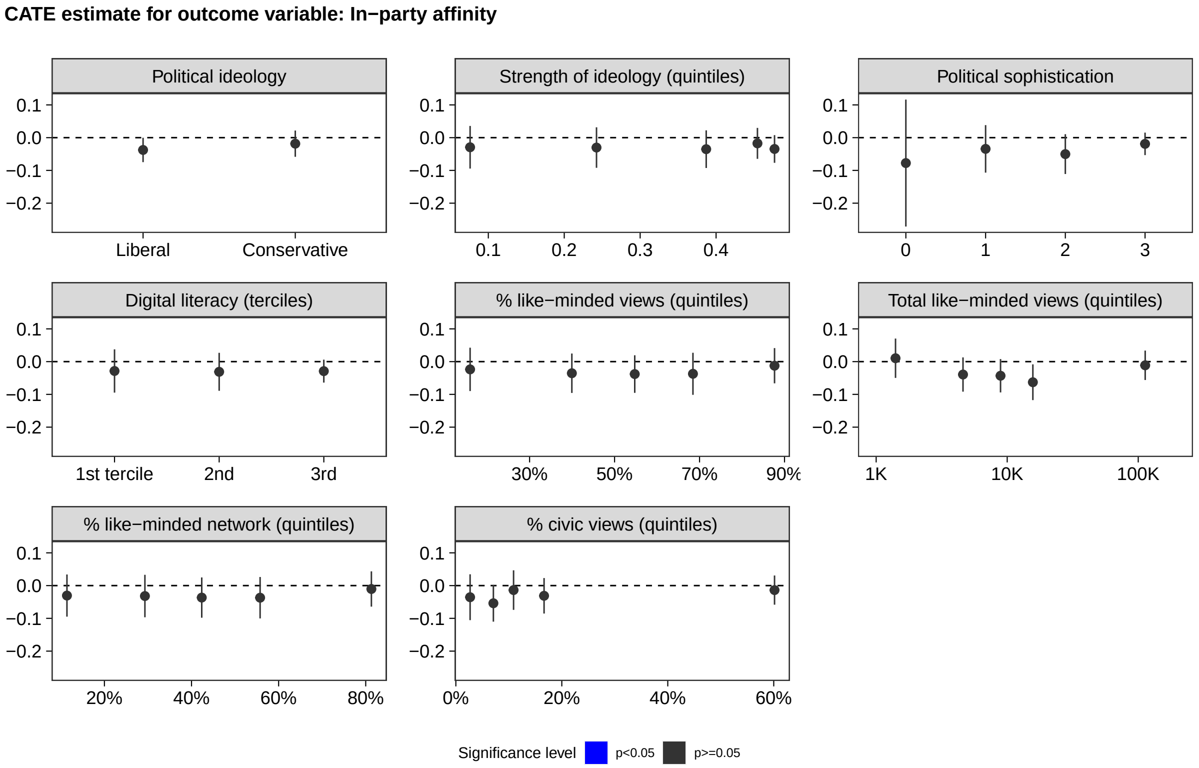

Conditional average treatment effects (including 95% confidence intervals) of reducing exposure to like-minded sources in the Facebook Feed from September 24–December 23, 2020 on own-party affinity across several preregistered moderators. Estimates were constructed using the DR-Learner approach (*57–59*). Tests of significance consider the two-tailed t-statistic for the test of whether the subgroup treatment effect is different from zero after controlling the false discovery rate (*55*). Political ideology and sophistication were treated as discrete variables; other moderators were treated as continuous. Survey outcome measures are standardized scales averaged across surveys conducted November 4–18, 2020 and/or December 9–23, 2020. N = 22,136. After adjusting for multiple comparisons, all p-values equal 1.

Figure 48: Ensemble weights for model of heterogeneous treatment effects of reducing exposure to like-minded sources on out-party antipathy

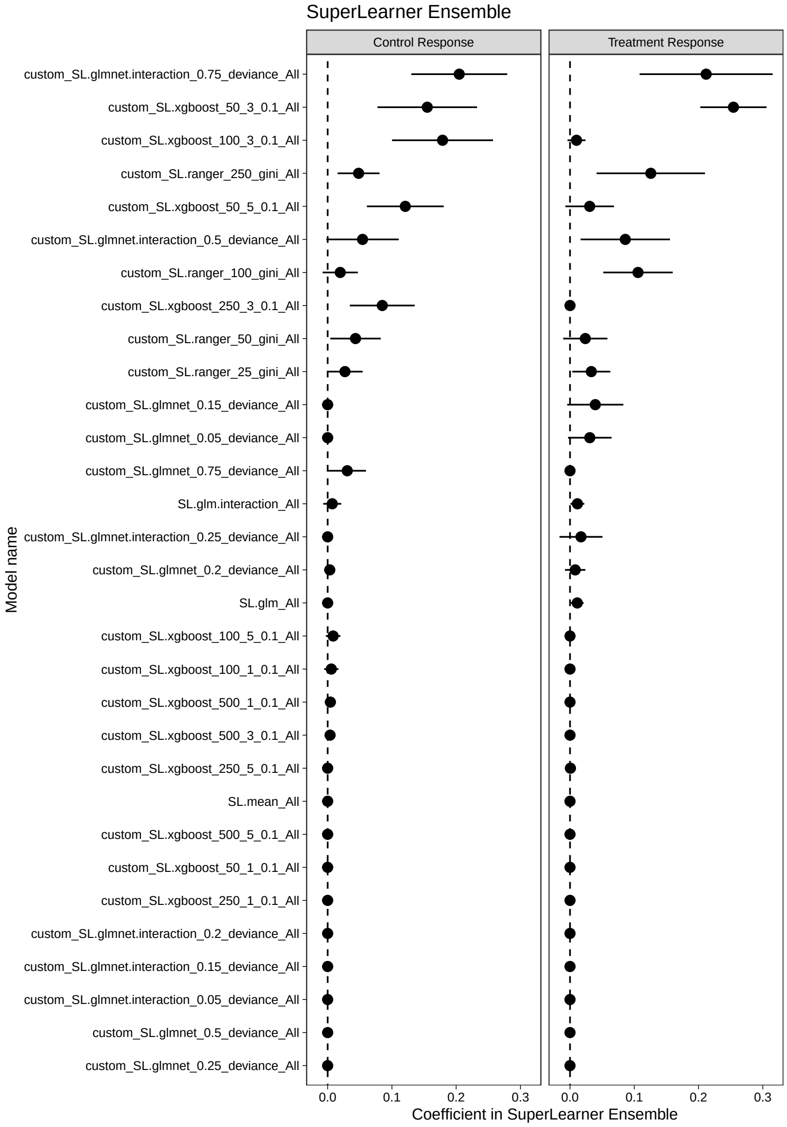

Ensemble weights (including 95% confidence intervals) estimated using the DR-Learner approach (*57–59*) for models testing for heterogeneous treatment effects of reducing exposure to like-minded sources in the Facebook Feed from September 24–December 23, 2020 on out-party antipathy across several preregistered moderators. N = 22,130.

Figure 49: Heterogeneous treatment effects of reducing exposure to like-minded sources on out-party antipathy

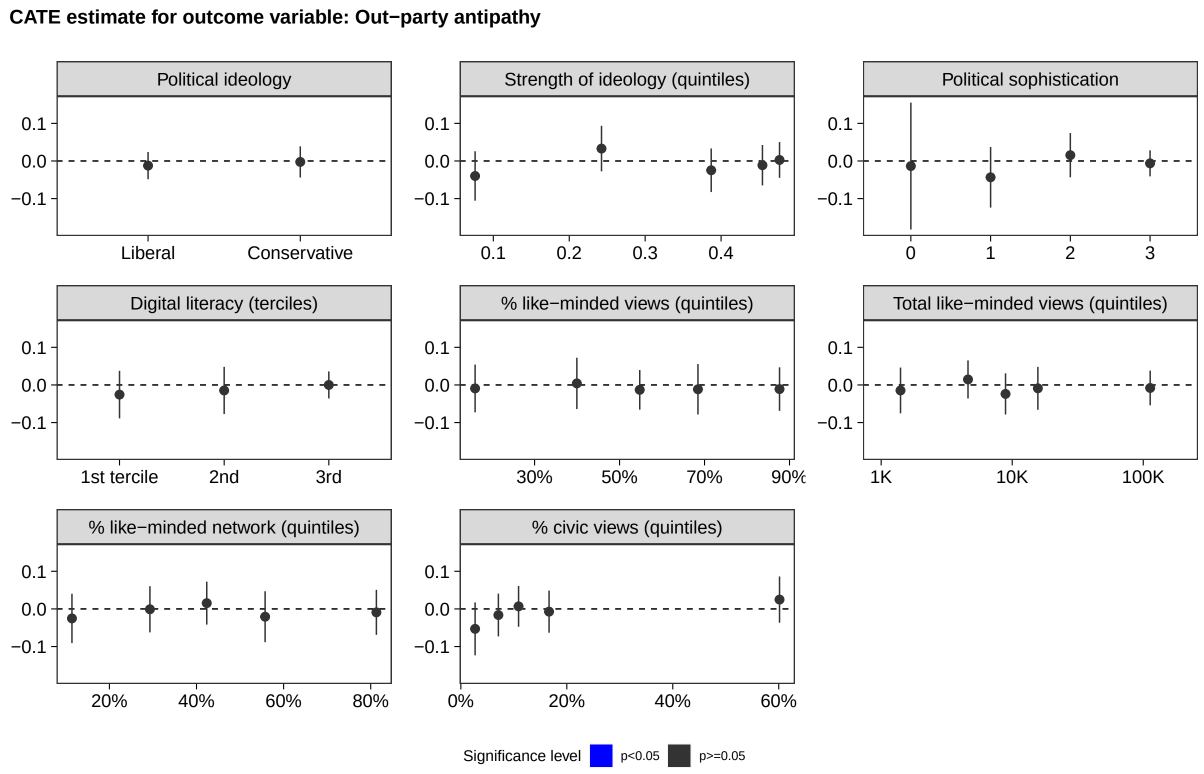

Conditional average treatment effects (including 95% confidence intervals) of reducing exposure to like-minded sources in the Facebook Feed from September 24–December 23, 2020 on out-party antipathy across several preregistered moderators. Estimates were constructed using the DR-Learner approach (*57–59*). Tests of significance consider the two-tailed t-statistic for the test of whether the subgroup treatment effect is different from zero after controlling the false discovery rate (*55*). Political ideology and sophistication were treated as discrete variables; other moderators were treated as continuous. Survey outcome measures are standardized scales averaged across surveys conducted November 4–18, 2020 and/or December 9–23, 2020. N = 22,130. After adjusting for multiple comparisons, all p-values equal 1.

Figure 50: Ensemble weights for model of heterogeneous treatment effects of reducing exposure to like-minded sources on belief in false claims

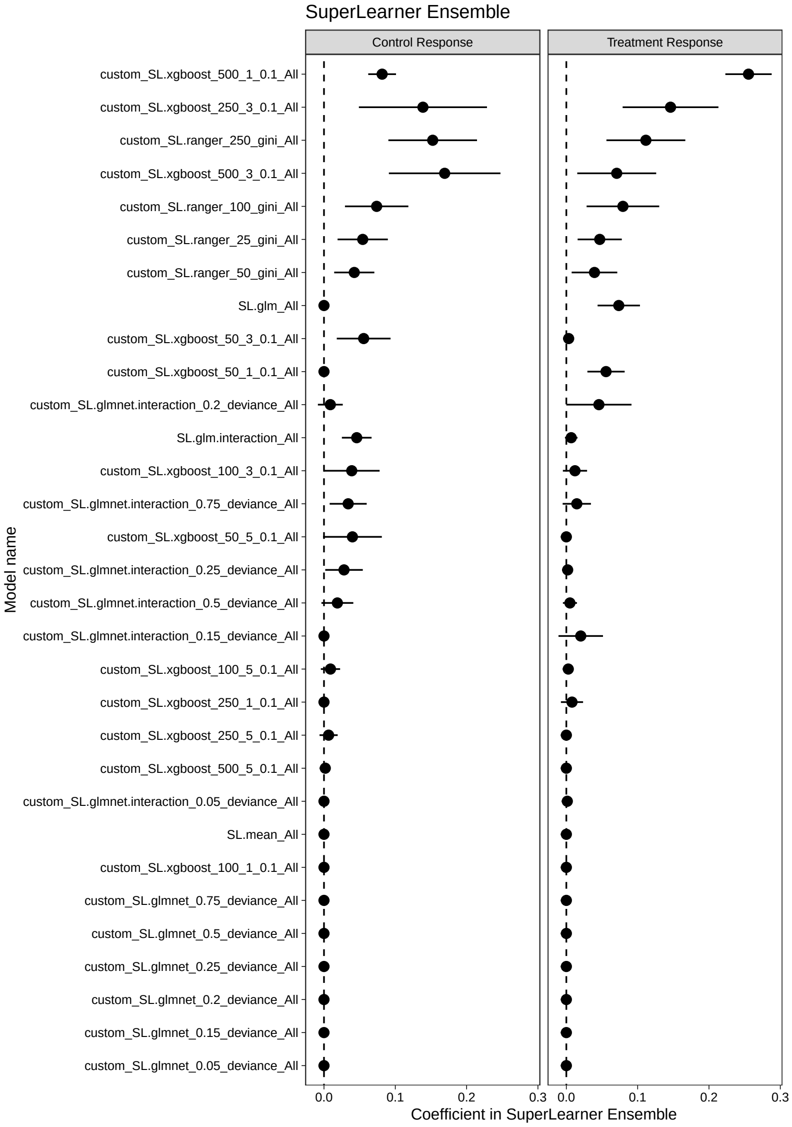

Ensemble weights (including 95% confidence intervals) estimated using the DR-Learner approach (*57–59*) for models testing for heterogeneous treatment effects of reducing exposure to like-minded sources in the Facebook Feed from September 24–December 23, 2020 on belief in false claims across several preregistered moderators. N = 22,136

Figure 51: Heterogeneous treatment effects of reducing exposure to like-minded sources on belief in false claims

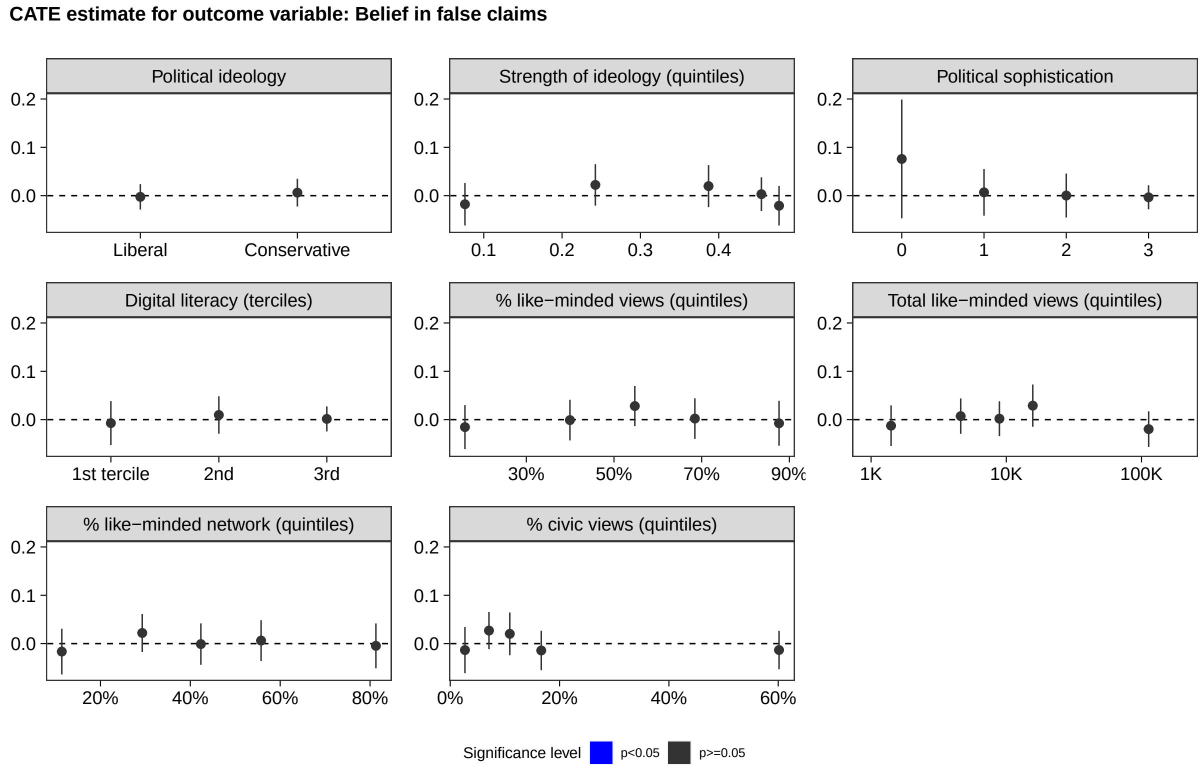

Conditional average treatment effects (including 95% confidence intervals) of reducing exposure to like-minded sources in the Facebook Feed from September 24–December 23, 2020 on belief in false claims across several preregistered moderators. Estimates were constructed using the DR-Learner approach (*57–59*). Tests of significance consider the two-tailed t-statistic for the test of whether the subgroup treatment effect is different from zero after controlling the false discovery rate (*55*). Political ideology and sophistication were treated as discrete variables; other moderators were treated as continuous. Survey outcome measures are standardized scales averaged across surveys conducted November 4–18, 2020 and/or December 9–23, 2020. N = 22,136. After adjusting for multiple comparisons, all p-values equal 1.

Figure 52: Ensemble weights for model of heterogeneous treatment effects of reducing exposure to like-minded sources on pro-attitudinal false claims

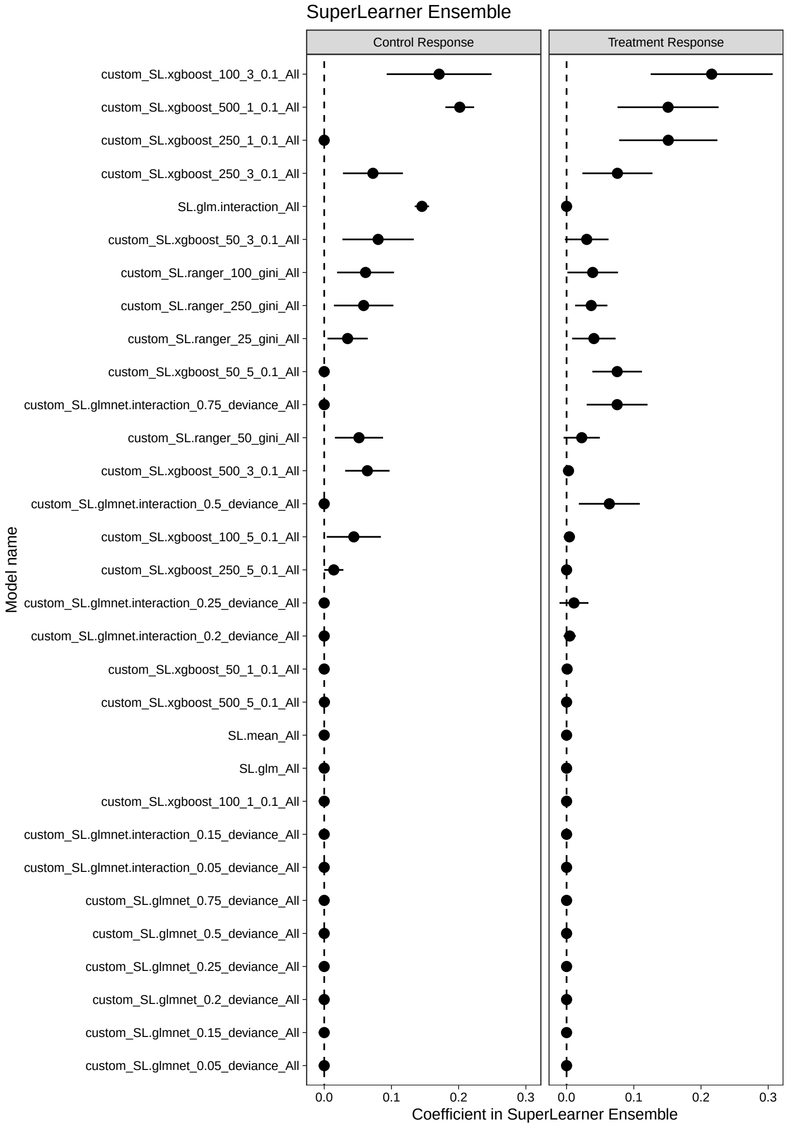

Ensemble weights (including 95% confidence intervals) estimated using the DR-Learner approach (*57–59*) for models testing for heterogeneous treatment effects of reducing exposure to like-minded sources in the Facebook Feed from September 24–December 23, 2020 on pro-attitudinal false claims across several preregistered moderators. N = 22,135.

Figure 53: Heterogeneous treatment effects of reducing exposure to like-minded sources on belief in pro-attitudinal false claims

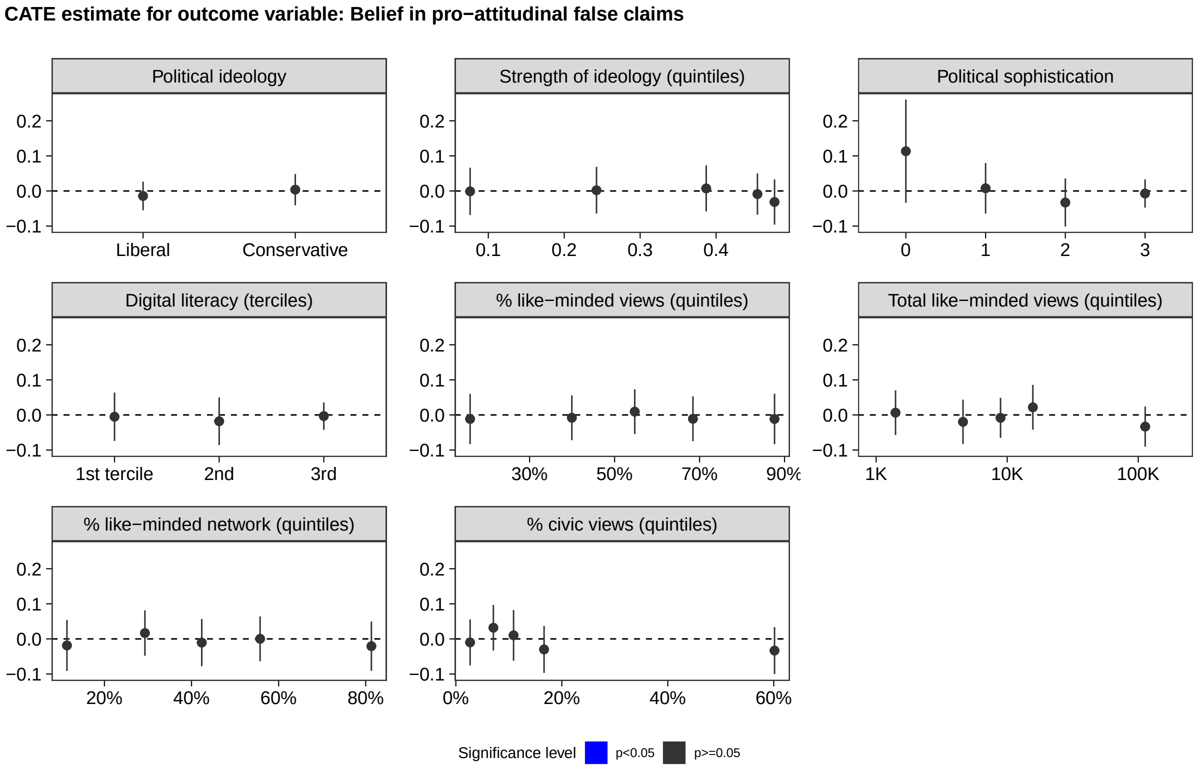

Conditional average treatment effects (including 95% confidence intervals) of reducing exposure to like-minded sources in the Facebook Feed from September 24–December 23, 2020 on belief in false claims across several preregistered moderators. Estimates were constructed using the DR-Learner approach (*57–59*). Tests of significance consider the two-tailed t-statistic for the test of whether the subgroup treatment effect is different from zero after controlling the false discovery rate (*55*). Political ideology and sophistication were treated as discrete variables; other moderators were treated as continuous. Survey outcome measures are standardized scales averaged across surveys conducted November 4–18, 2020 and/or December 9–23, 2020. N = 22,135. After adjusting for multiple comparisons, all p-values equal 1.

Figure 54: Ensemble weights for model of heterogeneous treatment effects of reducing exposure to like-minded sources on accurate knowledge of current events

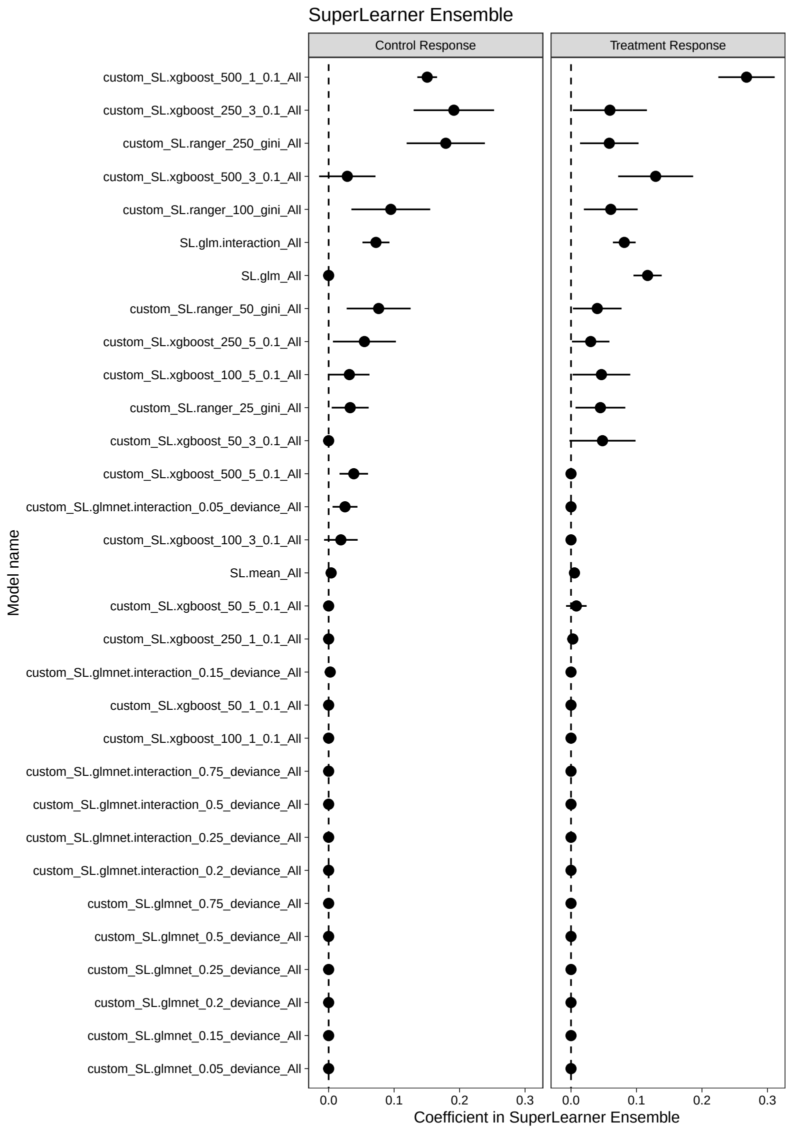

Ensemble weights (including 95% confidence intervals) estimated using the DR-Learner approach (*57–59*) for models testing for heterogeneous treatment effects of reducing exposure to like-minded sources in the Facebook Feed from September 24–December 23, 2020 on knowledge of current events across several preregistered moderators. N = 19,414.

Figure 55: Heterogeneous treatment effects of reducing exposure to like-minded sources on accurate knowledge of current events

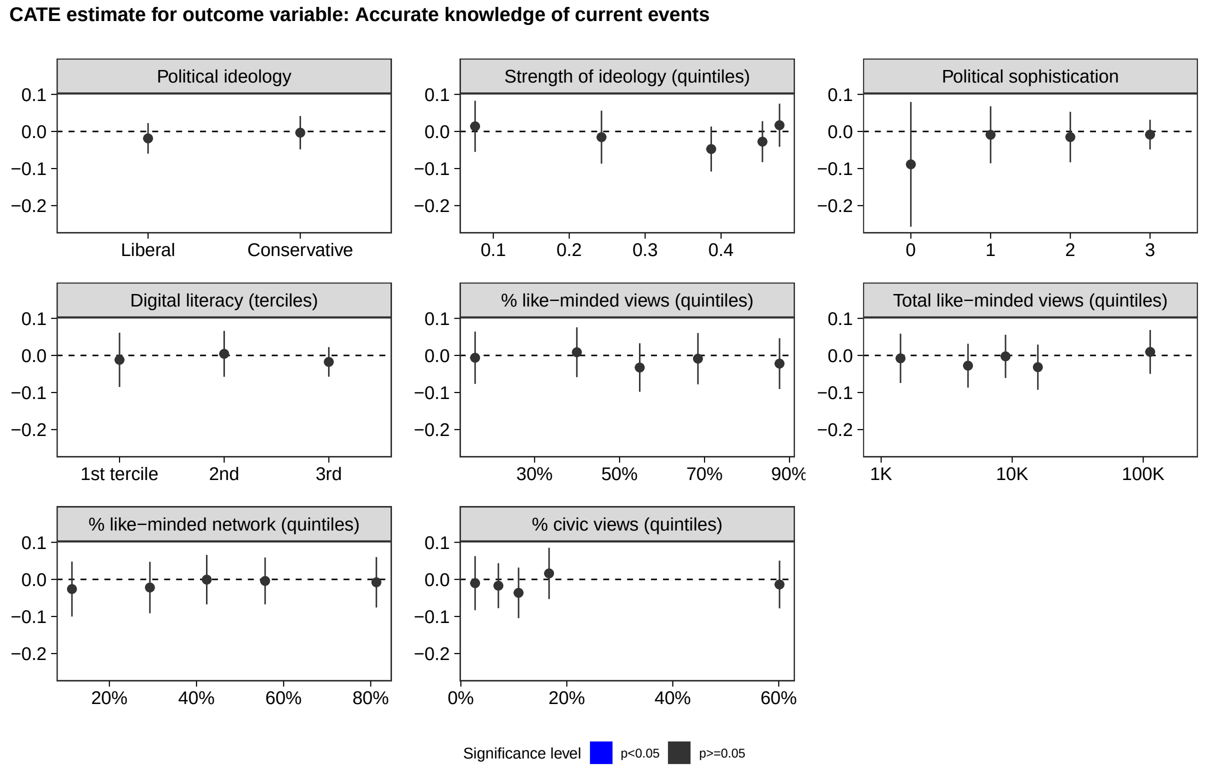

Conditional average treatment effects (including 95% confidence intervals) of reducing exposure to like-minded sources in the Facebook Feed from September 24–December 23, 2020 on knowledge of current events across several preregistered moderators. Estimates were constructed using the DR-Learner approach (*57–59*). Tests of significance consider the two-tailed t-statistic for the test of whether the subgroup treatment effect is different from zero after controlling the false discovery rate (*55*). Political ideology and sophistication were treated as discrete variables; other moderators were treated as continuous. Survey outcome measures are standardized scales averaged across surveys conducted November 4–18, 2020 and/or December 9–23, 2020. N $=$ 19,414. After adjusting for multiple comparisons, all p-values equal 1.

Figure 56: Ensemble weights for model of heterogeneous treatment effects of reducing exposure to like-minded sources on accurate knowledge of pro-attitudinal current events

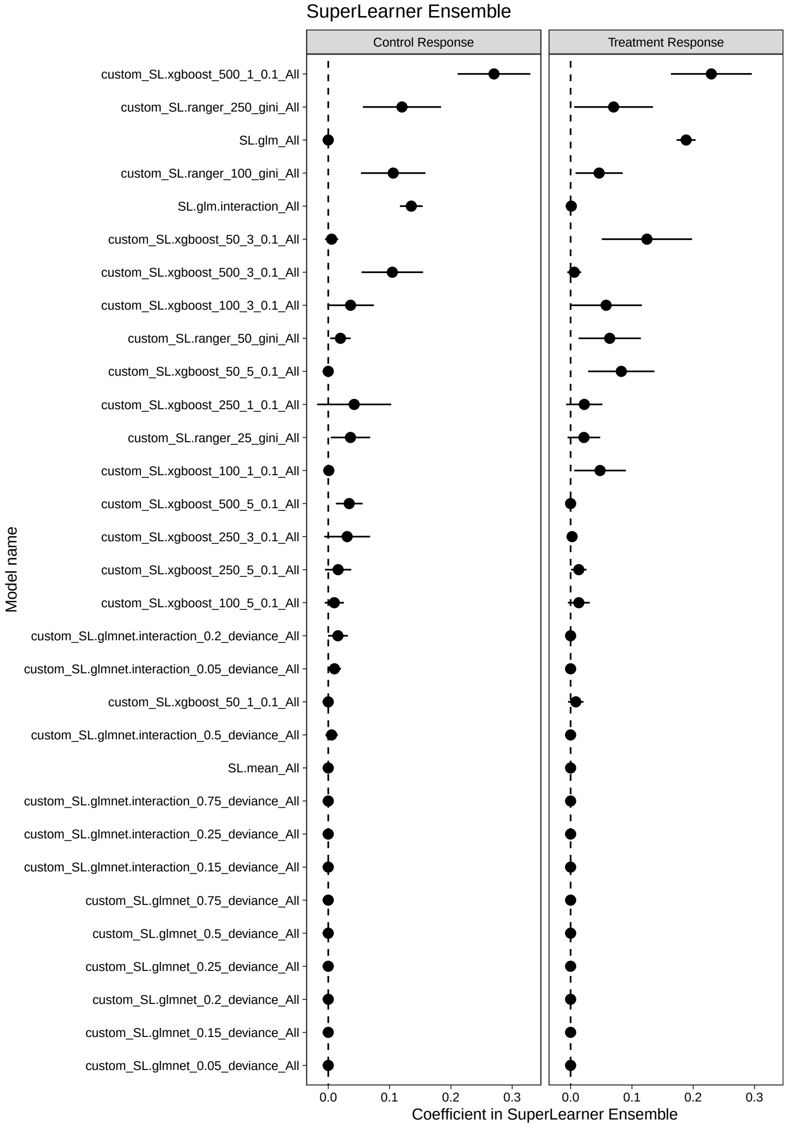

Ensemble weights (including 95% confidence intervals) estimated using the DR-Learner approach (*57*–*59*) for models testing for heterogeneous treatment effects of reducing exposure to like-minded sources in the Facebook Feed from September 24–December 23, 2020 on knowledge of pro-attitudinal current events across several preregistered moderators. N = 19,398.

Figure 57: Heterogeneous treatment effects of reducing exposure to like-minded sources on accurate knowledge of pro-attitudinal current events

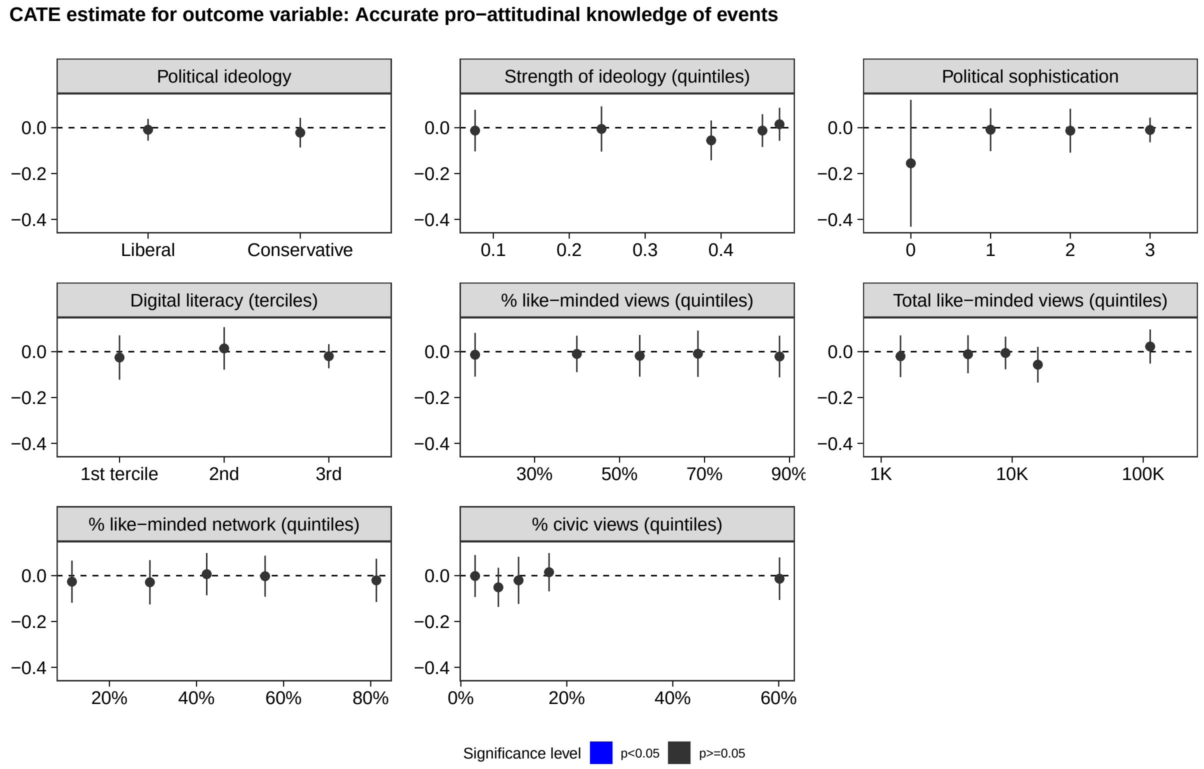

Conditional average treatment effects (including 95% confidence intervals) of reducing exposure to like-minded sources in the Facebook Feed from September 24–December 23, 2020 on knowledge of pro-attitudinal current events across several preregistered moderators. Estimates were constructed using the DR-Learner approach (*57–59*). Tests of significance consider the two-tailed t-statistic for the test of whether the subgroup treatment effect is different from zero after controlling the false discovery rate (*55*). Political ideology and sophistication were treated as discrete variables; other moderators were treated as continuous. Survey outcome measures are standardized scales averaged across surveys conducted November 4–18, 2020 and/or December 9–23, 2020. N = 19,398. After adjusting for multiple comparisons, all p-values equal 1.

Figure 58: Ensemble weights for model of heterogeneous treatment effects of reducing exposure to like-minded sources on perceived democratic performance

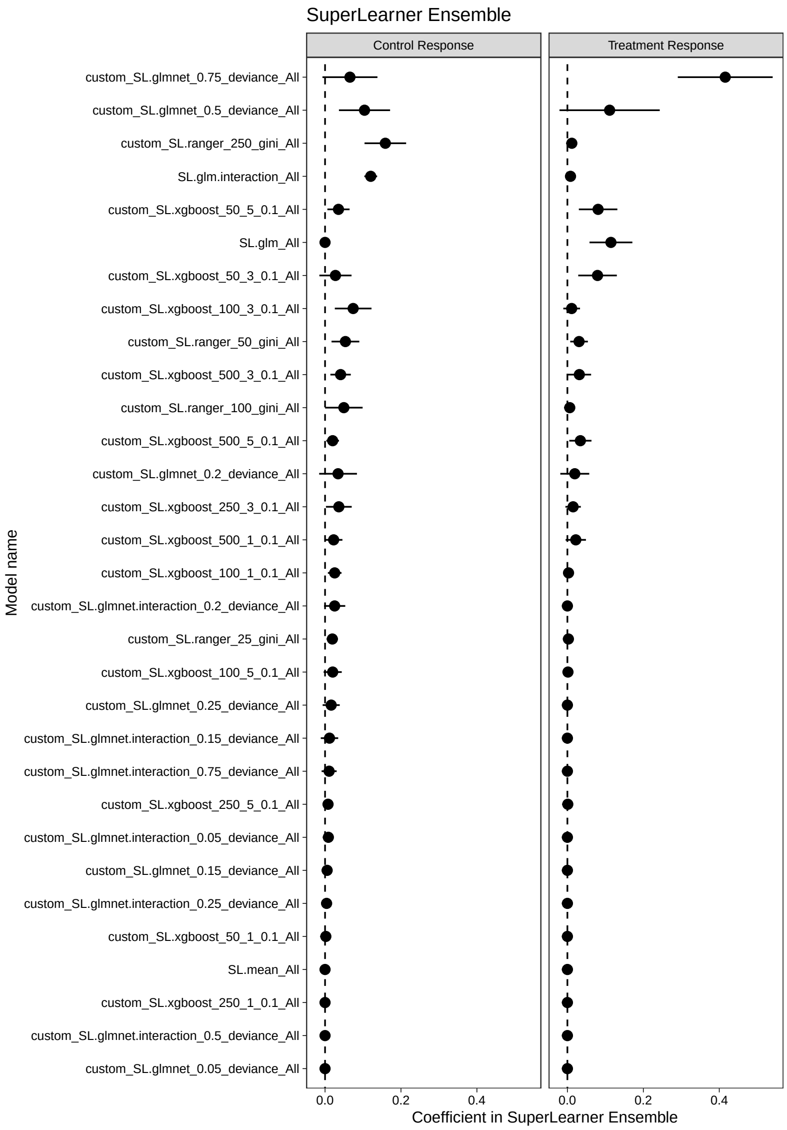

Ensemble weights (including 95% confidence intervals) estimated using the DR-Learner approach (*57–59*) for models testing for heterogeneous treatment effects of reducing exposure to like-minded sources in the Facebook Feed from September 24–December 23, 2020 on perceived democratic performance across several preregistered moderators. N = 22,138.

Figure 59: Heterogeneous treatment effects of reducing exposure to like-minded sources on perceived democratic performance

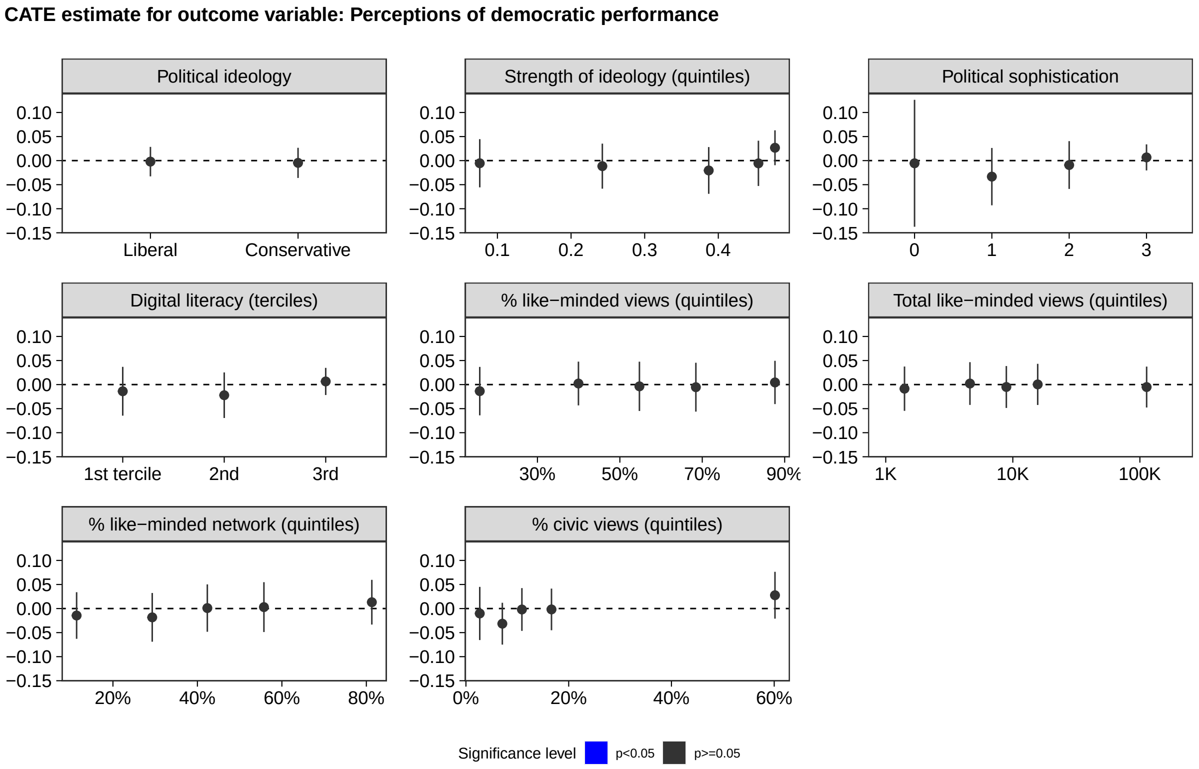

Conditional average treatment effects (including 95% confidence intervals) of reducing exposure to like-minded sources in the Facebook Feed from September 24–December 23, 2020 on perceived democratic performance across several preregistered moderators. Estimates were constructed using the DR-Learner approach (*57–59*). Tests of significance consider the two-tailed t-statistic for the test of whether the subgroup treatment effect is different from zero after controlling the false discovery rate (*55*). Political ideology and sophistication were treated as discrete variables; other moderators were treated as continuous. Survey outcome measures are standardized scales averaged across surveys conducted November 4–18, 2020 and/or December 9–23, 2020. N = 22,138. After adjusting for multiple comparisons, all p-values equal 1.

Figure 60: Ensemble weights for model of heterogeneous treatment effects of reducing exposure to like-minded sources on ideological extremity of online news diet (web browsing)

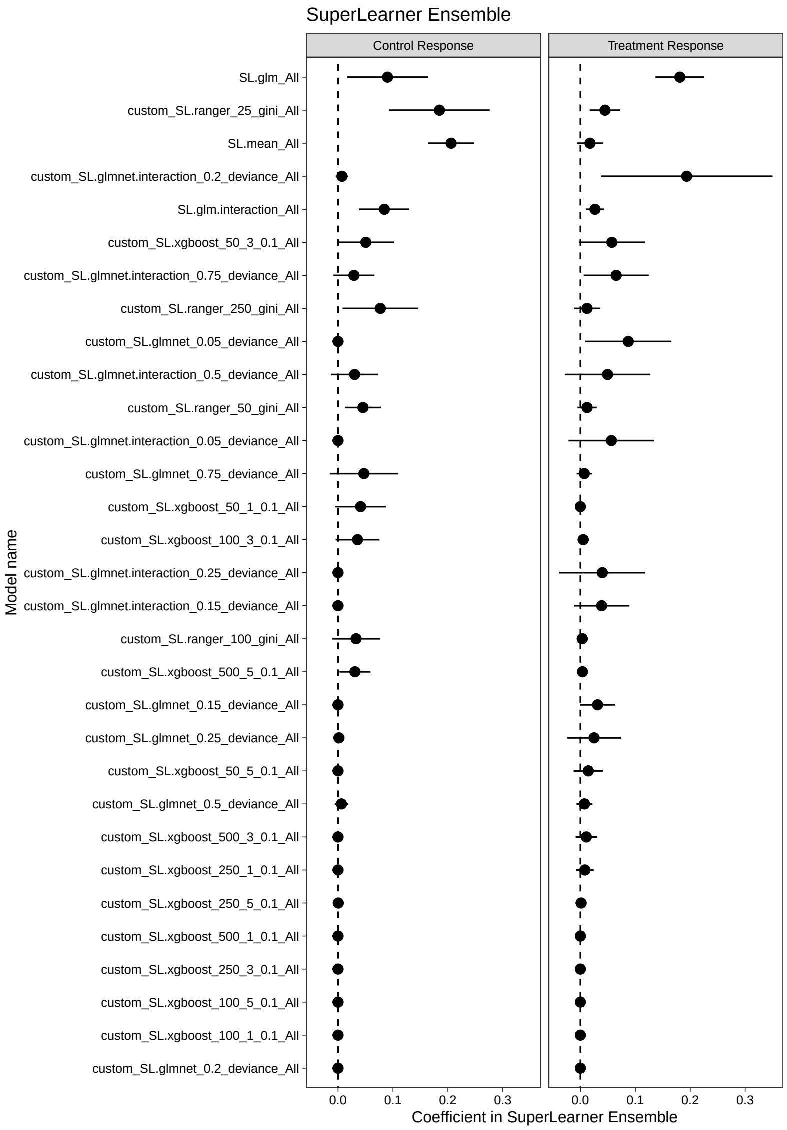

Ensemble weights (including 95% confidence intervals) estimated using the DR-Learner approach (*57–59*) for models testing for heterogeneous treatment effects of reducing exposure to like-minded sources in the Facebook Feed from September 24–December 23, 2020 on ideological extremity of online news diet (web browsing) across several preregistered moderators. N = 3,380.

Figure 61: Heterogeneous treatment effects of reducing exposure to like-minded sources on ideological extremity of online news diet (web browsing)

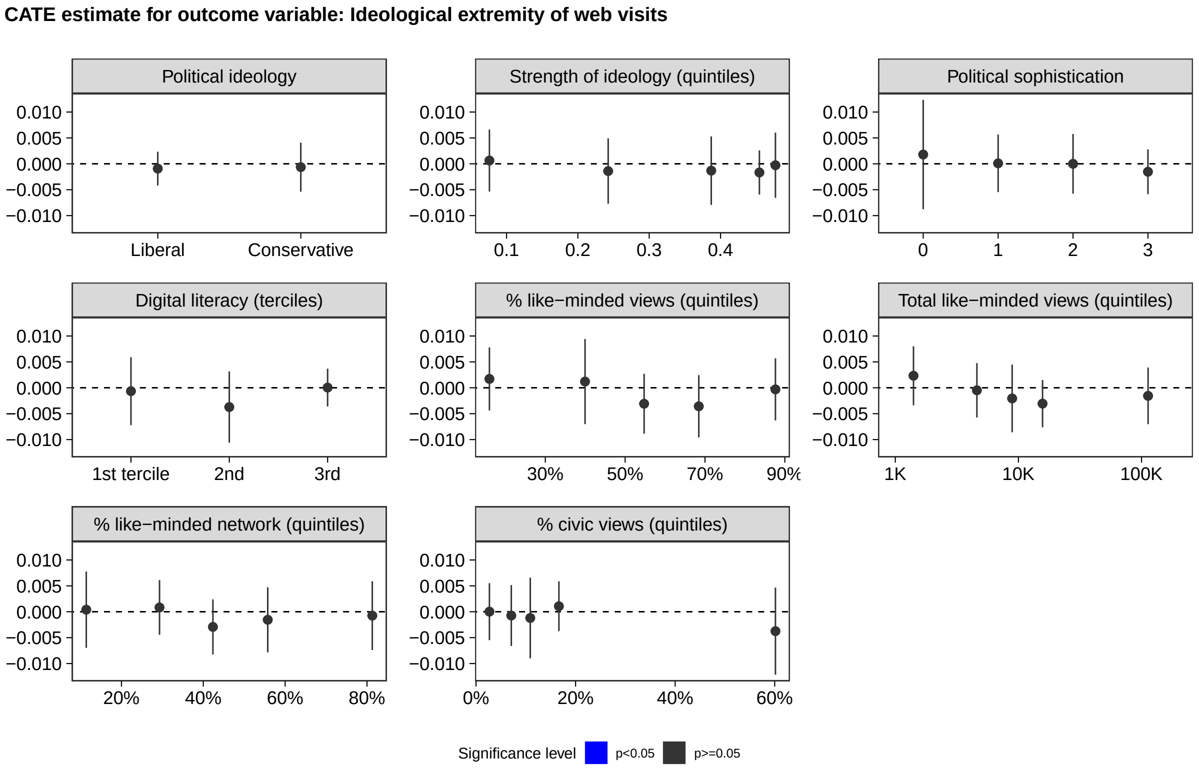

Conditional average treatment effects (including 95% confidence intervals) of reducing exposure to like-minded sources in the Facebook Feed from September 24–December 23, 2020 on ideological extremity of online news diet (web browsing) across several preregistered moderators. Estimates were constructed using the DR-Learner approach (*57–59*). Tests of significance consider the two-tailed t-statistic for the test of whether the subgroup treatment effect is different from zero after controlling the false discovery rate (*55*). Political ideology and sophistication were treated as discrete variables; other moderators were treated as continuous. Survey outcome measures are standardized scales averaged across surveys conducted November 4–18, 2020 and/or December 9–23, 2020. N $=$ 3,380. After adjusting for multiple comparisons, all p-values equal 1.

Figure 62: Variable importance estimates of heterogeneous treatment effects for primary hypotheses

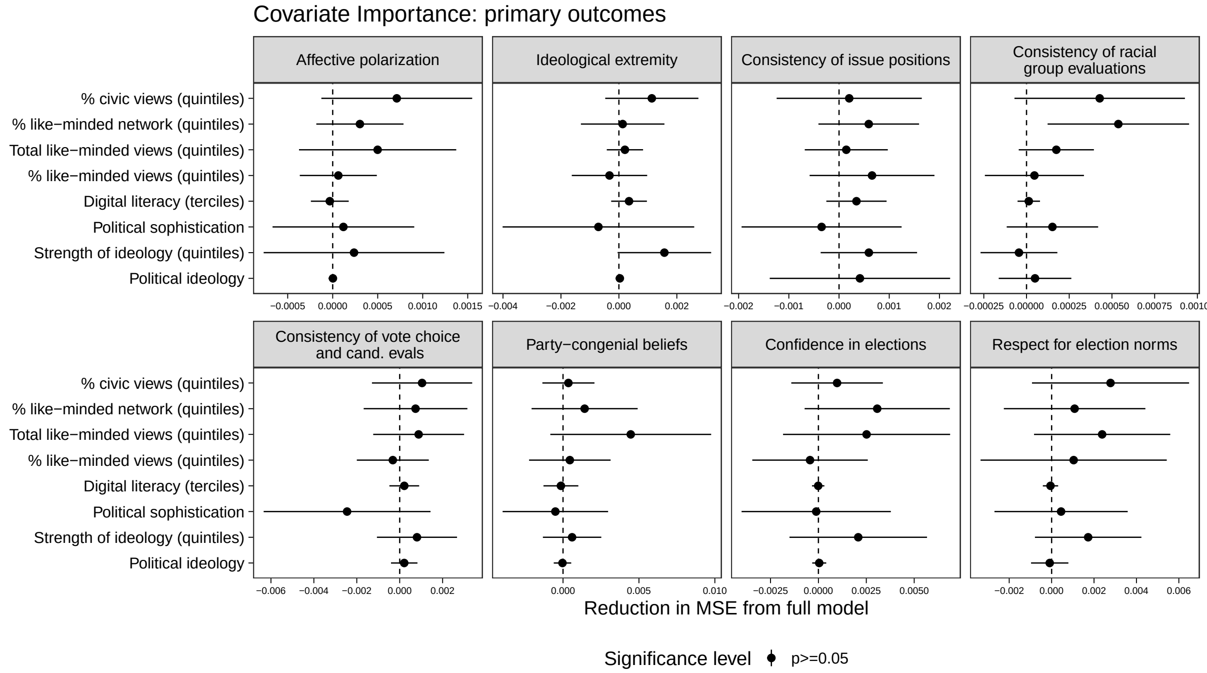

Estimates were constructed using the DR-Learner approach (*57–59*). We use the VIMP method for estimation of variable importance assuming model linearity (*69*). Statistical tests control the false discovery rate (*55*). Political ideology and sophistication were treated as discrete variables; other moderators were treated as continuous. Survey outcome measures are standardized scales averaged across surveys conducted November 4–18, 2020 and/or December 9–23, 2020. Error bars indicate 95% confidence intervals. See Table 46 for sample size included in each analysis. After adjusting for multiple comparisons, all p-values equal 1.

Figure 63: Variable importance estimates of heterogeneous treatment effects for research questions

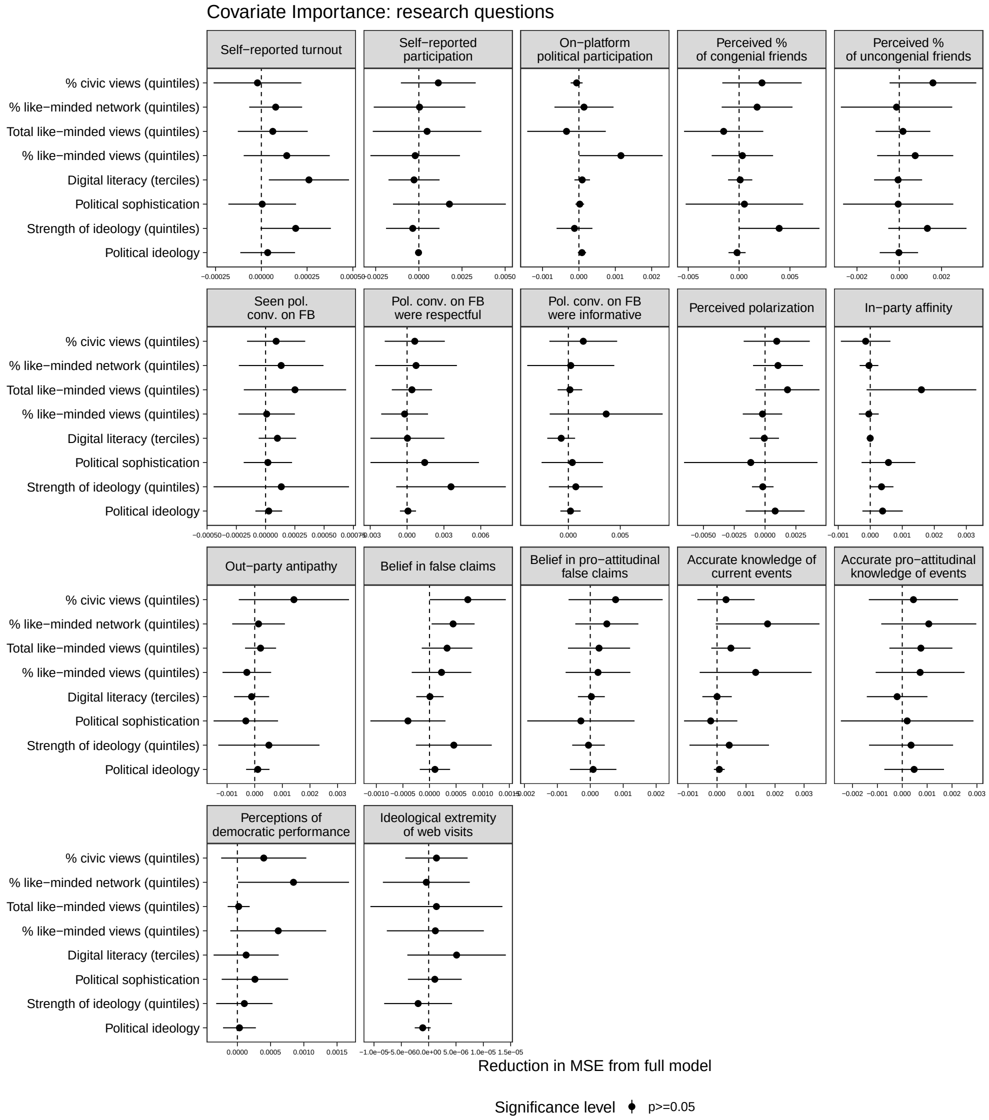

Estimates were constructed using the DR-Learner approach (*57–59*). We use the VIMP method for estimation of variable importance assuming model linearity (*69*). Statistical tests control the false discovery rate (*55*). Political ideology and sophistication were treated as discrete variables; other moderators were treated as continuous. Survey outcome measures are standardized scales averaged across surveys conducted November 4–18, 2020 and/or December 9–23, 2020. Error bars indicate 95% confidence intervals. See Table 47 for sample size included in each analysis. After adjusting for multiple comparisons, all p-values equal 1.

3.9.5 HTE analysis for exploratory moderators

Our pre-registration plan stated that we would conduct exploratory analyses to examine how treatment effects vary both by subgroups generated from survey measures (e.g., age, gender, race/ethnicity, in particular among African Americans and Hispanics), behavioral measures (e.g., Facebook tenure), and participant's location in a battleground state as determined by the Cook Report as of July 23, 2020. The tables reported below provide little evidence of heterogeneous effects of the treatment on exposure by source type. We therefore did not estimate comparable models testing for heterogeneous effects by these moderators on attitudes.

Table S57–S60 below show the treatment effect on exposure to content by like-minded, cross-cutting, and neither like-minded nor cross-cutting sources and for sources whose leaning cannot be estimated, respectively, for each subgroup.

Table 52: Exposure to content from like-minded sources on Facebook

Metric	Control	Treatment	N	Diff	p(diff!=0)
Age: 18-29	49.5	32.0	4214	-17.5	p=0.00
Age: 30-44	51.6	35.2	9925	-16.4	p=0.00
Age: 45-65	57.2	38.8	7539	-18.4	p=0.00
Age: 65+	61.4	42.2	1534	-19.2	p=0.00
Gender: female	52.3	34.2	13302	-18.2	p=0.00
Gender: male	55.3	38.8	9727	-16.5	p=0.00
Gender: other	66.7	49.4	183	-17.3	p=0.00
Race/ethnicity: non-Hispanic white	53.8	36.8	17021	-17.0	p=0.00
Race/ethnicity: non-Hispanic Black	62.0	41.0	1559	-21.0	p=0.00
Race/ethnicity: Hispanic	48.2	30.5	2786	-17.7	p=0.00
Race/ethnicity: other	53.8	36.3	1846	-17.6	p=0.00
FB tenure: 0-8 years	50.5	34.6	3241	-16.0	p=0.00
FB tenure: 8-12 years	54.2	36.7	8671	-17.5	p=0.00
FB tenure: 12+ years	54.6	36.5	9596	-18.1	p=0.00
Inventory: low	51.8	39.5	7629	-12.3	p=0.00
Inventory: mid	55.0	36.9	7340	-18.0	p=0.00
Inventory: high	54.3	32.5	8243	-21.7	p=0.00
In swing state	52.3	35.7	7943	-16.7	p=0.00
Not in swing state	54.4	36.5	15269	-17.9	p=0.00

165 observations (0.71%) dropped by listwise deletion.The last column reports the unadjusted p-value from a two-sided test of the hypothesis of no difference between treatment and control groups on each metric, computed using the baseline OLS model (see Section S1.5.1).

Table 56 presents the results of a series of OLS regressions where subgroup indicators were interacted with treatment status.

Table 53: Exposure to content from cross-cutting sources on Facebook

Metric	Control	Treatment	N	Diff	p(diff!=0)
Age: 18-29	21.1	27.4	4214	6.3	p=0.00
Age: 30-44	21.0	27.6	9925	6.6	p=0.00
Age: 45-65	20.1	28.2	7539	8.1	p=0.00
Age: 65+	20.1	29.9	1534	9.8	p=0.00
Gender: female	21.3	28.9	13302	7.5	p=0.00
Gender: male	20.0	26.8	9727	6.9	p=0.00
Gender: other	12.0	18.5	183	6.5	p=0.06
Race/ethnicity: non-Hispanic white	21.5	28.8	17021	7.3	p=0.00
Race/ethnicity: non-Hispanic Black	17.0	25.8	1559	8.8	p=0.00
Race/ethnicity: Hispanic	18.9	25.4	2786	6.5	p=0.00
Race/ethnicity: other	18.4	25.2	1846	6.8	p=0.00
FB tenure: 0-8 years	21.4	26.7	3241	5.3	p=0.00
FB tenure: 8-12 years	20.8	28.0	8671	7.2	p=0.00
FB tenure: 12+ years	20.3	28.7	9596	8.4	p=0.00
Inventory: low	22.2	27.3	7629	5.1	p=0.00
Inventory: mid	20.1	28.1	7340	8.0	p=0.00
Inventory: high	19.8	28.4	8243	8.6	p=0.00
In swing state	22.0	29.0	7943	7.1	p=0.00
Not in swing state	20.0	27.4	15269	7.4	p=0.00

165 observations (0.71%) dropped by listwise deletion.The last column reports the unadjusted p-value from a two-sided test of the hypothesis of no difference between treatment and control groups on each metric, computed using the baseline OLS model (see Section S1.5.1).

In addition, we conduct exploratory analyses testing for heterogeneous treatment effects for age and years since joining Facebook, two other variables that could moderate the effect of the treatment if greater prior Facebook use or more cumulative exposure to polarizing content makes people less sensitive to variation in like-minded source exposure.

We partition respondents by years of age using the survey options provided in the survey: 18–29, 30–44, 45–64, 65+. We partition respondents by years since joining Facebook (i.e., account age) into quintiles where 1 indicates the quintile of respondents with the newest accounts (38 days–9.7 years) and 5 indicates the quintile with the oldest accounts (13.1 years or more). Because these analyses are exploratory, we apply a more stringent sharpened FDR adjustment (*55*). Our preregistered heterogeneous treatment effects analysis adjusts for the number of main effects estimated on the outcomes for our primary hypotheses as well as the number of subgroup effects estimated for those outcomes. In this analysis, we incorporate the new set of subgroup effects into the total set of tests for which we wish to control the false discovery rate. After this adjustment, none of the estimated subgroup effects are statistically

Table 54: Exposure to content from sources that are neither cross-cutting nor like-minded on Facebook

Metric	Control	Treatment	N	Diff	p(diff!=0)
Age: 18-29	26.1	35.8	4214	9.6	p=0.00
Age: 30-44	24.7	33.4	9925	8.7	p=0.00
Age: 45-65	19.4	28.3	7539	8.9	p=0.00
Age: 65+	15.2	22.1	1534	6.9	p=0.00
Gender: female	23.8	33.3	13302	9.6	p=0.00
Gender: male	21.1	28.9	9727	7.9	p=0.00
Gender: other	16.9	26.5	183	9.6	p=0.00
Race/ethnicity: non-Hispanic white	22.4	31.2	17021	8.8	p=0.00
Race/ethnicity: non-Hispanic Black	18.3	28.6	1559	10.3	p=0.00
Race/ethnicity: Hispanic	26.0	34.7	2786	8.7	p=0.00
Race/ethnicity: other	22.5	31.2	1846	8.7	p=0.00
FB tenure: 0-8 years	24.2	32.7	3241	8.5	p=0.00
FB tenure: 8-12 years	22.2	31.3	8671	9.1	p=0.00
FB tenure: 12+ years	22.1	30.7	9596	8.6	p=0.00
Inventory: low	22.5	28.7	7629	6.2	p=0.00
Inventory: mid	22.2	30.9	7340	8.7	p=0.00
Inventory: high	23.0	34.5	8243	11.5	p=0.00
In swing state	22.7	31.1	7943	8.4	p=0.00
Not in swing state	22.5	31.6	15269	9.1	p=0.00

165 observations (0.71%) dropped by listwise deletion.The last column reports the unadjusted p-value from a two-sided test of the hypothesis of no difference between treatment and control groups on each metric, computed using the baseline OLS model (see Section S1.5.1).

significant. The results are provided below in Figures 64 and 65.

Table 55: Exposure to content from sources without audience ideology prediction on Facebook

Metric	Control	Treatment	N	Diff	p(diff!=0)
Age: 18-29	3.2	4.8	4214	1.6	p=0.00
Age: 30-44	2.8	3.8	9925	1.0	p=0.00
Age: 45-65	3.3	4.7	7539	1.4	p=0.00
Age: 65+	3.3	5.8	1534	2.5	p=0.00
Gender: female	2.6	3.6	13302	1.0	p=0.00
Gender: male	3.7	5.4	9727	1.8	p=0.00
Gender: other	4.4	5.6	183	1.2	p=0.31
Race/ethnicity: non-Hispanic white	2.3	3.2	17021	1.0	p=0.00
Race/ethnicity: non-Hispanic Black	2.7	4.6	1559	1.9	p=0.00
Race/ethnicity: Hispanic	6.9	9.4	2786	2.5	p=0.00
Race/ethnicity: other	5.3	7.4	1846	2.1	p=0.00
FB tenure: 0-8 years	3.9	6.0	3241	2.2	p=0.00
FB tenure: 8-12 years	2.8	4.0	8671	1.2	p=0.00
FB tenure: 12+ years	3.0	4.1	9596	1.1	p=0.00
Inventory: low	3.5	4.6	7629	1.1	p=0.00
Inventory: mid	2.8	4.1	7340	1.3	p=0.00
Inventory: high	2.9	4.6	8243	1.6	p=0.00
In swing state	3.0	4.2	7943	1.1	p=0.00
Not in swing state	3.1	4.5	15269	1.4	p=0.00

165 observations (0.71%) dropped by listwise deletion.The last column reports the unadjusted p-value from a two-sided test of the hypothesis of no difference between treatment and control groups on each metric, computed using the baseline OLS model (see Section S1.5.1).

Table 56: OLS regressions predicting source exposure on Facebook by ideological alignment

	Like-minded	Cross-cutting	No ideology	Neither like-minded nor cross-cutting
Treatment	−0.12 (0.01)<br>p = 0.00***	0.03 (0.01)<br>p = 0.0003***	0.01 (0.004)<br>p = 0.02**	0.08 (0.01)<br>p = 0.00***
Age: 30-44	0.003 (0.01)<br>p = 0.57	0.002 (0.004)<br>p = 0.56	−0.0001 (0.002)<br>p = 0.97	−0.01 (0.003)<br>p = 0.11
Age: 45-65	0.06 (0.01)<br>p = 0.00***	−0.01 (0.004)<br>p = 0.04**	0.01 (0.002)<br>p = 0.0001***	−0.06 (0.003)<br>p = 0.00***
Age: 65+	0.11 (0.01)<br>p = 0.00***	−0.01 (0.01)<br>p = 0.06*	0.01 (0.003)<br>p = 0.0004***	−0.10 (0.01)<br>p = 0.00***
Gender: Male	0.03 (0.004)<br>p = 0.00***	−0.02 (0.003)<br>p = 0.00***	0.01 (0.001)<br>p = 0.00***	−0.02 (0.002)<br>p = 0.00***
Gender: Other	0.17 (0.02)<br>p = 0.00***	−0.09 (0.02)<br>p = 0.00***	0.01 (0.01)<br>p = 0.05**	−0.09 (0.01)<br>p = 0.00***
Race: Black, non-Hispanic	0.09 (0.01)<br>p = 0.00***	−0.05 (0.01)<br>p = 0.00***	0.01 (0.002)<br>p = 0.003***	−0.05 (0.005)<br>p = 0.00***
Race: Hispanic	−0.04 (0.01)<br>p = 0.00***	−0.03 (0.005)<br>p = 0.00***	0.05 (0.002)<br>p = 0.00***	0.02 (0.004)<br>p = 0.0000***
Race: AAPI/Other	0.01 (0.01)<br>p = 0.35	−0.03 (0.01)<br>p = 0.00***	0.03 (0.002)<br>p = 0.00***	−0.002 (0.004)<br>p = 0.59
Tenure: 8-12 years	0.03 (0.01)<br>p = 0.0000***	−0.01 (0.004)<br>p = 0.19	−0.01 (0.002)<br>p = 0.0000***	−0.01 (0.003)<br>p = 0.0005***
Tenure: 12+ years	0.05 (0.01)<br>p = 0.00***	−0.02 (0.004)<br>p = 0.0004***	−0.004 (0.002)<br>p = 0.04**	−0.03 (0.004)<br>p = 0.00***
Inventory: mid	0.03 (0.004)<br>p = 0.00***	−0.02 (0.004)<br>p = 0.00***	−0.01 (0.001)<br>p = 0.0001***	−0.01 (0.003)<br>p = 0.08*
Inventory: high	0.03 (0.004)<br>p = 0.00***	−0.03 (0.004)<br>p = 0.00***	−0.005 (0.001)<br>p = 0.002***	0.0004 (0.003)<br>p = 0.89
In swing state	−0.02 (0.004)<br>p = 0.0000***	0.02 (0.003)<br>p = 0.00***	−0.002 (0.001)<br>p = 0.07*	0.002 (0.002)<br>p = 0.31
Treat × Age: 30-44	0.02 (0.01)<br>p = 0.03**	−0.01 (0.01)<br>p = 0.23	−0.004 (0.003)<br>p = 0.19	−0.01 (0.01)<br>p = 0.19
Treat × Age: 45-65	−0.001 (0.01)<br>p = 0.91	0.01 (0.01)<br>p = 0.26	0.001 (0.003)<br>p = 0.85	−0.01 (0.01)<br>p = 0.19
Treat × Age: 65+	−0.01 (0.01)<br>p = 0.42	0.03 (0.01)<br>p = 0.03**	0.01 (0.005)<br>p = 0.02**	−0.03 (0.01)<br>p = 0.01***
Treat × Gender: Male	−0.002 (0.01)<br>p = 0.74	−0.001 (0.01)<br>p = 0.91	0.01 (0.002)<br>p = 0.001***	−0.004 (0.004)<br>p = 0.33
Treat × Gender: Other	−0.003 (0.04)<br>p = 0.93	−0.02 (0.03)<br>p = 0.59	0.01 (0.01)<br>p = 0.63	0.01 (0.02)<br>p = 0.56
Treat × Race: Black, non-Hispanic	−0.03 (0.01)<br>p = 0.03**	0.01 (0.01)<br>p = 0.22	0.01 (0.004)<br>p = 0.05**	0.01 (0.01)<br>p = 0.39
Treat × Race: Hispanic	−0.002 (0.01)<br>p = 0.85	−0.004 (0.01)<br>p = 0.59	0.01 (0.003)<br>p = 0.0000***	−0.01 (0.01)<br>p = 0.19
Treat × Race: AAPI/Other	−0.004 (0.01)<br>p = 0.71	0.0001 (0.01)<br>p = 1.00	0.01 (0.004)<br>p = 0.002***	−0.01 (0.01)<br>p = 0.30
Treat × Tenure: 8-12 years	−0.004 (0.01)<br>p = 0.71	0.01 (0.01)<br>p = 0.09*	−0.01 (0.003)<br>p = 0.02**	−0.002 (0.01)<br>p = 0.76
Treat × Tenure: 12+ years	−0.02 (0.01)<br>p = 0.04**	0.03 (0.01)<br>p = 0.0001***	−0.01 (0.003)<br>p = 0.04**	−0.004 (0.01)<br>p = 0.52
Treat × Inventory: mid	−0.06 (0.01)<br>p = 0.00***	0.03 (0.01)<br>p = 0.0001***	0.01 (0.003)<br>p = 0.04**	0.02 (0.01)<br>p = 0.0000***
Treat × Inventory: high	−0.09 (0.01)<br>p = 0.00***	0.03 (0.01)<br>p = 0.0000***	0.01 (0.003)<br>p = 0.002***	0.05 (0.01)<br>p = 0.00***
Treat × In swing state	0.01 (0.01)<br>p = 0.03**	−0.004 (0.01)<br>p = 0.50	−0.002 (0.002)<br>p = 0.37	−0.01 (0.004)<br>p = 0.04**
Constant	0.45 (0.01)<br>p = 0.00***	0.25 (0.01)<br>p = 0.00***	0.02 (0.002)<br>p = 0.00***	0.28 (0.004)<br>p = 0.00***
Observations	21,610	21,610	21,610	21,610
R^2	0.17	0.06	0.08	0.15

OLS regression coefficients with standard errors in parentheses; $^{*}p$ <0.1; $^{**}p$ <0.05; $^{***}p$ <0.01 (two-sided t-tests; unadjusted).

Ref. categories: 18–29 years old, female, white non-Hispanic, Facebook tenure 0–8 years, low tercile of Feed inventory.

Figure 64: Treatment effects on outcomes for primary hypotheses by participant age group

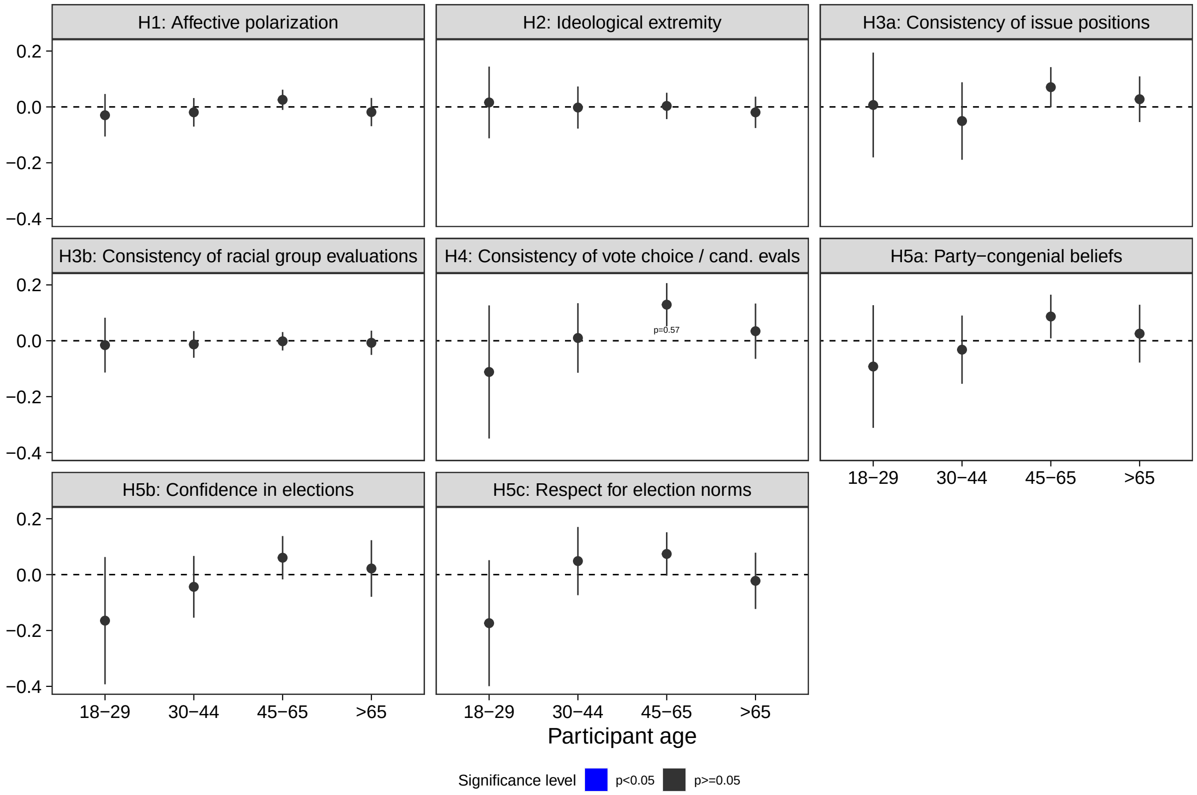

Population conditional average treatment effects (including 95% confidence intervals) of reducing exposure to like-minded sources in the Facebook Feed from September 24–December 23, 2020. The figure shows OLS estimate of population conditional average treatment effects using HC2 robust standard errors and survey weights. Tests of significance consider the two-tailed t-statistic for the test of whether the subgroup treatment effect is different from zero after controlling the false discovery rate (*55*). The sample of users is split into four subgroups defined by participants' self-reported age group: 18–29, 30–44, 45–64, and 65+. Survey outcome measures are standardized scales averaged across surveys conducted November 4–18, 2020 and/or December 9–23, 2020. After adjusting for multiple comparisons, all p-values equal 1. Sample size for each model is available in Table 46.

Figure 65: Treatment effects on outcomes for primary hypotheses by quintile of user account age

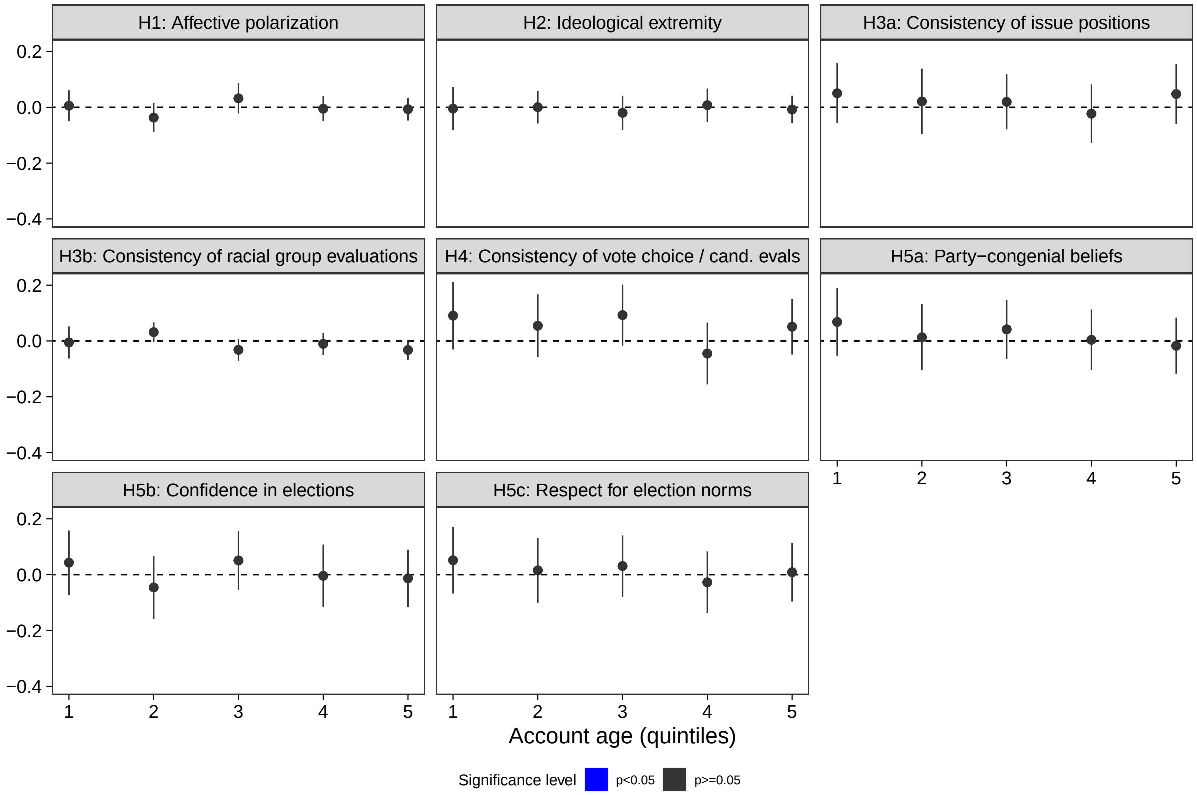

Population conditional average treatment effects (including 95% confidence intervals) of reducing exposure to like-minded sources in the Facebook Feed from September 24–December 23, 2020. The figure shows OLS estimate of population conditional average treatment effects using HC2 robust standard errors and survey weights. Tests of significance consider the two-tailed t-statistic for the test of whether the subgroup treatment effect is different from zero after controlling the false discovery rate (*55*). The sample of users is split into five quintiles of user account age (i.e., how long since they created their Facebook account). The quintile cutoffs are 38 days (the sample minimum), 9.7 years, 11.4 years, 12 years, 13.1 years, and 16.6 years (the sample maximum). Survey outcome measures are standardized scales averaged across surveys conducted November 4–18, 2020 and/or December 9–23, 2020. Unless otherwise reported in the figure, all p-values equal 1 after adjusting for multiple comparisons. Sample size for each model is available in Table 46.

3.10 Exploratory analysis: Robustness check for (0.4, 0.6) exclusion

Below we report exploratory results for our preregistered primary hypotheses and research questions excluding respondents with political leanings in the range of (0.4, 0.6) on a 0–1 scale. The results are consistent with those in the main text.

Table 57: Treatment effects on outcomes for primary hypotheses

Hypothesis	Est	ATE	95% CI	SE	N	p	Adj. p
H1: Affective polarization	PATE	-0.010	[-0.039, 0.018]	0.015	19162	0.480	1.000
	SATE	-0.021	[-0.037, -0.005]	0.008	19162	0.009	0.074
H2: Ideological extremity	PATE	-0.002	[-0.037, 0.032]	0.018	18305	0.892	1.000
	SATE	-0.008	[-0.028, 0.012]	0.010	18305	0.447	1.000
H3a: Consistency of issue positions	PATE	0.009	[-0.048, 0.066]	0.029	18311	0.765	1.000
	SATE	-0.003	[-0.032, 0.026]	0.015	18311	0.828	1.000
H3b: Consistency of racial group evaluations	PATE	-0.008	[-0.032, 0.016]	0.012	18313	0.517	1.000
	SATE	0.002	[-0.011, 0.015]	0.007	18313	0.729	1.000
H4a: Consistency of vote choice / cand. evals	PATE	0.046	[-0.016, 0.107]	0.031	18310	0.145	1.000
	SATE	0.006	[-0.023, 0.036]	0.015	18310	0.673	1.000
H5a: Party-congenial beliefs	PATE	0.015	[-0.046, 0.075]	0.031	17700	0.641	1.000
	SATE	-0.003	[-0.034, 0.028]	0.016	17700	0.830	1.000
H5b: Confidence in elections	PATE	-0.007	[-0.067, 0.052]	0.031	17700	0.811	1.000
	SATE	-0.012	[-0.043, 0.018]	0.016	17700	0.439	1.000
H5c: Respect for election norms	PATE	0.006	[-0.055, 0.068]	0.031	17650	0.839	1.000
	SATE	-0.011	[-0.041, 0.019]	0.015	17650	0.465	1.000

Estimates based on baseline OLS model (see Section S1.5.1). The last two columns report unadjusted and adjusted p-values from a two-sided t-test of the null hypothesis that each coefficient equals zero.

Figure 66: Treatment effects on outcomes for primary hypotheses

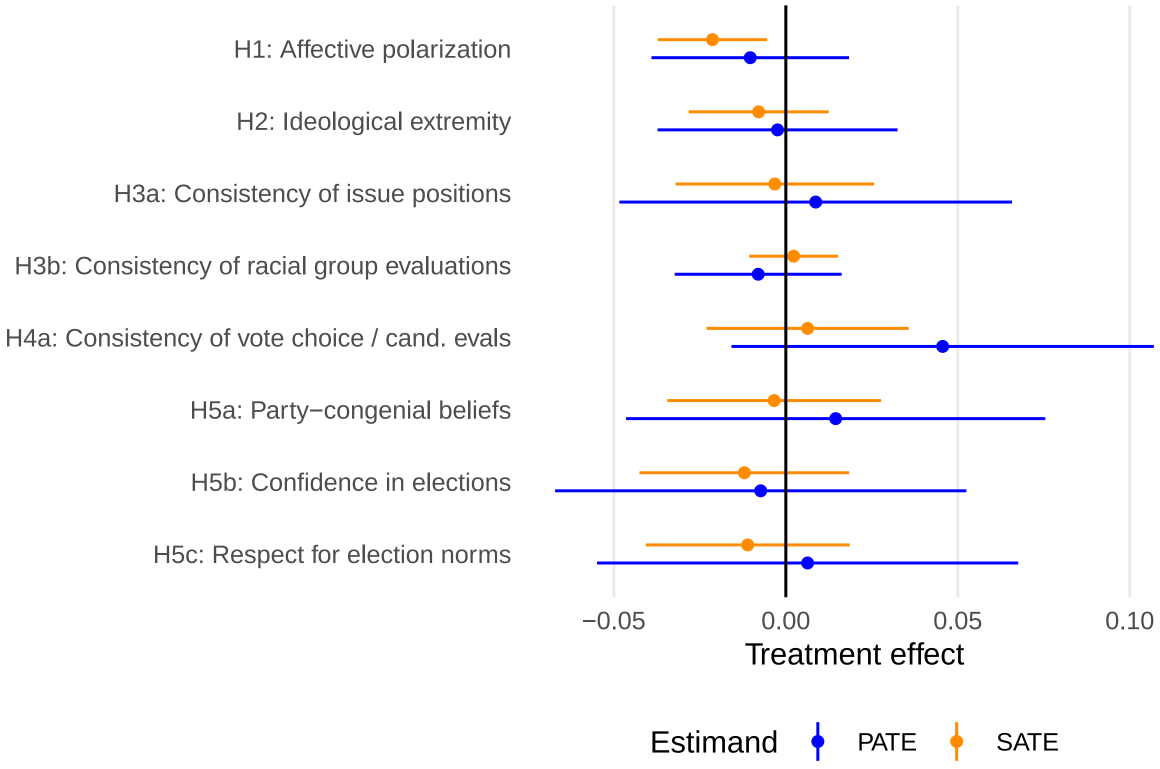

Average treatment effects of reducing exposure to like-minded sources in the Facebook Feed from September 24–December 23, 2020. OLS estimates of population average treatment effect using survey weights and HC2 robust standard errors. Survey outcome measures are standardized scales averaged across surveys conducted November 4–18, 2020 and/or December 9–23, 2020. Respondents with political leanings in the range of (0.4, 0.6) on a 0–1 scale are excluded. Sample size and p-values for each estimate are reported in Table 57.

Table 58: Treatment effects on outcomes for research questions

Hypothesis	Est	ATE	95% CI	SE	N	p	Adj. p
RQ1a: Validated turnout	PATE	-0.085	[-0.174, 0.004]	0.045	9017	0.061	1.000
	SATE	-0.030	[-0.075, 0.014]	0.023	9017	0.183	0.338
RQ1b: Self-reported turnout	PATE	-0.038	[-0.097, 0.021]	0.030	19170	0.208	1.000
	SATE	-0.026	[-0.054, 0.003]	0.014	19170	0.076	0.151
RQ1c: Self-reported participation	PATE	-0.005	[-0.040, 0.030]	0.018	18313	0.775	1.000
	SATE	-0.010	[-0.032, 0.012]	0.011	18313	0.370	0.487
RQ1d: On-platform political participation (Dim 1)	PATE	-0.001	[-0.016, 0.013]	0.007	20225	0.844	1.000
	SATE	-0.020	[-0.039, -0.002]	0.009	20225	0.033	0.084
RQ2a: perceived % of congenial friends	PATE	-0.013	[-0.063, 0.036]	0.025	18234	0.591	1.000
	SATE	-0.033	[-0.063, -0.003]	0.015	18234	0.031	0.084
RQ2b: perceived % of uncongenial friends	PATE	0.007	[-0.047, 0.061]	0.028	18233	0.788	1.000
	SATE	0.038	[0.007, 0.069]	0.016	18233	0.016	0.074
RQ2c: Seen pol. conv. on FB	PATE	0.023	[-0.043, 0.088]	0.033	18455	0.495	1.000
	SATE	-0.014	[-0.045, 0.017]	0.016	18455	0.387	0.487
RQ2d: Pol. conv. on FB were respectful	PATE	-0.038	[-0.091, 0.015]	0.027	16601	0.161	1.000
	SATE	-0.039	[-0.072, -0.007]	0.016	16601	0.017	0.074
RQ2e: Pol. conv. on FB were informative	PATE	-0.054	[-0.104, -0.004]	0.026	16603	0.035	1.000
	SATE	-0.054	[-0.085, -0.022]	0.016	16603	0.001	0.008
RQ3: Perceived polarization	PATE	-0.002	[-0.045, 0.041]	0.022	18310	0.913	1.000
	SATE	0.007	[-0.017, 0.031]	0.012	18310	0.582	0.605
RQ4a: In-party affinity	PATE	-0.017	[-0.050, 0.017]	0.017	19169	0.326	1.000
	SATE	-0.040	[-0.059, -0.021]	0.010	19169	0.000	0.001
RQ4c: Out-party antipathy	PATE	-0.010	[-0.044, 0.025]	0.018	19163	0.591	1.000
	SATE	-0.008	[-0.028, 0.012]	0.010	19163	0.424	0.500
RQ5a: Belief in false claims	PATE	-0.007	[-0.046, 0.032]	0.020	19169	0.726	1.000
	SATE	0.002	[-0.020, 0.025]	0.012	19169	0.834	0.773
RQ5b: Belief in pro-attitudinal false claims	PATE	-0.002	[-0.046, 0.041]	0.022	19169	0.910	1.000
	SATE	-0.013	[-0.040, 0.013]	0.013	19169	0.316	0.463
RQ5c: Accurate knowledge of current events	PATE	-0.001	[-0.041, 0.039]	0.020	16795	0.955	1.000
	SATE	-0.015	[-0.039, 0.009]	0.012	16795	0.219	0.364
RQ5d: Accurate pro-attitudinal knowledge of events	PATE	-0.015	[-0.065, 0.035]	0.025	16781	0.555	1.000
	SATE	-0.010	[-0.037, 0.018]	0.014	16781	0.499	0.523
RQ6: Perceptions of democratic performance	PATE	-0.028	[-0.070, 0.014]	0.021	19170	0.187	1.000
	SATE	-0.009	[-0.034, 0.016]	0.013	19170	0.470	0.523
RQ7: Ideological extremity of web visits	PATE	-0.011	[-0.129, 0.107]	0.060	2935	0.856	1.000
	SATE	-0.042	[-0.112, 0.028]	0.036	2935	0.242	0.364

Estimates based on baseline OLS model (see Section S1.5.1). The last two columns report unadjusted and adjusted p-values from a two-sided t-test of the null hypothesis that each coefficient equals zero.

Figure 67: Treatment effects on outcomes for research questions

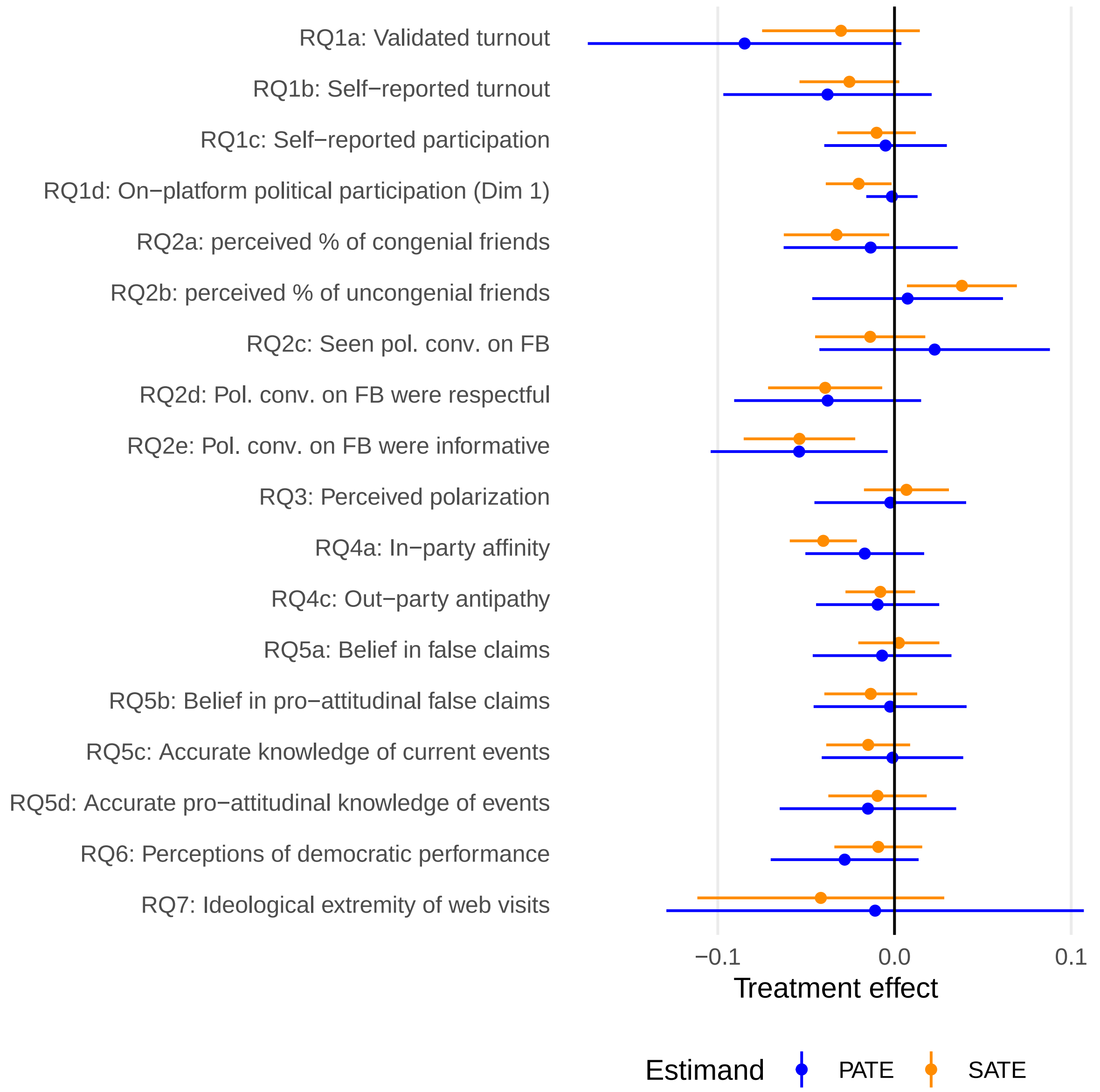

Average treatment effects of reducing exposure to like-minded sources in the Facebook Feed from September 24–December 23, 2020. The figure shows OLS estimates of sample average treatment effects as well as population average treatment effect using survey weights and HC2 robust standard errors. Engagement outcome measures were measured using Feed behavior by participants. Survey outcome measures are standardized scales averaged across surveys conducted November 4–18, 2020 and/or December 9–23, 2020, unless indicated otherwise. Respondents with political leanings in the range of (0.4, 0.6) on a 0–1 scale are excluded. Sample size and p-values for each estimate are reported in Table 58.

3.11 Exploratory analysis: Treatment effects on self-reported media use

Table 59 presents the results of an exploratory analysis examining the effect of the treatment on self-reported media use. This analysis was intended to test if respondents in the treatment group consumed more news from like-minded sources elsewhere (i.e., not on Facebook) in response to treatment. The outcome measure for this analysis was the mean of self-reported media consumption frequency on a four-point scale (4=every day, 1=never for outlets that we categorized as like-minded based on our classifier estimate of their political leaning; these scores were reverse-coded for outlets we classified as counter-attitudinal). Network news, MSNBC, CNN, and NPR were classified as like-minded for participants classified as left-leaning and Fox, Newsmax, and talk radio were classified as like-minded for participants classified as right-leaning. We computed mean self-reported consumption by outlet type across waves 4 and 5 and then computed the mean of those outlet-level values for each respondent. The resulting data provide no measurable evidence of greater pro-attitudinal media consumption among participants.

Table 59: Treatment effects on self-reported media consumption

	Est	ATE	95% CI	SE	N	p
Self-reported news consumption score	PATE	0.014	[-0.034, 0.061]	0.024	22143	0.572
	SATE	-0.015	[-0.041, 0.011]	0.013	22143	0.255

3.12 Exploratory analysis: Equivalence bounds for treatment effect estimates

Tables 60 to 64 display a series of exploratory equivalence confidence intervals that we estimated for all main results reported in Supplementary Information, sections 3.3, 3.4, and 3.5. Tables 65 and 66 display the equivalence confidence intervals for the robustness check report in Supplementary Information, section 3.10, which demonstrates the robustness of our results after excluding respondents with political leanings in the range of (0.4, 0.6). Finally, Table 67 displays the equivalence confidence intervals for the results reported in Table 59.

These equivalence confidence intervals indicate the lower and upper bound values for which we can reject the null hypothesis of no difference with the estimated effect, which implies that we can rule out effect sizes below or above these bounds.

Table 60: Equivalence confidence intervals for treatment effects on outcomes for primary hypotheses

Hypothesis	Est	Equivalence CI
H1: Affective polarization	PATE	[-0.030, 0.022]
	SATE	[-0.032, -0.002]
H2: Ideological extremity	PATE	[-0.037, 0.028]
	SATE	[-0.032, 0.007]
H3a: Consistency of issue positions	PATE	[-0.028, 0.075]
	SATE	[-0.031, 0.024]
H3b: Consistency of racial group evaluations	PATE	[-0.031, 0.016]
	SATE	[-0.013, 0.013]
H4a: Consistency of vote choice / cand. evals	PATE	[0.000, 0.110]
	SATE	[-0.023, 0.034]
H5a: Party-congenial beliefs	PATE	[-0.029, 0.082]
	SATE	[-0.032, 0.027]
H5b: Confidence in elections	PATE	[-0.045, 0.063]
	SATE	[-0.038, 0.020]
H5c: Respect for election norms	PATE	[-0.037, 0.074]
	SATE	[-0.043, 0.014]

Table 61: Equivalence confidence intervals for treatment effects on outcomes for research questions

Hypothesis	Est	Equivalence CI
RQ1a: Validated turnout	PATE	[-0.132, 0.029]
	SATE	[-0.073, 0.011]
RQ1b: Self-reported turnout	PATE	[-0.068, 0.040]
	SATE	[-0.048, 0.005]
RQ1c: Self-reported participation	PATE	[-0.033, 0.033]
	SATE	[-0.032, 0.010]
RQ1d: On-platform political participation (Dim 1)	PATE	[-0.016, 0.011]
	SATE	[-0.038, -0.002]
RQ2a: perceived % of congenial friends	PATE	[-0.054, 0.040]
	SATE	[-0.059, -0.002]
RQ2b: perceived % of uncongenial friends	PATE	[-0.049, 0.058]
	SATE	[0.006, 0.066]
RQ2c: Seen pol. conv. on FB	PATE	[-0.044, 0.080]
	SATE	[-0.039, 0.020]
RQ2d: Pol. conv. on FB were respectful	PATE	[-0.066, 0.036]
	SATE	[-0.064, -0.002]
RQ2e: Pol. conv. on FB were informative	PATE	[-0.086, 0.010]
	SATE	[-0.077, -0.017]
RQ3: Perceived polarization	PATE	[-0.045, 0.035]
	SATE	[-0.020, 0.025]
RQ4a: In-party affinity	PATE	[-0.037, 0.025]
	SATE	[-0.053, -0.017]
RQ4c: Out-party antipathy	PATE	[-0.041, 0.023]
	SATE	[-0.028, 0.009]
RQ5a: Belief in false claims	PATE	[-0.050, 0.026]
	SATE	[-0.023, 0.022]
RQ5b: Belief in pro-attitudinal false claims	PATE	[-0.035, 0.047]
	SATE	[-0.036, 0.015]
RQ5c: Accurate knowledge of current events	PATE	[-0.038, 0.039]
	SATE	[-0.032, 0.014]
RQ5d: Accurate pro-attitudinal knowledge of events	PATE	[-0.072, 0.022]
	SATE	[-0.040, 0.014]
RQ6: Perceptions of democratic performance	PATE	[-0.049, 0.031]
	SATE	[-0.032, 0.016]
RQ7: Ideological extremity of web visits	PATE	[-0.142, 0.085]
	SATE	[-0.097, 0.034]

Table 62: Equivalence confidence intervals for treatment effects on additional outcomes

Hypothesis	Est	Equivalence CI
1) Issue polarization	PATE	[-0.021, 0.078]
	SATE	[-0.026, 0.026]
2) Election knowledge	PATE	[-0.017, 0.074]
	SATE	[-0.027, 0.029]
3) News knowledge	PATE	[-0.049, 0.049]
	SATE	[-0.043, 0.011]
4a) #MeToo feeling thermometer	PATE	[-0.033, 0.024]
	SATE	[-0.020, 0.014]
4b) Disagree: 'women interpret innocent remarks as sexist'	PATE	[-0.067, 0.008]
	SATE	[-0.046, 0.001]
4c) Agree: 'sexual harassment widespread problem'	PATE	[-0.020, 0.058]
	SATE	[-0.028, 0.019]
5) Satisfaction with Facebook	PATE	[-0.055, 0.040]
	SATE	[-0.032, 0.025]
6a) Emotion during election: happy	PATE	[-0.041, 0.037]
	SATE	[-0.038, 0.008]
6b) Emotion during election: depressed (W4)	PATE	[-0.030, 0.037]
	SATE	[-0.019, 0.021]
6b) Emotion during election: depressed (W4)	PATE	[-0.030, 0.037]
	SATE	[-0.019, 0.021]
6b) Emotion during election: anxious (W4)	PATE	[-0.027, 0.041]
	SATE	[-0.020, 0.021]
6b) Emotion during election: anxious (W4)	PATE	[-0.027, 0.041]
	SATE	[-0.020, 0.021]

Table 63: Equivalence confidence intervals for treatment effects on additional outcomes

Hypothesis	Est	Equivalence CI
H4b: Consistency of Senate vote choice	PATE	[0.003, 0.162]
	SATE	[-0.045, 0.036]
H4c: Consistency of Gov. vote choice	PATE	[-0.186, 0.117]
	SATE	[-0.111, 0.057]
H4d: Consistency of pres. cand. evaluations (W5)	PATE	[-0.036, 0.062]
	SATE	[-0.036, 0.023]
H5d: Reg. voters were prevented from voting	PATE	[-0.036, 0.065]
	SATE	[-0.039, 0.022]
H5e: ...and that changed election outcome	PATE	[-0.036, 0.065]
	SATE	[-0.039, 0.022]
RQ1d: On-platform political participation (Dim 2)	PATE	[-0.026, 0.023]
	SATE	[-0.031, 0.028]
RQ1d: On-platform political participation (Dim 3)	PATE	[-0.081, 0.032]
	SATE	[-0.029, 0.008]
RQ4b: Own-party ”smart” perceptions	PATE	[-0.043, 0.037]
	SATE	[-0.031, 0.018]
RQ4d: Out-party ”smart” perceptions	PATE	[-0.046, 0.029]
	SATE	[-0.019, 0.028]

Table 64: Equivalence confidence intervals for treatment effects by survey wave

Hypothesis	Est	Equivalence CI
H1: Affective polarization (W4)	PATE	[-0.028, 0.028]
	SATE	[-0.029, 0.003]
H1: Affective polarization (W5)	PATE	[-0.035, 0.022]
	SATE	[-0.034, 0.000]
H4a: Consistency of cand. evals (W4)	PATE	[-0.039, 0.057]
	SATE	[-0.029, 0.028]
H4a: Consistency of cand. evals (W5)	PATE	[-0.036, 0.062]
	SATE	[-0.036, 0.023]
RQ4a: In-party affinity (W4)	PATE	[-0.039, 0.026]
	SATE	[-0.051, -0.012]
RQ4a: In-party affinity (W5)	PATE	[-0.036, 0.034]
	SATE	[-0.048, -0.007]
RQ4c: Out-party antipathy (W4)	PATE	[-0.053, 0.015]
	SATE	[-0.033, 0.007]
RQ4c: Out-party antipathy (W5)	PATE	[-0.030, 0.040]
	SATE	[-0.024, 0.018]
RQ5a: Belief in false claims (W4)	PATE	[-0.039, 0.043]
	SATE	[-0.015, 0.035]
RQ5a: Belief in false claims (W5)	PATE	[-0.063, 0.020]
	SATE	[-0.032, 0.015]
RQ5b: Belief in pro-attitudinal false claims (W4)	PATE	[-0.021, 0.068]
	SATE	[-0.029, 0.027]
RQ5b: Belief in pro-attitudinal false claims (W5)	PATE	[-0.050, 0.033]
	SATE	[-0.040, 0.012]
RQ5c: Accurate knowledge of current events (W4)	PATE	[-0.053, 0.025]
	SATE	[-0.038, 0.009]
RQ5c: Accurate knowledge of current events (W5)	PATE	[-0.019, 0.062]
	SATE	[-0.022, 0.027]
RQ5d: Accurate pro-attitudinal knowledge of events (W4)	PATE	[-0.081, 0.017]
	SATE	[-0.040, 0.016]
RQ5d: Accurate pro-attitudinal knowledge of events (W5)	PATE	[-0.050, 0.042]
	SATE	[-0.031, 0.022]
RQ6: Perceptions of democratic performance (W4)	PATE	[-0.052, 0.027]
	SATE	[-0.026, 0.022]
RQ6: Perceptions of democratic performance (W5)	PATE	[-0.045, 0.044]
	SATE	[-0.036, 0.017]

Table 65: Equivalence confidence intervals for treatment effects on outcomes for primary hypotheses (excludes respondents with political leanings in the range of (0.4, 0.6))

Hypothesis	Est	Equivalence CI
H1: Affective polarization	PATE	[-0.040, 0.019]
	SATE	[-0.038, -0.005]
H2: Ideological extremity	PATE	[-0.038, 0.033]
	SATE	[-0.029, 0.013]
H3a: Consistency of issue positions	PATE	[-0.049, 0.067]
	SATE	[-0.033, 0.026]
H3b: Consistency of racial group evaluations	PATE	[-0.033, 0.017]
	SATE	[-0.012, 0.015]
H4a: Consistency of vote choice / cand. evals	PATE	[-0.017, 0.107]
	SATE	[-0.024, 0.036]
H5a: Party-congenial beliefs	PATE	[-0.047, 0.076]
	SATE	[-0.035, 0.029]
H5b: Confidence in elections	PATE	[-0.068, 0.053]
	SATE	[-0.044, 0.018]
H5c: Respect for election norms	PATE	[-0.056, 0.068]
	SATE	[-0.042, 0.019]

Table 66: Equivalence confidence intervals for treatment effects on outcomes for research questions (excludes respondents with political leanings in the range of (0.4, 0.6))

Hypothesis	Est	Equivalence CI
RQ1a: Validated turnout	PATE	[-0.175, 0.004]
	SATE	[-0.076, 0.015]
RQ1b: Self-reported turnout	PATE	[-0.098, 0.021]
	SATE	[-0.055, 0.003]
RQ1c: Self-reported participation	PATE	[-0.041, 0.030]
	SATE	[-0.033, 0.013]
RQ1d: On-platform political participation (Dim 1)	PATE	[-0.017, 0.014]
	SATE	[-0.040, -0.001]
RQ2a: perceived % of congenial friends	PATE	[-0.064, 0.036]
	SATE	[-0.064, -0.003]
RQ2b: perceived % of uncongenial friends	PATE	[-0.048, 0.061]
	SATE	[0.006, 0.070]
RQ2c: Seen pol. conv. on FB	PATE	[-0.044, 0.088]
	SATE	[-0.046, 0.018]
RQ2d: Pol. conv. on FB were respectful	PATE	[-0.092, 0.015]
	SATE	[-0.073, -0.007]
RQ2e: Pol. conv. on FB were informative	PATE	[-0.105, -0.003]
	SATE	[-0.086, -0.021]
RQ3: Perceived polarization	PATE	[-0.046, 0.041]
	SATE	[-0.018, 0.032]
RQ4a: In-party affinity	PATE	[-0.051, 0.018]
	SATE	[-0.060, -0.021]
RQ4c: Out-party antipathy	PATE	[-0.045, 0.026]
	SATE	[-0.029, 0.012]
RQ5a: Belief in false claims	PATE	[-0.047, 0.033]
	SATE	[-0.021, 0.026]
RQ5b: Belief in pro-attitudinal false claims	PATE	[-0.047, 0.041]
	SATE	[-0.041, 0.013]
RQ5c: Accurate knowledge of current events	PATE	[-0.042, 0.040]
	SATE	[-0.040, 0.009]
RQ5d: Accurate pro-attitudinal knowledge of events	PATE	[-0.066, 0.035]
	SATE	[-0.038, 0.019]
RQ6: Perceptions of democratic performance	PATE	[-0.071, 0.014]
	SATE	[-0.035, 0.016]
RQ7: Ideological extremity of web visits	PATE	[-0.130, 0.108]
	SATE	[-0.113, 0.028]

Table 67: Equivalence confidence intervals for treatment effects on self-reported media consumption

	Est	Equivalence CI
Self-reported news consumption score	PATE	[-0.035, 0.062]
	SATE	[-0.042, 0.012]

4 Supplementary Notes

4.1 Factor loadings of survey scales

As described in more detail in Section 1.4, we preregistered that numerous survey scales would be constructed using exploratory factor analysis with varimax rotation, and that variables that did not clearly load on the same underlying dimension (i.e., the first principal component) would be analyzed separately (https://osf.io/3sjy2). While the pre-registration did not specify thresholds, we registered a clarification (prior to doing any hypothesis testing) that we would select variables that had factor loadings above 0.7 and missingness below 0.15. In addition, we clarified that in cases where a variable did not load on a post-treatment scale, that variable should also be excluded from the pre-treatment scale used as a control, and that in cases where a variable that is part of a scale occurs in only one of the two post-treatment waves, we will use only the subset that is common across both waves. We report the principal components factor loadings for these scales below (excluding loadings below 0.1).

Table 68: H3: Ideologically consistent issue positions and group evaluations: Race (Wave 2)

Variable	Factor loading
Black Lives Matter feeling thermometer (FT-PEOPLEGROUPS-I)	0.839
Fair treatment by police (BLACKWHITE-A)	0.872
Fair treatment in elections (BLACKWHITE-B)	0.865
Fair treatment by doctors (BLACKWHITE-C)	0.871
Fair treatment in hiring (BLACKWHITE-D)	0.876

Table 69: H3: Ideologically consistent issue positions and group evaluations: Race (Wave 4)

Variable	Factor loading
Black Lives Matter feeling thermometer (FT-PEOPLEGROUPS-I)	0.825
Fair treatment by police (BLACKWHITE-A)	0.867
Fair treatment in elections (BLACKWHITE-B)	0.860
Fair treatment by doctors (BLACKWHITE-C)	0.875
Fair treatment in hiring (BLACKWHITE-D)	0.882

Table 70: H4: Ideologically consistent vote choice and candidate evaluations

Variable	Factor loading
Presidential vote (VOTE-POST)	0.945
House vote (VOTEHOUSE)	0.883
Feelings toward congenial pres. candidate (FT-PEOPA/FT-PEOPB)	0.867
Feelings toward congenial pres. candidate (FT-PEOPA/FT-PEOPB)	-0.888

This final scale combines vote choice from both Wave 4 and Wave 5, and feeling thermometers are from Wave 4. See Section 1.4 for more details.

Table 71: H5: Partisan-congenial beliefs about elections

Variable	Factor loading
Who won election (ELECTWIN)	0.771
People voted illegally (IRREG2020B)	0.918
Illegal voting changed election outcome (ILLEGALVOTEEFFECT2020)	0.921

Variables in this scale were recoded based on the political leaning of the respondent. See Section 1.4 for details on the pre-registered recoding procedure.

Table 72: H5: Partisan-congenial views toward elections

Variable	Factor loading
Confidence in election officials (CONFOFFICIALS)	0.894
Votes were counted accurately (COUNTACCURATE)	0.920
Mail votes were counted accurately (MAILACCURATE)	0.920

Variables in this scale were recoded based on the political leaning of the respondent. See Section 1.4 for details on the pre-registered recoding procedure.

Table 73: RQ3: Perceived polarization (Wave 2)

Variable	Factor loading
Ideological difference between supporters of Dems & Reps	0.879
Ideological difference between Dems & Reps running for office	0.850
Ideological difference between Dems & Reps on Facebook	0.842

All variables in this scale were constructed from IDEOLOGYGROUP, which asked respondents to place various groups on a 1-7 ideology scale. See Section 1.4 for details on the pre-registered recoding procedure.

Table 74: RQ3: Perceived polarization (Wave 4)

Variable	Factor loading
Ideological difference between supporters of Dems & Reps	0.877
Ideological difference between Dems & Reps who ran for office	0.841
Ideological difference between Dems & Reps on Facebook	0.834

All variables in this scale were constructed from IDEOLOGYGROUP, which asked respondents to place various groups on a 1-7 ideology scale. See Section 1.4 for details on the pre-registered recoding procedure.

Table 75: RQ4: Own-party affinity (Wave 2)

Variable	Factor loading
Feelings toward supporters of congenial party (FTPEOPLEGROUPS)	0.955
Feelings toward candidate from congenial party (FTPEOPLEGROUPS)	0.955

This scale excludes the “smart” variable since it was not asked in Wave 5.

Table 76: RQ4: (Wave 4)

Variable	Factor loading
Feelings toward supporters of congenial party (FTPEOPLEGROUPS)	0.958
Feelings toward candidate from congenial party (FTPEOPLEGROUPS)	0.958

This scale excludes the “smart” variable since it was not asked in Wave 5.

Table 77: RQ4: Out-party antipathy (Wave 2; initial scale)

Variable	Factor loading
Feelings toward supporters of uncongenial party (FTPEOPLEGROUPS)	0.907
Feelings toward candidate from uncongenial party (FTPEOPLEGROUPS)	0.894
How smart people in uncongenial party are (DEMSMART/REPSMART)	0.785

Table 78: RQ4: Out-party antipathy (Wave 2)

Variable	Factor loading
Feelings toward supporters of uncongenial party (FTPEOPLEGROUPS)	0.940
Feelings toward candidate from uncongenial party (FTPEOPLEGROUPS)	0.940

This scale excludes the “smart” variable since it was not asked in Wave 5.

Table 79: RQ4: Out-party antipathy (Wave 4

Variable	Factor loading
Feelings toward supporters of uncongenial party (FTPEOPLEGROUPS)	0.945
Feelings toward candidate from uncongenial party (FTPEOPLEGROUPS)	0.945

This scale excludes the “smart” variable since it was not asked in Wave 5.

Table 80: RQ1d: Post-treatment measures of on-platform civic engagement (three dimensions)

	Factor loading		
Variable	(1)	(2)	(3)
Clicks on civic posts	0.714	0.157	
Reactions to civic posts	0.784		
Likes on civic posts	0.817		
Shares of civic posts	0.270	0.770	
Comments on civic posts	0.475	0.366	
Indicated interest in going to a civic event	0.142	0.162	
Indicated attending a civic event		0.223	
Views of Voting Information Center	0.281	0.180	
Clicks on Voting Information Center		0.126	0.103
Clicks on Town Hall			0.900
Officials followed via Town Hall			0.252
Officials contacted via Town Hall			0.895
Clicks on petitions	0.180	0.110	
Shares of petitions		0.533	
Donations to civic causes	0.105		
Enabling constituent badge	0.457		
Reactions to posts by politicians	0.688		
Likes of posts by politicians	0.731		
Comments on posts by politicians	0.356	0.196	
Reshares of posts by politicians	0.228	0.724	
Tags or mentions of politicians		0.120	

Variables in this scale measure on-platform behavior. See Section 1.4 for details.

4.2 Coding of on-platform behavior

We analyze the following metrics based on on-platform behavior:

1. Exposure-based metrics

   - These metrics aggregate counts of views on content on Facebook's Feed. A view on a post is counted whenever the post renders in the visible portion of their Web browser or mobile device for more than 250 milliseconds (i.e., a "validated viewport view" or VPV) on Facebook.
   - Unless otherwise noted, other surfaces on Facebook (e.g., Groups Tab or News Tab) are excluded from all exposure metrics since our intervention only manipulated the main platform feed.

2. Engagement-based metrics

   - We consider the following types of engagement: clicks, likes, reactions, comments, and reshares for Facebook. In some analyses we bundle these metrics, depending on how visible the actions are, into *active* (comments, reshares) and *passive*, which we further disaggregate into likes and reactions, and clicks.
   - Clicks are counted once per post and per day if a respondent clicked on any component of a post. This includes clicks on external URLs, clicks to "See More" and expand the text of a post, clicks on the post author's name, click to enlarge a picture, clicks to reshare, as well as clicks to add a like, reaction, comment, or any other type of engagement with the post.
   - The surfaces included in the engagement metrics are identical to those included when measuring exposure in order to be consistent across the two types of metrics. The only exception is reshares, which we aggregate over all surfaces because we are not able to identify the surface from which a user reshared a post due to logging limitations. For this reason, whenever we report engagement rates for reshares (normalized by views) in our analysis, we use the count of views across all surfaces instead of only views on Feed.

3. Production metrics

   - Our metrics of content production refer to the count of new posts created by the respondents in the study. For Facebook, this includes both original posts and reshared posts created on users' Feed, on their Profile, or on a Group. All types of posts are considered here (text posts, URL posts, image posts, photo albums, etc.).

4. Network metrics

- These metrics refer to the connections that respondents make to other users or entities on each platform. For Facebook, we measure connections to other users (i.e., friends), Pages, and groups.
- Our metrics of network exposure are defined based on the actor that is closest to the viewer. In other words, in the case of reshared posts, the connection is defined based on the respondent's relationship to who is resharing a post and not its original content creator. For example, if the respondent sees a post created by a Page and then reshared by one of the respondent's friends, we would count it as friend content.

5. Time spent

   - Our metric of time spent on Facebook is based on Meta's internal measures of the amount of time that each person actively engages with the app or website. This metric includes time spent on all surfaces on Facebook. We report standardized time spent, which was computed by subtracting the average time that U.S. monthly active users spend on the platform, and then divided by the standard deviation of this measure.
   - Our measures of time spent off-platform on other websites are based on the passive tracking data that a subset of our respondents provided (see Supplementary Information, section 4.4). In particular, we measured total web visits (with domain-level granularity only) on desktop/laptop devices, tablets, and smartphones on Windows, Mac, and Android systems.

6. Indices of on-platform political engagement

   - Index of on-platform political engagement, which scales several metrics related to respondents' engagement with political content or civic products on Facebook, and is used as an outcome variable in our analysis, as described in Section 1.4.

7. Ideological alignment

   - We use the US ideology classifier (described in Section 4.3) to determine ideological alignment between study participants and the sources of content (or their audience) they were exposed to or engaged with.
     - We use two-class classification, categorizing respondents as *liberal* if they have a predicted ideology score less than 0.50, or as *conservative* if they have a score greater than or equal to 0.50.
     - We then categorize content sources (e.g., friends, Pages, or groups) as *liberal* if they have a predicted user or audience ideology score less than or equal to 0.40, and as *conservative* if they have a score greater than or equal to 0.60. If their score is between 0.40 and 0.60, we consider it moderate for users (based on their predicted ideology) or ideologically mixed for Pages and groups (based

on the composition of their audiences). (See more details on how user ideology and entity audience ideology are measured in Section 4.3.)

- For reshared posts, this metric is defined based on the predicted (audience) ideology of the entity sharing the post and not the original content creator. For example, if a friend of a liberal study participant shared a Page post, we would consider that post as cross-cutting if that friend were predicted to be conservative.
- For predicted liberal and conservative participants and sources (based on their audiences), content from a friend, Page or group is then considered *like-minded* with respect to a participant if both the participant and source's predicted categories match or *cross-cutting* if they do not. Content from sources that are predicted to be neither liberal nor conservative (i.e., moderate users and entities with ideologically mixed audiences) is considered neither like-minded nor cross-cutting.
- The decision to assign all study participants to a *liberal* or *conservative* category is driven by our attempt to maximize coverage of our metrics that capture exposure to *like-minded* or *cross-cutting* sources. By doing so, we are able to compute these metrics for all respondents in our sample.

Depending on the analysis, we rely on metrics computed using different levels of aggregation:

- Study period: these metrics are based on computing the total count for the period that our intervention was running (2020-09-24 until 2020-12-22; intervention ended 2020-12-23 halfway through the day so metrics are aggregated until end of day on 2020-12-22). In some cases, these metrics are normalized by dividing by a total computed over the same period.
- Pre-treatment period: these metrics are based on computing the total count for a period of 90 days prior to treatment initiation (2020-06-26 to 2020-09-22).
- Static metrics: these metrics are reported as of treatment initiation (2020-09-23).

4.3 Classification and categorization methods

Our analysis relies on classifiers and categorization methods to characterize content and sources. We describe these further below.

4.3.1 Meta classifiers and categorization methods

The following classifiers, concepts, and categorization methods were either developed at Meta or are defined under Facebook's platform policies.

- Civic classifier
  - **Definition:** This classifier predicts whether a given post is related to *politics* (government, elections, politicians, activism, etc.) or *social issues* (major issues that affect a large group of people, such as the economy, inequality, racism, education, immigration, human rights, the environment, etc.).
  - **Usage:** We use the classifications for Facebook posts (links, photos, videos, text) posts that were created, seen, or engaged with by US users during the US 2020 Facebook and Instagram Election Study. We use the classifications for both English- and Spanish-language content. (We sometimes refer to content classified as such as "political" in the main text.)
  - **Performance:**
    * Based on a sample of approximately 10,000 labeled posts, the classifier has 83% precision and 82% recall on English-language Facebook content.
    * Based on a sample of approximately 17,000 labeled posts, the classifier has 81% precision and 85% recall on Spanish-language Facebook content.
- US ideology classifier
  - **Definition:** This classifier predicts adult US active Facebook users' political ideology.
  - **Methodology**
    * The classifier is trained to predict the self-reported ideology of adult US monthly active Facebook users based on their demographics, preferred language, location, and engagement with content, Pages, and groups. It outputs a numeric score ranging from 0 (indicating a user is predicted to be liberal) to 1 (indicating a user predicted to be conservative).
    * The classifier has high coverage — it is able to place up to 95% of adult US monthly active Facebook users on this numerical scale. The classifier also has high week-over-week stability — on average, the weekly scores for individual users have a correlation of 0.96.

    - To further categorize adult US monthly active Facebook users into distinct ideological groups, we discretize the continuous ideology scores as follows: users whose ideology score is less than or equal to 0.5 are classified as *liberal* and otherwise are classified as *conservative*.
    - **Entity audience ideology scores:**
        - We use the user-level ideology scores to generate similar ideology scores for Pages, groups, and web domains. We do so by computing a measure of the ideological composition of their audience: the average predicted ideology of Facebook users engaging with these entities in the last 28 days.
        - This approach places Pages, groups, and web domains on the same 0–1 numeric ideology scale as users. As such, we use the same thresholds for categorizing these entities into ideological groups as specified above, with one exception: we use the term "mixed" rather than "moderate" for the middle category in 3-class ideology, as the scores reflect the ideological composition of entities' audiences and not the ideology of the entities themselves.
- **Usage:**
    - We use the ideological classifications of US monthly active Facebook users 18 years or older who were active during the US 2020 FIES (Facebook Instagram Election Study).
    - We use the audience ideology classifications for all Pages, groups, and domains producing content that US active Facebook users saw or interacted with during the US 2020 FIES.
- **Performance:** We evaluated how well the user-level inferences performed at inferring self-reported ideology by comparing our classifications (as of 2020-09-23) to the survey responses of U.S. 2020 FIES panelists. Precision and recall for each ideological group are as follows:
    - Two-class classification
        - Self-reported liberals: 85% precision and 86% recall
        - Self-reported conservatives: 83% precision and 83% recall
    - Three-class classification
        - Self-reported liberals: 59% precision and 80% recall
        - Self-reported moderates: 52% precision and 21% recall
        - Self-reported conservatives: 56% precision and 78% recall
- We note that the reason precision is lower across all groups and recall is lowest for self-reported moderates in the three-class classification task is that the model tends to predict self-reported moderates to actually be left- or right-leaning. This finding is consistent with external research finding self-reported moderates often have non-centrist ideological positions on various issues (*72*).

- We also evaluated how well the audience ideology measure might approximate the ideological affiliation of the entities themselves, if one exists. We compared the audience ideology scores we computed for the official Facebook Pages of US Members of Congress to a widely-used external measure of those Congress members' ideology derived from their legislative voting history, DW-NOMINATE (*73*), and found a correlation of 0.96 (N = 409).
- As part of the analysis in this paper, we also separately evaluated the performance of this classifier. These analyses, available in 2.1, include confusion matrices that visualize the recall and accuracy of the classifier compared to self-reported political characteristics from our survey data, plots showing the distribution of self-reported ideology within bins of the classifier, and histograms of the distribution of the predicted ideology score within the sample for each subgroup of participants, as defined by their ideology, partisan identification, or Trump approval.

• News classifier

- **Definition:** This binary classifier predicts whether content is about current events, timely information, and follows journalistic standards such as citing sources and having a byline. See `https://www.facebook.com/business/help/22409977271922` for additional information.
- **Usage:** We use the classifier predictions for Facebook posts with a link or video that were created, seen, or engaged with by US active users during the US 2020 FIES. We use the predictions for both English- and Spanish-language content.
- **Performance:** Classification thresholds were chosen to yield 80% recall. Based on a sample of approximately 52,000 labeled links, precision at this threshold is 90% for US English-language news links. Based on a sample of approximately 36,000 labeled links, precision at this threshold is 42% for US Spanish-language news links.

• Topic classifier

- **Definition:** This classifier predicts content categories along 26 different broad topics. The full list of topics is: Animals & Pets; Books & Literature; Business, Finance & Economics; Crime & Tragedy; Education & Learning; Fashion & Style; Children & Parenting; Fitness & Workouts; Food & Drink; Games, Puzzles & Play; Health & Medical; History & Philosophy; Holidays & Celebrations; Home & Garden; Music & Audio; Performing Arts; Politics; Relationships, Friends & Family; Religion & Spirituality; Science & Tech; Social Issues; Sports; Travel & Leisure Activities; TV & Movies; Vehicles & Transportation; Visual Arts, Architecture & Crafts
- **Usage:** We use the classifications for text, video, photo, and link posts that appeared in Facebook Feed and were created, seen, or engaged with by US active users during the US 2020 FIES. We use the predictions for both English- and Spanish-language content.

- **Performance:** Classification thresholds were chosen to yield 80% precision. The recall across all topics at this threshold is 89% in English and 86% in Spanish. The recall for individual topics at this threshold ranges from 49% to 99%, with a median value of 87%.

- Misinformation
  - Misinformation refers to content that is directly rated "false" by one of Meta's independent fact-checking partners, or posts containing text, images, or videos that are matched to such content using matching algorithms. See `https://www.facebook.com/business/help/341102040382165` for a detailed description of how this rating category is defined.
  - Meta's third-party fact-checking partners are certified by the nonpartisan International Fact-Checking Network. A full list of these partners is available at `https://ifcncodeofprinciples.poynter.org/signatories`. In the US, this list includes organizations such as Snopes, Reuters, The Washington Post, Fact Checker, FactCheck.org and PolitiFact. All fact-checks are publicly available on the websites of these organizations and can be reviewed by any external source for accuracy.

4.3.2 Other classifiers and categorization methods

The following classifiers and categorization methods were either proposed by the academic team or adapted from published academic research. We relied primarily on existing performance metrics when available (referenced below in regard to each classifier). The US 2020 academic and Meta researchers did not conduct independent, large-scale evaluations of these classifiers, but where possible hand-labeled a small set of examples and gathered performance estimates based on those samples.

- Content with slur words classifier
  - **Definition:** This classification method is adapted from (*74*), which identifies content containing at least one term sourced from Hatebase and the Racial Slur Database and attempts to reduce false positives introduced by the inclusion of terms with ambiguous meaning. (*74*)'s classification method labels content as falling within any of eight different categories (listed below) as well as a joint category ("content with slur words") that encompasses all of them.
    - List of categories: anti-Asian, anti-Black, anti-Immigrant, anti-Muslim, anti-Semitic, anti-Latinx, homophobic, misogynistic
  - This categorization method aims to capture content that could be perceived as hateful but may not violate Meta's Community Standards or be captured by Meta's existing automated systems.

- **Methodology:** The method developed by (*74*) consists of a dictionary method that was augmented using machine learning methods in order to reduce the false positive rate. First, it identifies any post or comment that contains at least one slur associated with each of the eight categories described above, based on a list sourced from Hatebase and the Racial Slur Database. Second, it applies a text-based classifier trained on a random sample of tweets to attempt to reduce false positives from terms that have multiple meanings, not all of which may be considered a slur (for instance, a benign use of "sneakers" as opposed to its usage as an anti-Black slur).
- We note two limitations of this method, both of which are common to slur-based approaches to hate speech classification. First, the classification method only detects an inherently limited set of slurs. A great deal of hateful content is more nuanced, subtle, and/or complex, making it hard to automatically classify in any instance, but especially hard to do so using the detection of slurs (*75*). Second, in selecting this classification method, we chose to err on the side of false positives (recall) as opposed to false negatives (precision). As a result, some of the content classified as containing one or more slur words may capture ingroup discourse and banter (e.g., use of the n-word among Black communities and use of "bitch" among women), neither of which the false positive filter is designed to filter out. We therefore describe it here as "content with slur words" (a deviation from our preregistration). In general, we acknowledge that a slur-based approach may miss important context and produce both false positives and false negatives (*76*).
- **Usage:** We apply the externally trained classification method to generate predictions for Facebook posts and comments that were created, seen, or engaged with by US users during the US 2020 FIES. This classification method was applied to English-language content only.
- For posts, the classification method is applied to the text of the post, the text contained in attached images, and a transcription of any attached videos. For comments, the classification method is applied to the text of the comment only.
- For reshared posts, a post is classified as containing one or more slur words if the original post or the reshared post (including the text that was added to the reshared post) was classified as containing one or more slur words.
- **Performance:** (*74*) estimate this categorization method has 94% accuracy, 95% precision, and 90% recall for content with a slur. In our data, over 90% of content predicted to contain a slur word using this method falls under the "misogynistic" subcategory.

* Incivility classifier
  - **Definition:** The classifier aims to capture uncivil content, defined as: "Features of discussion that convey an unnecessarily disrespectful tone toward the discussion

forum's participants or its topics, which is including but not limited to: (1) Name-calling, mean-spirited or disparaging words directed at a person or group of people. (2) Aspersion, mean-spirited or disparaging words directed at an idea, plan, policy, or behavior. (3) Vulgarity, using profanity or language that would not be considered proper in professional discourse. (4) Pejorative for speech, disparaging remark about the way in which a person communicates." This approach follows the definitions from (*77*). Note that uncivil language as defined here may not necessarily be threatening or harmful and could be used to emphasize opinions, for example (*78*).

- **Methodology:** The classifier is a regularized logistic regression using unigrams as features that was trained on two datasets: (1) A random sample of 5,000 Reddit comments collected by (*79*) that was annotated by three undergraduate students (inter-coder agreement = 91%); (2) A random sample of 4,000 tweets that was annotated by crowd workers (intercoder agreement $> 80\%$) along with a synthetic set of 16,000 tweets labeled by the Google Perspective API collected by (*80*). The training dataset was complemented with 5 million labels generated using DistillBERT to improve its performance. We applied the externally trained classifier to the Facebook content categories described above, using text-based features and OCR'd text from images. For reshared posts, a post is classified as uncivil if the original post was classified uncivil or if the reshared post was classified as uncivil.
- **Usage:** We use the externally trained classifier to generate predictions for Facebook posts that were created, seen, or engaged with by US users during the US 2020 FIES. This classifier was applied to English-language content only.
- **Performance:**
  * Predictions from this model were estimated by (*79*) to have 85% precision and 72% recall on the Reddit dataset (computed on a test set not used for training) and 89% precision and 70% recall on the Twitter dataset.
  * Based on human annotations conducted by the Meta research team and validated by a subset of the academic researchers on the US 2020 FIES comprising a random sample of 100 Facebook public posts and 100 Facebook public comments, we estimate that the classifiers have 83% precision on Facebook posts and 86% on Facebook comments.
  * Based on a set of synthetic examples available in Jones (2015) from African-American Vernacular English, compared to their Modern Standard English variation, we found that this classifier yields similar predictions, which we take as evidence of adequate calibration.

• Untrustworthy sources

- **Definition**: For purposes of this research project, we define untrustworthy sources based on the number of misinformation "strikes" they have accrued under Meta's

Misinformation Repeat Offender Policy, where a strike may be counted when an entity produces content that is rated false or altered by one of Meta's independent fact-checking partners. We define untrustworthy sources to encompass Pages, groups, and domains on Facebook that have accrued two or more misinformation "strikes" since the MRO program began in 2018 (i.e., "lifetime" strikes).

– **Operationalization**: Due to data retention limitations, we can only estimate the number of strikes accrued by an entity using the number of pieces of content that received a false or altered rating by one of Meta's fact-checking partners. Pages or groups that have posted such content at least twice or domains with two or more URLs rated as "false" by a third-party fact checker are considered untrustworthy sources. In practice, the actual number of strikes an entity accrues does not necessarily equal the number of individual pieces of content that were fact-checked as "false" or "altered" due to the complexities of Facebook's Misinformation Repeat Offenders policy. The operationalization of how we determine whether a source is considered untrustworthy is therefore more expansive than what as originally pre-registered and will necessarily overestimate exposure to untrustworthy sources as originally defined for this research initiative. We exclude from this list of domains other social media platforms, hosting sites, and URL shorteners, even if they may host URLs that have been fact-checked as false.

4.4 Passive tracking data

Participants who completed Wave 1 of the study and provided valid email addresses were asked for their consent to track their mobile and desktop internet browsing behavior. We use this data to compare the average audience ideology of web domains seen on Facebook and web domains visited from a browser (outside of Facebook) as reported in Table 34.

To collect the passive measurement data, NORC partnered with two vendors: MDI Global and RealityMine. Users who consented to passive data tracking were asked to install an app and use a virtual private network (VPN) on their mobile or desktop devices to collect data about the number of visits and time spent on different web domains as well as usage and time spent on apps on their mobile device. The app was developed by MDI Global and the VPN was developed and maintained by RealityMine. Both firms collected the passive tracking data and sanitized, truncated, and/or categorized the URLs to minimize the risk of sharing any additional personally identifiable information (PII).

The passive measurement software collected data on which applications participants were using and for how long on mobile devices, but no data were collected on what participants were doing within those applications. On all devices, information was collected on the websites that participants were visiting, but no additional information was collected beyond the domain name. For instance, a participant searching for information on "election night meals" on Google would be logged as www.google.com rather than https://www.google.com/search?q=election+night+meals. Further, k-anonymization was applied to the domain-level data by excluding domains that had visits from fewer than 20 unique panelists. The applications and VPNs only collected data while installed on a participant's device. The software could be uninstalled at any time and data collection could be paused using functionality in the apps.

To recruit participants for the passive measurement, the respondent's email address provided in Wave 1 of the Facebook sample survey was used to invite respondents to enroll. Participants whose email addresses were valid were invited to download the passive monitoring software from September 11–21, 2020.

The recruitment language was as follows:

Subject: 2020 Election Research Project: Additional Study Opportunity

As a member of the 2020 Election Research Project, you have been selected to participate in an additional study to learn more about the apps you use and sites you visit.

You can earn up to $90 for choosing to participate in this additional study. To participate, you only need to install the software and keep it active for the 3 month study

NORC at the University of Chicago and the study sponsor, Facebook, would like to understand more about how you're using your device during this study. To participate, you'll need to download software to your device. When installed, this software will automatically collect data about your device and the websites you visit and apps you use. The data will only be used

for research purposes. Please note that passwords, and other information you might enter on websites, like your banking details, will not be collected.

Those who clicked to learn more were provided with the following additional information: NORC at the University of Chicago and the study sponsor, Facebook, would like to understand more about how you're using your device during this study. To participate, you'll need to download an app, install a Virtual Private Network (VPN), or a browser plugin to your device. This software is developed by NORC's partners, MDI and RealityMine. When installed, this software will automatically collect data about your device and how you use it as further specified below, and no further action will be required from you. Please note that passwords, and other information you might enter on websites, like your banking details, will not be collected. You may install the software on one or more devices.

Installing this software is completely optional. Should you choose to install it, researchers at New York University, The University of Texas at Austin, and other academic institutions, as well as Facebook will use the data to better understand how online behavior changes in response to events during the course of the study. More information on this software can be found here: (FAQ information appended below).

Earn $5 per device just for installing and setting up the software. For your first 2 weeks of data sharing, earn an additional $5 per device (maximum 2 devices). You'll then earn another $5 per device at the end of your first month, if your devices are still sharing data. That means you could earn up to $30 in your first month! Keep participating and you'll earn $10 per month, per device. If you complete all 3 months, you'll receive a bonus $20 for 2 devices, or $15 for one device. This means you can earn up to $90 for 3 months of participation in this study! You will be paid for a maximum of 2 devices, though you may install the software on as many devices as you'd like. You may forfeit the monthly payment if you fail to send data from your mobile device for 3 days in a row or from your computer for 8 days in a row.

- All of your mobile device's data will flow through a VPN connection on iOS
- Web data for specific browsers will flow through a VPN on Android
- All of your desktop or laptop data will flow through a browser plugin
- Of the data that flows through the VPN or browser plugin, NORC will collect data on:
  - Your operating system, device model and manufacturer, and device type (e.g., mobile, tablet, desktop)
  - Which apps you use, including app name and category, the date and time you use the app, and for how long

- Which browser you use and technical details about your session such as your IP address
- What websites you visit, the date and time you visit a website, when and for how long

- NORC will use this data in order to facilitate the research and for data quality assurance purposes
- Of the data collected, the following device data will be shared with Facebook and Facebook's academic research partners:
  - Your operating system, model and manufacturer, and device type (e.g., mobile, tablet, and desktop)
  - Which apps you use, including app name and category, and for how long
  - Which browser you use
  - What websites you visit, the date and time you visit a website, when and for how long
- Your device data will be linked to your survey responses as well as publicly available third-party data, like if you've voted or made a political contribution, if this third-party data is available
- Your device data will not be used for ads
- Facebook will also combine your device data, your survey responses and the third-party data with your activity on Facebook from the 2020 calendar year, collectively called Combined Data
- This Combined Data will be shared with Facebook's academic partners and, if legally required, with the Institutional Review Board (IRB) that reviewed this study.
- Once this study is over, de-identified data (i.e. data where identifiers such as your name and other information that could reasonably be linked to you are removed) will be stored and shared for future research on elections, to validate the findings of this study, or if required by law for an IRB inquiry.

The FAQ included the following:

Along with your traditional surveys, this study gives you the opportunity to add software to your online devices to understand mobile and desktop behavior.

How does it work?

On mobile (Android/iOS) we use VPN services to understand web data usage on those devices, from which we can understand what sort on websites you visit. We also use this web data on iOS, or OS information on Android, to see what your favorite applications are. You can also download the application onto Windows and Mac which installs Browser Extensions onto Chrome, Firefox and Safari depending on what browsers you have installed.

What data do you collect?
We can collect data on what apps you are using and for how long on mobile, but we do not see what you do within those applications. Across all platforms we also collect information on what websites you have been on, for instance we could see if you have been using Google or YouTube on your browser, however we would not be able to see what you were searching or viewing on those channels, e.g. www.google.com was used for 5 minutes

Can I stop data being collected?
The applications and VPNs will only collect data whilst installed on your device. You may uninstall at any time or even pause data collection using functionality in the apps.

What else do the apps do?
The apps will sit in the background and passively collect data, meaning all you need to do is keep them installed to earn your rewards. The Android and iOS applications will send you notifications periodically to keep you up to date with the study, for instance letting you know when there is a survey to complete.

Participants were also given a link to more privacy details and terms and provided with a website allowing them to withdraw from the study at any time. The passive measurement recruitment phase closed on September 21, 2020. A total of 51,435 Wave 1 respondents provided consent to participate in the study, of which 21,198 (41%) downloaded, installed, and activated the software on at least one device on a total of 29,700 devices. The participation rates are included in Table 81. The final sample we analyze in this study is further restricted to respondents in the treatment and control groups that are relevant to our analysis, which contains a total of 3,821 users (approximately 37% of the total sample).

Table 81: Online passive tracking participation: Sample sizes and participation rates

Invited sample	125,694
Completed enrollment survey	20,822
Enrollment survey yield (complete/invited)	17%
Consent to participate	20,077
Consent rate (consent/complete)	96%
Total compliant users – Sept. 21	7,730
Total compliant users – Oct. 19	7,104
Total compliant users – Nov. 23	6,980
Total compliant users – Dec. 23	6,485

Active rate (active users/consent) – Sept. 21	39%
Active rate (active users/consent) – Dec. 23	32%
Total active devices – Sept. 21	10,421
Total active devices – Oct. 19	9,016
Total active devices – Nov. 23	9,078
Total active devices – Dec. 23	8,219
Deactivated (users) – Dec. 23	123
Non-compliant (users) – Dec. 23	5,358
Total user universe (active+deactivated+non-compliant)	11,966
Rate of dropout (users)	46%

4.5 Sampling, strata definitions, randomization, and power analyses

This experiment (reducing exposure to content from like-minded sources) is one of multiple "on-platform interventions" conducted as part of the U.S. 2020 Facebook & Instagram Election Study. We describe sampling, power calculation, participant recruitment, randomization, and weighting for the set of on-platform interventions including the like-minded sources demotion for which a sample of Facebook users were jointly recruited and assigned to different treatment conditions or a shared control group. (Other studies that were conducted as part of the U.S. 2020 Facebook & Instagram Election Study were also pre-registered and will be reported in separate articles.)

4.5.1 Sampling approach

The sampling approach was designed to achieve specific sample targets across different stages of the study. The study consisted of six survey waves (the first five of which are relevant for this paper).

The sample targets for each type of study are provided in Table 82. These targets were chosen to achieve desired minimum detectable effect sizes (MDEs) across different subgroups among the set of respondents participating in the wave 1 (recruitment) and wave 2 (baseline) surveys as well as at least one of waves 4 or 5 of the study, which we refer to as *three-wave completes*.[5] We worked backward from our target MDEs in waves 4 and 5 to determine the number of respondents we would need at the recruitment stage ("initial 3-wave" in Table 82). The three-wave completes target sample for platform interventions was adjusted after observing the level of attrition between the wave 1 and wave 2. In response, and prior to treatment randomization, the academic researchers in the study decided to cut two studies from the set of planned platform interventions on Facebook, which would have varied content in the Voting Information Center and removed Pages and groups content from Facebook Feeds. The revised targets can be found in the last column of Table 82 ("final 3-wave").

Table 82: Target sample size for platform interventions and surveys

			Sample targets	
**Apps**	**Study sample**	**Wave 1**	**Initial 3-wave**	**Final 3-wave**
FB	Platform interventions	119,000	71,118	54,740
FB	Surveys only	8,333	5,000	5,000

The three-wave sample targets were revised after observing the wave 1 and wave 2 survey completions and adjusting the proposed number of treatment arms prior to randomizing participants into condition.

[5] See the power calculations subsection below for details on how MDEs were determined across studies.

The table also includes sample size targets for users who were interested in taking the surveys associated with the study but not in having their experiences change on Facebook ("surveys only"). This sample was included to get a sense of the biases associated with selection into the study. There were no specific demographic targets for this sample as there were for other samples. The share of respondents in the surveys-only sample coming from each of the individual study samples was proportional to the target sample size for each study.

4.5.2 Sampling frames and strata definitions

The sampling frames included all Facebook monthly active U.S.-based users 18 years of age or older eligible to receive general surveys on a given platform (these represent a random set of users from the overall Facebook populations) as of August 17, 2020. Participants were asked to confirm they were over 18 years of age and lived in the United States as part of the recruitment process. The Facebook sampling frame was trimmed by removing predicted fake accounts, employees, and advertisers.

To guarantee sufficient representation of specific users in the sample, the sampling frame was stratified along the following covariates: number of days a user was active on a given platform (the number of days a user logged in to Facebook in the 30 days on or before August 17, 2020, classified into three categories: 1-14 days, 15-29 days, and 30 days), a user's predicted census region (East, Midwest, South, West),[6] whether the user is predicted to live in a battleground state,[7] a user's predicted ideology (liberal, moderate, or conservative),[8] and the census ethnic/racial composition in the zip code in which a user is predicted to live (percent of Hispanic residents and Black residents).[9] The stratification of the sampling frame for these samples generated 621 population cells.

4.5.3 Sampling probabilities and target distributions

Having defined the sampling frames, sampling probabilities were computed to achieve specific sample distributions for the set of demographics encoded in the stratification step across each

[6]The classification of states across each of the census regions is available here.

[7]Following the two most recent Electoral College ratings by the Cook Political Report prior to August, we defined as battleground states those whose complete electoral geography was considered in the "Toss Up", "Lean Democrat", or "Lean Republican" in at least one of the reports. "Toss Up" states included: Arizona, Georgia, Maine, North Carolina; "Lean Democrat" or "Lean Republican" states included: Florida, Michigan, Minnesota, New Hampshire, Pennsylvania, Wisconsin, Iowa, Ohio, and Texas. Nebraska was excluded because only one of three congressional districts was identified as a battleground district.

[8]See the Classifiers appendix in Section 4.3 for additional details on how ideology is predicted. Liberal users have predicted ideology score below 0.35, conservative users have a predicted ideology score above 0.65; moderates have a predicted ideology score between 0.35 and 0.65.

[9]Some fields had missing values (e.g., predicted ideology, state, and zip code). Individual values were imputed probabilistically using the distribution of demographics in the population. In general, the percent of missing values for a given demographic was quite small, never exceeding more than a few percentage points of the population.

Table 83: Target sample demographic distributions across studies (Facebook users)

Demographic	Study sample	Target distribution
Number of days user logged in	All studies	<15 days (4%); 15–29 days (24%); 30 days (72%)
Minority users (Black/Hispanic)	Platform interventions	56% in battleground states; 58% elsewhere
Users in battleground states	Platform interventions	40%

of the samples of interest. The sampling probabilities took into account (a) differential non-response across different demographics [10] and (b) the desired sample size across the different studies.

The Wave 1 target sample size took into consideration a total attrition rate of 40% between Wave 1 and the combination of Wave 2 and at least one of Waves 4 or 5 (three-wave completes) of the study, while ensuring our ability to detect an MDE of 1.5 percentage points on turnout and vote choice. The specific distributions we aimed for these samples are included in Table 83.

As shown in Table 83, the target distribution for Facebook users included 4% of those logging on for fewer than 15 days, 24% between 15 and 29 days, and 72% for 30 of the past 30 days. In battleground states, the target sample of minority (Black or Hispanic) users was 56% on both platforms. In non-battleground states, the target sample was 58% minority users on Facebook. The remaining rows show the targets based on geography and predicted ideology.

In meeting these targets, it is important to take into account three considerations:

1. All else equal, the probability of a user being invited to participate in the study in a given strata is proportional to its size in the sampling frame.

2. There was no initial target distribution for ideology. We incorporated this dimension to the stratification in the second week of recruitment after seeing that self-reported white liberal users were more likely to consent to participate in the study. We did so by oversampling moderate and conservative users based on their predicted ideology. No specific targets were identified, but the proportion of users who self-identified as Democrats was reduced.

3. Meta informed the academics that it had limited race/ethnicity data coverage for its U.S. users and it could not be used to inform large scale probability sampling. Instead, the probability that a given survey respondent identifies with a given ethnic/racial category was derived based on the ethnic/racial distribution of a user's predicted zip code. The approach to sample minority users had mixed results in the early stages of the recruitment period, and in light of observed imbalances in ideology, ideology targets were prioritized.

[10] Responses to Facebook surveys with a similar design were used to model differential response rates.

Implementation of sampling scheme After defining the sampling probabilities, the sampling scheme was executed across Facebook. In the implementation of the sampling scheme two additional steps were taken:

1. The sampling probabilities were adjusted when the size of a given stratum was exhausted. The adjustment to the sampling probabilities for non-exhausted cells across the sampling frame was done in proportion to their size.

2. Sampling was executed sequentially to avoid users being invited to more than one intervention within a given app. This left a small probability that users of Facebook and Instagram could have been invited to participate in a similar or different experience across the two apps.

Sampling for race and ethnicity The project aimed for specific sample proportions of Black and Hispanic users in the sample. Meta informed the academics that it had limited race/ethnicity data coverage for its U.S. users and it could not be used to inform large scale probability sampling. Therefore, the probability a given user identified with one of the categories of interest within a given strata of the sampling frame was modeled. The focus was on achieving targets for the White and Other ethnic categories, which allowed us to obtain the desired distribution for minorities as the residual categories, based on people's predicted zip codes. The implementation of this approach involved the following steps:

1. Let i represent one of the stratum of the S strata in the sampling frame of a given app. Let X_i represent the vector of covariates defining the characteristics of strata i. The vector X_i has the following components: number of days users were active on the platform in the last 30 days, census region, battleground state, predicted ideology, zip code percent Black, zip code percent Hispanic.

2. Within a given X_i we compute the probability that a user belongs to one of three different categories: Black, Hispanic, and White or Other. The following steps were followed:

   (a) We use the quartiles of percent Black and Hispanic in a given zip code. For these two categories we have four potential values 0–25%, 25–50%, 50–75%, 75%+.

   (b) The lower and upper bounds of the residual category are backed out of the combination of these quartiles. For example, in a zip code with 0–25% Hispanic and 0–25% Black, we know that the White or Other population represents at least 50% or at most 100%.

   (c) Based on this information, the probability of a user's race/ethnicity was derived using the midpoints of each of the quartiles across all ethnic categories. In the example introduced in the previous bullet, the probabilities were as follows: Pr(Hispanic) = Pr(Black) = 0.125 and Pr(White or Other) = 0.75.

(d) The only exception is when the bounds for White or Other are 0–25%. In this situation, Pr(White or Other) = 0.125 and Pr(Hispanic) = Hispanic midpoint * (1 - Pr(White or Other)) and Pr(Black) = Black midpoint * (1 - Pr(White or Other)). This solution applies to cases when the percent Hispanic is 0–25% and the percent Black is 75–100%. Otherwise, if we simply used midpoints to assign probabilities would yield Pr(Hispanic) = 0.125, Pr(Black) = 0.875, and Pr(White or Other) = 0.125, which would sum to more than one.

3. For a given attrition and click-through rate we back out the number of Wave 1 respondents in the White or Other category needed to achieve the target for this group in the three-wave complete sample. In particular, the number of Wave 1 respondents in the White and Other category is:

$$n_o|stratum_i = pr(White\,or\,Other|stratum = i) * pr(s = 1|stratum = i) * N_i$$

where $pr(s = 1|stratum = i)$ represents the probability a user in stratum i is invited to participate in the study and N_i denotes stratum i's population size.

Solving for the sampling probability in stratum i yields:

$$pr(s = 1|stratum = i) = n_o|stratum_i/(pr(White\,or\,Other|stratum = i) * N_i)$$

4. Note that the $pr(s = 1|stratum = i)$ derived in the previous step implies that we have a total number of invitations of Black and Hispanic users. These are given by:

$$n_{Black}|stratum_i = pr(Black|stratum = i) * pr(s = 1|stratum = i) * N_i$$

$$n_{Hispanic}|stratum_i = pr(Hispanic|stratum = i) * pr(s = 1|stratum = i) * N_i$$

From these quantities, we can back out the implied numbers of respondents in the Wave 1 and 3-Wave sample in a given stratum.

5. We repeated steps 1–4 to obtain across all strata S of the sampling frame of a given app to try to obtain the desired targets. However, as noted above, we prioritized over-sampling Facebook users on predicted ideology because (a) our initial approach exhibited mixed results in terms of meeting the specified minority targets and because (b) the early recruitment numbers showed a skew towards Democrats. To correct the partisanship skew, we simply repeated steps 1–4 across each of three predicted ideology strata (liberal, moderate, and conservative) with a higher proportion of survey invitations allocated to the moderate and conservative strata.

4.5.4 Power calculations

We designed our sampling approach with the goal of recruiting the minimum number of respondents required to detect meaningful effect sizes. Our target minimum detectable effects (MDE) were:

- MDE=1.5 percentage points change in vote choice for the full sample.
- MDE=2.5 percentage points change in vote choice among respondents in battleground states, oversampling respondents from these states.
- MDE=2.5 percentage points change in vote choice for African-American and Hispanic respondents, with oversampling.
- Our calculations assumed a design effect of 1.3. In order to incorporate a user's frequency of app use as an oversample category, we relaxed the assumption to 2.0. The actual design effect differed based on the actual sample composition and weighting strategy.
- The size of the control and treatment groups was chosen based on what maximized power assuming a fixed budget.

Sample size calculation assumptions: For platform intervention experiments, which included the like-minded demotion intervention:

- An independent-arm study was used, as opposed to a factorial design, due to power considerations. Because we anticipated we would observe large interaction effects across treatment arms (*81*), we expected that a factorial design would not increase the power of our analysis while adding unnecessary complexity to the study.
- We assumed that all treatment groups would be equal in size (*82*) and the control group would be $\sqrt{t}$ times larger than the number of treatment groups where t = number of treatments (initially $t = 8$ for Facebook).
- We focused on two-party vote choice as the key outcome variable for sample size analysis and assumed it had mean 50% and standard deviation 0.5.
- We assumed that controlling for baseline candidate preferences reduce standard errors by 45% based on prior data (*83*). Thus, the residual standard deviation is 0.275.
- We required 80% power and alpha=0.05 in a two-sided test.

Estimated Minimum Detectable Effects (targets):

- Based on these assumptions, we initially estimated that we would require 81,918 respondents (three-wave completes) on Facebook — 26% (21,398) in the control group and 74% (60,520) in the treatment groups (7,565 per arm)

- These target sizes include both the standard platform interventions discussed above and an additional platform intervention that required a more targeted sample. Users in this second treatment were selected using a different sampling strategy. For that reason, respondents in that treatment arm for each platform were reassigned, as well as a proportional part of the control group, to a separate sample with target size 10,800 for each platform.

- Due to the observed attrition between wave 1 and wave 2, the number of treatment arms for our standard platform interventions were reduced from 7 to 5 for Facebook respondents.

  - The updated probabilities of assignment to control and treatment groups were computed using the optimal allocation rule described above, with the control group being $\sqrt{t}$ times the optimal treatment arm size.

  - In this new scenario, we estimated a target sample size (three-wave completes) for the standard platform interventions of 54,740 respondents on Facebook.[11]

- This sample size would allow for the detection of MDE = 1.5 on self-reported turnout for the entire sample (assuming that the residual standard deviation is 0.335) on Facebook. Note that the actual MDEs are different based on the consent rates, attrition, and covariate adjustment.

4.5.5 AmeriSpeak sample

In addition to our main study sample, a nationally representative general population sample of U.S. adults age 18+ who had responded to an AmeriSpeak survey invitation in the previous six months was selected from NORC's AmeriSpeak Panel for this study.

The wave 2 sample was selected from the AmeriSpeak panel using sampling strata based on age, race/Hispanic ethnicity, education, and gender (48 sampling strata in total). The size of the selected sample per sampling stratum was determined by the population distribution for each stratum. In addition, sample selection took into account expected differential survey completion rates by demographic groups so that the set of panel members with a completed interview for the study is a representative sample of the target population (general population age 18+). For panel households having more than one active adult panel member, only one adult in the household was eligible for study selection (random within-household sampling). Panelists selected for an AmeriSpeak study earlier in the business week are not eligible for sample selection until the following business week.

For technical information about the AmeriSpeak panel, including recruitment process and panel management policies, see `https://amerispeak.norc.org/research`.

[11]The new target sample sizes were computed as: $\sqrt{t}$ x N target 3-wave completes + t x N target 3-wave completes

All non-withdrawn respondents from AmeriSpeak who completed the wave 2 survey were invited to waves 3–5 regardless of whether they completed any of the other waves. Non-withdrawn study participants completing either waves 4 or 5 were invited to participate in wave 6.

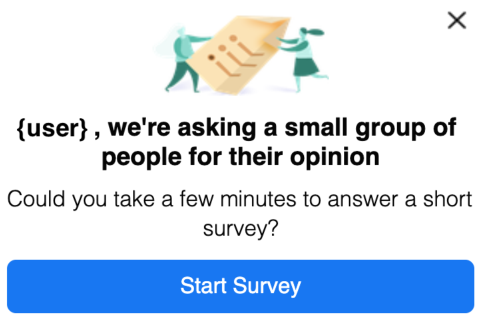

Figure 68: Image shown to recruit participants

4.6 Participant recruitment and consent

4.6.1 Recruitment language

At the top of their Facebook feed, randomly selected participants saw a recruitment message asking them if they would like to share their opinion as shown in Figure 68. Those clicking "Start Survey" were directed to a consent form.

Participants gave their consent to participate using an IRB-approved consent form as follows:

Do You Want to Participate in a Research Study About the US Election in November?

Your participation in this research will help researchers at New York University, The University of Texas at Austin, and other academic institutions, as well as Facebook, understand more about how people's experience with Facebook and Instagram affects their opinions and behaviors on elections.

How it Works

Over the next four months, you'll be asked to fill out a short survey each month. This monthly survey will take about 15 minutes, for a total of 60 minutes over four months. Our partner, NORC at the University of Chicago, will administer this research.

During this time, your [Facebook/Instagram] experience may be different than what you're used to. For example, you might:

- See more or fewer ads in specific categories such as retail, entertainment, or politics
- See more or fewer posts in [News Feed / your feed] related to specific topics
- See more content from some [friends/connections] and less content from other [friends/connections]
- See more or less content about voting and elections

You'll be paid at least $30 for participating in this study and completing all four surveys, including $5 for each of the first two surveys and $10 for each of the final two surveys.

- You will receive your reward as an electronic gift card, delivered within 1 day of completing each survey
- You can only take each survey once
- If you do not complete the first survey, you will be removed from this study

If you choose to participate in this study, your survey responses will be linked with your Facebook and Instagram activity data from the 2020 calendar year.

Benefits, Alternatives, and Risks

There are no benefits to participating in this research, nor are there risks greater than those encountered in everyday life, including risks related to the loss of confidentiality. You can learn more about how we're keeping your information safe in the Data Collection and Your Privacy section below. You can choose not to participate in this study.

Data Collection and Your Privacy If You Choose to Participate in the Study

- NORC will join your survey responses to publicly available third-party data like if you've voted or made a political contribution, if this data is available
- Facebook will combine this data with your activity on Facebook and Instagram from the 2020 calendar year, collectively called Combined Data
- This Combined Data will only be used for research purposes and will not be used to show you ads
- This Combined Data will be shared with our academic partners and, if legally required, with the Institutional Review Board (IRB) that reviewed this study
- All access to this Combined Data will be monitored and logged
- Once this study is over, de-identified data (i.e., data where identifiers such as your name and other information that could reasonably be linked to you are removed) will be stored and shared for future research on elections, to validate the findings of this study, or if required by law for an IRB inquiry

You can decide to stop participating in this study at any time, for any reason, and without consequences. You may withdraw by visiting the study website hosted by our survey administrator, NORC at the University of Chicago, at 2020erp.norc.org

If you have any questions related to this research, you can email NORC at erpStudy@norc.org, or call toll-free at (866) 270-2602 between 9:00 AM - 10:00 PM ET.

If you are a research participant and have questions about your rights, or have concerns or complaints about this research, you can email the NORC Institutional Review Board (IRB) at surveyhelp@norc.org or call (866) 856 - 6672 between 9:00 AM and 10:00 PM ET. Please note that by contacting or providing information to NORC IRB, NORC IRB may obtain information about you, including any personal information that you share. Even though NORC IRB is affiliated with Facebook as this research study's IRB, Facebook's Data Policy does not apply to any information about you shared with NORC IRB when you initiate contact.

If you join this study, you affirm that you are at least 18 years of age and live in the United States. Once you join this study, you'll be sent off [Facebook/Instagram] to a site hosted by our study administrator, NORC, to complete a 5-minute enrollment form.

4.6.2 Data collection timeline

Data collection began with a soft launch on August 31, 2020 and continued through March 2, 2021. The dates of each field wave were as follows:

- Wave 1: A subsample of Facebook-recruited respondents were invited to the survey on August 31 in a soft-launch. The remainder of sampled Facebook-recruited respondents were invited to the survey on September 1. The recruitment of the sample continued until Saturday, September 12. The wave included the recruitment and consent processes and a short survey.

- Wave 2: The field period for Wave 2 started on September 8 and continued through September 23. The wave included a baseline survey and was conducted prior to randomization.

- Wave 3: The field period for Wave 3 started on October 9 and continued through October 23.

- Wave 4: The field period for Wave 4 started on November 4 at 12:05 am Central Time and continued through November 18.

- Wave 5: The field period for Wave 5 started on December 9 and continued through December 23. The survey started approximately one week later than the original schedule due to obtaining approvals for updated informed consent language.

- Wave 6: The field period of Wave 6 started on February 16 and continued through March 2.

During this period, the platform interventions ran from September 24 to halfway through the day on December 23, 2020.

Table 84: Definition of blocks for randomization and treatment assignment probabilities on Facebook

Study sample	Covariates defining blocks	p(treatment) within block
On-platform (incl. like-minded demotion)	Battleground state, friend count, self-reported party ID, race (36 blocks)	Pr(Control) = 0.3090, Pr(Treatment = i) = 0.1382 for i = 1, . . . , 5.

4.6.3 Randomization

We adopted block randomization to minimize variance of treatment effect estimates and to ensure in-sample balance in a set of covariates that may be important determinants of the outcomes of interest.

To implement the block randomization approach, we first refined the set of study participants to include only those who completed the Wave 2 survey. This approach was adopted to minimize attrition between the baseline and the rest of the surveys in the study.

A combination of survey-based pre-treatment outcomes and Facebook data were used to define the blocks in the sample of interest. The proportions of the sample assigned to treatment and control groups were chosen as described in Section 4.5. The set of metrics used for blocked randomization and the treatment assignment probabilities in each of the samples can be found in Table 84.

The coding criteria used to create the blocks for treatment assignment are shown in Table 85.

Table 85: Coding criteria to create the strata for block randomization

Variable name	# potential values	Values
Battleground State	2	Yes, No
Following/friend count	3	Observed terciles in sample
Self-reported party ID	3	Democrat (with leaners), Independent, Republican (with leaners) [wave 1 survey response]
Race	2	White, non-white (wave 1 survey response)

4.6.4 Response rates

In Table 86, which shows the response rates, eligible users sampled (A) is based on the sampling methods described in Section 4.5. The number of participants passed to NORC (P) is lower than those who consented to the full study (F) because not all users successfully followed the URL link to the NORC website. Eligibility confirmed by NORC (Q) is based on respondent age (above 18), country (US), and removing duplicate cases.

Table 86: Survey completion and response rates

Row	Measure	Interventions	Definition
	**Pre-NORC recruitment**		
A	Eligible users sampled	22,965,580	
B	Viewed recruitment	14,643,120	
C	Clicked on recruitment	988,247	
F	Consented to full study	193,880	
G	Did not consent to full study	794,367	C-F
J	Recruitment viewers among sampled	63.8%	B/A
K	Recruitment clickers among recruitment viewers	6.7%	C/B
N	Consented among asked	19.6%	F/C
O	Response rate before pass to NORC	0.8%	J*K*N
	**Wave 1**		
P	Passed to NORC	189,792	
Q	Screened for eligibility	163,207	
R	Confirmed eligible	162,698	
S	Completed wave 1	139,193	
T	Wave 1 completion rate among passed to NORC	86.0%	Q/P
U	Eligibility rate among screened	99.7%	R/Q
V	Interview completion rate among eligible	85.6%	S/R
W	Response rate among those passed to NORC	73.6%	T*V
X	Cumulative response rate, pre-NORC recruitment wave 1	0.6%	O*W
	**Wave 2**		
Y	Screened for eligibility	77,438	
Z	Confirmed eligible	77,405	
AA	Completed wave 2	75,276	
AB	Screener completion rate among invited	55.6%	Y/X
AC	Eligibility rate among screened	100.0%	Z/Y
AD	Interview completion rate among eligible	97.3%	AA/Z

AE	Response rate among invited, wave 2	54.1%	AB*AD
AF	Cumulative response rate, pre-NORC recruitment waves 1 2	0.3%	X*AE
	**Wave 3**		
AG	Withdrawn before wave 3	87	AA-AH
AH	Invited	75,189	
AI	Completed wave 3	56,866	
AJ	Interview completion rate among invited	75.6%	AI/AH
AK	Response rate among invited + withdrawn, wave 3	75.5%	AI/(AM+AN)
AL	Cumulative response rate, pre-NORC recruitment waves 1 2 3	0.3%	AF*AK
	**Wave 4**		
AM	Withdrawn before wave 4	138	AA-AN
AN	Invited	75,138	
AO	Completed wave 4	56,195	
AP	Interview completion rate among invited	74.7%	AO/AN
AQ	Response rate among invited + withdrawn, wave 4	74.6%	AO/(AM+AN)
AR	Cumulative response rate, pre-NORC recruitment waves 1 2 4	0.3%	AF*AQ
	**Wave 5**		
AS	Withdrawn before wave 5	169	AA-AT
AT	Invited	75,107	
AU	Completed wave 5	54,208	
AV	Interview completion rate among invited	72.1%	AU/AT
AW	Response rate among invited + withdrawn, wave 5	72.0%	AU/(AS+AT)
AX	Cumulative response rate, pre-NORC recruitment waves 1 2 5	0.2%	AF*AW
	**Three-Wave (W1 W2 (W4 or W5)**		
AY	Completed waves 1 2 (4 or 5)	58,886	
AZ	Interview completion rate for wave 4 or 5 among wave 2 completes	78.2%	AY/AA
BA	Cumulative response rate among cases sent to NORC, waves 1 2 (4 or 5)	31.1%	W*AE*AZ
BB	Cumulative response rate, pre-NORC recruitment waves 1 2 (4 or 5)	0.3%	AF*AZ

4.7 Survey weights and PATE estimation

Survey weights were created to generalize treatment effects to the best estimate of adult monthly active users (all U.S. Facebook monthly active users 18 years of age or older eligible to receive general surveys on a given platform, which represent a random set of users from the overall Facebook population as of August 17, 2020) in the Facebook population. The general approach to creating the weights was to reduce bias while maintaining a low design effect.

Inverse Propensity Scores Weights (IPSW) were built using LASSO regression with Facebook log data. Covariates used for block randomization and variables presumed to predict treatment heterogeneity were prioritized. For the platform interventions, the weights calibrate to the full population of Facebook users.

When the variable was used as part of block randomization, the terciles were based on the intervention sample. If the variable was not used as part of the block randomization, then we used the population and sample to define the terciles.

For Facebook, weights were built using:

- Predicted ideology (divided into liberal, moderate, and conservative using the ideology classifier described in Supplementary Information, section 4.3 with cut points of 0.35 and 0.65).
- Friend count divided based on terciles
- Political Pages followed divided by tercile
- The number of days a user logged on to their account in the 30 days prior to August 17, 2020, divided into 29 or fewer versus 30.

For on-platform experiments, whether the respondent was in a swing state was also included.

If the variable was used in the block randomization step (see Table 84), the terciles were based on the intervention sample. If the variable was not used in the block randomization step, the sample is combined with the population to define the terciles of the variable in question.

Raking was used to create the set of final weights that calibrate to population estimates of race (white vs. non-white), party ID (Democrat, Independent, or Republican, including leaners as partisans), and education (less than a college degree vs. a college degree or more). The specific targets are based on the Wave 2 Amerispeak panel weights for those who reported having a Facebook (FBACCT_ACTIVE_ONE) account. More information about the Amerispeak panel is available upon request.

The final step was to trim the weights. Following the Cooperative Election Study, which trims weights above a particular threshold and the Pew Research Center, which has trimmed weights at the 1st and 99th percentiles, the top 1% of the survey weights were trimmed.

Design weights were not included in the computation of the survey weights as the weights increase the design effect significantly without appreciably decreasing the bias.

Because many of our outcomes rely on items from specific waves, we produced wave-specific weights. These wave-specific survey weights calibrate the sample who completed each

wave to the Facebook population, as described above, in order to deal with survey unit non-response and sample stratification. For outcomes that rely on survey items across multiple waves, we took the average of the weights across the survey waves used to compute the outcomes.

4.8 Meta/academic collaboration overview

The *U.S. 2020 Facebook & Instagram Election Study* was designed to address three intertwined concerns related to scientific understanding of the impact of social media on democratic processes. First, in the aftermath of the 2016 US elections, there was a widely recognized need to understand the impact of social media platforms on US elections. Second, research conducted solely by employees of these same platforms could possibly encounter skepticism from the mass public and policy community. At the same time, researchers not employed by the platforms faced legal and fiduciary challenges in securing access to the data and research pipelines to conduct the rigorous scientific analyses necessary to answer questions about the impact of social media platforms on elections.

The *U.S. 2020 Facebook & Instagram Election Study* is an attempted solution to this bundle of challenges. The project represents a novel form of collaboration between a team of researchers at Meta and a set of external researchers. The costs associated with the research (e.g., participant fees, recruitment, data collection, etc.) were paid by Meta. The external team members received no financial compensation from Meta for their participation in the project.

We acknowledge the importance of such transparent collaborations with academic researchers internationally and in the United States beyond the scope of this project. However, we also recognize that Meta's ability to engage in similar collaborations may be constrained by the broader regulatory environment. Given that both regulations and functionalities of Facebook change rapidly (e.g., the GDPR in the European Union or the disabling of ads targeting by ideology), replications and extensions of this project may be challenging (if not impossible in some places).

Professors Natalie Jomini Stroud of the University of Texas at Austin and Joshua A. Tucker of New York University, at the time Chairs of the North American Regional (Stroud) and Electoral Integrity (Tucker) Social Science One Advisory Committees, selected and co-chaired a team of 15 additional external academic researchers (that is, researchers not employed by Meta); as part of the agreement, Meta did not have veto power over the academics selected for the team. Although there were many qualified researchers who could have been involved, the original members of the academic team for this project were selected based on their prior involvement with Social Science One and their expertise in social media and politics. Additional researchers were brought on as needed based on their substantive and methodological expertise.

Chad Kiewiet de Jonge was the Meta research manager who oversaw day-to-day management of the research project at Meta. Annie Franco and Winter Mason co-led the Meta research team, which grew to include 16 researchers, two data engineers, one data scientist, and three interns working on various parts of the overall project.

Once assembled, the team of academics met beginning in March of 2020 to first brainstorm research ideas within the project's mandate of studying Facebook and Instagram's impact in the context of the 2020 elections and then to develop ideas for specific paper proposals. Concurrently, the team of Meta researchers began working with the academic team to provide feedback on research proposals, including the feasibility of possible designs and procedures for collecting the necessary data. As a result of this process, four general areas of inquiry were selected

to form the scope of project: (1) dis/mis/information, knowledge, and (mis)perceptions; (2) political polarization; (3) political participation, both online and offline, and including vote choice and turnout; and (4) attitudes and beliefs about democratic norms and the legitimacy of democratic institutions.

The next step in the project involved identifying specific paper topics within these general scope conditions. Based on their research interests, a subset of academic researchers served as "core authors" of each paper and were given control rights over final versions of the pre-analysis plans and papers.[12] Both the academic researchers and the Meta researchers worked together to design the pre-analysis plans.

Data collection was carried out by Meta and NORC, an independent survey research organization at the University of Chicago.[13] Meta recruited most participants (see Section 4.5 for details) and collected on-platform data, while NORC carried out all surveys associated with the project, collected and appended all supplemental data outside of the Facebook/Instagram on-platform data, and recruited additional survey panelists. The academic research team did not contact any human subjects as part of the research efforts. In the rare cases where members of the academic team – who had been publicly announced – were messaged by study participants, the messages were passed to NORC to respond.

At the data analysis stage, the Meta team wrote, and the academics reviewed and approved, pipeline code used to produce the data tables needed for this project from raw platform data (e.g., number of followers) and data created for other internal Meta purposes (e.g., predictions of ideology of U.S. Facebook users) that were employed in the analysis. The Meta researchers, and in some instances the academics, carried out the initial analyses as detailed in the pre-analysis plan and as deemed necessary by the full research team for mutually agreed upon research-relevant analyses. The academics' role in the analysis was to contribute to and monitor the results of data analyses conducted by the Meta research team, including: reviewing and, in some cases writing, code; inspecting de-identified samples or aggregated outputs through screen sharing; and, when possible, replicating the analyses within Meta's secure data-sharing Researcher Platform using data that had been stripped of any individually-identifying information.

Drafts of papers were written by the academic research team members with feedback from the Meta academic researchers. Final control rights rested with the specified core academic authors.

A full description of the roles and responsibilities of the academic research team, the Meta researchers, and NORC can be found at the Open Science Foundation: https://osf.io/upkns/. A public FAQ answering common questions about the U.S. 2020 Facebook & In-

[12]By *control rights*, we mean that in the event of disagreements between members of the research team, the core authors would have the final say in resolving these disagreements.

[13]NORC was selected following a competitive bidding process involving other online survey research firms. To be clear, employees of NORC who implemented the data collection process were not members of the academic research team. More details about NORC can be found at: https://www.norc.org/Pages/default.aspx.

stagram Election Study is available here: https://medium.com/@2020_election_research_project/266d30cbe95b.

Research transparency and integrity: One of the primary goals in designing the project was to build in transparency concerning the research process given the constraints under which we were operating. With this in mind, five conventions were adopted to guide the research process.

First, none of the academic researchers nor their institutions received financial compensation (e.g., support for student assistants, course buyouts, research funds) from Meta for their participation in the project.

Second, the analyses for all the papers resulting from the project, including this one (https://osf.io/3sjy2), were preregistered at the Open Science Foundation. The pre-registrations were embargoed while the research was being carried out, but are being made available to reviewers and will be publicly released at time of publication.

Third, for every paper, a set of core authors with control rights over the final content of the paper were specified in the pre-analysis plan. These core authors consist only of external academic researchers (i.e., not employees of Meta).

Fourth, Meta publicly agreed that there would be no pre-publication approval of papers for publication on the basis of their findings. At the time the pre-analysis plans were proposed – but before any data analysis was conducted – Meta conducted legal, privacy, and feasibility reviews of the studies. Meta was entitled to review papers prior to publication, but could only request changes to protect confidential or personally identifiable information or to abide by their existing legal obligations.[14] For this article, Meta did not request any changes following the pre-publication review. Meta did request revisions to statements about race and ethnicity included in the appendices of another article in the US 2020 Facebook and Instagram Election Study. The revised sentences originally stated that Meta does not have any data on the race or ethnicity of its users. Meta requested the clarification that it had limited race/ethnicity data coverage for its U.S. users and it could not be used to inform large scale probability sampling. As a result, we made corresponding changes to the corresponding language in the SM of this article.

Fifth, we appointed a rapporteur for the project — Professor Michael Wagner of the University of Wisconsin, Madison — who was neither a paid employee of Meta nor a member of the academic research team. The rapporteur was given access to all participants, allowed to join project-related meetings, and had access to project documents. The rapporteur will not be a co-author on any of the papers resulting from the study, but the expectation is that the rapporteur will publish both academic and popular press articles assessing the research process itself.

Finally, Meta plans to make de-identified datasets from each published study conducted under this initiative and designed in collaboration with the academic team available to the broader research community, so that others can reproduce the analyses and conduct further election studies.

Below we list declarations from the academic author team. For consistency, we use the

[14] https://about.fb.com/news/2020/08/research-impact-of-facebook-and-instagram-on-us-election/.

following key:

a	Current employee (Meta)
b	Past employee (Meta)
c	Own individual stocks (Meta)
d	Paid consulting work (Meta)
e	Direct research funding from Meta (grant to you as PI or Co-PI)
f	Received an honorarium/fee (from Meta) for attending or hosting an event/serving as outside expert
g	Attended a Meta event where food, travel, or lodging was paid for by the company
h	Current employee (at a related company: Twitter, TikTok, Google/YouTube)
i	Past employee (at a related company)
j	Own individual stocks (at a related company)
k	Paid consulting work (at a related company)
l	Direct research funding from a related company (grant to you as PI or Co-PI)
m	Received an honorarium/fee (from a related company) for attending or hosting an event/serving as outside expert
n	Attended an event (at a related company) where food, travel, or lodging was paid for by the company

Academic author declarations:

- Hunt Allcott: former Microsoft employee; none of the above
- Annie Chen: none of the above
- Deen Freelon: g
- Matthew Gentzkow: f, g, m, n
- Sandra González-Bailón: g, l
- Andrew Guess: e, g
- Edward Kennedy: none of the above
- Young Mie Kim: g
- David Lazer: g, n
- Neil Malhotra: g, n
- Brendan Nyhan: e, g, n
- Jennifer Pan: e, f, g

- Jaime Settle: c, e, g, j
- Natalie Jomini Stroud: d, e, g, l, n
- Emily Thorson: g
- Rebekah Tromble: e, g, l
- Joshua A. Tucker: e, f, g, n
- Magdalena Wojcieszak: e, g, n

Authors employed by Meta during the project are listed below:

- Pablo Barberá
- Taylor Brown
- Adriana Crespo-Tenorio
- Drew Dimmery
- Devra Moehler
- Daniel Robert Thomas
- Carlos Velasco Rivera
- Arjun Wilkins
- Beixian Xiong
- Annie Franco
- Chad Kiewiet de Jonge
- Winter Mason

We also provide the following additional disclosures:

- Ancillary support (e.g., research assistants, course buyouts, etc.) was sourced by academics from the Democracy Fund, the Guggenheim Foundation, the John S. and James L. Knight Foundation, the Charles Koch Foundation, the Hewlett Foundation, Hopewell Fund, the Alfred P. Sloan Foundation, the University of Texas at Austin, New York University, Stanford University, the Stanford Institute for Economic Policy Research, the University of Wisconsin-Madison.

- Hunt Allcott received compensation from another source for consulting on a matter related to Meta's business. H.A. was an employee of Microsoft Research until December 2022.

- Matthew Gentzkow received compensation from Compass Lexecon for advice on a project for which Meta was the client. He has been a paid consultant for Amazon and done economic consulting for Analysis Group for clients including Google. He has received compensation as a member of the Toulouse Network for Information Technology, a research group funded in part by Microsoft.

- Brendan Nyhan has served as a member of the Misinformation Interventions Working Group at Meta since 2022. Meta has donated the honorarium he would receive for participating in this group to Doctors Without Borders on his behalf.

- Jennifer Pan served as a member of Meta's News Integrity Circle from fall 2020 to fall 2022. She received an honorarium for participating in the fall 2020 and spring 2021 session; the honorarium she would have received for participating was donated to Reporters without Borders for the fall 2021, spring 2022, and fall 2022 sessions.

- Natalie Jomini Stroud provided consultant services to Facebook in 2018–2019 on a prior research project; she was paid before the current project began.

- Joshua A. Tucker received a fee from Facebook to compensate him for administrative time spent in organizing a 1-day conference for approximately 30 academic researchers and a dozen Facebook product managers and data scientists that was held at NYU in the summer of 2017 to discuss research related to civic engagement; his fee was paid before the current project began. He is currently a Kroll Institute Fellow and Senior Advisor at Kroll.

4.9 Additional ethics considerations

Researchers involved in the project considered a number of ethical concerns related to the research and designed the studies to minimize potential harms to respondents as well as any broader social harms.

4.9.1 Ethics and individual participants

All experimental treatments involve withholding components of Facebook or Instagram that have been identified in the academic literature as having potentially negative effects (e.g., feed algorithms). Individual-level participation in the experimental analyses and surveys was compensated and required informed consent (see section 4.6 for details). We believe that the societal benefits of the study (e.g., the knowledge about Facebook and Instagram's impact in the election that will be generated) outweigh its potential harms to respondents, which will not be larger than what individuals experience in their ordinary life. Accordingly, Meta sought review from and was granted approval to conduct the experimental studies by the NORC Institutional Review Board (Protocol number 20.08.10, Project number 8870). Academic collaborators worked with their respective university IRB's to ensure compliance with Human Subjects Research regulations in their authorship of papers, including analysis of aggregated, de-identified data collected by Meta and NORC.

4.9.2 Re-identification risk

For the individual-level data, the following variables were coarsened to reduce re-identification risks.

- INCOME (18 categories) → INCOME (3 categories: less than $49,999, $50,000 to $99,999, $100,000 or more); see Wave 1
- EDUCAT (14 categories) → EDUC5 (5 categories: less than high school, high school diploma, vocational degree / some college, college degree, graduate degree); see Wave 1
- HISPAN (8 categories), RACE_1 (15 categories) → RACETHNICITY (6 categories: White, non-Hispanic; Black, non-Hispanic; Other, non-Hispanic; Hispanic; 2+ non-Hispanic; Asian, non-Hispanic); see Wave 1
- ZIP (41,692 categories) → IS_SWING_STATE (2 categories based on Cook Political Report); see Wave 1
- ZIP (41,692 categories) → IS_SWING_CD (2 categories based on Cook Political Report); see Wave 1
- RELIGION (12 categories) → RELIGION (4 categories: Protestant, Roman Catholic, Mormon, Eastern or Greek Orthodox & not born-again; Protestant, Roman Catholic,

Mormon, Eastern or Greek Orthodox & born-again; Jewish, Muslim, Buddhist, Hindu, Something else; Atheist, Agnostic, Nothing in particular); see Wave 5

4.9.3 Ethics and experiments during an election

As a mitigation strategy to minimize unanticipated negative effects, we implemented a stopping rule, inspired by clinical trials, which would have ended a treatment if we detected that it was generating changes in specific variables relevant to individual welfare that were much larger than expected. The results of this analysis are described in Section 1.2

An additional concern related to running experimental studies during an election period is the downstream risk of inadvertently impacting the outcome of an election. In order to mitigate against this possibility, we calculated the largest possible impact on an election outcome we could expect from our study as part of the process of designing the size of our treatments. As the study was designed, the number of people recruited into any of the treatment groups would have been at most 0.044% of the citizen voting-age population in the US (i.e., citizens who are eligible to vote). Participants were distributed randomly across the US, with some oversampling of people in battleground states. Under the largest effect scenario, i.e., that in which our interventions have the same effect as mobilization or persuasion campaigns, we would expect at most a change of 49 votes (in either direction) in the largest state or 1 vote (in either direction) in the largest congressional district;[15] and an increase in turnout of at most 1,175 votes in the largest state and 35 votes in the largest congressional district.

4.9.4 Aggregated platform data

Platform-wide data includes aggregated data on adult U.S.-based monthly active Facebook users, including the creation of, impressions on, and engagement with content during the pre-treatment period or the study period. This data is referred to as 'US MAP' in our tables.

In intentionally building privacy protections into the research design, the academic research team did not have access to individual-level MAP data. Rather, MAP data were shared with the academic team in a summarized, aggregate form. Code to collect and process the data from Meta servers was written by Meta researchers and then reviewed by at least one member of the academic research team. The academic researchers only had access to code that was implemented within Meta specifically for this project (e.g., the pre-existing code to aggregate the ratings from third-party fact checkers into misinformation "strikes," as defined under the company's Misinformation Repeat Offender policy, was not provided for review).

While the use of this MAP data is permissible under Meta's Data Policy, care was nevertheless taken to ensure that user privacy was protected. These procedures included the following:

- First, all requests were reviewed by Meta Legal and Privacy to ensure that no individual-level or identifying data were shared with the academic research team.

[15] To be clear, there were no experiments included in the study that we expected to benefit any particular candidate; these are simply the largest effects we could expect to occur *in either direction* based on prior research.

- Second, techniques were used to reduce the risk of any individually identifying data being shared. These techniques included ensuring that all metrics reported in this paper are based on at least 100 potential contributing users.

4.9.5 Professional ethics advice

Meta retained the services of *Ethical Resolve*, a data ethics firm that was consulted by both Meta and academic researchers at various stages of the project prior to implementation of the research to evaluate whether it met long-running traditions of research ethics as well as emerging norms and best practices for conducting digital research.[16]

[16] https://ethicalresolve.com/

4.10 Deviations

This study follows the final version of the study preregistration filed with the Open Science Framework on March 3, 2021 (https://osf.io/3sjy2) with the exception of the deviations listed below. (Note: The first study preregistration was filed on September 24, 2020, an amended version was filed on December 1, 2020, and the final version was filed on March 3, 2021, before access to data from the study was available. Date-stamped versions of each preregistration are available at https://osf.io/3sjy2. Each amended version also uses color-coded text to indicate where edits took place.)

PAP page	Original plan	Revised plan	Justification
5	"For each of the following categories of policy violations, the proportion of views with links to domains or to content from Pages or Groups that have been assigned two or more lifetime strikes for the following: violence or incitement, misinformation and health harm, misinformation and harm, terrorists and organized hate groups, coordinating harm, voter suppression and fraud, bullying and harassment , engagement abuse, content spam, inflating distribution, hate speech, coordinated inauthentic behavior"	Reduce to 1 strike for domains; for Pages or groups, include if 2 or more strikes overall and then count in category if 1 or more strikes for that category	Some of these categories can only get one strike
5	We did not specify whether the analysis of web tracking data is conditional on survey completion	We will only use respondents who completed Wave 3, 4, or 5	In the analysis of the FB log data, we're also subsetting to respondents who completed the study.
6	"content classified as hateful and intolerant speech"	Content classified as "containing one or more slur words"	In selecting a classifier to detect potentially hateful content, we chose to err on the side of false positives (recall) as opposed to false negatives (precision). As a result, some of the content labeled as containing slur words may capture ingroup discourse and banter (e.g., use of the n-word among Black communities and use of "bitch" among women), neither of which the classifier is designed to filter out.
6	"Mean/median/distribution (e.g., p2.5 p97.5) of days active and average time spent per day relative to the U.S. mean among monthly active users"	Average number of standard deviations of time spent that a respondent is above or below the running monthly average time spent for monthly active users	Clarification

(continued on next page)

PAP page	Original plan	Revised plan	Justification
7	"In addition, we will conduct exploratory analyses to examine how treatment effects vary both by subgroups generated from survey measures (e.g., age, gender, race/ethnicity, in particular among African Americans and Hispanics), behavioral measures (e.g., Facebook or IG tenure), and participant's location in a battleground state as determined by Cook Report as of July 23, 2020."	"Treatment effects" as written here refers to treatment effects on exposure/behavior. We will examine how exposure to untrustworthy sources varies across these subgroups, then investigate behavioral measures as well if we see meaningful differences in exposure.	Clarification
9–19	For scales that did not employ factor analysis, we did not specify thresholds for (1) loading and (2) missingness.	When factor analysis is specified for scale creation in PAP: Include item in scale only if factor analysis loading $>$ .7 and missingness $<$ .15	Clarification
10	In H2, we specified that we would operationalize 'Partisan and ideological extremity' with post-treatment self-reports of PID and ideology	Instead, we will only use ideology	Party ID was not included in a post-treatment wave
12	In H5, we say that we will determine party-congenial beliefs based on whether the respondent is a Democrat or Republican.	We will use inferred ideology to determine congeniality rather than self-reported partisanship.	Maintains consistency with the rest of the PAP, where we use inferred ideology rather than self-reported partisanship.
12	In H4, we do not specify what wave we will use to measure candidate vote choice post-treatment	Some people did not complete the vote choice question in W4 but did complete it in W5. In these cases, we used their vote choice response from W5.	Clarification: Unlike with other outcome measures, we do not anticipate any 'decay' between W4 and W5 because the outcome measure in question is a behavior, so we pool across waves to ensure the maximum number of respondents.
14	In RQ1, we do not specify what wave we will use for self-reported turnout.	People who did not complete W4 received the turnout question in W5. We will pool those responses.	Clarification: Unlike with other outcome measures, we do not anticipate any 'decay' between W4 and W5 because the outcome measure in question is a behavior, so we will pool across waves to ensure the maximum number of respondents.
14	"Engagement with Voter Hub (click, like, comment, share, or reaction)"	Engagement with Voter Hub (click or view)	Engagement not logged in Facebook table
14	"Engagement with Town Hall (click, like, comment, share, or reaction)"	Engagement with Town Hall (click, follow or contact)	Townhall product currently only allows click, follow, or contact

(continued on next page)

PAP page	Original plan	Revised plan	Justification
14	"Sharing that you voted with others using 'Share You Voted' featured"	Not included	'Share Your Vote' product not used in 2020 election
15	In RQ1, we say "For each on-platform political participation measure, we will take the natural log of the ratio of the frequency of that metric to the frequency of views of civic content"	Instead, for each on-platform political participation measure, we will take the ratio of the natural log of the frequency of that metric to the frequency of views of civic content.	Typo in initial writeup
15	In RQ1, we say "We will then create a composite scale using exploratory factor analysis with varimax rotation to select items in each list above. Those that do not clearly load on the same underlying dimension (i.e., the first principal component) will be analyzed separately. We will construct a scale as the average of the standardized measures that are included."	Instead, we will use exploratory factor analysis with varimax rotation to select items, then analyze the factor scores of the resulting solution in up to three dimensions.	The large set of metrics in this measurement represent an eclectic set of platform actions. Because we did not have strong theoretical expectations about the underlying behavior(s) captured by these metrics, we will rely on the data to reveal what items load together and the relative importance of these factors.
18-19	The Wave 5 misinformation battery as pre-registered did not include the false, liberal-congenial item "Church bells rang in Paris to celebrate Joe Biden's victory."	The item will be included in the appropriate scales.	We inadvertently left this item out of the scale.
20	For 'issue polarization' under 'additional analyses,' we specify an index of standardized responses to issue opinion questions re-signed so that on each question higher values are closer to the congenial-party mean and lower values are closer to the non-congenial-party mean.	Clarifying that by 'congenial party' we mean party that is congenial with regard to the respondent's self-reported party identification.	Clarification: In order to facilitate comparisons across studies, we opted for the operationalization that most other studies were using (as opposed to inferred ideology, which is used by most of the other analyses in this paper).
23	"Behavioral data: Observational/descriptive: We will measure these for the full Facebook population and for both the treatment and control group pre-treatment (where relevant/applicable). These measures are for the 90 days prior to treatment otherwise specified."	We will run the analyses in this category for the full Facebook population, the treatment group, and the control group, at two time periods: before and during the experiment.	Clarification

(continued on next page)

PAP page	Original plan	Revised plan	Justification
23-24	"Mean/median/distribution (e.g., p2.5 p97.5) of proportion of friends that are like-minded as a proportion of the total number of friends (as of Sept. 22, 2020), Mean/median/distribution (e.g., p2.5 p97.5) of proportion of Pages/Groups followed that are like-minded as a proportion of the total number of Pages/Groups followed (as of Sept. 22, 2020), Mean/median/distribution (e.g., p2.5 p97.5) of proportion of Pages/Groups followed that are like-minded as a proportion of the total number of Pages/Groups followed (as of Sept. 22, 2020)"	Numbers as of September 23 instead of September 22	Intervention actually started on September 24, not September 23
23-24	All analyses in the 'Behavioral data: Observational/descriptive' section, including Exposure 1–6, Sources and characteristics 1–3, Behavior 1–2	Original analyses specified that we would examine exposure to, sources and characteristics of, and behavior towards like-minded content. For each of these analyses, we will also examine cross-cutting content.	Inadvertently left out of original PAP
24	"Mean proportion of incivility of posts viewed from like-minded Pages and Groups versus posts from Pages and Groups that are not like-minded"	Expand analysis to include users: so, mean proportion of incivility of posts viewed from like-minded Pages and Groups and users versus posts from Pages and groups and users that are not like-minded. (also note another deviation expands to cross-cutting as well)	Inadvertently left out of original PAP
24	"Mean proportion of incivility in comments on posts from like-minded (and cross-cutting) friends versus comments on posts from friends that are not like-minded (and cross-cutting)"	Mean percentage of uncivil comments that users make on the posts of like-minded (and cross-cutting) friends, groups, and Pages versus comments on posts from friends, groups, and Pages that are not like-minded (and cross-cutting). Pages and groups will be treated as the post creators for the purposes of determining like-minded/cross-cutting, not the page admin or person posting in the Group.	Clarifying operationalization of 'mean proportion' and adding Pages and groups (inadvertently left out).
24	"We designate all outcome measures listed above for the hypotheses and research questions as primary outcomes for the purposes of multiple comparisons testing."	We will treat all research question tests as secondary outcomes.	Many of the research questions have separate outcome measures, substantially inflating the number of primary tests. We also have weaker theoretical expectations for the research questions than for our hypotheses.

(continued on next page)

PAP page	Original plan	Revised plan	Justification
24	"All items that are excluded from preregistered indices or scales are considered to be primary outcomes."	We will report all items that are excluded from preregistered indices or scales as auxiliary tests reported only in an appendix with unadjusted p-values.	If these items don't load together with the other items, it might be because they are measured with error or maybe they were not good survey items after all. We don't have preregistered theoretical expectations for individual outcome measures that don't load with the other items in the scale. Finally, these individual outcome measures are less important in the context of the project. For example, there are no strong theoretical reasons to elevate e.g., 'congenial vote choice for Governor' or 'civic engagement with Town Hall product' (used by 0.3% of our sample) to the same level as affective polarization.
27	"Models estimating treatment effects for outcome variables that are measured in multiple survey waves will be estimated separately by wave."	We will combine items measured in multiple waves into a single measure that takes the average across waves (i.e., mean response among non-missing data for waves 4 and 5).	The debate over the election continued after Election Day, so we expect our treatment to continue to have meaningful effects on exposure to political content throughout that period.
28	"We estimate OLS regressions of the treatment indicator on covariates listed in the section on estimation above (in addition to dummy variables for blocks) and calculate a heteroskedasticity-robust Wald statistic for the hypothesis that all the coefficients on the covariates (excluding block dummies) are zero."	Clarification: We use a permutation test to calculate the p-value associated with the Wald statistic.	We follow the standard operating procedures for Don Green's lab at Columbia, which was the source of the original PAP language.
28	"Unless otherwise specified, for variables based on log data (either covariates or outcome variables), we recommend winsorizing at the 99th percentile in order to deal with possible outliers. This would mean recoding any values above the 99th percentile to the value that corresponds to the 99th percentile."	We only winsorize raw counts, not proportions.	Winsorization is only necessary to deal with outliers. By construction, proportions will not have outliers.

(continued on next page)

PAP page	Original plan	Revised plan	Justification
29	"Political sophistication: number of correct answers on political knowledge scale (0-2) and whether respondent correctly places Republicans running for office to the right of Democrats running for office (0/1) (Wave 4)"	Political sophistication should be measured using data from W2 rather than W4 so that it is pre-treatment	This was a typo.
29	"For observational descriptive analyses, we will analyze monthly active users; that is, users with L30>0, where L30 is defined as the number of days (over the last 30) that a user logged on the platform at least once."	We deviate from our preregistered approach to use L28>0 instead in Table 2, which compares Facebook activity over time between the respondent sample and the Facebook sample.	L28 is a better metric for this table because it captures the same number of days of each type (e.g., four Saturdays). Given strong weekly seasonality, that makes L30 more unstable over time. As preregistered, we still use L30 elsewhere to define the population of monthly active users because that is the metric that was used to construct the sampling frame for participants.
29	"The list of primary moderators we will test is listed below (we designate no secondary moderators). These will be treated as continuous for the purpose of analysis except for our binary measure of estimated political ideology."	Instead, we will operationalize these variables as discrete.	Adding weights requires the variables to be operationalized as discrete, and the categories also make the results easier to interpret.
30	We did not fully specify the pre-treatment handling of variables that did not load on post-treatment scales	In cases where a variable does not load on a post-treatment scale, that variable should also be excluded from the pre-treatment scale in which it is used as a control. In cases where a variable that is part of a scale occurs in only one of the two post-treatment waves, we will use only the subset that is common across both waves.	Clarification
32	"For all models, we will use the VIMP method for non-parametric estimation of variable importance (Williamson et al. 2020)."	We instead use the "linear" measure of VIMP, which simply tests the difference in mean squared error between a model incorporating all covariates and one that leaves out a particular moderator. In contrast to the non-parametric version, this approach requires assuming that the linear model is the correct specification of the CATE.	The fully non-parametric formulations we specified in the pre-analysis plan entail unacceptably large amounts of variance.

(continued on next page)

PAP page	Original plan	Revised plan	Justification
N/A	N/A		From the afternoon of November 2 until the morning of November 3 PST, the production servers that implemented the experimental treatment were unable to access the data table created for purposes of the study that stored the ideology scores of US users, Pages and groups. The Meta research team had received notice, as part of Facebook's data privacy efforts, that potential access to the table was wider than intended. Out of an abundance of caution prior to the election, the table was locked down, but server access was inadvertently blocked. Therefore, users in the treatment group who logged on during this brief period did not experience reduced exposure to like-minded content.
N/A	Not specified	In analyses that employ Facebook U.S. monthly active adult user data, we will exclude users who do not have an ideology classification.	The analyses in this paper are designed to understand exposure to and interaction with like-minded and cross-cutting content. If a user does not have an ideology classification, it is not possible to determine whether content they see is like-minded or cross-cutting. Note that about 95% of U.S. monthly active users have an ideology classification.
5	A domain with two or more strike URLs in the past 90 days is a domain repeat offender.	We exclude from this list of domains other social media platforms, hosting sites, and URL shorteners even if they may host URLs that have been fact-checked as false.	This allows us to avoid identifying as untrustworthy domains for which misinformation may represent a small proportion of their URLs.

(continued on next page)

PAP page	Original plan	Revised plan	Justification
N/A	Not specified	Misinformation ratings are as of February 15, 2021.	These ratings can change over time so it was necessary to define them as of a fixed date.

4.11 Survey questionnaire

Survey Questionnaires

Wave 1

**Client**	Facebook
**Project Name**	ERP 2020
**Project Number**	8870
**Survey length (median)**	10 minute survey
**Population**	CONSENTED FB/IG USERS
**Main**	N=309,243
**MODE**	CAWI WEB ONLY
**Language**	English/Spanish
**Sample Source**	Facebook Instagram recruited sample
**Incentive**	$0
**Survey description**	WAVE 1 ENROLLMENT Election and Politics Study 2020
**Eligibility Rate**	100%

LANGSWITCH.

Welcome to the 2020 Election Research Project
Bienvenidos al Proyecto de Investigación Electoral 2020

Let's get started with an easy question.
Empecemos con una pregunta fácil.

This survey is currently available in English and Spanish. Which language would you prefer to use to share your opinions?
Esta encuesta está actualmente disponible en inglés y en español. ¿Qué idioma prefiere usar para compartir sus opiniones?

1. English/Inglés
2. Spanish/Español

DISPLAY – OPTINTRO.
Thank you for enrolling in the **2020 Election Research Project**!
¡Gracias por inscribirse en el Proyecto de Investigación Electoral 2020!

This study is going to ask about your opinions, and will help researchers at New York University, The University of Texas at Austin, and other academic institutions, as well as Facebook, understand more about how people's experience with Facebook and Instagram affects their attitudes and behaviors concerning elections.
Este estudio va a pedir sus opiniones, y ayudará a los investigadores de la Universidad de Nueva York, la Universidad de Texas en Austin, y otras instituciones académicas, así como Facebook, a entender más acerca de cómo la experiencia de la gente con Facebook e Instagram afecta sus actitudes y comportamientos en relación con las elecciones.

After you complete the enrollment today, we will be sending you four more surveys between September and December. You'll be paid at least $30 for participating in this study and completing all four surveys.
Después de que complete la inscripción hoy, le enviaremos cuatro encuestas más entre septiembre y diciembre. Se le pagará al menos 30 dólares por participar en este estudio y completar las cuatro encuestas.

Let's get started! We ask for your help today to tell us about yourself.
¡Empecemos! Le pedimos su ayuda hoy para que nos hable de usted.

GENDER.
How do you describe yourself?
¿Cómo se describe a sí mismo?

RESPONSE OPTIONS:
1. Male
2. Female
3. I identify in some other way

1. Hombre
2. Mujer
3. Me identifico de otra manera

[FORCE RESPONSE: "Please tell us your age range. We require this information for your responses to be counted"/ "Por favor díganos su rango de edad. Esta información es necesaria para contar sus respuestas."]

AGE2.

Which of the following categories includes your current age?

¿Cuál de las siguientes categorías incluye su edad actual?

RESPONSE OPTIONS:

1. 17 or younger
2. 18 to 24
3. 25 to 34
4. 35 to 44
5. 45 to 54
6. 55 to 64
7. 65+

RESPONSE OPTIONS:

17 años o menos

1. 18 a 24
2. 25 a 34
3. 35 a 44
4. 45 a 54
5. 55 a 64
6. 65+

[IF AGE2<18, TERMINATE AND SET QUAL=2]

[custom prompt: "Information about any possible Hispanic ethnicity is very important. We greatly appreciate your response to this question."]

[custom prompt: "Información sobre cualquier posible etnia hispana es muy importante. Realmente apreciamos su respuesta a esta pregunta."]

HISPAN.

This question is about Hispanic ethnicity. Are you of Spanish, Hispanic, or Latino descent?

Esta pregunta se refiere a la etnia hispana. ¿Es usted de ascendencia española, hispana o latina?

RESPONSE OPTIONS:

1. No, I am not
   Yes, Mexican, Mexican-American, Chicano
2. Yes, Puerto Rican
3. Yes, Cuban
4. Yes, Central American
5. Yes, South American
6. Yes, Caribbean
7. Yes, Other Spanish/Hispanic/Latino

No, no soy

1. Sí, Mexicano/a, Mexico-americano/a, Chicano/a
2. Sí, Puertorriqueño/a
3. Sí, Cubano/a
4. Sí, Centroamericano/a
5. Sí, Sudamericano/a
6. Sí, Caribeño/a
7. Sí, otro Español/a, Hispano/a, Latino/a

RACE_1.
Please indicate what you consider your racial background to be. We greatly appreciate your help. The categories we use may not fully describe you, but they do match those used by the Census Bureau. It helps us to know how similar the group of participants is to the U.S. population.
Por favor, indique lo que considere que es su origen racial. Estamos muy agradecidos por su ayuda. Las categorías que utilizamos puede que no lo describan completamente a usted, pero sí que coinciden con las utilizadas por la Oficina del Censo. Nos ayuda a saber cuán similar es el grupo de participantes a la población de EE.UU.

Please check one or more categories below to indicate what <u>race or races</u> you consider yourself to be.
Por favor marque una o más de las siguientes categorías para indicar a qué <u>raza o razas</u> usted se considera pertenecer.

RESPONSE OPTIONS:

1 White
2 Black or African American
3 American Indian or Alaska Native – *Type in name of enrolled or principal tribe.* [TEXTBOX]
4 Asian Indian
5 Chinese
6 Filipino
7 Japanese
8 Korean
9 Vietnamese
10 Other Asian – *Type in race* [TEXTBOX]
11 Native Hawaiian
12 Guamanian or Chamorro
13 Samoan

14 Other Pacific Islander – *Type in race* [TEXTBOX]
15 Some other race – *Type in race* [TEXTBOX]

1 Blanca
2 Negra o Afroamericana
3 Indígena de las américas o nativa de Alaska–*Ingrese el nombre de la tribu en la cual está inscripto/a o tribu principal.* TEXTBOX]
4 India Asiática
5 China
6 Filipina
7 Japonesa
8 Coreana
9 Vietnamita
10 Otra asiática –*Escriba la raza*[TEXTBOX]
11 Nativa de Hawái
12 Guameña o Chamorra
13 Samoana
14 Otra de las islas del Pacífico – *Escriba la raza* [TEXTBOX]
15 Otra raza – *Escriba la raza* [TEXTBOX]

EDUCAT.
What is the highest level of school you have completed?
¿Cuál es el nivel escolar más alto que usted ha completado?

RESPONSE OPTIONS:

1. No formal education
2. 1st, 2nd, 3rd, or 4th grade
3. 5th or 6th grade
4. 7th or 8th grade
5. 9th grade
6. 10th grade
7. 11th grade
8. 12th grade no diploma
9. High school graduate – high school diploma or the equivalent (GED)
10. Some college, no degree
11. Associate degree
12. Bachelor’s degree
13. Master’s degree
14. Professional or Doctorate degree

1. Educación informal
2. 1º, 2º, 3º, ó 4º grado
3. 5º ó 6º grado
4. 7º ó 8º grado
5. 9º grado
6. 10º grado
7. 11º grado

8. 12º grado SIN DIPLOMA
9. Graduado de escuela secundaria – diploma de secundaria o su equivalente (GED)
10. Un poco de universidad, ningún título
11. Título de asociado
12. Licenciatura
13. Maestría
14. Título profesional o doctorado

INCOME.

The next question is about the total income of YOUR HOUSEHOLD for 2019. Please include your own income PLUS the income of all members living in your household (including cohabiting partners and armed forces members living at home). Please count income BEFORE TAXES and from all sources (such as wages, salaries, tips, net income from a business, interest, dividends, child support, alimony, and Social Security, public assistance, pensions, or retirement benefits).

La siguiente pregunta es sobre los ingresos totales de SU HOGAR en 2019. Por favor incluya sus propios ingresos MÁS los ingresos de todos los miembros que residen en su hogar (incluyendo a parejas cohabitantes y miembros de las fuerzas armadas que vivan en su hogar). Por favor cuente los ingresos ANTES DE LOS IMPUESTOS y de todas las fuentes (como sueldos, salarios, propinas, ingresos netos de un negocio, intereses, dividendos, manutención de hijos, pensión alimenticia, y Seguridad Social, asistencia pública, pensiones o prestaciones por jubilación).

RESPONSE OPTIONS:

1. Less than $5,000
2. $5,000 to $9,999
3. $10,000 to $14,999
4. $15,000 to $19,999
5. $20,000 to $24,999
6. $25,000 to $29,999
7. $30,000 to $34,999
8. $35,000 to $39,999
9. $40,000 to $49,999
10. $50,000 to $59,999
11. $60,000 to $74,999
12. $75,000 to $84,999
13. $85,000 to $99,999
14. $100,000 to $124,999
15. $125,000 to $149,999
16. $150,000 to $174,999
17. $175,000 to $199,999
18. $200,000 or more

1. Menos de $5,000
2. $5,000 a $9,999
3. $10,000 a $14,999
4. $15,000 a $19,999
5. $20,000 a $24,999
6. $25,000 a $29,999
7. $30,000 a $34,999
8. $35,000 a $39,999
9. $40,000 a $49,999
10. $50,000 a $59,999
11. $60,000 a $74,999
12. $75,000 a $84,999
13. $85,000 a $99,999
14. $100,000 a $124,999
15. $125,000 a $149,999
16. $150,000 a $174,999
17. $175,000 a $199,999
18. $200,000 o más

ZIP.
What is your ZIP Code?
¿Cuál es su código postal?

IDEO1.
How would you rate yourself on this scale?
¿Cómo se calificaría usted mismo en esta escala?

IF RND_01=0; SHOW 1-2-3-4-5
IF RND_01=1; SHOW 5-4-3-2-1:
ROTATE RESPONSE OPTIONS:
1. Very liberal
2. Somewhat liberal
3. Middle of the road
4. Somewhat conservative
5. Very conservative

ROTATE RESPONSE OPTIONS:
1. Muy liberal
2. Algo liberal
3. A la mitad del camino
4. Algo conservador
5. Muy conservador

PID.
Generally speaking, do you usually think of yourself as a Democrat, a Republican, an independent, or what?
En términos generales, ¿suele pensar en sí mismo como demócrata, republicano, independiente, o qué?

RESPONSE OPTIONS:

1. Democrat
2. Republican
3. Independent
4. Something else, please specify: [TEXTBOX]

1. Demócrata
2. Republicano/a
3. Independiente
4. Algo más, por favor especifique: [TEXTBOX]

[SHOW IF PID=1]
PIDSTRENGTH_D.
Would you call yourself a strong Democrat or a not very strong Democrat?
¿Se llamaría a sí mismo fuertemente demócrata, no muy fuertemente demócrata?

RESPONSE OPTIONS:

1. Strong Democrat
2. Not very strong Democrat
1. Completamente demócrata
2. No tan demócrata

[SHOW IF PID=2]
PIDSTRENGTH_R.
Would you call yourself a strong Republican or a not very strong Republican?
¿Se llamaría a sí mismo fuertemente republicano o no muy fuertemente republicano?

RESPONSE OPTIONS:

1. Strong Republican
2. Not very strong Republican

1. Completamente republicano
2. No tan republicano

[SHOW IF PID=3, 4, 77, 98, 99]
PIDLEAN.
Do you think of yourself as closer to the Republican Party or to the Democratic Party?
¿Se considera más cercano al Partido Republicano o al Partido Demócrata?

RESPONSE OPTIONS:
1. Closer to the Republican Party
2. Closer to the Democratic Party
3. Neither

RESPONSE OPTIONS:
1. Más cercano/a al Partido Republicano
2. Más cercano/a al Partido Demócrata
3. Ninguno de los dos\

VOTE16.
In 2016 Hillary Clinton ran on the Democratic ticket against Donald Trump for the Republicans. Do you remember for sure whether or not you voted in that election?
En 2016 Hillary Clinton se presentó en la candidatura Demócrata contra Donald Trump para los Republicanos. ¿Recuerda con seguridad si votó o no en esa elección?

RESPONSE OPTIONS:
1. Yes, voted
2. No, didn't vote

RESPONSE OPTIONS:
1. Sí, vote
2. No, no vote

[SHOW IF VOTE16=1]
CAND16.
Which candidate did you vote for?
¿Por qué candidato votó?

RESPONSE OPTIONS:
1. Hillary Clinton
2. Donald Trump
3. Other

RESPONSE OPTIONS:
1. Hillary Clinton
2. Donald Trump
3. Otro

[SHOW IF P_PLATFORM=2]
FBACCT_EVER.
Have you ever used Facebook?
¿Alguna vez ha usado Facebook?

RESPONSE OPTIONS:

1. Yes
2. No

RESPONSE OPTIONS:

1. Sí
2. No

[SHOW IF P_PLATFORM=1 OR FBACCT_EVER=1]
FBACCT_MULTIPLE.
How many Facebook accounts do you currently have?
¿Cuántas cuentas de Facebook tiene actualmente?

RESPONSES:

1. 1 account
2. 2 or more accounts
3. None

RESPONSES:

1. 1 cuenta
2. 2 o más cuentas
3. Ninguna

[SHOW IF FBACCT_MULTIPLE=1]
FBACCT_ACTIVE_ONE.
In the past 30 days, have you used your Facebook account?
En los últimos 30 días, ¿ha usado su cuenta de Facebook?

RESPONSES:

1. Yes
2. No

RESPONSES:

1. Sí
2. No

[SHOW IF FBACCT_MULTIPLE=2]
FBACCT_ACTIVE_MULTIPLE.
In the past 30 days, how many Facebook accounts have you used?
En los últimos 30 días, ¿cuántas cuentas de Facebook ha usado?

RESPONSES:
1. 1
2. 2
3. 3
4. 4
5. 5
6. 6 or more accounts
7. None

RESPONSES:
1. 1
2. 2
3. 3
4. 4
5. 5
6. 6 o más cuentas
7. Ninguna

[SHOW IF P_PLATFORM=1]
INSTACCT _EVER.
Have you ever used Instagram?
¿Ha usado alguna vez Instagram?

RESPONSES:
1. Yes
2. No

RESPONSES:
1. Sí
2. No

[SHOW IF P_PLATFORM=2 OR INSTACCT_EVER=1]
INSTACCT_MULTIPLE.
How many Instagram accounts do you currently have?
¿Cuántas cuentas Instagram tiene actualmente?

RESPONSES:

1. 1 account
2. 2 or more accounts
3. None

RESPONSES:

1. 1 cuenta
2. 2 o más cuentas
3. Ninguna

[SHOW IF INSTACCT_MULTIPLE=1]
INSTACCT_ACTIVE_ONE.
In the past 30 days, have you used your Instagram account?
En los últimos 30 días, ¿ha utilizado su cuenta Instagram?

RESPONSES:

1. Yes
2. No

RESPONSES:

1. Sí
2. No

[SHOW IF INSTACCT_MULTIPLE=2]
INSTACCT_ACTIVE_MULTIPLE.
In the past 30 days, how many Instagram accounts have you used?
En los últimos 30 días, ¿cuántas cuentas de Instagram ha utilizado?

RESPONSES:

1. 1
2. 2
3. 3
4. 4
5. 5
6. 6 or more accounts
7. None

RESPONSES:

1. 1
2. 2
3. 3
4. 4
5. 5
6. 6 o más cuentas
7. Ninguna

[DISPLAY_CONTACT]
So that we can send you rewards and our election surveys, we will be asking you for contact information. We will never share your information with third parties for marketing purposes or mailing lists.
Para poder enviarle los premios y nuestras encuestas electorales, le pediremos información de contacto. Nunca compartiremos su información con terceros para fines de marketing o listas de correo.

Let us explain why we need your email address. For you to participate in the 2020 Election Research Project, we need to be able to send you survey invitations and your rewards to an email address. Please provide your email address to participate in the study. We will use your email address only for the 2020 Election Research Project, and not for any other purposes.
Déjenos explicarle por qué necesitamos su dirección de correo electrónico. Para que usted participe en el Proyecto de Investigación Electoral 2020, necesitamos poder enviarle invitaciones a encuestas y sus premios a una dirección de correo electrónico. Por favor, proporcione su dirección de correo electrónico para participar en el estudio. Utilizaremos su dirección de correo electrónico solo par el Proyecto de Investigación Electoral 2020, y para ningún otro propósito.

We hope you will reconsider and will decide to provide your email address. Please enter your email address to make sure your voice is heard in the 2020 Election Research Project. We look forward to hearing about your opinions!
Esperamos que lo reconsidere y decida proporcionar su dirección de correo electrónico. Por favor, introduzca su dirección de correo electrónico para asegurarse de que su voz se oiga en el Proyecto de Investigación Electoral 2020. ¡Esperamos escuchar sus opiniones!

EMAIL1.
Please provide your name and an email address that we can use for sending you survey invitations and to receive your rewards.
Por favor proporcione su nombre y una dirección de correo electrónico que podamos usar para enviarle invitaciones a encuestas e información sobre sus premios.

First Name: [TEXTBOX] Last Name: [TEXTBOX]
Primer Nombre: [TEXTBOX] Apellido: [TEXTBOX]

Email Address: [TEXT BOX]
Dirección de correo electrónico: [TEXT BOX]

[MUST SELECT EMAIL_2=1]
EMAIL1_2.
Just to confirm: is this email correct?
Sólo para confirmar: ¿este correo electrónico es correcto?

EMAIL_2. [Pipe in response to EMAIL]

CAWI RESPONSE OPTIONS:

1. Yes
2. No
1. Sí
2. No

PHONE.
What will be the best contact phone number for you?
¿Cuál es el mejor número de teléfono para ponernos en contacto con usted?

Phone: [NUMBOX]
I don't want to provide my phone number
Teléfono:
No quiero dar mi número de teléfono

PHONE1_TYPE. Is this a landline phone or a cell phone?
¿Es este un teléfono fijo o un teléfono móvil?

RESPONSE OPTIONS:

1. Landline
2. Cell

1. Fijo
2. Celular

[SHOW IF PHONE=SHOWN AND PHONE1_TYPE=2]
TXTALERT.
The surveys in this study will only be available for a short time. If you'd like, we can send SMS text invitations and reminders to your cell phone.
Las encuestas de este estudio sólo estarán disponibles por un corto tiempo. Si lo desea, podemos enviarle invitaciones de texto SMS y recordatorios a su teléfono celular.

Can we send you text invitations, reminders, and notifications?
¿Podemos enviarle mensajes de texto con invitaciones, recordatorios y notificaciones?

By providing this number, you allow NORC to text you using an automated text system. Standard messaging and data rates may apply. We will only use your phone number for these research studies and will not share, sell or otherwise use this number unless you give us permission to do. You can reply STOP to our text messages to opt out at any time.

Al proporcionarnos este número, usted permite al NORC enviarle mensajes de texto mediante un sistema de mensajes automatizado. Pueden aplicarse tarifas estándar de mensajería y datos. Solo usaremos su número de teléfono para estos estudios de investigación y no lo compartiremos, venderemos o usaremos de otra manera a menos que usted nos dé permiso para hacerlo. Puede responder STOP a nuestros mensajes de texto para optar por no participar en cualquier momento.

RESPONSE OPTIONS:

1. Yes
2. No
3. I don’t have a cell phone

1. Sí
2. No
3. No tengo teléfono celular

[IF CAWI and selecting TXTALERT=1]
[TEXT_PHONE_CAWI]
We will be using the below number to send you SMS texts. Please review and change if necessary.
Usaremos el siguiente número para enviarle un mensaje de texto. Por favor, revíselo y modifíquelo si es necesario.

[FOR ANY CELL PHONE OR UNKNOWN TEL TYPE (IF PHONE=SHOWN AND PHONE1_TYPE=2,77,98,99)]

AUTOTEL
Do you authorize NORC to call you using an automated telephone dialing system for the following phone numbers you have just given to us?
Please note that we will only use your phone number for this study and will not share, sell or otherwise use these numbers without your prior consent. This feature simply allows our phone researchers to get connected to you faster rather than having to manually punch in the number for your cell. Once connected, an actual person will be speaking to you. So, this is **not** robocalling, which auto dials numbers with a prerecorded voice message.
¿Autoriza a NORC a llamarle usando un sistema de marcación telefónica automática para los siguientes números de teléfono que nos acaba de dar?
Por favor tenga en cuenta que sólo utilizaremos su número de teléfono para este estudio y no compartiremos, venderemos ni utilizaremos de ninguna otra forma estos números sin su consentimiento previo. Esta función simplemente permite a nuestros investigadores telefónicos conectarse a usted más rápido en lugar de tener que marcar manualmente el número de su celular. Una vez conectado, una persona real le hablará. Por lo tanto, esto **no** es robocalling, que marca automáticamente los números con un mensaje de voz pregrabado.

DISPLAY PHONE NUMBER

CAWI RESPONSE OPTIONS:

1. Yes
2. No

1. Sí
2. No

[SHOW IF CAWI-ONLY]
QFINAL3.
We are almost done.
Ya casi terminamos.

Which emoji best represents how you feel about completing the four surveys we are going to send you over the next few months?
¿Qué emoji representa mejor cómo se siente acerca de completar las cuatro encuestas que le enviaremos en los próximos meses?

FLIP RESPONSE OPTIONS:

1. 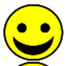
2. 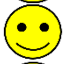
3. 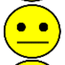
4. 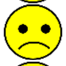
5. 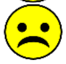

END.
Those are all the questions we have. The survey is now complete. Thank you! Please keep an eye out for an email in the next couple of days that will give you important additional information you need to continue with the rest of the study and start earning rewards. We will come back to you for the next survey in early September.
Estas son todas las preguntas que tenemos. La encuesta ya está completa. ¡Gracias! Por favor, esté atento a un correo electrónico en los próximos días que le dará información adicional importante que necesita para continuar con el resto del estudio y empezar a ganar premios. Volveremos a usted para la próxima encuesta a principios de septiembre.

[DISPLAY IF SAMPLE_GROUP = 1,2,3,4] As a member of the 2020 Election Research Project, you may be selected to participate in an additional study to learn more about the apps you use and sites you visit.
[DISPLAY IF SAMPLE_GROUP = 1,2,3,4] Como miembro del Proyecto de Investigación Electoral de 2020, es posible que sea seleccionado/a para participar en un estudio adicional para obtener más información sobre las aplicaciones que usted utiliza y los sitios que usted visita.

[DISPLAY IF SAMPLE_GROUP = 1,2,3,4] In the coming weeks, you may receive an invitation from NORC at erpStudy@norc.org to enroll in the 2020 Election Research Project Online Behavior Study. This study will help us understand more about how people are using the internet. Participants in the ERP Online Behavior Study can earn up to $90 for participation during the three month study.
[DISPLAY IF SAMPLE_GROUP = 1,2,3,4] En las próximas semanas, puede recibir una invitación de NORC en erpStudy@norc.org para inscribirse en el Estudio de Comportamiento en Línea del Proyecto de Investigación Electoral 2020. Este estudio nos ayudará a comprender mejor cómo las personas usan el Internet. Los participantes del Estudio de Comportamiento en Línea del Proyecto de Investigación Electoral pueden ganar hasta $90 por participar durante los tres meses del estudio.

[DISPLAY IF SAMPLE_GROUP = 1,2,3,4] Please be on the lookout for additional details about the study!
[DISPLAY IF SAMPLE_GROUP = 1,2,3,4] ¡Por favor, esté atento a los detalles adicionales sobre el estudio!

You can close your browser window now.
Ya puede cerrar la ventana del navegador.

Wave 2

**Client**	Facebook
**Project Name**	ERC 2020 Wave 2
**Project Number**	8870
**Survey length (median)**	25 minute survey
**Population**	CONSENTED FB/IG USERS, AmeriSpeak and ABS
**Main**	N=309,243 for FB/IG, n=11,000 for AmeriSpeak, n=9,300 for ABS
**MODE**	CAWI/CATI for ABS/AmeriSpeak, CAWI only for FB/IG
**Language**	English/Spanish
**Sample Source**	Facebook Instagram recruited sample, AmeriSpeak panel, ABS sample
**Incentive**	$5 regular/$10 late for FB/IG, $10 for ABS, $10 for AmeriSpeak
**Survey description**	Election and Politics Study 2020 Wave 2
**Eligibility Rate**	100%

Standard sample preloads

Variable Name	Include on Preload Testing-only page?	Variable Type	Variable Label
PANEL_TYPE	Y	Numeric	1 AmeriSpeak<br>2 Next Generation<br>3 GenF Extended (not in use)<br>4 AmeriSpeak Teen Panel<br>20 Lucid<br>21 SSI<br>22 ABS<br>23 FB/IG<br>50 Household 13-17<br>51 Household < 13<br>52 Household Adult

LANGSWITCH.

Welcome to the 2020 Election Research Project
Bienvenidos al Proyecto de Investigación Electoral 2020

Let's get started with an easy question.
Empecemos con una pregunta fácil.

This survey is currently available in English and Spanish. Which language would you prefer to use to share your opinions?
Esta encuesta está actualmente disponible en inglés y en español. ¿Qué idioma prefiere usar para compartir sus opiniones?

1. English/Inglés
2. Spanish/Español

[SHOW IF PANEL_TYPE=1,22,23]
DISPLAY – OPTINTRO.

[SHOW IF PANEL_TYPE=1,22
We're asking a small group of people what they think.
Estamos preguntando a un pequeño grupo de personas lo que piensan.

Your participation will help researchers at New York University, The University of Texas at Austin, and other academic institutions, as well as Facebook, understand more about how people's experience with Facebook and Instagram affects their opinions and behaviors concerning elections.
Su participación ayudará a los investigadores de la Universidad de Nueva York, la Universidad de Texas en Austin, y otras instituciones académicas, así como Facebook, a entender más acerca de cómo la experiencia de la gente con Facebook e Instagram afecta sus actitudes y comportamientos en relación con las elecciones.

We need all kinds of people to participate in the survey – both people who use social media and people who do not use social media.
Necesitamos que todo tipo de personas participe en la encuesta -- tanto la gente que usan los redes sociales como la gente que no use redes sociales.

We ask you to fill out this survey that will take about 20 minutes. Over the next three months, you'll be asked to take a short survey each month that will take about 15 minutes, for a total of about an hour of your time.]
Le pedimos que complete esta encuesta que le llevará unos 20 minutos. Durante los próximos tres meses, se le pedirá que haga una breve encuesta cada mes que le tomará unos 15 minutos, para un total de una hora de su tiempo.

[SHOW IF PANEL_TYPE=23
Thank you for your participation in the 2020 Election Research Project (ERP Study). Your participation helps researchers at New York University, The University of Texas at Austin, and other academic institutions, in partnership with Facebook, to learn more about the role of social media in elections in the United States.
Gracias por su participación en el Proyecto de Investigación Electoral 2020 (Estudio ERP). Su participación ayuda a los investigadores de la Universidad de Nueva York, la Universidad de Texas en Austin y otras instituciones académicas, en colaboración con Facebook, a aprender más sobre el papel de las redes sociales en las elecciones en los Estados Unidos.

We ask you to fill out this survey that will take about 20 minutes. After you complete the survey today, we will be sending you three more surveys between October and December. You'll be paid $5 for your participation in this survey and an additional $25 for completing the three follow up surveys.
Le pedimos que complete esta encuesta que le tomará unos 20 minutos. Después de que complete la encuesta hoy, les enviaremos tres encuestas más entre octubre y diciembre. Se le pagará 5 dólares por su participación en esta encuesta y 25 dólares adicionales por completar las tres encuestas de seguimiento.

Once this study is over, de-identified data will be stored and shared by Facebook for future research on elections, to validate the findings of this study, or if required by law for an inquiry by the Institutional Review Board (IRB) that reviewed this study.
Una vez que este estudio termine, los datos desidentificados serán almacenados y compartidos por Facebook para futuras investigaciones sobre las elecciones, para validar los resultados de este estudio, o si la ley lo requiere para una investigación de la Junta de Revisión Institucional (IRB) que revisó este estudio.

There are no benefits to participating in this research, nor are there risks greater than those encountered in everyday life, including risks related to the loss of confidentiality. Your participation is completely voluntary.]
No hay beneficios por participar en esta investigación, ni tampoco hay riesgos mayores que los que se encuentran en la vida cotidiana, incluyendo los riesgos relacionados con la pérdida de confidencialidad. Su participación es completamente voluntaria.]

[[SHOW IF PANEL TYPE=1]
You’ll be paid [INCENTWCOMMA] for participating in this and you will receive a bonus of 15,000 AmeriPoints after completing all four surveys.
Se le pagará [INCENTWCOMMA] por participar en esto y recibirá un bono de 15,000 AmeriPoints después de completar las cuatro encuestas.

[SHOW IF PANEL TYPE=22]
You’ll be paid $40 for participating in this study by completing all four surveys, including $10 after completing each survey.
Se le pagarán 40 dólares por participar en este estudio al completar las cuatro encuestas, incluyendo 10 dólares después de completar cada encuesta.

Once this study is over, de-identified data will be stored and shared by Facebook for future research on elections, to validate the findings of this study, or if required by law for an inquiry by the Institutional Review Board (IRB) that reviewed this study.
Una vez que este estudio termine, los datos desidentificados serán almacenados y compartidos por Facebook para futuras investigaciones sobre las elecciones, para validar los resultados de este estudio o, si la ley lo requiere, para una investigación de la Junta de Revisión Institucional (IRB) que revisó este estudio.

There are no benefits to participating in this research, nor are there risks greater than those encountered in everyday life, including risks related to the loss of confidentiality. Your participation is completely voluntary.
No hay beneficios por participar en esta investigación, ni tampoco hay riesgos mayores que los que se encuentran en la vida cotidiana, incluyendo los riesgos relacionados con la pérdida de la confidencialidad. Su participación es completamente voluntaria.

[[SHOW IF PANEL TYPE=1]
You may withdraw at any time by emailing support@amerispeak.org or calling toll-free (888) 326-9424.
Puede retirarse en cualquier momento enviando un correo electrónico a ayuda@amerispeak.org o llamando al número gratuito (888) 326-9424.

[SHOW IF PANEL TYPE=22]
You may withdraw at any time by visiting 2020erp.norc.org, by emailing erpSurvey@norc.org or by calling toll-free (877) 839-1505.
Puede retirarse en cualquier momento visitando 2020erp.norc.org, enviando un correo electrónico a erpSurvey@norc.org o llamando al teléfono gratuito (877) 839-1505.

[SHOW IF PANEL TYPE=23]
You may withdraw at any time by visiting 2020erp.norc.org, by emailing erpStudy@norc.org or by calling toll-free (866) 270-2602
Puede retirarse en cualquier momento visitando 2020erp.norc.org, enviando un correo electrónico a erpStudy@norc.org o llamando al teléfono gratuito (866) 270-2602

Let's get started! We ask for your help today to tell us about yourself.
¡Empecemos! Le pedimos su ayuda hoy para que nos hable de usted.

[SHOW IF PANEL_TYPE=22]
GENDER.
How do you describe yourself?
¿Cómo se describe a sí mismo?

CAWI RESPONSE OPTIONS:

1. Male
2. Female
3. I identify in some other way

1. Hombre
2. Mujer

3. Me identifico de alguna otra manera

CAWI RESPONSE OPTIONS:

1. Male
2. Female
3. You identify in some other way

1. Hombre
2. Mujer
3. Se identifica de alguna otra manera

1.
[SHOW IF PANEL TYPE=22,23]
DOB
What is your date of birth?
¿Cuál es su fecha de nacimiento?

We ask for your date of birth so that we can group your responses with others who are about your age.
If you do not feel comfortable providing your full birthday, please provide the year.
Le preguntamos su fecha de nacimiento para agrupar sus respuestas con las de personas de aproximadamente su misma edad.
Si no se siente cómodo dando su cumpleaños completo, por favor proporciona el año.

___ ___ / ___ ___ / ___ ___ ___ ___
M M D D Y Y Y Y
Mes(mm) / Dia(DD) / Año(AAAA)

[IF PANEL_TYPE=23 AND DOB_YYYY > 2002 AFTER PROMPT, TERMINATE AND SET QUAL=2]
2.

[SHOW IF PANEL_TYPE=22 AND DOB_YYYY>2002]

AGE2.
Which of the following categories includes your current age?
¿Cuál de las siguientes categorías incluye su edad actual?

RESPONSE OPTIONS:

8. 17 or younger
9. 18 to 24
10. 25 to 34
11. 35 to 44
12. 45 to 54
13. 55 to 64
14. 65+

RESPONSE OPTIONS:

8. 17 años o menos
9. 18 a 24
10. 25 a 34
11. 35 a 44
12. 45 a 54
13. 55 a 64
14. 65+

[IF AGE2=1,77,98,99, TERMINATE AND SET QUAL=2]

3.

4.

TERMSORRY.

[SHOW IF PANEL_TYPE=22,23]

Thank you for your interest in our study about the upcoming election. At this time, it does not appear that you are a match to join this study.

Gracias por su interés en nuestro estudio sobre las próximas elecciones. En este momento, no parece que usted sea compatible para unirse a este estudio.

5.

[SHOW IF PANEL_TYPE=22]

6.

HISPAN.

This question is about Hispanic ethnicity. Are you of Spanish, Hispanic, or Latino descent?

Esta pregunta se refiere a la etnia hispana. ¿Es usted de ascendencia española, hispana o latina?

RESPONSE OPTIONS:

1. [CAWI: No, I am not [CATI: No, you are not]
2. Yes, Mexican, Mexican-American, Chicano
3. Yes, Puerto Rican
4. Yes, Cuban
5. Yes, Central American
6. Yes, South American
7. Yes, Caribbean
8. Yes, Other Spanish/Hispanic/Latino

1. [CAWI: No, no soy [CATI: No, no lo eres]
2. Sí, Mexicano/a, Mexico-americano/a, Chicano/a
3. Sí, Puertorriqueño/a
4. Sí, Cubano/a
5. Sí, Centroamericano/a
6. Sí, Sudamericano/a
7. Sí, Caribeño/a
8. Sí, otro Español/a, Hispano/a, Latino/a

[SHOW IF PANEL_TYPE=22]

RACE_1.
Please indicate what you consider your racial background to be. We greatly appreciate your help. The categories we use may not fully describe you, but they do match those used by the Census Bureau. It helps us to know how similar the group of participants is to the U.S. population.
Por favor, indique lo que considere que es su origen racial. Estamos muy agradecidos por su ayuda. Las categorías que utilizamos puede que no lo describan completamente a usted, pero sí que coinciden con las utilizadas por la Oficina del Censo. Nos ayuda a saber cuán similar es el grupo de participantes a la población de EE.UU.

[CAWI: Please check one or more categories below to indicate][CATI: Please tell me] what race or races you consider yourself to be.
[CAWI: Por favor marque una o más de las siguientes categorías para indicar][CATI: Por favor, dígame]a qué raza o razas usted se considera pertenecer.

RESPONSE OPTIONS:

1 White
2 Black or African American
3 American Indian or Alaska Native – *Type in name of enrolled or principal tribe* [TEXTBOX]
4 Asian Indian
5 Chinese
6 Filipino
7 Japanese
8 Korean
9 Vietnamese
10 Other Asian – *Type in race* [TEXTBOX]
11 Native Hawaiian
12 Guamanian or Chamorro
13 Samoan
14 Other Pacific Islander – *Type in race* [TEXTBOX]
15 Some other race – *Type in race* [TEXTBOX]

1 Blanca
2 Negra o Afroamericana
3Indígena de las américas o nativa de Alaska – *Ingrese el nombre de la tribu en la cual está inscripto/a o tribu principal.* [TEXTBOX]
4 India Asiática
5 China
6 Filipina
7 Japonesa
8 Coreana
9 Vietnamita
10 Otra asiática – *Escriba la raza*[TEXTBOX]
02 Nativa de Hawái
12 Guameña o Chamorra

13 Samoana
14 Otra de las islas del Pacífico – *Escriba la raza* [TEXTBOX]
15 Otra raza – *Escriba la raza* [TEXTBOX]

[SHOW IF PANEL_TYPE=22]

EDUCAT.
What is the highest level of school you have completed?
¿Cuál es el nivel escolar más alto que usted ha completado?
RESPONSE OPTIONS:

1. No formal education
2. 1st, 2nd, 3rd, or 4th grade
3. 5th or 6th grade
4. 7th or 8th grade
5. 9th grade
6. 10th grade
7. 11th grade
8. 12th grade no diploma
9. High school graduate – high school diploma or the equivalent (GED)
10. Some college, no degree
11. Associate degree
12. Bachelor's degree
13. Master's degree
14. Professional or Doctorate degree

7.

1. Educación informal
2. 1º, 2º, 3º, ó 4º grado
3. 5º ó 6º grado
4. 7º ó 8º grado
5. 9º grado
6. 10º grado
7. 11º grado
8. 12º grado SIN DIPLOMA
9. Graduado de escuela secundaria – diploma de secundaria o su equivalente (GED)
10. Un poco de universidad, ningún título
11. Título de asociado
12. Licenciatura
13. Maestría
14. Título profesional o doctorado

[SHOW IF PANEL_TYPE=22]

INCOME.
The next question is about the total income of your household for 2019. Please include your own income plus the income of all members living in your household (including cohabiting partners and armed forces members living at home). Please count income before taxes and from all sources (such as wages, salaries, tips, net income from a business, interest, dividends, child support, alimony, and Social Security, public assistance, pensions, or retirement benefits).
La siguiente pregunta es sobre los ingresos totales de su hogar en 2019. Por favor incluya sus propios ingresos más los ingresos de todos los miembros que residen en su hogar (incluyendo a parejas cohabitantes y miembros de las fuerzas armadas que vivan en su hogar). Por favor cuente los ingresos antes de los impuestos y de todas las fuentes (como sueldos, salarios, propinas, ingresos netos de un negocio, intereses, dividendos, manutención de hijos, pensión alimenticia, y Seguridad Social, asistencia pública, pensiones o prestaciones por jubilación).
[CATI:

What was the total income of your household in 2019?
¿Cuál fue el ingreso total de su hogar en 2019?]

RESPONSE OPTIONS:
1. Less than $5,000
2. $5,000 to $9,999
3. $10,000 to $14,999
4. $15,000 to $19,999
5. $20,000 to $24,999
6. $25,000 to $29,999
7. $30,000 to $34,999
8. $35,000 to $39,999
9. $40,000 to $49,999
10. $50,000 to $59,999
11. $60,000 to $74,999
12. $75,000 to $84,999
13. $85,000 to $99,999
14. $100,000 to $124,999
15. $125,000 to $149,999
16. $150,000 to $174,999
17. $175,000 to $199,999
18. $200,000 or more

1. Menos de $5,000
2. $5,000 a $9,999
3. $10,000 a $14,999
4. $15,000 a $19,999
5. $20,000 a $24,999
6. $25,000 a $29,999
7. $30,000 a $34,999
8. $35,000 a $39,999

9. $40,000 a $49,999
10. $50,000 a $59,999
11. $60,000 a $74,999
12. $75,000 a $84,999
13. $85,000 a $99,999
14. $100,000 a $124,999
15. $125,000 a $149,999
16. $150,000 a $174,999
17. $175,000 a $199,999
18. $200,000 o más

[SHOW IF PANEL_TYPE=22]
8. **Z**
IP.
9. W
hat is your ZIP Code?
10. ¿
Cuál es su código postal?
11.

[SHOW IF PANEL_TYPE=1,22]

IDEO1.
How would you rate yourself on this scale?
¿Cómo se calificaría usted mismo en esta escala?

IF RND_01=0; SHOW 1-2-3-4-5
IF RND_01=1; SHOW 5-4-3-2-1:
RESPONSE OPTIONS:
1. Very liberal
2. Somewhat liberal
3. Middle of the road
4. Somewhat conservative
5. Very conservative

RESPONSE OPTIONS:
1. Muy liberal
2. Algo liberal
3. A mitad de camino
4. Algo conservador
5. Muy conservador

[SHOW IF PANEL_TYPE=1,22]

PID.
Generally speaking, do you usually think of yourself as a Democrat, a Republican, an independent, or what?
En términos generales, ¿suele pensar en sí mismo como demócrata, republicano, independiente, o qué?

RESPONSE OPTIONS:
1. Democrat
2. Republican
3. Independent
4. Something else, please specify: [TEXTBOX]

1. Demócrata
2. Republicano/a
3. Independiente
4. Algo más, por favor especifique: [TEXTBOX]

[SHOW IF PID=1]

PIDSTRENGTH_D.
Would you call yourself a strong Democrat or a not very strong Democrat?
¿Se llamaría a sí mismo fuertemente demócrata, no muy fuertemente demócrata?

RESPONSE OPTIONS:
1. Strong Democrat
2. Not very strong Democrat

1. Fuertemente demócrata
2. No tan demócrata

[SHOW IF PID=2]

PIDSTRENGTH_R.
Would you call yourself a strong Republican or a not very strong Republican?
¿Se llamaría a sí mismo fuertemente republicano o no muy fuertemente republicano?

RESPONSE OPTIONS:
1. Strong Republican
2. Not very strong Republican

1. Fuertemente republicano
2. No tan republicano

[SHOW IF PID=3, 4, 77, 98, 99]

PIDLEAN.
Do you think of yourself as closer to the Republican Party or to the Democratic Party?
¿Se considera más cercano al Partido Republicano o al Partido Demócrata?

RESPONSE OPTIONS:
1. Closer to the Republican Party
2. Closer to the Democratic Party
3. Neither

RESPONSE OPTIONS:
1. Más cercano/a al Partido Republicano
2. Más cercano/a al Partido Demócrata
3. Ninguno de los dos

[SHOW IF PANEL_TYPE=1,22]

VOTE16.
In 2016 Hillary Clinton ran on the Democratic ticket against Donald Trump for the Republicans. Do you remember for sure whether or not you voted in that election?
En 2016 Hillary Clinton se presentó en la candidatura demócrata contra Donald Trump para los republicanos. ¿Recuerda con seguridad si votó o no en esa elección?

CAWI RESPONSE OPTIONS:
1. Yes, voted
2. No, didn't vote

CAWI RESPONSE OPTIONS:
1. Sí, voté
2. No, no voté

CATI RESPONSE OPTIONS:
1. YES, VOTED
2. NO, DIDN'T VOTE

CATI RESPONSE OPTIONS:
1. SI, VOTÉ
2. NO, NO VOTÉ

[SHOW IF VOTE16=1]

CAND16.
Which candidate did you vote for?
¿Por qué candidato votó?

CAWI RESPONSE OPTIONS:
1. Hillary Clinton
2. Donald Trump
3. Other

CAWI RESPONSE OPTIONS:
1. Hillary Clinton
2. Donald Trump
3. Otro

CATI RESPONSE OPTIONS:
1. HILLARY CLINTON
2. DONALD TRUMP
3. OTHER

CATI RESPONSE OPTIONS:
1. HILLARY CLINTON
2. DONALD TRUMP
3. OTRO

[SHOW IF PANEL_TYPE=1,22]

FBACCT_EVER.
Have you ever used Facebook?
¿Alguna vez ha usado Facebook?

CAWI RESPONSE OPTIONS:
1. Yes
2. No
1. Sí
2. No

CATI RESPONSE OPTIONS:
1. YES
2. NO
1. SÍ
2. NO

[SHOW IF FBACCT_EVER=1]

FBACCT_MULTIPLE.
How many Facebook accounts do you currently have?
¿Cuántas cuentas de Facebook tiene actualmente?

RESPONSES:

1. 1 account
2. 2 or more accounts
3. None

1. 1 cuenta
2. 2 o más cuentas
3. Ninguna

[SHOW IF FBACCT_MULTIPLE=1]

FBACCT_ACTIVE_ONE.
In the past 30 days, have you used your Facebook account?
En los últimos 30 días, ¿ha usado su cuenta de Facebook?

CAWI RESPONSES:

1. Yes
2. No

1. Sí
2. No

CATI RESPONSE OPTIONS:

1. YES
2. NO

1. SÍ
2. NO

[SHOW IF FBACCT_MULTIPLE=2]

FBACCT_ACTIVE_MULTIPLE.
In the past 30 days, how many Facebook accounts have you used?
En los últimos 30 días, ¿cuántas cuentas de Facebook ha usado?

RESPONSES:

1. 1
2. 2
3. 3
4. 4
5. 5
6. 6 or more accounts

7. None
1. 1
2. 2
3. 3
4. 4
5. 5
6. 6 o más cuentas
7. Ninguna

[SHOW IF PANEL_TYPE=1,22]

INSTACCT _EVER.
Have you ever used Instagram?
¿Ha usado alguna vez Instagram?

CAWI RESPONSES:
1. Yes
2. No
1. Sí
2. No

CATI RESPONSE OPTIONS**:**
1. YES
2. NO
1. SÍ
2. NO

[SHOW IF INSTACCT_EVER=1]

INSTACCT_MULTIPLE.
How many Instagram accounts do you currently have?
¿Cuántas cuentas de Instagram tiene actualmente?

RESPONSES:
1. 1 account
2. 2 or more accounts
3. None
1. 1 cuenta
2. 2 o más cuentas
3. Ninguna

[SHOW IF INSTACCT_MULTIPLE=1]

INSTACCT_ACTIVE_ONE.

In the past 30 days, have you used your Instagram account?
En los últimos 30 días, ¿ha utilizado su cuenta Instagram?

CAWI RESPONSES:

1. Yes
2. No
1. Sí
2. No

CATI RESPONSE OPTIONS:

1. YES
2. NO
1. SÍ
2. NO

[SHOW IF INSTACCT_MULTIPLE=2]

INSTACCT_ACTIVE_MULTIPLE.
In the past 30 days, how many Instagram accounts have you used?
En los últimos 30 días, ¿cuántas cuentas de Instagram ha utilizado?

RESPONSES:

1. 1
2. 2
3. 3
4. 4
5. 5
6. 6 or more accounts
7. None
1. 1
2. 2
3. 3
4. 4
5. 5
6. 6 o más cuentas
7. Ninguna

CREATE DOV_FB_USER
IF FBACCT_ACTIVE_ONE=1 OR FBACCT_ACTIVE_MULTIPLE=1-6, DOV_FB_USER=1
ELSE DOV_FB_USER=0.

CREATE DOV_IG_USER
IF INSTACCT_ACTIVE_ONE=1 OR INSTACCT_ACTIVE_MULTIPLE=1-6, DOV_IG_USER=1
ELSE DOV_IG_USER=0.

SHOW DOV_FB_USER AND DOV_IG_USER ON TESTING ONLY SCREEN

DISPLAY_MEDIA.
[INSERT IF PANEL_TYPE=1,22: Now][INSERT IF PANEL_TYPE=23: First] we have some questions about your media use.
[INSERT IF PANEL_TYPE=1,22: Ahora][INSERT IF PANEL_TYPE=23: Primero] tenemos algunas preguntas sobre su uso de los medios.

POLINFO_SOURCE.
How often in the past week have you gotten political information from the following sources?
¿Con qué frecuencia en la última semana ha obtenido información política de las siguientes fuentes?

[CATI: TI INSTRUCTIONS: Read response options out loud as: "A-B-C", "C-B-S", "N-B-C", "Fox", "M-S-N-B-C", "C-N-N", "N-P-R".]

GRID ITEMS, RANDOMIZE:

A. National network TV news like ABC, CBS, or NBC
B. Print newspapers
C. Online news websites
D. Local TV news
E. Facebook
F. Instagram
G. Twitter
H. FOX News
I. MSNBC
J. CNN
K. Talk radio programs like Sean Hannity or Rush Limbaugh
L. Public radio/NPR
M. Friends and family
N. YouTube
A. Noticias de televisión nacional como ABC, CBS, or NBC
B. Periódico impreso
C. Sitios web de noticias en línea
D. Noticias de la televisión local
E. Facebook
F. Instagram
G. Twitter
H. Noticias FOX
I. MSNBC
J. CNN
K. Los programas de radio como Sean Hannity o Rush Limbaugh
L. Radio público/NPR
M. Amigos y familiares
N. YouTube

IF RND_01=0 1,2,3,4
IF RND_01=1 4,3,2,1
RESPONSE OPTIONS:

1. Every day
2. Several times
3. Once
4. Never
1. Todos los dĺas
2. Varias veces
3. Una vez
4. Nunca

INFOTRUST_SOURCE.
How much do you think political information from each of these sources can be trusted?
¿Cuánto cree usted que se puede confiar en la información política de cada una de estas fuentes?

GRID ITEMS, RANDOMIZE:

A. Local news
B. National newspapers
C. Facebook
D. Instagram
E. Twitter
F. National network TV news like ABC, CBS, or NBC
G. MSNBC
H. CNN
I. FOX News
A. Noticias locales
B. Periódicos nationales
C. Facebook
D. Instagram
E. Twitter
F. Noticias de televisión nacional como ABC, CBS, o NBC
G. MSNBC
H. CNN
I. Noticias FOX

IF RND_01=0 1,2,3,4,5
IF RND_01=1 5,4,3,2,1
RESPONSE OPTIONS:

1. Not at all
2. A little
3. A moderate amount
4. A lot
5. A great deal

1. Nada
2. Un poco
3. Algo
4. Mucho
5. Muchísimo

DISPLAY_POL.
Next [IF CAWI:we, IF CATI:I] have some questions about your interest in politics.
A continuación [IF CAWI:tenemos, IF CATI:tengo] algunas preguntas sobre su interés en la política.

POLINT.
How often do you pay attention to what's going on in government and politics?
¿Con qué frecuencia presta atención a los asuntos del gobierno y de la política?

IF RND_01=0 1,2,3,4,5
IF RND_01=1 5,4,3,2,1
RESPONSE OPTIONS:
1. Always
2. Most of the time
3. About half the time
4. Some of the time
5. Never
1. Siempre
2. La mayoría del tiempo
3. Casi la mitad del tiempo
4. Algunas veces
5. Nunca

POLPART.
During the past month, have you done any of the following?
Durante el pasado mes , ¿ha hecho algo de lo siguiente?

[CAWI - remove bold] *Select all that apply.*
[CAWI - remove bold] *Seleccione todos los que correspondan.*
[CATI] SELECT ALL THAT APPLY.
[CATI] SELECCIONE TODOS LOS QUE CORRESPONDAN.

RESPONSE OPTIONS, RANDOMIZE:
1. Attended a protest or rally
2. Contributed money to a political candidate or organization
3. Signed an online petition
4. Tried to convince someone how to vote (online or in-person)

5. Wrote and posted political messages online
6. Talked about politics with someone you know
7. None of the above [ANCHOR]
1. Asistió a una protesta o a un mitin
2. Contribuyó dinero a un candidato u organización política
3. Firmó una petición en línea
4. Trató de convencer a alguien de cómo votar (en línea o en persona)
5. Escribió y publicó mensajes políticos en línea
6. Habló de política con alguien que conoce
7. Ninguno de los anteriores [ANCHOR]

EPE1.
Do you agree or disagree with the following statements?
¿Está de acuerdo o en desacuerdo con las siguientes declaraciones?

[CAWI: I][CATI: You] feel confident that [CAWI: I][CATI: you] can find the truth about political issues.
[CAWI: Me siento][CATI: Se siente] seguro de que [CAWI: puedo][CATI: puede] encontrar la verdad sobre los asuntos políticos.

[CATI] IF R SAYS AGREE: Is that agree strongly or agree somewhat?
[CATI] IF R SAYS DISAGREE: Is that disagree strongly or disagree somewhat?
[CATI] IF R SAYS AGREE: ¿Está completamente de acuerdo o algo de acuerdo?
[CATI] IF R SAYS DISAGREE: ¿Está completamente en desacuerdo o algo en desacuerdo?

IF RND_01=0 1,2,3,4,5
IF RND_01=1 5,4,3,2,1
CAWI RESPONSE OPTIONS:
1. Agree strongly
2. Agree somewhat
3. Neither agree nor disagree
4. Disagree somewhat
5. Disagree strongly
1. Completamente de acuerdo
2. Algo de acuerdo
3. Ni de acuerdo ni en desacuerdo
4. Algo en desacuerdo
5. Completamente en desacuerdo

IF RND_01=0 1,2,3,4,5
IF RND_01=1 5,4,3,2,1
CATI RESPONSE OPTIONS:
1. AGREE STRONGLY
2. AGREE SOMEWHAT
3. NEITHER AGREE NOR DISAGREE
4. DISAGREE SOMEWHAT
5. DISAGREE STRONGLY

1. COMPLETAMENTE DE ACUERDO
2. ALGO DE ACUERDO
3. NI DE ACUERDO NI EN DESACUERDO
4. ALGO EN DESACUERDO
5. COMPLETAMENTE EN DESACUERDO

EPE2.
Do you agree or disagree with the following statements?
¿Está de acuerdo o en desacuerdo con las siguientes declaraciones?

If [CAWI: I][CATI: you] wanted to, [CAWI: I][CATI: you] could figure out the facts behind most political disputes.
Si [CAWI: yo][CATI: usted] quisiera, [CAWI: yo][CATI: usted] podría averiguar los hechos detrás de la mayoría de las disputas políticas.
[CATI] IF R SAYS AGREE: Is that agree strongly or agree somewhat?
[CATI] IF R SAYS DISAGREE: Is that disagree strongly or disagree somewhat?
[CATI] IF R SAYS AGREE: ¿Está completamente de acuerdo o algo de acuerdo?
[CATI] IF R SAYS DISAGREE: ¿Está completamente en desacuerdo o algo en desacuerdo?

IF RND_01=0 1,2,3,4,5
IF RND_01=1 5,4,3,2,1
CAWI RESPONSE OPTIONS:
1. Agree strongly
2. Agree somewhat
3. Neither agree nor disagree
4. Disagree somewhat
5. Disagree strongly
1. Completamente de acuerdo
2. Algo de acuerdo
3. Ni de acuerdo ni en desacuerdo
4. Algo en desacuerdo
5. Completamente en desacuerdo

CATI RESPONSE OPTIONS:
1. AGREE STRONGLY
2. AGREE SOMEWHAT
3. NEITHER AGREE NOR DISAGREE
4. DISAGREE SOMEWHAT
5. DISAGREE STRONGLY
1. COMPLETAMENTE DE ACUERDO
2. ALGO DE ACUERDO
3. NI DE ACUERDO NI EN DESACUERDO
4. ALGO EN DESACUERDO
5. COMPLETAMENTE EN DESACUERDO

DISPLAY_ELECT.

Now, [IF CAWI:we, IF CATI:I] have several questions about the election this November.
Ahora, [IF CAWI:tenemos, IF CATI:tengo] varias preguntas sobre la elección de noviembre.

VOTE_LIKELY.
How likely are you to vote in the general election this November?
¿Qué probabilidad hay de que vote en las elecciones generales de noviembre?

IF RND_01=0 1,2,3,4
IF RND_01=1 4,3,2,1
RESPONSE OPTIONS:

1. Definitely will vote
2. Probably will vote
3. Probably will not vote
4. Definitely will not vote
1. Definitivamente votará
2. Probablemente votará
3. Probablemente no votará
4. Definitivamente no votará

---

reg.
Are you now registered to vote, or are you not registered? [CATI: If you are not sure, you can say that too.]
¿Está usted registrado para votar o actualmente no está registrado? [CATI: Si no está seguro, también puede decir eso.]

CAWI RESPONSE OPTIONS:

1. Registered
2. Not registered
77. Not sure
1. Registrado
2. No registrado
77. No estoy seguro

CAtI RESPONSE OPTIONS:

1. REGISTERED
2. NOT REGISTERED
77. NOT SURE
1. REGISTRADO
2. NO REGISTRADO
77. NO ESTOY SEGURO

VOTE_PREELEC.

We'd like to ask you about the election for President to be held on November 3, in which [SHOW IF RND_00=0: Joe Biden is running against Donald Trump; SHOW IF RND_00=1: Donald Trump is running against Joe Biden]. Which candidate do you prefer for President of the United States?

Ahora nos gustariá preguntarle sobre la elección para Presidente que se celebrará el 3 de noviembre, en la que [SHOW IF RND_00=0: Joe Biden se está postulando contra Donald Trump; SHOW IF RND_00=1: Donald Trump se está postulando contra Joe Biden]. ¿Qué candidato prefiere para Presidente de los Estados Unidos?

SHOW IF RND_00=0:
RESPONSE OPTIONS:

1. Joe Biden (Democrat)
2. Donald Trump (Republican)
3. Jo Jorgensen (Libertarian)
4. Howie Hawkins (Green)
5. Other candidate, please specify: [TEXTBOX]
77. Not sure
1. Joe Biden (demócrata)
2. Donald Trump (republicano)
3. Jo Jorgensen (libertario)
4. Howie Hawkins (verde)
5. Otro candidato, por favor especifique: [TEXTBOX]
77. No estoy seguro

SHOW IF RND_00=1:
RESPONSE OPTIONS:

2. Donald Trump (Republican)
1. Joe Biden (Democrat)
3. Jo Jorgensen (Libertarian)
4. Howie Hawkins (Green)
5. Other candidate, please specify: [TEXTBOX]
77. Not sure
2. Donald Trump (republicano)
1. Joe Biden (demócrata)
3. Jo Jorgensen (libertario)
4. Howie Hawkins (verde)
5. Otro candidato, por favor especifique: [TEXTBOX]
77. No estoy seguro

APPROVAL.

How much do you approve or disapprove of the way Donald Trump is handling his job as president?

¿Cuánto aprueba o desaprueba la manera en que Donald Trump está haciendo su trabajo como presidente?

IF RND_01=0 1,2,3,4,5
IF RND_01=1 5,4,3,2,1

RESPONSE OPTIONS:

1. Strongly approve
2. Somewhat approve
3. Neither approve nor disapprove
4. Somewhat disapprove
5. Strongly disapprove
1. Aprueba totalmente
2. Aprueba de alguna manera
3. Ni aprueba ni desaprueba
4. Desaprueba de alguna manera
5. Desaprueba totalmente

DISPLAY_PERCEPT.

The next set of questions asks about your perceptions of various people and groups.

El siguiente serie de preguntas se refiere a sus percepciones de varias personas y grupos.

FT_PEOPLEGROUPS.

Please rate the person or group on a thermometer that runs from 0 to 100 degrees. Rating above 50 means that you feel favorable and warm toward the person or group. Rating below 50 means that you feel unfavorable and cool toward the person or group.

Por favor califique a la persona o grupo usando un termómetro que va de 0 a 100 grados. Una calificación por encima de 50 significa que tiene sentimientos favorables y positivos hacia esa persona o grupo. Una calificación por debajo de 50 significa que tiene sentimientos desfavorables y frío hacia la persona o grupo.

[CAWI:*Click on the line for the indicator to appear, then slide the indicator on the scale where it best reflects your answer.*

Haga clic en la línea para que aparezca el indicador, luego deslice el indicador por la escala para indicar dónde se refleja mejor su respuesta.]

Screenshot of question format below:

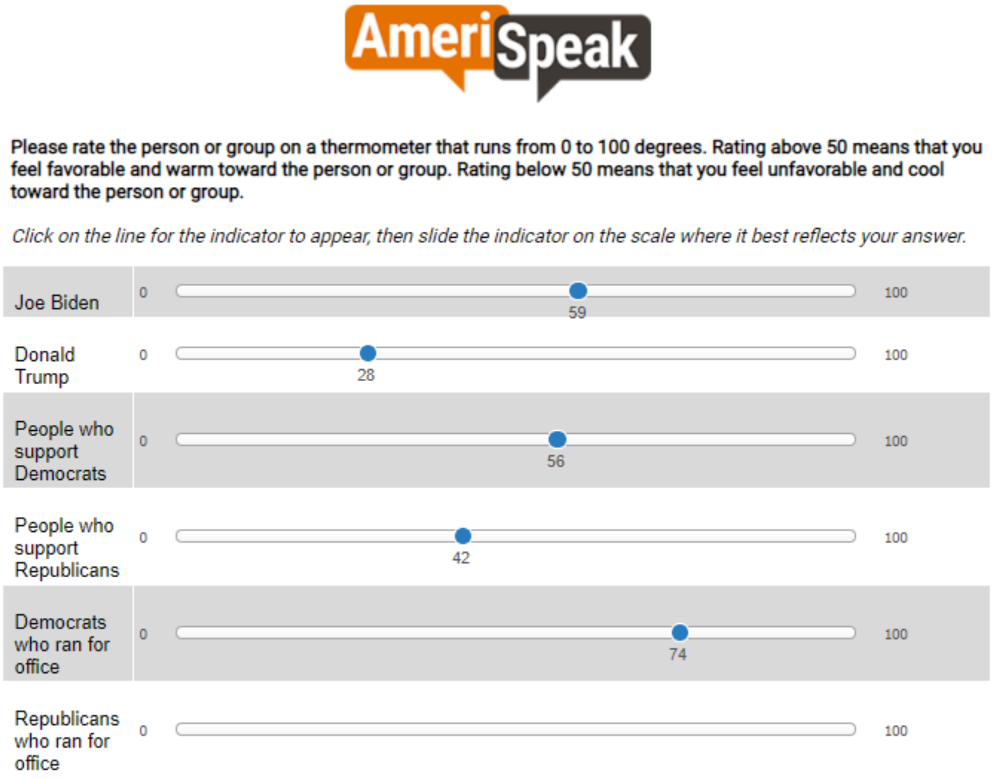
AmeriSpeak

Please rate the person or group on a thermometer that runs from 0 to 100 degrees. Rating above 50 means that you feel favorable and warm toward the person or group. Rating below 50 means that you feel unfavorable and cool toward the person or group.

Click on the line for the indicator to appear, then slide the indicator on the scale where it best reflects your answer.

Joe Biden	0	59	100
Donald Trump	0	28	100
People who support Democrats	0	56	100
People who support Republicans	0	42	100
Democrats who ran for office	0	74	100
Republicans who ran for office	0		100

SHOW IF RND_00=0:

A. Joe Biden [SLIDER SCALE]
B. Donald Trump [SLIDER SCALE]
C. People who support Democrats [SLIDER SCALE]
D. People who support Republicans [SLIDER SCALE]
E. Democrats running for office [SLIDER SCALE]
F. Republicans running for office [SLIDER SCALE]
G. Undocumented immigrants [SLIDER SCALE]
H. Rural Americans [SLIDER SCALE]
I. Black Lives Matter [SLIDER SCALE]
J. #MeToo Movement [SLIDER SCALE]
A. Joe Biden [SLIDER SCALE]
B. Donald Trump [SLIDER SCALE]
C. Las personas que apoyan a los demócratas [SLIDER SCALE]
D. Las personas que apoyan a los republicanos [SLIDER SCALE]
E. Los Demócratas que se presentan a las elecciones [SLIDER SCALE]
F. Los Republicanos que se presentan a las elecciones [SLIDER SCALE]
G. Inmigrantes indocumentados [SLIDER SCALE]
H. Los americanos rurales [SLIDER SCALE]
I. Movimiento Black Lives Matter [SLIDER SCALE]
J. Movimiento #YoTambién [SLIDER SCALE]

SHOW IF RND_00=1:

B. Donald Trump [SLIDER SCALE]
A. Joe Biden [SLIDER SCALE]
D. People who support Republicans [SLIDER SCALE]
C. People who support Democrats [SLIDER SCALE]
F. Republicans running for office [SLIDER SCALE]
E. Democrats running for office [SLIDER SCALE]
H. Rural Americans [SLIDER SCALE]
G. Undocumented immigrants [SLIDER SCALE]
I. Black Lives Matter [SLIDER SCALE]
J. #MeToo Movement [SLIDER SCALE]
B. Donald Trump [SLIDER SCALE]
A. Joe Biden [SLIDER SCALE]
D. Las personas que apoyan a los Republicanos [SLIDER SCALE]
C. Las personas que apoyan a los Demócratas [SLIDER SCALE]
F. Los republicanos que se presentan a las elecciones [SLIDER SCALE]
E. Los demócratas que se presentan a las elecciones [SLIDER SCALE]
H. Los americanos rurales [SLIDER SCALE]
G. Inmigrantes indocumentados [SLIDER SCALE]
I. Movimiento Black Lives Matter [SLIDER SCALE]
J. Movimiento #YoTambién [SLIDER SCALE]

[IF RND_00=0, SHOW DEMSMART BEFORE REPSMART. IF RND_00=1, SHOW REPSMART BEFORE DEMSMART]

DEMSMART.
In general, how smart are people who support Democrats?
En general, ¿cuán inteligentes son las personas que apoyan a los demócratas?

IF RND_01=0 1,2,3,4,5
IF RND_01=1 5,4,3,2,1
RESPONSE OPTIONS:

1. Extremely
2. Very
3. Somewhat
4. A little
5. Not at all
1. Extremadamente
2. Muy
3. Algo
4. No muy
5. Nada en absoluto

REPSMART.
In general, how smart are people who support Republicans?
En general, ¿cuán inteligentes son las personas que apoyan a los republicanos?

IF RND_01=0 1,2,3,4,5
IF RND_01=1 5,4,3,2,1
RESPONSE OPTIONS:
1. Extremely
2. Very
3. Somewhat
4. A little
5. Not at all
1. Extremadamente
2. Muy
3. Algo
4. No muy
5. Nada en absoluto

IDEOLOGY_GROUP.
How would you rate each of the following individuals and groups?
¿Cómo calificaría a cada uno de los siguientes individuos y grupos?

SHOW IF RND_00=0:
GRID ITEMS:
A. Yourself
B. Democrats running for office
C. Republicans running for office
D. People who support Democrats
E. People who support Republicans
F. [SHOW IF P_FB_USER=1 OR DOV_FB_USER=1: People you see on Facebook who support Democrats]
G. [SHOW IF P_FB_USER=1 OR DOV_FB_USER=1: People you see on Facebook who support Republicans]
H. [SHOW IF P_IG_USER=1 OR DOV_IG_USER=1: People you see on Instagram who support Democrats]
I. [SHOW IF P_IG_USER=1 OR DOV_IG_USER=1: People you see on Instagram who support Republicans]
A. Usted mismo
B. Los demócratas que se presentan a las elecciones
C. Los republicanos que se presentan a las elecciones
D. Las personas que apoyan a los demócratas
E. Las personas que apoyan a los republicanos
F. [SHOW IF P_FB_USER=1 OR DOV_FB_USER=1: La gente que se ve en Facebook que apoya a los demócratas]
G. [SHOW IF P_FB_USER=1 OR DOV_FB_USER=1: La gente que se ve en Facebook que apoya a los republicanos]

H. [SHOW IF P_IG_USER=1 OR DOV_IG_USER=1: La gente que se ve en Instagram que apoya a los demócratas]
I. [SHOW IF P_IG_USER=1 OR DOV_IG_USER=1: La gente que se ve en Instagram que apoya a los republicanos]

SHOW IF RND_00=1:
GRID ITEMS:

A. Yourself
C. Republicans running for office
B. Democrats running for office
E. People who support Republicans
D. People who support Democrats
G. [SHOW IF P_FB_USER=1 OR DOV_FB_USER=1: People you see on Facebook who support Republicans]
F. [SHOW IF P_FB_USER=1 OR DOV_FB_USER=1: People you see on Facebook who support Democrats]
I. [SHOW IF P_IG_USER=1 OR DOV_IG_USER=1: People you see on Instagram who support Republicans]
H. [SHOW IF P_IG_USER=1 OR DOV_IG_USER=1: People you see on Instagram who support Democrats]
A. Usted mismo
C. Los republicanos que se presentan a las elecciones
B. Los demócratas que se presentan a las elecciones
E. Las personas que apoyan a los republicanos
D. Las personas que apoyan a los demócratas
G. [SHOW IF P_FB_USER=1 OR DOV_FB_USER=1: La gente que se ve en Facebook que apoya a los republicanos]
F. [SHOW IF P_FB_USER=1 OR DOV_FB_USER=1: La gente que se ve en Facebook que apoya a los demócratas]
I. [SHOW IF P_IG_USER=1 OR DOV_IG_USER=1: La gente que se ve en Instagram que apoya a los republicanos]
H. [SHOW IF P_IG_USER=1 OR DOV_IG_USER=1: La gente que se ve en Instagram que apoya a los demócratas]

IF RND_01=0 1,2,3,4,5,6,7
IF RND_01=1 7,6,5,4,3,2,1
RESPONSE OPTIONS:

1. Very Liberal
2. Liberal
3. Somewhat Liberal
4. Middle of the road
5. Somewhat conservative
6. Conservative
7. Very conservative
1. Muy liberal
2. Liberal
3. Algo liberal
4. Moderado(a)

5. Algo conservador(a)
6. Conservador(a)
7. Muy conservador(a)

[SHOW IF (P_FB_USER=1 OR DOV_FB_USER=1) AND (NOT P_SAMPLE_GROUP=2, 3, OR 4)]

NETDIVFF_GROUP.
Think about your friends and family.
Piense en sus amigos y familia.

[CAWI: [SHOW IF RND_00=0: How many are Democrats, and how many are Republicans?;
SHOW IF RND_00=1: How many are Republicans, and how many are Democrats?]
[SHOW IF RND_00=0: ¿Cuántos son demócratas y cuántos republicanos?;
SHOW IF RND_00=1: ¿Cuántos son republicanos y cuántos son demócratas?]

Your best guess is fine.]
Su mejor suposición está bien.]
[CATI: IF NEEDED: Your best guess is fine.]
[CATI: IF NEEDED: Su mejor suposición está bien.]

SHOW IF RND_00=0:
GRID ITEMS:

A. How many of your friends and family are Democrats?
B. How many of your friends and family are Republicans?
A. ¿Cuántos de sus amigos y familiares son demócratas?
B. ¿Cuántos de sus amigos y familiares son republicanos?

SHOW IF RND_00=1:
GRID ITEMS:

B. How many of your friends and family are Republicans?
A. How many of your friends and family are Democrats?
B. ¿Cuántos de sus amigos y familiares son republicanos?
A. ¿Cuántos de sus amigos y familiares son demócratas?

IF RND_01=0 1,2,3,4,5
IF RND_01=1 5,4,3,2,1
RESPONSE OPTIONS:

1. None or almost none
2. A few
3. About half
4. A lot
5. All or nearly all
1. Ninguno o casi ninguno
2. Unos cuantos
3. Alrededor de la mitad
4. Muchos
5. Todos o casi todos

[SHOW IF (P_FB_USER=1 OR DOV_FB_USER=1) AND (NOT P_SAMPLE_GROUP=2, 3, OR 4)]

NETDIVFB_GROUP.
Now think about your Facebook “friends.”
Ahora piensa en sus "amigos" de Facebook.

[CAWI: Among your “friends” on Facebook, [SHOW IF RND_00=0: how many are Democrats, and how many are Republicans?; SHOW IF RND_00=1: how many are Republicans, and how many are Democrats?]
[SHOW IF RND_00=0: ¿cuántos son demócratas y cuántos republicanos?;
SHOW IF RND_00=1: ¿cuántos son republicanos y cuántos son demócratas?]

Your best guess is fine.]
Su mejor suposición está bien.]
[CATI: IF NEEDED: Your best guess is fine.]
[CATI: IF NEEDED: Su mejor suposición está bien.]

SHOW IF RND_00=0:
GRID ITEMS:
A. How many of your Facebook friends are Democrats?
B. How many of your Facebook friends are Republicans?
A. ¿Cuántos de sus amigos de Facebook son demócratas?
B. ¿Cuántos de sus amigos de Facebook son republicanos?

SHOW IF RND_00=1:
GRID ITEMS:
B. How many of your Facebook friends are Republicans?
A. How many of your Facebook friends are Democrats?
B. ¿Cuántos de sus amigos de Facebook son republicanos?
A. ¿Cuántos de sus amigos de Facebook son demócratas?

IF RND_01=0 1,2,3,4,5
IF RND_01=1 5,4,3,2,1
RESPONSE OPTIONS:
1. None or almost none
2. A few
3. About half
4. A lot
5. All or nearly all
1. Ninguno o casi ninguno
2. Unos cuantos
3. Alrededor de la mitad
4. Muchos
5. Todos o casi todos

DISPLAY_ISSUE.

Now, [IF CAWI:we, IF CATI:I] have questions about several issues facing the country.
Ahora, [IF CAWI:tenemos, IF CATI:tengo] preguntas sobre varios asuntos que enfrenta el país.

ECONOMY.
Compared to one year ago, is the nation's economy now better, the same, or worse?
Comparada con la de hace un año, ¿la economía de la nación está ahora mejor, igual o peor?

IF RND_01=0 1,2,3,4,5
IF RND_01=1 5,4,3,2,1
RESPONSE OPTIONS:
1. Much better
2. Somewhat better
3. The same
4. Somewhat worse
5. Much worse
1. Mucho mejor
2. Algo mejor
3. Igual
4. Algo peor
5. Mucho peor

BLACKWHITE_ISSUE.
In general in our country these days, would you say that [SHOW IF RND_01=0: black people are treated less fairly than white people, white people are treated less fairly than black people; SHOW IF RND_01=1: white people are treated less fairly than black people, black people are treated less fairly than white people], or both are treated about equally in each of the following situations?
¿En general, en nuestro país en estos días, ¿diría usted que [SHOW IF RND_01=0: las personas negras son tratadas menos justamente que las personas blancas, las personas blancas son tratadas menos justamente que las personas negras; SHOW IF RND_01=1:las personas blancas son tratadas menos justamente que las personas negras, las personas negras son tratadas menos justamente que las personas blancas] o ambas son tratadas más o menos por igual en cada una de las siguientes situaciones?

GRID ITEMS, RANODMIZE:
A. In dealing with the police
B. When voting in elections
C. When seeking medical treatment
D. In hiring, pay, and promotions
A. En el trato con la policía
B. Cuando se vota en las elecciones
C. Cuando se busca tratamiento medico
D. En la contratación, el pago y los ascensos

SHOW IF RND_01=0:
RESPONSE OPTIONS:
1. Black people are treated much less fairly than white people
2. Black people are treated somewhat less fairly than white people
3. Both are treated about equally

4. White people are treated somewhat less fairly than black people
5. White people are treated much less fairly than black people
1. Los negros son tratados mucho menos justamente que los blancos
2. Los negros son tratados de manera algo menos justa que los blancos
3. Ambos son tratados casi por igual
4. Los blancos son tratados de manera algo menos justa que los negros
5. Los blancos son tratados mucho menos justamente que los negros

SHOW IF RND_01=1:
RESPONSE OPTIONS:

5. White people are treated much less fairly than black people
4. White people are treated somewhat less fairly than black people
3. Both are treated about equally
2. Black people are treated somewhat less fairly than white people
1. Black people are treated much less fairly than white people
5. Los blancos son tratados mucho menos justamente que los negros
4. Los blancos son tratados de manera algo menos justa que los negros
3. Ambos son tratados casi por igual
2. Los negros son tratados de manera algo menos justa que los blancos
1. Los negros son tratados mucho menos justamente que los blancos

---

SEXISM1_2.
Do you agree or disagree with the following statements?
¿Está de acuerdo o en desacuerdo con las siguientes declaraciones?

[CATI] IF R SAYS AGREE: Is that agree strongly or agree somewhat?
[CATI] IF R SAYS DISAGREE: Is that disagree strongly or disagree somewhat?
[CATI] IF R SAYS AGREE: ¿Está completamente de acuerdo o algo de acuerdo?
[CATI] IF R SAYS DISAGREE: ¿Está fuertemente en desacuerdo o algo en desacuerdo?

GRID ITEMS, RANDOMIZE:

A. Most women interpret innocent remarks or acts as being sexist.
B. Recent allegations of sexual harassment and assault reflect widespread problems in society.
A. Muchas mujeres malinterpretan comentarios o actos inocentes como sexistas.
B. Las recientes denuncias de acoso y agresión sexual reflejan problemas generalizados en la sociedad.

IF RND_01=0 1,2,3,4,5
IF RND_01=1 5,4,3,2,1
CAWI RESPONSE OPTIONS:

1. Agree strongly
2. Agree somewhat
3. Neither agree nor disagree
4. Disagree somewhat
5. Disagree strongly

1. Fuertemente de acuerdo
2. Algo de acuerdo
3. Ni de acuerdo ni en desacuerdo
4. Algo en desacuerdo
5. Fuertemente en desacuerdo

IF RND_01=0 1,2,3,4,5
IF RND_01=1 5,4,3,2,1
CATI RESPONSE OPTIONS:
1. AGREE STRONGLY
2. AGREE SOMEWHAT
3. NEITHER AGREE NOR DISAGREE
4. DISAGREE SOMEWHAT
5. DISAGREE STRONGLY
1. FUERTEMENTE DE ACUERDO
2. ALGO DE ACUERDO
3. NI DE ACUERDO NI EN DESACUERDO
4. ALGO EN DESACUERDO
5. FUERTEMENTE EN DESACUERDO

USDEMOC_TRAIT.
How well does the United States meet the following standards?
¿Qué tan bien cumple los Estados Unidos con las siguientes normas?

GRID ITEMS, RANDOMIZE:
A. Government does not interfere with journalists or news organizations
B. Government protects individuals' right to engage in unpopular speech or expression
C. Elections are free from foreign influence
D. All adult citizens have equal opportunity to vote
E. Elections are conducted without fraud
F. Voters are knowledgeable about candidates and issues
A. El gobierno no interfiere con los periodistas o las organizaciones de noticias
B. El gobierno protege el derecho de las personas a participar en discursos o expresiones impopulares
C. Las elecciones están libres de influencia extranjera
D. Todos los ciudadanos adultos tienen la misma oportunidad de votar
E. Las elecciones se llevan a cabo sin fraude
F. Los votantes son conocedores de los candidatos y de las cuestiones

IF RND_01=0 1,2,3,4
IF RND_01=1 4,3,2,1
RESPONSE OPTIONS:
1. The U.S. does not meet this standard
2. The U.S. partly meets this standard
3. The U.S. mostly meets this standard
4. The U.S. fully meets this standard
1. Los EE.UU. no cumplen con este estándar

2. Los EE.UU. cumplen en parte con este estándar
3. Los EE.UU. en su mayoría cumplen con este estándar
4. Los EE.UU. cumplen plenamente con este estándar

KNOWLEDGE_PRE.
The next set of questions helps us learn what types of information are commonly known to the public. Please answer these questions on your own without asking anyone or looking up the answers. Many people don't know the answers to these questions, but [IF CAWI:we'd; IF CATI: I'd] be grateful if you would please answer every question even if you're not sure what the right answer is.
It is important to us that you do not use outside sources like the Internet to search for the correct answer. Will you answer the following questions without help from outside sources?
El siguiente serie de preguntas nos ayuda a saber qué tipo de información es comúnmente conocida por el público. Por favor, conteste estas preguntas por su cuenta sin preguntar a nadie o buscar las respuestas. Mucha gente no conoce las respuestas a estas preguntas, pero le [IF CAWI: agradeceríamos; IF CATI: agradecería] que por favor respondiera a cada pregunta aunque no esté seguro de cuál es la respuesta correcta.
Es importante para nosotros que usted no utilice fuentes externas como Internet para buscar la respuesta correcta. ¿Responderá a las siguientes preguntas sin ayuda de fuentes externas?

CAWI RESPONSE OPTIONS:
1. Yes
2. No
1. Sí
2. No

CATI RESPONSE OPTIONS:
1. YES
2. NO
1. SÍ
2. NO

KNOW_HOUSE.
Which party has a majority of seats in the U.S. House of Representatives?
¿Qué partido tiene la mayoría de los escaños en la Cámara de Representantes?

RESPONSE OPTIONS, RANDOMIZE:
1. Democrats
2. Republicans
3. Neither [ANCHOR]
1. Demócratas
2. Republicanos
3. Ningúno [ANCHOR]

KNOW_SENATE.
Which party has a majority of seats in the U.S. Senate?
¿Qué partido tiene la mayoría de los escaños en el Senado de los Estados Unidos?

RESPONSE OPTIONS:
1. Democrats
2. Republicans
3. Neither
1. Demócratas
2. Republicanos
3. Ningúno

DIGLITERACY_TERM.
How familiar are you with the following computer- and internet-related items? [CAWI: Please indicate your understanding of the following items:]
¿Qué tan familiarizado esta usted con los siguientes artículos relacionados con la computadora e Internet? [CAWI: Por favor, indique si entiende los siguientes elementos:]

GRID ITEMS, RANDOMIZE:
A. Viral
B. PDF
C. Selfie
D. Wiki
E. Hashtag
F. Emoji
G. Privacy settings
H. Proxypod
A. Viral
B. PDF
C. Selfie
D. Wiki
E. Hashtag
F. Emoji
G. Configuración de la privacidad
H. Proxypod

IF RND_01=0 1,2,3,4,5
IF RND_01=1 5,4,3,2,1
RESPONSE OPTIONS:
1. Full understanding
2. A lot of understanding
3. Some understanding
4. Little understanding
5. No understanding
1. Entendimiento total
2. Mucho entendimiento
3. Algo de entendimiento
4. Poco entendimiento
5. No entiendo

DISPLAY_SELF.

Lastly, [CAWI: we'd][CATI: I'd] like to ask you a few questions about yourself.
Finalmente, [CAWI: nos][CATI: me] gustaría hacerle algunas preguntas sobre usted.

EMOT.
Please tell [CAWI: us][CATI: me] how much of the time during the past 4 weeks you felt…
Por favor, [CAWI: díganos][CATI: dígame] cuánto tiempo durante las últimas 4 semanas se sintió…

GRID ITEMS, RANDOMIZE:

A. Happy
B. Depressed
C. Anxious
A. Feliz
B. Deprimido
C. Ansioso

IF RND_01=0 1,2,3,4,5
IF RND_01=1 5,4,3,2,1
RESPONSE OPTIONS:

1. All the time
2. Often
3. Sometimes
4. Rarely
5. Never
1. Todo el tiempo
2. A menudo
3. A veces
4. Raramente
5. Nunca

[SHOW IF P_SAMPLE_GROUP=3,4]

DEACTIVATION.
When you agreed to participate in this study, you said you'd be willing to deactivate your [INSERT IF P_SAMPLE_GROUP=3: Facebook][INSERT IF P_SAMPLE_GROUP=4: Instagram] account for 1 to 6 weeks, at a rate of $25 per week, starting on September 22. During your assigned deactivation period, you can continue to use messenger and WhatsApp[INSERT IF P_SAMPLE_GROUP=3: and log into apps and websites with Facebook]. When the deactivation period starts, we'll automatically deactivate your account, and you'll need to avoid logging back into [INSERT IF P_SAMPLE_GROUP=3: Facebook][INSERT IF P_SAMPLE_GROUP=4: Instagram] for the rest of the period. When you reactivate your account, it will be just as you left it.
Cuando aceptó participar en este estudio, dijo que estaría dispuesto a desactivar su cuenta de[INSERT IF P_SAMPLE_GROUP=3: Facebook][INSERT IF P_SAMPLE_GROUP=4: Instagram] durante 1 a 6 semanas, a cambio de 25 dólares por semana, a partir del 22 de septiembre. Durante el período de desactivación asignado, puede seguir utilizando el mensajero y WhatsApp [INSERT IF P_SAMPLE_GROUP=3: e iniciar sesión en aplicaciones y sitios web con Facebook]. Cuando comience el período de desactivación, desactivaremos automáticamente su cuenta y deberá evitar volver a iniciar sesión en su [INSERT IF

P_SAMPLE_GROUP=3: Facebook][INSERT IF P_SAMPLE_GROUP=4: Instagram] durante el resto del período. Cuando usted reactive su cuenta, estará tal como la dejó.
You will be randomly assigned to deactivate your [INSERT IF P_SAMPLE_GROUP=3: Facebook][INSERT IF P_SAMPLE_GROUP=4: Instagram] for either:

- 1 week, until September 29, for $25

OR

- 6 weeks, until November 3, for $150

Se le asignará al azar desactivar su [INSERT IF P_SAMPLE_GROUP=3: Facebook][INSERT IF P_SAMPLE_GROUP=4: Instagram] para:

- 1 semana, hasta el 29 de septiembre por $25

O

- 6 semanas hasta el 3 de noviembre, por $150

In both cases you will be paid in mid November and you will be asked to take three surveys for additional payment between October and December. If you are still willing to deactivate for both 1 week or 6 weeks, choose "Yes, Join Study." If not, you will still be paid for this survey but will no longer be part of the study.
En ambos casos se le pagará a mediados de noviembre y se le pedirá que realice tres encuestas para recibir un pago adicional entre octubre y diciembre. Si todavía está dispuesto a desactivar tanto por 1 o 6 semanas, elija "Sí, unirse al estudio". Si no, todavía le pagaremos por esta encuesta pero ya no formará parte del estudio.

RESPONSE OPTIONS:

1. Yes, Join Study
2. No, End Study

RESPONSE OPTIONS:
1. Sí, unirse al estudio
2. No, terminar el estudio

Wave 3

**Client**	Facebook
**Projec Language t Name**	Election Research Project W3
**Project Number**	8870
**Survey length (median)**	15 minute survey
**Population**	Age 18+
**Pretest**	N/A
**Main**	N=233,530
**MODE**	CAWI/CATI-fied web
	English/Spanish
**Sample Source**	AmeriSpeak + IG/FB sourced + ABS (from W2 completes)
**Incentive**	AmeriSpeak (PANEL_TYPE=1): 5,000<br>ABS (PANEL_TYPE=22): $10<br>Facebook/Instagram (PANEL_TYPE=23): $5
**Survey description**	Election and Politics Study 2020 Wave 3
**Eligibility Rate**	100%

Custom survey specific preloads

Variable Name	Include on Preload Testing-only page?	Variable Type	Variable Label
**P_FB_USER**	Y	Numeric	0=Not P30 day FB user<br>1=P30 day FB user
**P_IG_USER**	Y	Numeric	0=Not P30 day IG user<br>1=P30 day IG user
**PANEL_TYPE**	Y	Numeric	1 AmeriSpeak<br>2 Next Generation<br>3 GenF Extended (not in use)<br>4 AmeriSpeak Teen Panel<br>20 Lucid<br>21 SSI<br>22 ABS<br>23 FB/IG<br>50 Household 13-17<br>51 Household < 13<br>52 Household Adult

This survey will use the following RND_xx variables:

Note, these are randomized in the script (NOT preloads)

RND_xx	Associated survey Qs
RND_00	VOTEDYET, VOTE_PREELECTREPORTED, VOTE_PREELECTINTENTION, FT_PEOPLEGROUPS
RND_01	VOTE_LIKELY, CHILDPOLICY, ACAPOLICY, MINWAGEPOLICY, PROTEST1, COVIDWORRY, MISINFO, COVIDAPPROVE
RND_02	RUSSIAPOLICY, ECONOMY, POLCON, CONFINST, PROTEST2, PROTEST3, POLVIOLENCE, DEMATT_FEATURES, VOTEINFO, VOTETRUST,
RND_03	EDUCPOLICY
RND_04	FBSAT, INSTSAT

LANGSWITCH.

Welcome Back to the 2020 Election Research Project
Bienvenidos al Proyecto de Investigación Electoral 2020

Thanks for your participation in the earlier survey in the beginning of September.
Gracias por su participación en la encuesta anterior a principio de septiembre.

Let's get started with an easy question.
Empecemos con una pregunta fácil.

This survey is currently available in English and Spanish. Which language would you prefer to use to share your opinions?
Esta encuesta está actualmente disponible en inglés y en español. ¿Qué idioma prefiere usar para compartir sus opiniones?

1. English/Inglés
2. Spanish/Español

[SHOW IF PANEL_TYPE=1,22,23]
DISPLAY – OPTINTRO.

[SHOW IF PANEL_TYPE=1,22,23]
We ask you to fill out this survey that will take about 15 minutes. After you complete the survey today, we will be sending you two more surveys in November and December.
Le pedimos que complete esta encuesta que le tomará unos 15 minutos. Después de que complete la encuesta hoy, les enviaremos dos encuestas más en noviembre y diciembre.

Your participation helps researchers at New York University, The University of Texas at Austin, and other academic institutions, in partnership with Facebook, to learn more about the role of social media in elections in the United States.
Su participación ayuda a los investigadores de la Universidad de Nueva York, la Universidad de Texas en Austin y otras instituciones académicas, en colaboración con Facebook, a aprender más sobre el papel de las redes sociales en las elecciones en los Estados Unidos.

Once this study is over, de-identified data will be stored and shared by Facebook for future research on elections, to validate the findings of this study, or if required by law for an inquiry by the Institutional Review Board (IRB) that reviewed this study.
Una vez que este estudio termine, los datos desidentificados serán almacenados y compartidos por Facebook para futuras investigaciones sobre las elecciones, para validar los resultados de este estudio, o si la ley lo requiere, para una auditoría de la Junta de Revisión Institucional (IRB), la cual revisó este estudio.

There are no benefits to participating in this research, nor are there risks greater than those encountered in everyday life, including risks related to the loss of confidentiality. Your participation is completely voluntary.
No hay beneficios por participar en esta investigación, ni tampoco hay riesgos mayores que los que se encuentran en la vida cotidiana, incluyendo riesgos relacionados con la pérdida de confidencialidad. Su participación es completamente voluntaria.

[[SHOW IF PANEL TYPE=1]
You may withdraw at any time by emailing support@amerispeak.org or calling toll-free (888) 326-9424.
Puede retirarse en cualquier momento enviando un correo electrónico a ayuda@amerispeak.org o llamando al número gratuito (888) 326-9424.

[SHOW IF PANEL TYPE=22]
You may withdraw at any time by visiting 2020erp.norc.org, by emailing erpSurvey@norc.org or by calling toll-free (877) 839-1505.
Puede retirarse en cualquier momento visitando 2020erp.norc.org, enviando un correo electrónico a erpSurvey@norc.org o llamando al teléfono gratuito (877) 839-1505.

[SHOW IF PANEL TYPE=23]
You may withdraw at any time by visiting 2020erp.norc.org, by emailing erpStudy@norc.org or by calling toll-free (866) 270-2602
Puede retirarse en cualquier momento visitando 2020erp.norc.org, enviando un correo electrónico a erpStudy@norc.org o llamando al teléfono gratuito (866) 270-2602

[SHOW IF PANEL_TYPE=1,22,23]
Let's get started! We ask for your help today to tell us about yourself.
¡Empecemos! Le pedimos su ayuda hoy para que nos hable de usted.

INTRO_1.
First, we have several questions about the election for President.
Primero, tenemos varias preguntas sobre la elección para presidente.

REG_PREELEC.
Are you now registered to vote, or are you not registered? [CATI: If you are not sure, you can say that too.]
¿Está usted registrado para votar o actualmente no está registrado? [CATI: Si no está seguro, también puede decir eso.]

CAWI RESPONSE OPTIONS:

3. Registered
4. Not registered

78. Not sure
3. Registrado
4. No registrado
78. No estoy seguro

CATI RESPONSE OPTIONS:
3. REGISTERED
4. NOT REGISTERED
78. NOT SURE
3. REGISTRADO
4. NO REGISTRADO
78. NO ESTOY SEGURO

[SHOW IF REG_PREELEC=1]
REG_STATE.
In what state are you registered to vote now?
¿En qué estado está registrado/a para votar ahora?

[DROPDOWN WITH 50 STATES AND DC]

VOTEDYET.
We'd like to ask you about the election for President to be held on November 3, in which [SHOW IF RND_00=0: Joe Biden is running against Donald Trump; SHOW IF RND_00=1: Donald Trump is running against Joe Biden]. Have you already voted in that election, or have you not voted?
Ahora nos gustaría preguntarle sobre la elección para presidente que se celebrará el 3 de noviembre, en la que [SHOW IF RND_00=0: Joe Biden se está postulando contra Donald Trump; SHOW IF RND_00=1: Donald Trump se está postulando contra Joe Biden]. ¿Ya ha votado en esa elección o aún no ha votado?

RESPONSE OPTIONS:
1. Have voted
2. Have not voted
1. Ha votado
2. No ha votado

[SHOW IF VOTEDYET=1]
HOWVOTED.
Which one of the following best describes how you voted?
¿Cuál de las siguientes opciones describe mejor cómo votó?

CAWI RESPONSE OPTIONS. RANDOMIZE ORDER OF 1 AND 2:
1. Definitely voted in person at a polling place before election day
2. Definitely voted before election day by mailing in my ballot or depositing my mail ballot into a drop box
3. Definitely voted in some other way

77. Not completely sure whether I voted or not
1. Definitivamente voté en persona en un lugar de votación antes del día de las elecciones
2. Definitivamente voté antes del día de las elecciones enviando mi boleta por correo o depositando mi boleta por correo en un buzón
3. Definitivamente voté de otra manera
77. No estoy completamente seguro de si voté o no

CATI RESPONSE OPTIONS:
1. Definitely voted in person at a polling place before election day
2. Definitely voted before election day by mailing in your ballot or depositing your mail ballot into a drop box
3. Definitely voted in some other way
77. Not completely sure whether you voted or not
1. Definitivamente votó en persona en un lugar de votación antes del día de las elecciones.
2. Definitivamente votó antes del día de las elecciones enviando su boleta por correo o depositando su boleta por correo en un buzón
3. Definitivamente votó de otra manera
77. No está completamente seguro de si votó o no

[SHOW IF VOTEDYET=1]
VOTE_PREELECTREPORTED.
For whom did you vote for President of the United States?
¿Por quién votó para presidente de los Estados Unidos?

SHOW IF RND_00=0:
RESPONSE OPTIONS:
6. Joe Biden (Democrat)
7. Donald Trump (Republican)
8. Jo Jorgensen (Libertarian)
9. Howie Hawkins (Green)
10. Other candidate, please specify: [TEXTBOX]
11. [CAWI I][CATI You] didn't vote in this race
78. Not sure
6. Joe Biden (demócrata)
7. Donald Trump (republicano)
8. Jo Jorgensen (libertario)
9. Howie Hawkins (verde)
10. Otro candidato, por favor especifique: [TEXTBOX]
11. [CAWI Yo no voté][CATI Usted no votó] en esta elección
78. No estoy seguro

SHOW IF RND_00=1:
RESPONSE OPTIONS:
2. Donald Trump (Republican)
1. Joe Biden (Democrat)
3. Jo Jorgensen (Libertarian)
4. Howie Hawkins (Green)

5. Other candidate, please specify: [TEXTBOX]
6. [CAWI I][CATI You] didn’t vote in this race
77. Not sure

2. Donald Trump (republicano)
1. Joe Biden (demócrata)
3. Jo Jorgensen (libertario)
4. Howie Hawkins (verde)
5. Otro candidato, por favor especifique: [TEXTBOX]
6. [CAWI Yo no voté][CATI Usted no votó] en esta elección
77. No estoy seguro

[SHOW IF VOTEDYET=2]
VOTE_PREELECTINTENTION.
Which candidate do you prefer for President of the United States?
¿Qué candidato prefiere para Presidente de los Estados Unidos?

SHOW IF RND_00=0:
RESPONSE OPTIONS:
1. Joe Biden (Democrat)
2. Donald Trump (Republican)
3. Jo Jorgensen (Libertarian)
4. Howie Hawkins (Green)
5. Other candidate, please specify: [TEXTBOX]
6. [CAWI: I][CATI: You] don't intend to vote in this race
77. Not sure
1. Joe Biden (demócrata)
2. Donald Trump (republicano)
3. Jo Jorgensen (libertario)
4. Howie Hawkins (verde)
5. Otro candidato, por favor especifique: [TEXTBOX]
6. [CAWI: Yo no tengo][CATI: Usted no tiene] la intención de votar en esta elección
77. No estoy seguro

SHOW IF RND_00=1:
RESPONSE OPTIONS:
2. Donald Trump (Republican)
1. Joe Biden (Democrat)
3. Jo Jorgensen (Libertarian)
4. Howie Hawkins (Green)
5. Other candidate, please specify: [TEXTBOX]
6. **[CAWI:** I**][CATI:** You**]** don't intend to vote in this race
77. Not sure
2. Donald Trump (republicano)
1. Joe Biden (demócrata)
3. Jo Jorgensen (libertario)
4. Howie Hawkins (verde)
5. Otro candidato, por favor especifique: [TEXTBOX]
6. [CAWI: Yo no tengo][CATI: Usted no tiene] la intención de votar en esta elección
77. No estoy seguro

[SHOW IF VOTEDYET=2]
VOTE_LIKELY.
How likely are you to vote in the general election this November?
¿Qué probabilidad hay de que vote en las elecciones generales de noviembre?

IF RND_01=0 1,2,3,4
IF RND_01=1 4,3,2,1
RESPONSE OPTIONS:

5. Definitely will vote
6. Probably will vote
7. Probably will not vote
8. Definitely will not vote
5. Definitivamente votará
6. Probablemente votará
7. Probablemente no votará
8. Definitivamente no votará

INTRO_2.
The next set of questions asks about your perceptions of various people and groups.
La siguiente serie de preguntas se refiere a sus percepciones sobre varias personas y grupos.

FT_PEOPLEGROUPS.
Please rate the person or group on a thermometer that runs from 0 to 100 degrees. Rating above 50 means that you feel favorable and warm toward the person or group. Rating below 50 means that you feel unfavorable and cool toward the person or group.
Por favor califique a la persona o grupo usando un termómetro que va de 0 a 100 grados. Una calificación por encima de 50 significa que tiene sentimientos favorables y positivos hacia esa persona o grupo. Una calificación por debajo de 50 significa que tiene sentimientos desfavorables y frío hacia la persona o grupo.

[CAWI: *Click on the line for the indicator to appear, then slide the indicator on the scale where it best reflects your answer.*
Haga clic en la línea para que aparezca el indicador, luego deslice el indicador por la escala para indicar dónde se refleja mejor su respuesta.]

SHOW IF RND_00=0:

K. Joe Biden [SLIDER SCALE]
L. Donald Trump [SLIDER SCALE]
M. People who support Democrats [SLIDER SCALE]
N. People who support Republicans [SLIDER SCALE]
O. Democrats running for office [SLIDER SCALE]
P. Republicans running for office [SLIDER SCALE]
Q. Undocumented immigrants [SLIDER SCALE]
R. Rural Americans [SLIDER SCALE]
S. Black Lives Matter [SLIDER SCALE]
T. #MeToo Movement [SLIDER SCALE]

K. Joe Biden [SLIDER SCALE]
L. Donald Trump [SLIDER SCALE]
M. Las personas que apoyan a los demócratas [SLIDER SCALE]
N. Las personas que apoyan a los republicanos [SLIDER SCALE]
O. Los Demócratas que se presentan a las elecciones [SLIDER SCALE]
P. Los Republicanos que se presentan a las elecciones [SLIDER SCALE]
Q. Inmigrantes indocumentados [SLIDER SCALE]
R. Los americanos rurales [SLIDER SCALE]
S. Movimiento Black Lives Matter [SLIDER SCALE]
T. Movimiento #YoTambién [SLIDER SCALE]

SHOW IF RND_00=1:

B. Donald Trump [SLIDER SCALE]
A. Joe Biden [SLIDER SCALE]
D. People who support Republicans [SLIDER SCALE]
C. People who support Democrats [SLIDER SCALE]
F. Republicans running for office [SLIDER SCALE]
E. Democrats running for office [SLIDER SCALE]
H. Rural Americans [SLIDER SCALE]
G. Undocumented immigrants [SLIDER SCALE]
I. Black Lives Matter [SLIDER SCALE]
J. #MeToo Movement [SLIDER SCALE]
B. Donald Trump [SLIDER SCALE]
A. Joe Biden [SLIDER SCALE]
D. Las personas que apoyan a los Republicanos [SLIDER SCALE]
C. Las personas que apoyan a los Demócratas [SLIDER SCALE]
F. Los republicanos que se presentan a las elecciones [SLIDER SCALE]
E. Los demócratas que se presentan a las elecciones [SLIDER SCALE]
H. Los americanos rurales [SLIDER SCALE]
G. Inmigrantes indocumentados [SLIDER SCALE]
I. Movimiento Black Lives Matter [SLIDER SCALE]
J. Movimiento #YoTambién [SLIDER SCALE]

INTRO_3.

Next, we have some questions about issues facing the country.

A continuación, tenemos algunas preguntas sobre los problemas que enfrenta el país.

CHILDPOLICY.

Do you [INSERT IF RND_01=0 favor or oppose][INSERT IF RND_01=1 oppose or favor] separating the children of detained immigrants, rather than keeping them with their parents in adult detention centers?

¿Usted [INSERT IF RND_01=0 está a favor o en contra [INSERT IF RND_01= en contra o a favor] a separar a los hijos de inmigrantes detenidos, en lugar de mantenerlos con sus padres en centros de detención para adultos?

CATI: IF FAVOR OR OPPOSE: Is that strongly or somewhat?
CATI: IF FAVOR OR OPPOSE: ¿Es eso muy o algo?

RND_01=0 1,2,3,4
RND_01=1 4,3,2,1
CAWI RESPONSE OPTIONS:

1. Strongly favor
2. Somewhat favor
3. Somewhat oppose
4. Strongly oppose
1. Muy a favor
2. Algo a favor
3. Algo en contra
4. Muy en contra

RND_01=0 1,2,3,4
RND_01=1 4,3,2,1
CATI RESPONSE OPTIONS:

1. STRONGLY FAVOR
2. SOMEWHAT FAVOR
3. SOMEWHAT OPPOSE
4. STRONGLY OPPOSE
1. MUY A FAVOR
2. ALGO A FAVOR
3. ALGO EN CONTRA
4. MUY EN CONTRA

ACAPOLICY.
Do you [INSERT IF RND_01=0 approve or disapprove][INSERT IF RND_01=1 disapprove or approve] of the Affordable Care Act of 2010, sometimes referred to as Obamacare?
¿Usted [INSERT IF RND_01 = 0 aprueba o desaprueba][INSERT IF RND_01 = 1 desaprueba o aprueba] la Ley de Cuidado de Salud a Bajo Precio de 2010, a veces conocida como Obamacare?

CATI: IF APPROVE OR DISAPPROVE: Is that strongly or somewhat?
CATI: IF APPROVE OR DISAPPROVE: ¿Es eso fuertemente o algo?

RND_01=0 1,2,3,4
RND_01=1 4,3,2,1
CAWI RESPONSE OPTIONS

1. Strongly approve
2. Somewhat approve
3. Somewhat disapprove
4. Strongly disapprove
1. Aprueba mucho
2. Aprueba algo
3. Desaprueba algo
4. Desaprueba mucho

RND_01=0 1,2,3,4
RND_01=1 4,3,2,1
CATI RESPONSE OPTIONS

1. STRONGLY APPROVE
2. SOMEWHAT APPROVE
3. SOMEWHAT DISAPPROVE
4. STRONGLY DISAPPROVE
1. APRUEBA MUCHO
2. APRUEBA ALGO
3. DESAPRUEBA ALGO
4. DESAPRUEBA MUCHO

RUSSIAPOLICY.
How much is Russia a threat to the United States?
¿Qué tanto es Rusia una amenaza para los Estados Unidos?

RND_02=0 1,2,3,4,5
RND_02=1 5,4,3,2,1
CAWI RESPONSE OPTIONS

1. Not at all
2. A little
3. A moderate amount
4. A lot
5. A great deal
1. Nada en lo absoluto
2. Un poco
3. Una cantidad moderada
4. Mucho
5. Una gran cantidad

EDUCPOLICY.
All things considered, what do you think K-12 schools in your area should currently be providing?
Teniendo en cuenta todas las cosas, ¿qué cree que las escuelas K-12 en su zona deberían ofrece actualmente?

RND_03=0 1,2,3
RND_03=1 2,1,3
RESPONSE OPTIONS

1. In-person instruction five days a week
2. Online instruction five days a week
3. A mix of in-person and online instruction
1. Instrucción presencial cinco días a la semana
2. Instrucción en línea cinco días a la semana
3. Una combinación de instrucción presencial y en línea

MINWAGEPOLICY.
Should the federal minimum wage be...?
¿El salario mínimo federal debería ser ...?

RND_01=0 1,2,3,4
RND_01=1 4,3,2,1
RESPONSE OPTIONS

1. Raised
2. Kept the same
3. Lowered but not eliminated
4. Eliminated altogether
1. Aumentado
2. Mantenido en su nivel actual
3. Disminuído pero no eliminado
4. Eliminado por completo

CONFINST.
How much confidence do you have in each of the following?
¿Cuánta confianza tiene en cada uno de los siguientes?

GRID ITEMS, RANDOMIZE:

A. Presidency/executive branch
B. Congress
C. Police
D. Supreme Court
E. Your local government
F. Your state government
G. Scientific community
H. Large corporations
A. Presidencia / poder ejecutivo
B. Congreso
C. Policía
D. Tribunal Supremo
E. Su gobierno local
F. Su gobierno estatal
G. Comunidad científica
H. Grandes corporaciones

RND_02=0 1,2,3,4,5
RND_02=1 5,4,3,2,1
RESPONSE OPTIONS:

1. None
2. A little
3. A moderate amount
4. A lot
5. A great deal

1. Nada
2. Poca
3. Una cantidad moderada
4. Mucho
5. Una gran cantidad

APPROVAL.
How much do you [INSERT IF RND_02=0 approve or disapprove][INSERT IF RND_02=1 disapprove or approve]of the way Donald Trump is handling his job as president?
¿Cuánto [INSERT IF RND_02=0 aprueba o desaprueba][INSERT IF RND_02=1 desaprueba o aprueba] la manera en que Donald Trump está haciendo su trabajo como presidente?

IF RND_02=0 1,2,3,4,5
IF RND_02=1 5,4,3,2,1
RESPONSE OPTIONS:

6. Strongly approve
7. Somewhat approve
8. Neither approve nor disapprove
9. Somewhat disapprove
10. Strongly disapprove

6. Aprueba totalmente
7. Aprueba de alguna manera
8. Ni aprueba ni desaprueba
9. Desaprueba de alguna manera
10. Desaprueba totalmente

ECONOMY.
Compared to one year ago, is the nation's economy now INSERT IF RND_02=0 better, the same, or worse][INSERT IF RND_02=1 worse, the same, or better]?
En comparación con la de hace un año, ¿la economía de la nación está ahora [INSERT IF RND_02=0 mejor, igual o peor][INSERT IF RND_02=1 peor, igual o mejor]?

IF RND_02=0 1,2,3,4,5
IF RND_02=1 5,4,3,2,1
RESPONSE OPTIONS:

6. Much better
7. Somewhat better
8. The same
9. Somewhat worse
10. Much worse

6. Mucho mejor
7. Algo mejor
8. Igual
9. Algo peor
10. Mucho peor

PROTEST1.
Thinking about what it means to be a good citizen, how important is it to protest if you think government actions are wrong?
Pensando en lo que significa ser un buen ciudadano, ¿qué tan importante es protestar si cree que las acciones del gobierno están mal?

RND_01=0 1,2,3,4
RND_01=1 4,3,2,1
RESPONSE OPTIONS:

1. Very important
2. Somewhat important
3. Not too important
4. Not at all important

1. Muy importante
2. Algo importante
3. No es demasiado importante
4. Nada importante

PROTEST2.
Do you [INSERT IF RND_02=0 support or oppose][INSERT IF RND_02=1 oppose or support] the people protesting police misconduct?
¿Usted [INSERT IF RND_02 = 0 está a favor o en contra][INSERT IF RND_02 = 1 se opone o apoya] de que las personas proteste por la mala conducta policial?

RND_02=0 1,2,3,4,5
RND_02=1 5,4,3,2,1
RESPONSE OPTIONS:

1. Strongly support
2. Somewhat support
3. Neither support nor oppose
4. Somewhat oppose

5. Strongly oppose
1. Muy a favor
2. Algo a favor
3. Ni apoya a favor ni en contra
4. Algo en contra
5. Muy en contra

PROTEST3.
During the past few months, would you say that most of the actions taken by people protesting police misconduct have been [INSERT IF RND_02=0 violent, or have most of these actions been peaceful][INSERT IF RND_02=1 peaceful, or have most of these actions been violent]?
Durante los últimos meses, ¿usted diría que la mayoría de las acciones tomadas por las personas que han protestado por la mala conducta policial han sido [INSERT IF RND_02 = 0 violentas, o la mayoría de estas acciones han sido pacíficas] [INSERT IF RND_02 = 1 pacíficas, o la mayoría de estas acciones han sido violentas]?

RND_02=0 1,2,3,4,5
RND_02=1 5,4,3,2,1
RESPONSE OPTIONS:
1. Most have been violent
2. More violent than peaceful
3. Some violent, some peaceful
4. More peaceful than violent
5. Most have been peaceful
1. La mayoría han sido violentas
2. Más violentas que pacíficas
3. Algunas violentas, otros pacíficas
4. Más pacíficas que violentas
5. La mayoría han sido pacíficas

POLVIOLENCE.
Suppose that a presidential candidate declares victory even though that candidate did not legitimately win the election. To what extent do you feel like violence would be justified to ensure the actual winner is president?
Supongamos que un candidato presidencial declara la victoria a pesar de que ese candidato no ganó legítimamente las elecciones. ¿Hasta qué punto cree que la violencia estaría justificada para garantizar que el verdadero ganador sea el presidente?

RND_02=0 1,2,3,4,5
RND_02=1 5,4,3,2,1
RESPONSE OPTIONS:
1. Not at all
2. A little
3. A moderate amount
4. A lot
5. A great deal

1. Nada en lo absoluto
2. Un poco
3. Una cantidad moderada
4. Mucho
5. Una gran cantidad

INTRO_4.
We now have some questions about COVID-19, the disease caused by the coronavirus.
Ahora tenemos algunas preguntas sobre COVID-19, la enfermedad causada por el coronavirus.

COVIDAPPROVE.
How would you rate the job each of the following has done responding to COVID-19?
¿Cómo calificaría el trabajo que cada uno de los siguientes ha hecho en respuesta al COVID-19?

GRID ITEMS, RANDOMIZE:

A. President Trump
B. Your state government
C. Your local government
D. Centers for Disease Control and Prevention (CDC)
E. U.S. Congress
A. Presidente Trump
B. Su gobierno estatal
C. Su gobierno local
D. Centros para el Control y la Prevención de Enfermedades (CDC)
E. Congreso de los Estados Unidos

RND_01=0 1,2,3,4
RND_01=1 4,3,2,1
RESPONSE OPTIONS

1. Excellent
2. Good
3. Fair
4. Poor
1. Excelente
2. Bueno
3. Justo
4. Deficiente

COVIDWORRY.

How worried, if at all, are you about the risk of COVID-19?

¿Qué tan preocupado/a, si es que lo está, está por el riesgo de exposición al COVID-19?

RND_01=0 1,2,3,4
RND_01=1 4,3,2,1
RESPONSE OPTIONS

1. Very worried
2. Somewhat worried
3. Not too worried
4. Not at all worried

1. Muy preocupado/a
2. Algo preocupado/a
3. No muy preocupado/a
4. Nada preocupado/a

COVIDEXP.

For each of the following, indicate whether or not it is something that happened to <u>you or someone in your household</u> because of the COVID-19 outbreak.

Para cada uno de los siguientes, indique si es algo que le sucedió a <u>usted o alguien en su hogar</u> debido al brote de COVID-19.

[CAWI - remove bold] *Select all that apply.*
[CATI] SELECT ALL THAT APPLY.
[CAWI - remove bold] *Seleccione todas las opciones que correspondan.*
[CATI] SELECCIONE TODAS LAS OPCIONES QUE CORRESPONDAN.

RESPONSE OPTONS:

1. Tested positive for COVID-19
2. Been laid off or lost a job
3. Had to take a cut in pay due to reduced hours or demand for their work
4. None of the above

1. Probó positivo de COVID-19
2. Ha sido despedido o perdió un trabajo
3. Tuvo que aceptar un recorte salarial debido a la reducción de horas o la demanda de su trabajo
4. Ninguna de las anteriores

COVIDINFO.

In the last 24 hours, did you get any news or information related to COVID-19 from the following sources?

En las últimas 24 horas, ¿recibió alguna noticia o información relacionada al COVID-19 de las siguientes fuentes?

[CAWI - remove bold] *Select all that apply.*
[CATI] SELECT ALL THAT APPLY.
[CAWI - remove bold] *Seleccione todas las opciones que correspondan.*
[CATI] SELECCIONE TODAS LAS OPCIONES QUE CORRESPONDAN.

RESPONSE OPTIONS, RANDOMIZE:

1. National news outlets
2. Public health organizations and officials
3. Local news outlets
4. Your state/local elected officials
5. President Trump
6. Facebook
7. Instagram
8. Other, please specify: [TEXTBOX, ANCHOR]
9. I did not receive any news or information related to COVID-19 [ANCHOR]

1. Medios de comunicación nacionales
2. Organizaciones y funcionarios de salud pública
3. Medios de comunicación locales
4. Sus funcionarios electos a nivel estatal / local
5. Presidente Trump
6. Facebook
7. Instagram
8. Otro, por favor especifique: [TEXTBOX, ANCHOR]
9. No recibió ninguna noticia o información relacionada al COVID-19 [ANCHOR]

INTRO_5.
Next, we have some questions about your opinions on U.S. government.
A continuación, tenemos algunas preguntas sobre sus opiniones sobre el gobierno de EE. UU.

DEMATT_FEATURES.
How important is it that the United States meets the following standards?
¿Qué tan importante es que los Estados Unidos cumpla con los siguientes estándares?

GRID ITEMS, RANDOMIZE:

A. Government does not interfere with journalists or news organizations
B. Government protects individuals' right to engage in unpopular speech or expression
C. Elections are free from foreign influence
D. All adult citizens have equal opportunity to vote
E. Elections are conducted without fraud
F. Voters are knowledgeable about candidates and issues

A. Un gobierno que no interfiere con periodistas u organizaciones de noticias
B. Un gobierno que protege el derecho de las personas a participar en discursos o expresiones impopulares
C. Las elecciones libres de influencias extranjeras
D. Todos los ciudadanos adultos tienen la misma oportunidad de votar
E. Las elecciones que se llevan a cabo sin fraude
F. Votantes que conocen los candidatos y los problemas

RND_02=0 1,2,3,4,5
RND_02=1 5,4,3,2,1
RESPONSE OPTIONS:

1. Not important at all
2. Slightly important
3. Moderately important
4. Very important
5. Extremely important
1. Nada importante
2. Ligeramente importante
3. Moderadamente importante
4. Muy importante
5. Extremadamente importante

KNOWLEDGE_PRE.
The next set of questions helps us learn what types of information are commonly known to the public. Please answer these questions on your own without asking anyone or looking up the answers. Many people don't know the answers to these questions, but [IF CAWI:we'd; IF CATI: I'd] be grateful if you would please answer every question even if you're not sure what the right answer is.
It is important to us that you do not use outside sources like the Internet to search for the correct answer. Will you answer the following questions without help from outside sources?
El siguiente serie de preguntas nos ayuda a saber qué tipo de información es comúnmente conocida por el público. Por favor, conteste estas preguntas por su cuenta sin preguntar a nadie o buscar las respuestas. Mucha gente no conoce las respuestas a estas preguntas, pero le [IF CAWI: agradeceríamos; IF CATI: agradecería] que por favor respondiera a cada pregunta aunque no esté seguro de cuál es la respuesta correcta.
Es importante para nosotros que usted no utilice fuentes externas como Internet para buscar la respuesta correcta. ¿Responderá a las siguientes preguntas sin ayuda de fuentes externas?

CAWI RESPONSE OPTIONS:

3. Yes
4. No
3. Sí
4. No

CATI RESPONSE OPTIONS:

3. YES
4. NO
3. SÍ
4. NO

MISINFO.
To the best of your knowledge, how accurate are the following statements?
Hasta donde usted sabe, qué tan precisas son las siguientes afirmaciones?

GRID ITEMS, RANDOMIZE:

3. A new loss of taste or smell is a symptom of COVID-19
4. Coronavirus can be spread by people who do not show symptoms
5. The Chinese government created the coronavirus as a bioweapon
6. The medication hydroxychloroquine is proven to cure or prevent COVID-19
7. Antibiotics are effective in preventing and treating COVID-19
3. Una nueva pérdida del gusto u olfato es un síntoma de COVID-19
4. El coronavirus puede ser transmitido por personas que no presentan síntomas
5. El gobierno chino creó el coronavirus como una arma biológica
6. Se ha comprobado que el medicamento hidroxicloroquina cura o previene el COVID-19
7. Los antibióticos son eficaces para prevenir y tratar COVID-19

RND_01=0 1,2,3,4
RND_01=1 4,3,2,1
RESPONSE OPTIONS:

1. Not at all accurate
2. Not very accurate
3. Somewhat accurate
4. Very accurate
1. Para nada precisa
2. No muy precisa
3. Algo precisa
4. Muy precisa

[SHOW IF P_FB_USER=1 OR P_IG_USER=1]
INTRO_6.
Last, we have some questions about your experiences with social media products.
Por último, tenemos algunas preguntas sobre sus experiencias con los productos de redes sociales.

[SHOW IF P_FB_USER=1]
FBSAT.
Overall, how satisfied [INSERT IF P_SAMPLE_GRP=3: were; INSERT IF P_SAMPLE_GRP<>3: are] you with your Facebook experience [INSERT IF P_SAMPLE_GRP=3: before joining the study]?
En general, ¿qué tan satisfecho/a estaba con su experiencia en Facebook antes de unirse al estudio?

RND_04=0 1,2,3,4,5,6,7
RND_04=1 7,6,5,4,3,2,1
RESPONSE OPTIONS:

1. Completely satisfied
2. Very satisfied
3. Fairly satisfied
4. Neither satisfied nor dissatisfied
5. Fairly dissatisfied
6. Very dissatisfied
7. Completely dissatisfied

1. Completamente satisfecho/a
2. Muy satisfecho/a
3. Algo satisfecho/a
4. Ni satisfecho/a ni insatisfecho/a
5. Bastante insatisfecho/a
6. Muy insatisfecho/a
7. Completamente insatisfecho/a

[SHOW IF P_IG_USER=1]
INSTSAT.
Overall, how satisfied INSERT IF P_SAMPLE_GRP=4: were; INSERT IF P_SAMPLE_GRP<>4: are] you with your Instagram experience [INSERT IF P_SAMPLE_GRP=4: before joining the study]?
En general, ¿qué tan satisfecho/a estaba con su experiencia en Instagram antes de unirse al estudio?

RND_04=0 1,2,3,4,5,6,7
RND_04=1 7,6,5,4,3,2,1
RESPONSE OPTIONS:

1. Completely satisfied
2. Very satisfied
3. Fairly satisfied
4. Neither satisfied nor dissatisfied
5. Fairly dissatisfied
6. Very dissatisfied
7. Completely dissatisfied

1. Completamente satisfecho/a
2. Muy satisfecho/a
3. Algo satisfecho/a
4. Ni satisfecho/a ni insatisfecho/a
5. Bastante insatisfecho/a
6. Muy insatisfecho/a
7. Completamente insatisfecho/a

[SHOW IF P_FB_USER=1 AND P_SAMPLE_GRP<>3]
FBPOLCON.
In the past week, have you seen any political conversations on Facebook?
En la última semana, ¿ha visto alguna conversaciónes política en Facebook?

CAWI RESPONSE OPTIONS:
1. Yes
2. No
1. Sí
2. No

CATI RESPONSE OPTIONS:
3. YES
4. NO
3. SÍ
4. NO

[SHOW IF FBPOLCON=1]
FBPOLDES.
How well does each of the following describe the political conversations you have seen on Facebook in the last week?
¿Qué tanto describen describen cada uno de los siguientes adjetivos las conversaciones políticas que ha visto en Facebook durante la última semana?

GRID ITEMS, RANDOMIZE:
A. Respectful
B. Informative
C. Overwhelming
A. Respetuosa
B. Informativa
C. Abrumadora

RND_02=0 1,2,3,4,5
RND_02=1 5,4,3,2,1
CAWI RESPONSE OPTIONS
1. Not at all
2. A little
3. A moderate amount
4. A lot
5. A great deal
1. Para nada
2. Un poco
3. Una cantidad moderada
4. Mucho
5. Una gran cantidad

[SHOW IF P_IG_USER=1 AND P_SAMPLE_GRP<>4]
INSTPOLCON.
In the past week, have you seen any political conversations on Instagram?
En la última semana, ¿ha visto alguna conversaciónes política en Instagram?

CAWI RESPONSE OPTIONS:

1. Yes
2. No
1. Sí
2. No

CATI RESPONSE OPTIONS:

1. YES
2. NO
1. SÍ
2. NO

[SHOW IF INSTPOLCON=1]
INSTAPOLDES.
How well does each of the following describe the <u>political</u> conversations you have seen on Instagram in the last week?
¿Qué tanto describen describen cada uno de los siguientes adjetivos las conversaciones <u>políticas</u> que ha visto en Instagram durante la última semana?

GRID ITEMS, RANDOMIZE:

A. Respectful
B. Informative
C. Overwhelming
A. Respetuosa
B. Informativa
C. Abrumadora

RND_02=0 1,2,3,4,5
RND_02=1 5,4,3,2,1
CAWI RESPONSE OPTIONS

1. Not at all
2. A little
3. A moderate amount
4. A lot
5. A great deal
1. Para nada
2. Un poco
3. Una cantidad moderada
4. Mucho
5. Una gran cantidad

[DISPLAY]
DEBRIEF.
Earlier in this survey we asked you about whether you thought several statements were accurate. We did this because we are trying to understand what information people think is true and false. Before you leave the survey, we wanted to share with you that that the following statements are indeed true:

- A new loss of taste or smell is a symptom of COVID-19
- COVID-19 can be spread by people who do not show symptoms

And the following statements are not correct:

- The Chinese government created COVID-19 as a bioweapon
- The medication hydroxychloroquine is proven to cure or prevent COVID-19
- Antibiotics are effective in preventing and treating COVID-19

Al principio de esta encuesta, le preguntamos si pensaba que varias afirmaciones eran precisas. Hicimos esto porque estamos tratando de comprender qué información piensa la gente que es verdadera y falsa. Antes de salir de la encuesta, queríamos compartir con usted que las siguientes afirmaciones son verdaderas:

- Una nueva pérdida del gusto u olfato es un síntoma de COVID-19
- COVID-19 puede ser transmitido por personas que no presentan síntomas

Y las siguientes declaraciones no son correctas:

- El gobierno chino creó COVID-19 como arma biológica
- Se ha comprobado que el medicamento hidroxicloroquina cura o previene el COVID-19
- Los antibióticos son eficaces para prevenir y tratar COVID-19

Wave 4

**Client**	Facebook
**Project Name**	Election Research Project W4
**Project Number**	8870
**Survey length (median)**	15 minute survey
**Population**	Age 18+
**Pretest**	N/A
**Main**	N= 184,955
**MODE**	CAWI/CATI-fied web
**Language**	English/Spanish
**Sample Source**	AmeriSpeak + IG/FB sourced + ABS (from W2 completes)
**Incentive**	AmeriSpeak (PANEL_TYPE=1): 5,000<br>ABS (PANEL_TYPE=22): $10<br>Facebook/Instagram (PANEL_TYPE=23): $20
**Survey description**	Election and Politics Study 2020 Wave 4
**Eligibility Rate**	100%

Custom survey specific preloads

Variable Name	Include on Preload Testing-only page?	Variable Type	Variable Label
P_FB_USER	Y	Numeric	0=Not P30 day FB user<br>1=P30 day FB user
P_IG_USER	Y	Numeric	0=Not P30 day IG user<br>1=P30 day IG user
PANEL_TYPE	Y	Numeric	1 AmeriSpeak<br>2 Next Generation<br>3 GenF Extended (not in use)<br>4 AmeriSpeak Teen Panel<br>20 Lucid<br>21 SSI<br>22 ABS<br>23 FB/IG<br>50 Household 13-17<br>51 Household < 13<br>52 Household Adult

This survey will use the following RND_xx variables:

Note, these are randomized in the script (NOT preloads)

RND_xx	Associated survey Qs
RND_00	VOTE_POSTELEC, FT_PEOPLEGROUPS, DEMSMART, REPSMART, IDEOLOGY_GROUP, NETDIVFF_GROUP, NETDIVFB_GROUP
RND_01	POLINFO_SOURCE, TURNOUT_POSTELEC, USDEMOC_TRAIT, SPECKNOWEVENT, MISINFO
RND_02	INFOTRUST_SOURCE, POLINT, EPE1, EPE2, EPE3, APPROVAL, DEMSMART, REPSMART, NETDIVFF_GROUP, NETDIVFB_GROUP, IMMIGPOLICY, HEALTHPOLICY, UNEMPLOYMENTPOLICY, COVIDPOLICY, FOREIGNPOLICY, POLICEPOLICY, ECONOMY, BLACKWHITE_ISSUE, SEXISM1_2, EMOT
RND_03	IDEOLOGY_GROUP
RND_04	SPECKNOWPOLICY
RND_05	
RND_06	

LANGSWITCH.

Welcome Back to the 2020 Election Research Project
Bienvenidos al Proyecto de Investigación Electoral 2020

Thanks for your participation in the earlier survey in the beginning of September.
Gracias por su participación en la encuesta anterior a principios de septiembre.

Let's get started with an easy question.
Empecemos con una pregunta fácil.

This survey is currently available in English and Spanish. Which language would you prefer to use to share your opinions?
Esta encuesta está actualmente disponible en inglés y en español. ¿Qué idioma prefiere usar para compartir sus opiniones?

1. English/Inglés
2. Spanish/Español

If LANGSWITCH=1, 77, 98, 99 continue in English
IF LANGSWITCH=2, switch to Spanish language version of the survey

---

PROGRAMMING NOTE: FOR ALL PROMPTS: We would really like your answer to this question.]
PROGRAMMING NOTE: FOR ALL PROMPTS: Realmente nos gustaría una respuesta a esta pregunta.]

PROGRAMMING NOTE: IN CAWI MODE, HIDE BACK BUTTON IN APROD
CATI MODE MUST HAVE BACK BUTTON

[SHOW IF PANEL_TYPE=1,22,23]
DISPLAY – OPTINTRO.

We ask you to fill out this survey that will take about 20 minutes. After you complete the survey today, we will be sending you one more survey in early December.
Le pedimos que complete esta encuesta que le tomará unos 20 minutos. Después de que complete la encuesta hoy, le enviaremos una encuesta más a principios de diciembre.

Your participation helps researchers at New York University, The University of Texas at Austin, and other academic institutions, in partnership with Facebook, to learn more about the role of social media in elections in the United States.
Su participación ayuda a los investigadores de la Universidad de Nueva York, la Universidad de Texas en Austin y otras instituciones académicas, en colaboración con Facebook, a aprender más sobre el papel de las redes sociales en las elecciones en los Estados Unidos.

Once this study is over, de-identified data will be stored and shared by Facebook for future research on elections, to validate the findings of this study, or if required by law for an inquiry by the Institutional Review Board (IRB) that reviewed this study.
Una vez que este estudio termine, los datos desidentificados serán almacenados y compartidos por Facebook para futuras investigaciones sobre las elecciones, para validar los resultados de este estudio, o si la ley lo requiere, para una auditoría de la Junta de Revisión Institucional (IRB), la cual revisó este estudio.

There are no benefits to participating in this research, nor are there risks greater than those encountered in everyday life, including risks related to the loss of confidentiality. Your participation is completely voluntary.
No hay beneficios por participar en esta investigación, ni tampoco hay riesgos mayores que los que se encuentran en la vida cotidiana, incluyendo riesgos relacionados con la pérdida de confidencialidad. Su participación es completamente voluntaria.

[[SHOW IF PANEL TYPE=1]
You may withdraw at any time by emailing support@amerispeak.org or calling toll-free (888) 326-9424.
Puede retirarse en cualquier momento enviando un correo electrónico a ayuda@amerispeak.org o llamando al número gratuito (888) 326-9424.

[SHOW IF PANEL TYPE=22]
You may withdraw at any time by visiting 2020erp.norc.org, by emailing erpSurvey@norc.org or by calling toll-free (877) 839-1505.
Puede retirarse en cualquier momento visitando 2020erp.norc.org, enviando un correo electrónico a erpSurvey@norc.org o llamando al teléfono gratuito (877) 839-1505.

[SHOW IF PANEL TYPE=23]
You may withdraw at any time by visiting 2020erp.norc.org, by emailing erpStudy@norc.org or by calling toll-free (866) 270-2602
Puede retirarse en cualquier momento visitando 2020erp.norc.org, enviando un correo electrónico a erpStudy@norc.org o llamando al teléfono gratuito (866) 270-2602

Let’s get started! We ask for your help today to tell us about yourself.
¡Empecemos! Le pedimos su ayuda hoy para que nos hable de usted.

DISPLAY_MED.
First we have some questions about your media use.
Primero tenemos algunas preguntas sobre su uso de los medios de comunicación.

[GRID; 5,5,4; SP]

POLINFO_SO.
How often in the past week have you gotten political information from the following sources?
¿Con qué frecuencia en la última semana ha obtenido información política de las siguientes fuentes?

GRID ITEMS, RANDOMIZE:

A. National network TV news like ABC, CBS, or NBC
B. Print newspapers
C. Online news websites
D. Local TV news
E. Facebook
F. Instagram
G. Twitter
H. FOX News
I. MSNBC
J. CNN
K. Talk radio programs like Sean Hannity or Rush Limbaugh
L. Public radio/NPR
M. Friends and family
N. YouTube
A. Noticias de televisión nacional como ABC, CBS, o NBC
B. Periódico impreso
C. Sitios web de noticias en línea
D. Noticias de la televisión local
E. Facebook
F. Instagram
G. Twitter
H. Noticias FOX
I. MSNBC
J. CNN
K. Los programas de radio como Sean Hannity o Rush Limbaugh
L. Radio público/NPR
M. Amigos y familiares
N. YouTube

IF RND_01=0   1,2,3,4
IF RND_01=1   4,3,2,1

RESPONSE OPTIONS:

1. Every day
2. Several times
3. Once
4. Never
1. Todos los días
2. Varias veces
3. Una vez
4. Nunca

[GRID; 5,4; SP]

INFOTRUST.

How much do you think political information from each of these sources can be trusted?

¿Cuánto cree usted que se puede confiar en la información política de cada una de estas fuentes?

GRID ITEMS, RANDOMIZE:

A. Local news
B. National newspapers
C. Facebook
D. Instagram
E. Twitter
F. National network TV news like ABC, CBS, or NBC
G. MSNBC
H. CNN
I. FOX News
A. Noticias locales
B. Periódicos nacionales
C. Facebook
D. Instagram
E. Twitter
F. Noticias de televisión nacional como ABC, CBS, o NBC
G. MSNBC
H. CNN
I. Noticias FOX

IF RND_02=0 1,2,3,4,5
IF RND_02=1 5,4,3,2,1
RESPONSE OPTIONS:

1. Not at all
2. A little
3. A moderate amount
4. A lot
5. A great deal
1. Nada
2. Un poco
3. Algo

4. Mucho
5. Muchísimo

DISPLAY_POL.

Next we have some questions about your interest in politics.

A continuación tenemos algunas preguntas sobre su interés en la política.

POLINT.

How often do you pay attention to what's going on in government and politics?

¿Con qué frecuencia presta atención a los asuntos del gobierno y de la política?

IF RND_02=0 1,2,3,4,5
IF RND_02=1 5,4,3,2,1
RESPONSE OPTIONS:

1. Always
2. Most of the time
3. About half the time
4. Some of the time
5. Never
1. Siempre
2. La mayoría del tiempo
3. Casi la mitad del tiempo
4. Algunas veces
5. Nunca

POLPART.

During the past month, have you done any of the following?

Durante el pasado mes , ¿ha hecho algo de lo siguiente?

Select all that apply.

Seleccione todos los que correspondan.

RESPONSE OPTIONS, RANDOMIZE:

1. Attended a protest or rally
2. Contributed money to a political candidate or organization
3. Signed an online petition
4. Tried to convince someone how to vote (online or in-person)
5. Wrote and posted political messages online
6. Talked about politics with someone you know
7. None of the above
1. Asistió a una protesta o a un mitin
2. Contribuyó dinero a un candidato u organización política
3. Firmó una petición en línea

4. Trató de convencer a alguien de cómo votar (en línea o en persona)
5. Escribió y publicó mensajes políticos en línea
6. Habló de política con alguien que conoce
7. Ninguno de los anteriores

[SHOW IF POLPART=2]

CONTRIBUT.
How much money did you contribute to political candidates or organizations in the last month? Choose the amount that is closest.
¿Cuánto dinero contribuyó a los candidatos u organizaciones políticas en el último mes? Seleccione la cantidad que más se acerque.

RESPONSE OPTIONS:
1. $0
2. $25
3. $50
4. $100
5. $150
6. $200
7. $350
8. $500
9. $1000
10. More than $1000
1. $0
2. $25
3. $50
4. $100
5. $150
6. $200
7. $350
8. $500
9. $1000
10. Más de $1000

EPE1.
Do you agree or disagree with the following statement?
¿Está de acuerdo o en desacuerdo con la siguiente declaración?

[CAWI: I][CATI: You] feel confident that [CAWI: I][CATI: you] can find the truth about political issues.
[CAWI: Me siento][CATI: Se siente] seguro de que [CAWI: puedo][CATI: puede] encontrar la verdad sobre los asuntos políticos.

[CATI] IF R SAYS AGREE: Is that agree strongly or agree somewhat?
[CATI] IF R SAYS DISAGREE: Is that disagree strongly or disagree somewhat?

[CATI] IF R SAYS AGREE: ¿Está completamente de acuerdo o algo de acuerdo?
[CATI] IF R SAYS DISAGREE: ¿Está completamente en desacuerdo o algo en desacuerdo?

IF RND_02=0 1,2,3,4,5
IF RND_02=1 5,4,3,2,1
CAWI RESPONSE OPTIONS:

1. Agree strongly
2. Agree somewhat
3. Neither agree nor disagree
4. Disagree somewhat
5. Disagree strongly
1. Completamente de acuerdo
2. Algo de acuerdo
3. Ni de acuerdo ni en desacuerdo
4. Algo en desacuerdo
5. Completamente en desacuerdo

IF RND_02=0 1,2,3,4,5
IF RND_02=1 5,4,3,2,1
CATI RESPONSE OPTIONS:

1. AGREE STRONGLY
2. AGREE SOMEWHAT
3. NEITHER AGREE NOR DISAGREE
4. DISAGREE SOMEWHAT
5. DISAGREE STRONGLY
1. COMPLETAMENTE DE ACUERDO
2. ALGO DE ACUERDO
3. NI DE ACUERDO NI EN DESACUERDO
4. ALGO EN DESACUERDO
5. COMPLETAMENTE EN DESACUERDO

EPE2.
Do you agree or disagree with the following statements?
¿Está de acuerdo o en desacuerdo con las siguientes declaraciones?

If [CAWI: I][CATI: you] wanted to, [CAWI: I][CATI: you] could figure out the facts behind most political disputes.
Si [CAWI: yo][CATI: usted] quisiera, [CAWI: yo][CATI: usted] podría averiguar los hechos detrás de la mayoría de las disputas políticas.

[CATI] IF R SAYS AGREE: Is that agree strongly or agree somewhat?
[CATI] IF R SAYS DISAGREE: Is that disagree strongly or disagree somewhat?
[CATI] IF R SAYS AGREE: ¿Está completamente de acuerdo o algo de acuerdo?
[CATI] IF R SAYS DISAGREE: ¿Está completamente en desacuerdo o algo en desacuerdo?

IF RND_02=0 1,2,3,4,5
IF RND_02=1 5,4,3,2,1
CAWI RESPONSE OPTIONS:

1. Agree strongly
2. Agree somewhat
3. Neither agree nor disagree
4. Disagree somewhat
5. Disagree strongly
1. Completamente de acuerdo
2. Algo de acuerdo
3. Ni de acuerdo ni en desacuerdo
4. Algo en desacuerdo
5. Completamente en desacuerdo

CATI RESPONSE OPTIONS:

1. AGREE STRONGLY
2. AGREE SOMEWHAT
3. NEITHER AGREE NOR DISAGREE
4. DISAGREE SOMEWHAT
5. DISAGREE STRONGLY
1. COMPLETAMENTE DE ACUERDO
2. ALGO DE ACUERDO
3. NI DE ACUERDO NI EN DESACUERDO
4. ALGO EN DESACUERDO
5. COMPLETAMENTE EN DESACUERDO

EPE3.
Do you agree or disagree with the following statements?
¿Está de acuerdo o en desacuerdo con las siguientes declaraciones?

People like [CAWI: me][CATI: you] don't have any say in what the government does.
La gente como [CAWI: yo][CATI: usted] no tiene voz en lo que hace el gobierno.

IF RND_02=0 1,2,3,4,5
IF RND_02=1 5,4,3,2,1
CAWI RESPONSE OPTIONS:

1. Agree strongly
2. Agree somewhat
3. Neither agree nor disagree
4. Disagree somewhat
5. Disagree strongly
1. Completamente de acuerdo
2. Algo de acuerdo
3. Ni de acuerdo ni en desacuerdo
4. Algo en desacuerdo
5. Completamente en desacuerdo

CATI RESPONSE OPTIONS:

1. AGREE STRONGLY
2. AGREE SOMEWHAT
3. NEITHER AGREE NOR DISAGREE
4. DISAGREE SOMEWHAT
5. DISAGREE STRONGLY
1. COMPLETAMENTE DE ACUERDO
2. ALGO DE ACUERDO
3. NI DE ACUERDO NI EN DESACUERDO
4. ALGO EN DESACUERDO
5. COMPLETAMENTE EN DESACUERDO

DISPLAY_PRES.

Next, we have several questions about the election for President.
A continuación, tenemos varias preguntas sobre la elección para presidente.

TURNOUT.

In talking to people about elections, we often find that a lot of people were not able to vote because they weren't registered, they were sick, or they just didn't have time.
Al hablar con la gente sobre las elecciones, a menudo nos encontramos con que muchas personas no pudieron votar porque no estaban registradas, estaban enfermas o simplemente no tenían tiempo.

Which of the following statements best describes you:
Cuál de las siguientes declaraciones lo/a describe mejor:

RND_01=0 1,2,3,4
RND_01=1 4,3,2,1
CAWI RESPONSE OPTIONS:

1. I did not vote in the 2020 presidential election
2. I thought about voting this time, but didn't
3. I usually vote, but didn't this time
4. I am sure I voted in the 2020 presidential election
1. No voté en las elecciones presidenciales de 2020
2. Pensé en votar esta vez, pero no lo hice
3. Normalmente voto, pero esta vez no lo hice
4. Estoy seguro de que voté en las elecciones presidenciales de 2020

RND_01=0 1,2,3,4
RND_01=1 4,3,2,1
CATI RESPONSE OPTIONS:

1. You did not vote in the 2020 presidential election
2. You thought about voting this time, but didn't
3. You usually vote, but didn't this time
4. You are sure you voted in the 2020 presidential election
1. No votó en las elecciones presidenciales de 2020
2. Pensó en votar esta vez, pero no lo hizo
3. Normalmente vota, pero esta vez no lo hizo
4. Está seguro/a de que votó en las elecciones presidenciales de 2020

[SHOW IF TURNOUT=4]
HOWVOTED.
Which one of the following best describes how you voted?
¿Cuál de las siguientes declaraciones describe mejor cómo votó?

CAWI RESPONSE OPTIONS:

1. Definitely voted in person at a polling place before election day
2. Definitely voted in person at a polling place on election day
3. Definitely voted before election day by mailing in my ballot or depositing my mail ballot into a drop box
4. Definitely voted on election day by mailing in my ballot or depositing my mail ballot into a drop box
5. Definitely voted in some other way
1. Not completely sure whether I voted or not
1. Definitivamente voté en persona en un lugar de votación antes el día de la elección
2. Definitivamente voté en persona en un lugar de votación en el día de la elección
3. Definitivamente voté antes del día de la elección enviando mi boleta o depositando mi boleta en un buzón
4. Definitivamente voté en el día de la elección enviando mi boleta o depositando mi boleta en un buzón
5. Definitivamente voté de alguna otra manera
77. No estoy completamente seguro de si voté o no

CATI RESPONSE OPTIONS:

1. Definitely voted in person at a polling place before election day
2. Definitely voted in person at a polling place on election day
3. Definitely voted before election day by mailing in your ballot or depositing your mail ballot into a drop box
4. Definitely voted on election day by mailing in your ballot or depositing your ballot into a drop box
5. Definitely voted in some other way
1. Not completely sure whether you voted or not

1. Definitivamente votó en persona en un lugar de votación antes el día de la elección
2. Definitivamente votó en persona en un lugar de votación en el día de la elección
3. Definitivamente votó antes del día de la elección enviando su boleta o depositando su boleta en un buzón
4. Definitivamente votó en el día de la elección enviando su boleta o depositando su boleta en un buzón
5. Definitivamente votó de alguna otra manera
77. No está completamente seguro de si votó o no

[SHOW IF TURNOUT=4]

VOTE_POST.
For whom did you vote for President of the United States?
¿Por quién votó usted para Presidente de los Estados Unidos?

SHOW IF RND_00=0:
RESPONSE OPTIONS:
1. Joe Biden (Democrat)
2. Donald Trump (Republican)
3. Jo Jorgensen (Libertarian)
4. Howie Hawkins (Green)
5. Other candidate, please specify:
6. [CAWI I][CATI You] didn't vote in this race
1. Not sure
1. Joe Biden (demócrata)
2. Donald Trump (republicano)
3. Jo Jorgensen (libertario)
4. Howie Hawkins (verde)
5. Otro candidato, por favor especifique:
6. [CAWI Yo no voté][CATI Usted no votó] en esta elección
1. No estoy seguro

SHOW IF RND_00=1:
RESPONSE OPTIONS:
2. Donald Trump (Republican)
1. Joe Biden (Democrat)
3. Jo Jorgensen (Libertarian)
4. Howie Hawkins (Green)
5. Other candidate, please specify:
6. [CAWI I][CATI You] didn't vote in this race
77. Not sure
2. Donald Trump (republicano)
1. Joe Biden (demócrata)
3. Jo Jorgensen (libertario)
4. Howie Hawkins (verde)
5. Otro candidato, por favor especifique:
6. [CAWI Yo no voté][CATI Usted no votó] en esta elección

77. No estoy seguro

[SHOW IF TURNOUT=4 AND P_SCMPGN=1]

[INSERT IF S_STATE=GA]
Your state has 2 senate seats up for election in 2020. Please let us know who you voted for in each race.
Su estado tiene dos escaños en el Senado para las elecciones de 2020. Por favor, díganos por quién votó en la contienda por cada uno de los escaños.

[SHOW ALL]

VOTESENATE.
For whom did you vote for U.S. Senator?
¿Por quién votó usted para Senador de los EE.UU.?

RESPONSE OPTIONS, RANDOMIZE:

1. [SHOW IF P_SCANDE1 NOT BLANK] [INSERT: P_SCANDE1] [CATI: CANDIDATE NAME PRONUNCIATION INSERT P_SCPRO1]
2. [SHOW IF P_SCANDE2 NOT BLANK] [INSERT: P_SCANDE2 [CATI: CANDIDATE NAME PRONUNCIATION INSERT P_SCPRO2]
3. [SHOW IF P_SCANDE3 NOT BLANK] [INSERT: P_SCANDE3 [CATI: CANDIDATE NAME PRONUNCIATION INSERT P_SCPRO3]
4. [SHOW IF P_SCANDE4 NOT BLANK] [INSERT: P_SCANDE4 [CATI: CANDIDATE NAME PRONUNCIATION INSERT P_SCPRO4]
5. [SHOW IF P_SCANDE5 NOT BLANK] [INSERT: P_SCANDE5 [CATI: CANDIDATE NAME PRONUNCIATION INSERT P_SCPRO5]
6. [SHOW IF P_SCANDE6 NOT BLANK] [INSERT: P_SCANDE6 [CATI: CANDIDATE NAME PRONUNCIATION INSERT P_SCPRO6]
7. [SHOW IF P_SCANDE7 NOT BLANK] [INSERT: P_SCANDE7 [CATI: CANDIDATE NAME PRONUNCIATION INSERT P_SCPRO7]
8. [SHOW IF P_SCANDE8 NOT BLANK] [INSERT: P_SCANDE8 [CATI: CANDIDATE NAME PRONUNCIATION INSERT P_SCPRO8]
9. [SHOW IF P_SCANDE9 NOT BLANK] [INSERT: P_SCANDE9 [CATI: CANDIDATE NAME PRONUNCIATION INSERT P_SCPRO9]
10. [SHOW IF P_SCANDE10 NOT BLANK] [INSERT: P_SCANDE10] [CATI: CANDIDATE NAME PRONUNCIATION INSERT P_SCPRO10]
11. Other, please specify:
12. [CAWI I][CATI You] didn't vote in this race

1. [SHOW IF P_SCANDS1 NOT BLANK] [INSERT: P_SCANDS1] [CATI: CANDIDATE NAME PRONUNCIATION INSERT P_SCPRO1]
2. [SHOW IF P_SCANDS2 NOT BLANK] [INSERT: P_SCANDS2 [CATI: CANDIDATE NAME PRONUNCIATION INSERT P_SCPRO2]
3. [SHOW IF P_SCANDS3 NOT BLANK] [INSERT: P_SCANDS3 [CATI: CANDIDATE NAME PRONUNCIATION INSERT P_SCPRO3]
4. [SHOW IF P_SCANDS4 NOT BLANK] [INSERT: P_SCANDS4 [CATI: CANDIDATE NAME PRONUNCIATION INSERT P_SCPRO4]
5. [SHOW IF P_SCANDS5 NOT BLANK] [INSERT: P_SCANDS5 [CATI: CANDIDATE NAME PRONUNCIATION INSERT P_SCPRO5]
6. [SHOW IF P_SCANDS6 NOT BLANK] [INSERT: P_SCANDS6 [CATI: CANDIDATE NAME PRONUNCIATION INSERT P_SCPRO6]
7. [SHOW IF P_SCANDS7 NOT BLANK] [INSERT: P_SCANDS7 [CATI: CANDIDATE NAME PRONUNCIATION INSERT P_SCPRO7]
8. [SHOW IF P_SCANDS8 NOT BLANK] [INSERT: P_SCANDS8 [CATI: CANDIDATE NAME PRONUNCIATION INSERT P_SCPRO8]
9. [SHOW IF P_SCANDS9 NOT BLANK] [INSERT: P_SCANDS9 [CATI: CANDIDATE NAME PRONUNCIATION INSERT P_SCPRO9]
10. [SHOW IF P_SCANDS10 NOT BLANK] [INSERT: P_SCANDS10] [CATI: CANDIDATE NAME PRONUNCIATION INSERT P_SCPRO10]
11. Otro, por favor especifique:
12. [CAWI Yo no voté][CATI Usted no votó] en esta carrera

[INSERT IF S_STATE=GA]

VOTESENATE2
For whom did you vote for U.S. Senator?
¿Por quién votó usted para Senador de los EE.UU.?

1. [SHOW IF P_SCANDE12 NOT BLANK] [INSERT: P_SCANDE12] [CATI: CANDIDATE NAME PRONUNCIATION INSERT P_SCPRO12]
2. [SHOW IF P_SCANDE22 NOT BLANK] [INSERT: P_SCANDE22 [CATI: CANDIDATE NAME PRONUNCIATION INSERT P_SCPRO22]
3. [SHOW IF P_SCANDE32 NOT BLANK] [INSERT: P_SCANDE32 [CATI: CANDIDATE NAME PRONUNCIATION INSERT P_SCPRO32]
4. Other, please specify:
5. [CAWI I][CATI You] didn't vote in this race
1. [SHOW IF P_SCANDS12 NOT BLANK] [INSERT: P_SCANDS12] [CATI: CANDIDATE NAME PRONUNCIATION INSERT P_SCPRO12]
2. [SHOW IF P_SCANDS22 NOT BLANK] [INSERT: P_SCANDS22 [CATI: CANDIDATE NAME PRONUNCIATION INSERT P_SCPRO22]
3. [SHOW IF P_SCANDS32 NOT BLANK] [INSERT: P_SCANDS32 [CATI: CANDIDATE NAME PRONUNCIATION INSERT P_SCPRO32]
4. Otro, por favor especifique:
5. [CAWI Yo no voté][CATI Usted no votó] en esta carrera

[SHOW IF TURNOUT=4 AND P_GCMPGN=1]

VOTEGOV.
For whom did you vote for Governor?
¿Por quién votó usted para Gobernador?

RESPONSE OPTIONS, RANDOMIZE:
1. [SHOW IF P_GCANDE1 NOT BLANK] [INSERT: P_GCANDE1] [CATI: CANDIDATE NAME PRONUNCIATION INSERT P_GCPRO1]
2. [SHOW IF P_GCANDE2 NOT BLANK] [INSERT: P_GCANDE2] [CATI: CANDIDATE NAME PRONUNCIATION INSERT P_GCPRO2]
3. [SHOW IF P_GCANDE3 NOT BLANK] [INSERT: P_GCANDE3] [CATI: CANDIDATE NAME PRONUNCIATION INSERT P_GCPRO3]
4. [SHOW IF P_GCANDE4 NOT BLANK] [INSERT: P_GCANDE4] [CATI: CANDIDATE NAME PRONUNCIATION INSERT P_GCPRO4]
5. Other, please specify:
6. [CAWI I][CATI You] didn't vote in this race
1. [SHOW IF P_GCANDS1 NOT BLANK] [INSERT: P_GCANDS1] [CATI: CANDIDATE NAME PRONUNCIATION INSERT P_GCPRO1]
2. [SHOW IF P_GCANDS2 NOT BLANK] [INSERT: P_GCANDS2] [CATI: CANDIDATE NAME PRONUNCIATION INSERT P_GCPRO2]
3. [SHOW IF P_GCANDS3 NOT BLANK] [INSERT: P_GCANDS3] [CATI: CANDIDATE NAME PRONUNCIATION INSERT P_GCPRO3]
4. [SHOW IF P_GCANDS4 NOT BLANK] [INSERT: P_GCANDS4] [CATI: CANDIDATE NAME PRONUNCIATION INSERT P_GCPRO4]5.
5. Otro, por favor especifique:
6. [CAWI Yo no voté][CATI Usted no votó] en esta carrera

[SHOW IF TURNOUT=4]

VOTEHOUSE.
For whom did you vote for U.S. House?
¿Por quién votó usted para la Cámara de Representantes de los EE.UU.?

RESPONSE OPTIONS, RANDOMIZE:
1. A Democratic candidate
2. A Republican candidate
3. Other, please specify:
4. [CAWI I][CATI You] didn't vote in this race
1. Un candidato demócrata
2. Un candidato republican
3. Otro, por favor especifique:
4. [CAWI Yo no voté][CATI Usted no votó] en esta carrera

APPROVAL.

How much do you [INSERT IF RND_02=0 approve or disapprove][INSERT IF RND_02=1 disapprove or approve] of the way Donald Trump is handling his job as president?

¿Qué tanto [INSERT IF RND_02=0 aprueba o desaprueba][INSERT IF RND_02=1 desaprueba o aprueba] la manera en que Donald Trump está haciendo su trabajo como presidente?

IF RND_02=0 1,2,3,4,5
IF RND_02=1 5,4,3,2,1
RESPONSE OPTIONS:

1. Strongly approve
2. Somewhat approve
3. Neither approve nor disapprove
4. Somewhat disapprove
5. Strongly disapprove
1. Aprueba totalmente
2. Aprueba de alguna manera
3. Ni aprueba ni desaprueba
4. Desaprueba de alguna manera
5. Desaprueba totalmente

INTRO_2.

The next set of questions asks about your perceptions of various people and groups.

La siguiente serie de preguntas se refiere a sus percepciones sobre varias personas y grupos.

[CAWI: HORIZONTAL SCALE; SP; LABEL ENDPOINTS 0 AND 100; 6,4]
[CATI: NUMBOXES; VALIDATION BETWEEN 0 AND 100; 6,4]

FT_PEOP.

Please rate the person or group on a thermometer that runs from 0 to 100 degrees. Rating above 50 means that you feel favorable and warm toward the person or group. Rating below 50 means that you feel unfavorable and cool toward the person or group.

Por favor califique a la persona o grupo usando un termómetro que va de 0 a 100 grados. Una calificación por encima de 50 significa que tiene sentimientos favorables y positivos hacia esa persona o grupo. Una calificación por debajo de 50 significa que tiene sentimientos desfavorables y frío hacia la persona o grupo.

Click on the line for the indicator to appear, then slide the indicator on the scale where it best reflects your answer.

Haga clic en la línea para que aparezca el indicador, luego deslice el indicador por la escala para indicar dónde se refleja mejor su respuesta.

SHOW IF RND_00=0:

A. Joe Biden
B. Donald Trump
C. People who support Democrats
D. People who support Republicans
E. Democrats running for office
F. Republicans running for office
G. Undocumented immigrants
H. Rural Americans
I. Black Lives Matter
J. #MeToo Movement
A. Joe Biden
B. Donald Trump
C. Las personas que apoyan a los demócratas
D. Las personas que apoyan a los republicanos
E. Los Demócratas que se presentan a las elecciones
F. Los Republicanos que se presentan a las elecciones
G. Inmigrantes indocumentados
H. Los americanos rurales
I. Movimiento Black Lives Matter
J. Movimiento #YoTambién

SHOW IF RND_00=1:

A. Joe Biden
B. Donald Trump
D. People who support Republicans
C. People who support Democrats
F. Republicans running for office
E. Democrats running for office
H. Rural Americans
G. Undocumented immigrants
I. Black Lives Matter
J. #MeToo Movement
A. Joe Biden
B. Donald Trump
D. Las personas que apoyan a los Republicanos
C. Las personas que apoyan a los Demócratas
F. Los republicanos que se presentan a las elecciones
E. Los demócratas que se presentan a las elecciones
H. Los americanos rurales
G. Inmigrantes indocumentados
I. Movimiento Black Lives Matter
J. Movimiento #YoTambién

[IF RND_00=0, SHOW DEMSMART BEFORE REPSMART. IF RND_00=1, SHOW REPSMART BEFORE DEMSMART]

DEMSMART.
In general, how smart are people who support Democrats?
En general, ¿qué tan inteligentes son las personas que apoyan a los demócratas?

IF RND_02=0 1,2,3,4,5
IF RND_02=1 5,4,3,2,1
RESPONSE OPTIONS:

1. Extremely
2. Very
3. Somewhat
4. A little
5. Not at all
1. Extremadamente
2. Muy
3. Algo
4. No muy
5. Nada en absoluto

REPSMART.
In general, how smart are people who support Republicans?
En general, ¿qué tan inteligentes son las personas que apoyan a los republicanos?

IF RND_02=0 1,2,3,4,5
IF RND_02=1 5,4,3,2,1
RESPONSE OPTIONS:

1. Extremely
2. Very
3. Somewhat
4. A little
5. Not at all
1. Extremadamente
2. Muy
3. Algo
4. No muy
5. Nada en absoluto

[GRID; 5,4; SP]

IDEO_GR.
How would you rate each of the following individuals and groups?
¿Cómo calificaría a cada uno de los siguientes individuos y grupos?

SHOW IF RND_00=0:
GRID ITEMS:

A. Yourself
B. Democrats running for office
C. Republicans running for office
D. People who support Democrats
E. People who support Republicans
F. [SHOW IF P_FB_USER=1: People you see on Facebook who support Democrats]
G. [SHOW IF P_FB_USER=1: People you see on Facebook who support Republicans]
H. [SHOW IF P_IG_USER=1: People you see on Instagram who support Democrats]
I. [SHOW IF P_IG_USER=1: People you see on Instagram who support Republicans]
A. Usted mismo
B. Los demócratas que se presentan a las elecciones
C. Los republicanos que se presentan a las elecciones
D. Las personas que apoyan a los demócratas
E. Las personas que apoyan a los republicanos
F. [SHOW IF P_FB_USER=1: La gente que usted ve en Facebook que apoya a los demócratas]
G. [SHOW IF P_FB_USER=1: La gente que usted ve en Facebook que apoya a los republicanos]
H. [SHOW IF P_IG_USER=1: La gente que usted ve en Instagram que apoya a los demócratas]
I. [SHOW IF P_IG_USER=1: La gente que usted ve en Instagram que apoya a los republicanos]

SHOW IF RND_00=1:
GRID ITEMS:

A. Yourself
B. Democrats running for office
C. Republicans running for office
D. People who support Democrats
E. People who support Republicans
F. [SHOW IF P_FB_USER=1: People you see on Facebook who support Democrats]
G. [SHOW IF P_FB_USER=1: People you see on Facebook who support Republicans]
H. [SHOW IF P_IG_USER=1: People you see on Instagram who support Democrats]
I. [SHOW IF P_IG_USER=1: People you see on Instagram who support Republicans]

A. Usted mismo
B. Los demócratas que se presentan a las elecciones
C. Los republicanos que se presentan a las elecciones
D. Las personas que apoyan a los demócratas
E. Las personas que apoyan a los republicanos
F. [SHOW IF P_FB_USER=1: La gente que usted ve en Facebook que apoya a los demócratas]
G. [SHOW IF P_FB_USER=1: La gente que usted ve en Facebook que apoya a los republicanos]
H. [SHOW IF P_IG_USER=1: La gente que usted ve en Instagram que apoya a los demócratas]
I. [SHOW IF P_IG_USER=1: La gente que usted ve en Instagram que apoya a los republicanos]

IF RND_03=0 1,2,3,4,5,6,7
IF RND_03=1 7,6,5,4,3,2,1
RESPONSE OPTIONS:

1. Very liberal
2. Liberal
3. Somewhat liberal
4. Middle of the road
5. Somewhat conservative
6. Conservative
7. Very conservative
1. Muy liberal
2. Liberal
3. Algo liberal
4. Moderado(a)
5. Algo conservador(a)
6. Conservador(a)
7. Muy conservador(a)

[SHOW IF P_FB_USER=1 AND (NOT P_SAMPLE_GROUP=2, 3, OR 4)]

[GRID, SP]
NETDIVFF.
Think about your friends and family.
Piense en sus amigos y familia.

[CAWI: [SHOW IF RND_00=0: How many are Democrats, and how many are Republicans?;
SHOW IF RND_00=1: How many are Republicans, and how many are Democrats?]
[SHOW IF RND_00=0: ¿Cuántos son demócratas y cuántos republicanos?;
SHOW IF RND_00=1: ¿Cuántos son republicanos y cuántos son demócratas?]

Your best guess is fine.]
Su mejor suposición está bien.]

SHOW IF RND_00=0:

GRID ITEMS:

A. How many of your friends and family are Democrats?
B. How many of your friends and family are Republicans?
A. ¿Cuántos de sus amigos y familiares son demócratas?
B. ¿Cuántos de sus amigos y familiares son republicanos?

SHOW IF RND_00=1:

GRID ITEMS:

A. How many of your friends and family are Democrats?
B. How many of your friends and family are Republicans?
A. ¿Cuántos de sus amigos y familiares son demócratas?
B. ¿Cuántos de sus amigos y familiares son republicanos?

IF RND_02=0 1,2,3,4,5
IF RND_02=1 5,4,3,2,1
RESPONSE OPTIONS:

1. None or almost none
2. A few
3. About half
4. A lot
5. All or nearly all
1. Ninguno o casi ninguno
2. Unos cuantos
3. Alrededor de la mitad
4. Muchos
5. Todos o casi todos

[SHOW IF P_FB_USER=1 AND (NOT P_SAMPLE_GROUP=2, 3, OR 4)]
[GRID, SP]

NETDIVFB.
Now think about your Facebook "friends."
Ahora piensa en sus "amigos" de Facebook.

[CAWI: Among your "friends" on Facebook, [SHOW IF RND_00=0: how many are Democrats, and how many are Republicans?; SHOW IF RND_00=1: how many are Republicans, and how many are Democrats?]
[SHOW IF RND_00=0: ¿cuántos son demócratas y cuántos republicanos?;
SHOW IF RND_00=1: ¿cuántos son republicanos y cuántos son demócratas?]

Your best guess is fine.]
Su mejor suposición está bien.]
[CATI: IF NEEDED: Your best guess is fine.]
[CATI: IF NEEDED: Su mejor suposición está bien.]

SHOW IF RND_00=0:
GRID ITEMS:
A. How many of your Facebook friends are Democrats?
B. How many of your Facebook friends are Republicans?
A. ¿Cuántos de sus amigos de Facebook son demócratas?
B. ¿Cuántos de sus amigos de Facebook son republicanos?

SHOW IF RND_00=1:
GRID ITEMS:
A. How many of your Facebook friends are Democrats?
B. How many of your Facebook friends are Republicans?
A. ¿Cuántos de sus amigos de Facebook son demócratas?
B. ¿Cuántos de sus amigos de Facebook son republicanos?

IF RND_02=0 1,2,3,4,5
IF RND_02=1 5,4,3,2,1
RESPONSE OPTIONS:
1. None or almost none
2. A few
3. About half
4. A lot
5. All or nearly all
1. Ninguno o casi ninguno
2. Unos cuantos
3. Alrededor de la mitad
4. Muchos
5. Todos o casi todos

DISP_ISSUE.
Next, we have some questions about issues facing the country.
A continuación, tenemos algunas preguntas sobre los problemas que enfrenta el país.

[GRID]

POL.
How strongly do you [INSERT IF RND_02=0 support or oppose][INSERT IF RND_02=1 oppose or support] the following policies?
¿Qué tanto [INSERT IF RND_02=0 apoya o se opone][INSERT IF RND_02=1 se opone o apoya] a las siguientes políticas?

GRID ITEMS, RANDOMIZE:

IMMIG. Decrease the number of civilian refugees allowed into the United States from countries where people are trying to escape violence and war

HEALTH. Repeal the Affordable Care Act, also known as Obamacare

UNEMPLOY. Bring back the extra $600-per-week unemployment benefit to address economic problems resulting from the coronavirus outbreak

COVID. Require all Americans to wear face masks in public when they're around other people

FOREIGN. Ban apps that are owned by Chinese companies (like TikTok and WeChat) from operating in the United States

POLICE. Reduce funding for police departments and spend that money on social services instead

IMMIG. Reducir el número de refugiados civiles permitidos en los Estados Unidos de países donde la gente está tratando de escapar de la violencia y la guerra

SALUD. Derogar la Ley de Cuidado de Salud Asequible, también conocida como Obamacare

UNEMPLOY. Reintroducir los 600 dólares extra por semana del subsidio de desempleo para hacer frente problemas económicos derivados del brote de coronavirus

COVID. Requerir que todos los americanos usen máscaras faciales en público cuando estén cerca de otras personas

FOREIGN. Prohibir que las aplicaciones que son propiedad de empresas chinas (como TikTok y WeChat) operen en los Estados Unidos

POLICE. Reducir los fondos para los departamentos de policía y en su lugar gastar ese dinero en servicios sociales

RND_02=0 1,2,3,4,5
RND_02=1 5,4,3,2,1

RESPONSE OPTIONS:

1. Strongly support
2. Somewhat support
3. Neither support nor oppose
4. Somewhat oppose
5. Strongly oppose
1. Muy a favor
2. Algo a favor
3. Ni apoya a favor ni en contra
4. Algo en contra
5. Muy en contra

ECONOMY.

Compared to one year ago, is the nation's economy now [RND_02=0 better, the same, or worse][RND_02=1 worse, the same, or better]?

Comparada con la de hace un año, ¿la economía de la nación está ahora [RND_02=0 mejor, igual o peor][RND_02=1 peor, igual, o mejor]?

IF RND_02=0 1,2,3,4,5
IF RND_02=1 5,4,3,2,1
RESPONSE OPTIONS:
1. Much better
2. Somewhat better
3. The same
4. Somewhat worse
5. Much worse
1. Mucho mejor
2. Algo mejor
3. Igual
4. Algo peor
5. Mucho peor

[GRID, SP]

BLACKWHITE.
In general, in our country these days, would you say that [SHOW IF RND_02=0: black people are treated less fairly than white people, white people are treated less fairly than black people; SHOW IF RND_02=1: white people are treated less fairly than black people, black people are treated less fairly than white people], or both are treated about equally in each of the following situations?
¿En general, en nuestro país en estos días, ¿diría usted que [SHOW IF RND_02=0: las personas negras son tratadas menos justamente que las personas blancas, las personas blancas son tratadas menos justamente que las personas negras; SHOW IF RND_02=1:las personas blancas son tratadas menos justamente que las personas negras, las personas negras son tratadas menos justamente que las personas blancas] o ambas son tratadas más o menos por igual en cada una de las siguientes situaciones?

GRID ITEMS, RANODMIZE:
A. In dealing with the police
B. When voting in elections
C. When seeking medical treatment
D. In hiring, pay, and promotions
A. En el trato con la policía
B. Cuando se vota en las elecciones
C. Cuando se busca tratamiento médico
D. En la contratación, el pago y los ascensos

SHOW IF RND_02=0:
RESPONSE OPTIONS:
1. Black people are treated much less fairly than white people
2. Black people are treated somewhat less fairly than white people
3. Both are treated about equally
4. White people are treated somewhat less fairly than black people
5. White people are treated much less fairly than black people

1. Los negros son tratados mucho menos justamente que los blancos
2. Los negros son tratados de manera algo menos justa que los blancos
3. Ambos son tratados casi por igual
4. Los blancos son tratados de manera algo menos justa que los negros
5. Los blancos son tratados mucho menos justamente que los negros

SHOW IF RND_02=1:
RESPONSE OPTIONS:

5. White people are treated much less fairly than black people
4. White people are treated somewhat less fairly than black people
3. Both are treated about equally
2. Black people are treated somewhat less fairly than white people
1. Black people are treated much less fairly than white people
5. Los blancos son tratados mucho menos justamente que los negros
4. Los blancos son tratados de manera algo menos justa que los negros
3. Ambos son tratados casi por igual
2. Los negros son tratados de manera algo menos justa que los blancos
1. Los negros son tratados mucho menos justamente que los blancos

---

[GRID, SP]

SEXISM1_2.
Do you agree or disagree with the following statements?
¿Está de acuerdo o en desacuerdo con las siguientes declaraciones?

GRID ITEMS, RANDOMIZE:

A. Most women interpret innocent remarks or acts as being sexist
B. Recent allegations of sexual harassment and assault reflect widespread problems in society
A. Muchas mujeres malinterpretan comentarios o actos inocentes como sexistas
B. Las recientes denuncias de acoso y agresión sexual reflejan problemas generalizados en la sociedad

IF RND_02=0 1,2,3,4,5
IF RND_02=1 5,4,3,2,1
CAWI RESPONSE OPTIONS:

1. Agree strongly
2. Agree somewhat
3. Neither agree nor disagree
4. Disagree somewhat
5. Disagree strongly
1. Fuertemente de acuerdo
2. Algo de acuerdo
3. Ni de acuerdo ni en desacuerdo
4. Algo en desacuerdo
5. Fuertemente en desacuerdo

IF RND_02=0 1,2,3,4,5

IF RND_02=1 5,4,3,2,1
CATI RESPONSE OPTIONS:

1. AGREE STRONGLY
2. AGREE SOMEWHAT
3. NEITHER AGREE NOR DISAGREE
4. DISAGREE SOMEWHAT
5. DISAGREE STRONGLY
1. FUERTEMENTE DE ACUERDO
2. ALGO DE ACUERDO
3. NI DE ACUERDO NI EN DESACUERDO
4. ALGO EN DESACUERDO
5. FUERTEMENTE EN DESACUERDO

[GRID, SP]

USDEMOC.
How well does the United States meet the following standards?
¿Qué tan bien cumple los Estados Unidos con las siguientes normas?

GRID ITEMS, RANDOMIZE:

A. Government does not interfere with journalists or news organizations
B. Government protects individuals' right to engage in unpopular speech or expression
C. Elections are free from foreign influence
D. All adult citizens have equal opportunity to vote
E. Elections are conducted without fraud
F. Voters are knowledgeable about candidates and issues
A. El gobierno no interfiere con los periodistas o las organizaciones de noticias
B. El gobierno protege el derecho de las personas a participar en discursos o expresiones impopulares
C. Las elecciones están libres de influencia extranjera
D. Todos los ciudadanos adultos tienen la misma oportunidad de votar
E. Las elecciones se llevan a cabo sin fraude
F. Los votantes son conocedores de los candidatos y de las cuestiones

IF RND_01=0 1,2,3,4
IF RND_01=1 4,3,2,1
RESPONSE OPTIONS:

1. The U.S. does not meet this standard
2. The U.S. partly meets this standard
3. The U.S. mostly meets this standard
4. The U.S. fully meets this standard
1. Los EE.UU. no cumplen con este estándar
2. Los EE.UU. cumplen en parte con este estándar
3. Los EE.UU. en su mayoría cumplen con este estándar
4. Los EE.UU. cumplen plenamente con este estándar

KNOWLEDGE.

The next set of questions helps us learn what types of information are commonly known to the public. Please answer these questions on your own without asking anyone or looking up the answers. Many people don't know the answers to these questions, but [IF CAWI: we'd; IF CATI: I'd] be grateful if you would please answer every question even if you're not sure what the right answer is.

La siguiente serie de preguntas nos ayuda a saber qué tipo de información es comúnmente conocida por el público. Por favor, conteste estas preguntas por su cuenta sin preguntar a nadie o buscar las respuestas. Mucha gente no conoce las respuestas a estas preguntas, pero le [IF CAWI: agradeceríamos; IF CATI: agradecería] que por favor respondiera a cada pregunta aunque no esté seguro de cuál es la respuesta correcta.

It is important to us that you do not use outside sources like the Internet to search for the correct answer. Will you answer the following questions without help from outside sources?

Es importante para nosotros que usted no utilice fuentes externas como Internet para buscar la respuesta correcta. ¿Responderá a las siguientes preguntas sin ayuda de fuentes externas?

CAWI RESPONSE OPTIONS:

1. Yes
2. No

1. Sí
2. No

CATI RESPONSE OPTIONS:

1. YES
2. NO

1. SÍ
2. NO

[GRID]

SPECKNOWEV.

The following is a list of events. Please indicate how certain you are about whether each event did or did not happen in the last few weeks.

La siguiente es una lista de eventos. Por favor, indique que tan seguro está de que cada evento haya ocurrido o no haya ocurrido en las últimas semanas.

GRID ITEMS, RANDOMIZE:

A. France lifted all COVID-related restrictions
B. Donald Trump announced that he would stop holding public rallies out of concern for COVID-related risks
C. A militia's plot to kidnap Michigan governor Gretchen Whitmer was foiled by undercover agents
D. Derek Chauvin, the Minneapolis police officer who killed George Floyd, was promoted
E. Pope Francis voiced support for same-sex civil unions
F. During the final presidential debate, each candidate was given time to speak while the other candidate's microphone was muted
G. Amy Coney Barrett, Donald Trump's nominee, became the newest Supreme Court justice
A. Francia levantó todas las restricciones relacionadas con el COVID
B. Donald Trump anunció que dejaría de hacer mítines públicos por preocupación por los riesgos relacionados con COVID
C. El complot de una milicia para secuestrar a la gobernadora de Michigan Gretchen Whitmer fue frustrado por agentes encubiertos
D. Derek Chauvin, el policía de Minneapolis que mató a George Floyd, fue ascendido de puesto
E. El Papa Francisco expresó su apoyo a las uniones civiles entre personas del mismo sexo
F. Durante el debate presidencial final, cada candidato tuvo tiempo de hablar mientras el micrófono del otro candidato estaba silenciado
G. Amy Coney Barrett nominada por Donald Trump, se convirtió en la nueva jueza de la Corte Suprema

RND_01=0 1,2,3,4
RND_01=1 4,3,2,1
RESPONSE OPTIONS:

1. Definitely did happen
2. Probably did happen
3. Probably didn't happen
4. Definitely didn't happen
1. Definitivamente sucedió
2. Probablemente sucedió
3. Probablemente no sucedió
4. Definitivamente no sucedió

[GRID]
SPECKNOWPO.
CAWI: Below is a list of policies. Please indicate whether either [INSERT IF RND_04=0: Joe Biden or Donald Trump][INSERT IF RND_04=1: Donald Trump or Joe Biden] has publicly voiced their support for each of these policies, or if the policy is supported by neither candidate. If you're not sure, just give your best guess.
CATI: I am about to read a list of policies. Please tell me whether either [INSERT IF RND_04=0: Joe Biden or Donald Trump][INSERT IF RND_04=1: Donald Trump or Joe Biden] has publicly voiced their support for each of these policies, or if the policy is supported by neither candidate. If you're not sure, just give your best guess.
CAWI: A continuación encontrará una lista de políticas. Por favor, indique si [INSERT IF RND_04=0: Joe Biden o Donald Trump][INSERT IF RND_04=1: Donald Trump o Joe Biden] ha expresado públicamente su apoyo a cada una de esta políticas, o no son apoyadas por ninguno de los candidatos. Si no está seguro, sólo dé su mejor estimación.
CATI: Voy a leer una lista de políticas. Por favor, dígame si [INSERT IF RND_04=0: Joe Biden o Donald Trump][INSERT IF RND_04=1: Donald Trump o Joe Biden] ha expresado públicamente su apoyo a cada una de esta políticas, o no son apoyadas por ninguno de los candidatos. Si no está seguro, sólo dé su mejor estimación.

GRID ITEMS, RANDOMIZE:

A. Allow undocumented immigrants to get insurance through Medicaid
B. Raise the federal minimum wage to $15 per hour
C. Withdraw the United States from the World Health Organization (WHO)
D. Allow fossil fuel extraction in the Arctic National Wildlife Refuge
E. Replace the electoral college with a national popular vote
F. Eliminate taxes on corporations based in the U.S.
A. Permitir a los inmigrantes indocumentados obtener un seguro a través de Medicaid
B. Aumentar el salario mínimo federal a $15 por hora
C. Retirar a los Estados Unidos de la Organización Mundial de la Salud (OMS)
D. Permitir la extracción de combustibles fósiles en el Refugio Nacional de Vida Silvestre del Ártico
E. Sustituir el colegio electoral por un voto popular nacional
F. Eliminar los impuestos a las corporaciones con sede en los Estados Unidos.

RND_04=0 1,2,3
RND_04=1 2,1,3
RESPONSE OPTIONS:

1. Supported by Joe Biden
2. Supported by Donald Trump
3. Supported by neither candidate
1. Apoyado por Joe Biden
2. Apoyado por Donald Trump
3. Apoyado por ninguno de los dos candidatos

[GRID]
MISINFO.
Next [CAWI: you will see]CATI: I will read to you] a series of statements.] We'd like to know how accurate you think each of the statements are to the best of your knowledge.
A continuación [CAWI: verá][CATI: le leeré] una serie de declaraciones. Nos gustaría saber cuán precisas cree que son cada una de las declaraciones según su conocimiento.

GRID ITEMS, RANDOMIZE:

A. Evidence found on Hunter Biden's laptop proves Joe Biden took bribes from foreign powers
B. The current FBI director, Christopher Wray, has said that the greatest domestic terrorist threat is white supremacists
C. Amy Coney Barrett said that a woman needs a man's permission to own property
D. The U.S. government has a plan to force a COVID-19 vaccine on everyone
E. Masks and face coverings are not effective in preventing the spread of COVID-19
F. Millions of fraudulent ballots were cast in the 2020 presidential election
G. Donald Trump held a Bible upside-down in front of a church
H. In October, most rural counties were in the COVID-19 "red zone" based on their high rates of new cases
I. At the beginning of the COVID-19 pandemic, Anthony Fauci did not recommend wearing masks in public
J. Prior to the 2016 presidential election, Donald Trump arranged a payment to an adult film star
K. Joe Biden is a pedophile
A. Las pruebas encontradas en el portátil de Hunter Biden prueban que Joe Biden aceptó sobornos de potencias extranjeras
B. El director actual del FBI, Christopher Wray, ha dicho que la mayor amenaza terrorista doméstica son los supremacistas blancos
C. Amy Coney Barrett dijo que una mujer necesita el permiso de un hombre para tener una propiedad
D. El gobierno de EE.UU. tiene un plan para forzar una vacuna COVID-19 a todos
E. Las mascarillas y las coberturas faciales no son eficaces para prevenir la propagación de COVID-19
F. Se emitieron millones de votos fraudulentos en las elección presidencial de 2020
G. Donald Trump sostuvo una Biblia al revés frente a una iglesia
H. En octubre, la mayoría de los condados rurales estuvieron en la "zona roja" de COVID-19, basándose en sus altos índices de nuevos casos
I. Al principio de la pandemia de COVID-19, Anthony Fauci no recomendó usar mascarillas en público
J. Antes de las elecciones presidenciales de 2016, Donald Trump arregló un pago a una estrella de cine para adultos
K. Joe Biden es un pedófilo

RND_01=0 1,2,3,4
RND_01=1 4,3,2,1
RESPONSE OPTIONS:

1. Not at all accurate
2. Not very accurate
3. Somewhat accurate
4. Very accurate
1. Para nada preciso
2. No es muy preciso
3. Algo preciso
4. Muy preciso

DISPLAY_SELF.
Lastly, [CAWI: we'd][CATI: I'd] like to ask you a few questions about yourself.
Finalmente, [CAWI: nos][CATI: me] gustaría hacerle algunas preguntas sobre usted.

EMOT.
Please tell [CAWI: us][CATI: me] how much of the time during the past 4 weeks you felt…
Por favor, [CAWI: díganos][CATI: dígame] cuánto tiempo durante las últimas 4 semanas se sintió…

GRID ITEMS, RANDOMIZE:

A. Happy
B. Depressed
C. Anxious
A. Feliz
B. Deprimido
C. Ansioso

IF RND_02=0 1,2,3,4,5
IF RND_02=1 5,4,3,2,1
RESPONSE OPTIONS:

1. All the time
2. Often
3. Sometimes
4. Rarely
5. Never
1. Todo el tiempo
2. A menudo
3. A veces
4. Raramente
5. Nunca

---

reg.

Are you now registered to vote, or are you not registered? [CATI: If you're not sure, you can say that too.]
¿Está usted registrado para votar o actualmente no está registrado? [CATI: Si no está seguro/a, puede decir eso también.]

CAWI RESPONSE OPTIONS:

1. Registered
2. Not registered
1. Not sure
1. Registrado
2. No registrado
1. No estoy seguro

CATI RESPONSE OPTIONS:

1. REGISTERED
2. NOT REGISTERED
1. NOT SURE
1. REGISTRADO
2. NO REGISTRADO
1. NO ESTOY SEGURO

[SHOW IF reg=1]

[SHOW IF P_MAILADDRESS AND P_CITY AND S_STATE AND P_ZIP NOT MISSING]
regloc1.
Where are you registered to vote?
¿Dónde está registrado para votar?

CAWI RESPONSE OPTIONS:

1. At [P_MAILADDRESS P_CITY, S_STATE P_ZIP]
2. At another address
1. Not sure
1. En [P_MAILADDRESS P_CITY, S_STATE P_ZIP]
2. En otra dirección
1. No estoy seguro

[SHOW IF regloc1=2 OR (reg=1 AND P_MAILADDRESS OR P_CITY OR S_STATE OR P_ZIP MISSING)]

regloc2.
What is the address where you are registered to vote now?
¿Cuál es la dirección donde está registrado para votar ahora?

regloc2_add. Address [SMALL TEXT BOX]
regloc2_city. City [SMALL TEXT BOX]
regloc2_st. State [DROPDOWN WITH 50 STATES AND DC]
regloc2_zip. Zip [NUMBER BOX RANGE 01001 to 99950; SAVE LEADING ZERO]
regloc2_add. Dirección [SMALL TEXT BOX]
regloc2_city. Ciudad [SMALL TEXT BOX]
regloc2_st. Estado [DROPDOWN WITH 50 STATES AND DC]
regloc2_zip. Código postal [NUMBER BOX RANGE 01001 to 99950; SAVE LEADING ZERO]

[SHOW IF regloc1 = 77,98,99 or regloc2_state = 98]

[DROPDOWN]
regstate.
In what state are you registered to vote now?
¿En qué estado está registrado para votar ahora?

[DROPDOWN WITH 50 STATES AND DC]

THIS IS THE IG/FB ACCOUNT LINKING SECTION – SHOWN TO AMSP + ABS SAMPLE SOURCES WHO ARE FB or IG USER BASED ON PRELOADED SURVEY RESPONSES AT W2
[SHOW IF CAWI AND (PANEL_TYPE=1,22 AND (P_FB_USER=1 OR P_IG_USER=1))]

INTRO_7.
Next, we ask for your help on a related voluntary research study of how people use Facebook and Instagram to learn about current events.
A continuación, le pedimos su ayuda en un estudio de investigación voluntario sobre cómo las personas usan Facebook e Instagram para conocer temas de actualidad.

[SHOW IF CAWI AND (PANEL_TPYE=1,22 AND (P_FB_USER=1 OR P_IG_USER=1))]

CONSENT_FBIG.

[INSERT IF PANEL_TYPE=1]
<u>The Data Collected and Your Privacy If You Choose to Participate in the Study</u>
Los datos recopilados y su privacidad si decide participar en el estudio

- NORC will join your survey responses to publicly available third-party data like if you've voted or made a political contribution, if this data is available
- Facebook will combine this data with your activity on Facebook and Instagram from the 2020 calendar year, collectively called Combined Data
- This Combined Data will only be used for research purposes and will not be used to show you ads
- This Combined Data will be shared with Facebook, their academic partners and, if legally required, with the Institutional Review Board (IRB) that reviewed this study
- All access to this Combined Data will be monitored and logged by Facebook and NORC

- Once this study is over, de-identified data may be stored and shared by Facebook for future research on elections, to validate the findings of this study, or if required by law for an IRB inquiry
- NORC cruzará sus respuestas a la encuesta con datos de terceros disponibles públicamente, como por ejemplo si usted ha votado o hecho una contribución política, si estos datos están disponibles
- Facebook combinará estos datos con su actividad en Facebook e Instagram en el año 2020, colectivamente llamados Datos Combinados
- Estos datos combinados sólo se utilizarán con fines de investigación y no se utilizarán para mostrarle anuncios
- Estos Datos Combinados se compartirán con Facebook, sus socios académicos y, si se requiere legalmente, con la Junta de Revisión Institucional (IRB) que estuvo a cargo de revisó este estudio
- Todo el acceso a estos datos combinados será monitoreado y registrado por Facebook y NORC
- Una vez finalizado este estudio, Facebook puede almacenar y compartir datos anónimos para futuras investigaciones sobre elecciones, para validar los resultados de este estudio o, si así lo exige la ley, para una consulta del IRB

You can decide to stop participating in this study at any time, for any reason, and without consequences. You may withdraw from the study by emailing support@amerispeak.org or calling AmeriSpeak support at (888) 326-9424.
Puede decidir dejar de participar en este estudio en cualquier momento, por cualquier motivo y sin consecuencias. Puede retirarse del estudio enviando un correo electrónico a support@amerispeak.org o llamando a la unidad de soporte de AmeriSpeak al (888) 326-9424.

Do you agree to share this information with Facebook?
¿Acepta compartir esta información con Facebook?

[INSERT IF PANEL_TYPE=22]
The Data Collected and Your Privacy If You Choose to Participate in the Study
Los datos recopilados y su privacidad si decide participar en el estudio

- NORC will join your survey responses to publicly available third-party data like if you've voted or made a political contribution, if this data is available
- Facebook will combine this data with your activity on Facebook and Instagram from the 2020 calendar year, collectively called Combined Data
- This Combined Data will only be used for research purposes and will not be used to show you ads
- This Combined Data will be shared with Facebook, their academic partners and, if legally required, with the Institutional Review Board (IRB) that reviewed this study
- All access to this Combined Data will be monitored and logged by Facebook and NORC
- Once this study is over, de-identified data may be stored and shared by Facebook for future research on elections, to validate the findings of this study, or if required by law for an IRB inquiry

- NORC cruzaráunirá sus respuestas a la encuesta con datos de terceros disponibles públicamente, como por ejemplo ha votado o hecho una contribución política, si estos datos están disponibles
- Facebook combinará estos datos con su actividad en Facebook e Instagram en el año 2020, colectivamente llamados Datos Combinados
- Estos datos combinados sólo se utilizarán con fines de investigación y no se utilizarán para mostrarle anuncios
- Estos datos combinados se compartirán con Facebook, sus socios académicos y, si se requiere legalmente, con la Junta de Revisión Institucional (IRB) que estuvo a cargo de revisó este estudio
- Todo el acceso a estos datos combinados será monitoreado y registrado por Facebook y NORC
- Una vez finalizado este estudio, Facebook puede almacenar y compartir datos anónimos para futuras investigaciones sobre elecciones, para validar los resultados de este estudio o, si así lo exige la ley, para una consulta del IRB

You can decide to stop participating in this study at any time, for any reason, and without consequences. You may withdraw from the study by visiting 2020erp.norc.org, by emailing erpSurvey@norc.org or by calling toll-free (877) 839-1505.
Puede decidir dejar de participar en este estudio en cualquier momento, por cualquier motivo y sin consecuencias. Puede retirarse del estudio visitando 2020erp.norc.org, enviando un correo electrónico a erpSurvey@norc.org o llamando al número gratuito (877) 839-1505.

Do you agree to share this information with Facebook?
¿Acepta compartir esta información con Facebook?

CAWI REPONSE OPTIONS:
1. Yes, I agree
2. No, I do not agree
1. Sí, estoy de acuerdo
2. No, no estoy de acuerdo

CATI REPONSE OPTIONS:
1. Yes, you agree
2. No, you do not agree
1. Sí, está de acuerdo
2. No, no está de acuerdo

END.
Those are all the questions we have. The survey is now complete. Thank you!
Esas fueron todas las preguntas. La encuesta ya está completa. ¡Gracias!

We will come back to you for the next survey in early December.
Volveremos a usted para la próxima encuesta a principios de diciembre.

[IF P_SAMPLE_GRP=3,4] You may now reactivate your [INSERT IF P_SAMPLE_GRP=3: Facebook][INSERT IF P_SAMPLE_GRP=4: Instagram] account.
[IF P_SAMPLE_GRP=3,4] Ahora puede reactivar su cuenta de [INSERT IF P_SAMPLE_GRP=3: Facebook][INSERT IF P_SAMPLE_GRP=4: Instagram].

[IF PANEL_TYPE=1] We will add [INCENTWCOMMA] AmeriPoints to your AmeriPoints balance for completing the survey today. [SHOW IF P_W3COMP=1 As a reminder, if you complete the final wave of this study in early December, you will be eligible for a bonus 15,000 AmeriPoints.] If you have any questions at all for us, you can email us at support@AmeriSpeak.org or call us toll-free at **888-326-942**4. [CATI: Let me repeat that again: email us at support@AmeriSpeak.org or call us at **888-326-9424**.] Thank you for participating in our new AmeriSpeak survey!
[IF PANEL_TYPE=1] Agregaremos [INCENTWCOMMA] AmeriPoints a su saldo de AmeriPoints por completar la encuesta hoy. [SHOW IF P_W3COMP=1 Como recordatorio, si completa la última parte del estudio a principios de diciembre, tendrá derecho a una bonificación de 15.000 AmeriPoints.] Si tiene alguna pregunta, puede enviarnos un correo electrónico a ayuda@AmeriSpeak.org o llamarnos al número gratuito **888-326-9424**. [CATI: Permítame repetirlo nuevamente: envíenos un correo electrónico a ayuda@AmeriSpeak.org o llámenos al **888-326-9424**.] ¡Gracias por participar en nuestra nueva encuesta AmeriSpeak!

[CAWI: Please click Continue below to submit your answers.]
[CAWI: Por favor haga clic en Continuar a continuación para enviar sus respuestas.]

Wave 5

**Client**	Facebook
**Project Name**	Election Research Project W5
**Project Number**	8870
**Survey length (median)**	20 minute survey
**Population**	Age 18+
**Pretest**	N/A
**Main**	N= 160,906
**MODE**	CAWI/CATI-fied web
**Language**	English/Spanish
**Sample Source**	AmeriSpeak + IG/FB sourced + ABS (from W2 completes)
**Incentive**	AmeriSpeak (PANEL_TYPE<20): 5,000<br>ABS (PANEL_TYPE=22): $10<br>Facebook/Instagram (PANEL_TYPE=23): $20
**Survey description**	Election and Politics Study 2020 Wave 5
**Eligibility Rate**	100%

This survey will use the following RND_xx variables:

Note, these are randomized in the script (NOT preloads)

RND_xx	Associated survey Qs
RND_00	FT_PEOP
RND_01	POLINFO_SO, USDEMOC, COVIDWORRY, VACCINE, PROTEST1, TRUMPCONCEDE, MISINFO
RND_02	INFOTRUST, CONFINST, POLVIOLENCE, ELECT, CONFOFFICIALS, COUNTACCURATE, MAILACCURATE, EMOT
RND_03	ELECTWIN
RND_04	FBSAT, INSTSAT, SOCMEDIAUSE
RND_05	
RND_06	

LANGSWITCH.

Welcome Back to the 2020 Election Research Project
Bienvenidos al Proyecto de Investigación Electoral 2020

Thanks for your participation in the earlier surveys in this project.
Gracias por su participación en las encuestas anteriores de este proyecto.

Let’s get started with an easy question.
Empecemos con una pregunta fácil.

This survey is currently available in English and Spanish. Which language would you prefer to use to share your opinions?
Esta encuesta está actualmente disponible en inglés y en español. ¿Qué idioma prefiere usar para compartir sus opiniones?

1. English/Inglés
2. Spanish/Español

If LANGSWITCH=1, 77, 98, 99 continue in English
IF LANGSWITCH=2, switch to Spanish language version of the survey

PROGRAMMING NOTE: FOR ALL PROMPTS: We would really like your answer to this question.]
PROGRAMMING NOTE: FOR ALL PROMPTS: Realmente nos gustaría una respuesta a esta pregunta.]

PROGRAMMING NOTE: IN CAWI MODE, HIDE BACK BUTTON IN APROD
CATI MODE MUST HAVE BACK BUTTON

[SHOW IF PANEL_TYPE=<20 1,22,23]

DISPLAY – OPTINTRO.
[CAWI: We ask you to fill out this survey that will take about 20 minutes.][CATI: This survey will take about 20 minutes.]
[CAWI: Le pedimos que complete esta encuesta que le tomará unos 20 minutos.][CATI: Esta encuesta tomará unos 20 minutos.]

Your participation helps researchers at New York University, The University of Texas at Austin, and other academic institutions, in partnership with Facebook, to learn more about the role of social media in elections in the United States.
Su participación ayuda a los investigadores de la Universidad de Nueva York, la Universidad de Texas en Austin y otras instituciones académicas, en colaboración con Facebook, a aprender más sobre el papel de las redes sociales en las elecciones en los Estados Unidos.

Once this study is over, de-identified data will be stored and shared by Facebook for future research on elections, to validate the findings of this study, or if required by law for an inquiry by the Institutional Review Board (IRB) that reviewed this study.
Una vez que este estudio termine, los datos desidentificados serán almacenados y compartidos por Facebook para futuras investigaciones sobre las elecciones, para validar los resultados de este estudio, o si la ley lo requiere, para una auditoría de la Junta de Revisión Institucional (IRB), la cual revisó este estudio.

There are no benefits to participating in this research, nor are there risks greater than those encountered in everyday life, including risks related to the loss of confidentiality. Your participation is completely voluntary.
No hay beneficios por participar en esta investigación, ni tampoco hay riesgos mayores que los que se encuentran en la vida cotidiana, incluyendo riesgos relacionados con la pérdida de confidencialidad. Su participación es completamente voluntaria.

[[SHOW IF PANEL TYPE=1]
You may withdraw at any time by emailing support@amerispeak.org or calling toll-free (888) 326-9424.
Puede retirarse en cualquier momento enviando un correo electrónico a ayuda@amerispeak.org o llamando al número gratuito (888) 326-9424.

[SHOW IF PANEL TYPE=22]
You may withdraw at any time by visiting 2020erp.norc.org, by emailing erpSurvey@norc.org or by calling toll-free (877) 839-1505.
Puede retirarse en cualquier momento visitando 2020erp.norc.org, enviando un correo electrónico a erpSurvey@norc.org o llamando al teléfono gratuito (877) 839-1505.

[SHOW IF PANEL TYPE=23]
You may withdraw at any time by visiting 2020erp.norc.org, by emailing erpStudy@norc.org or by calling toll-free (866) 270-2602
Puede retirarse en cualquier momento visitando 2020erp.norc.org, enviando un correo electrónico a erpStudy@norc.org o llamando al teléfono gratuito (866) 270-2602

Let's get started! We ask for your help today to tell us about yourself.
¡Empecemos! Le pedimos su ayuda hoy para que nos hable de usted.

DISPLAY_MED.
First we have some questions about your media use.
Primero tenemos algunas preguntas sobre su uso de los medios de comunicación.

[GRID; 5,5,4; SP]
POLINFO_SO.
How often in the past week have you gotten political information from the following sources?
¿Con qué frecuencia en la última semana ha obtenido información política de las siguientes fuentes?

GRID ITEMS, RANDOMIZE:

A. National network TV news like ABC, CBS, or NBC
B. Print newspapers
C. Online news websites
D. Local TV news
E. Facebook
F. Instagram
G. Twitter
H. FOX News
I. MSNBC
J. CNN
K. Newsmax
L. Talk radio programs like Sean Hannity or Rush Limbaugh
M. Public radio/NPR
N. Friends and family
O. YouTube
P. TikTok
A. Noticias de televisión nacional como ABC, CBS, o NBC
B. Periódico impreso
C. Sitios web de noticias en línea
D. Noticias de la televisión local
E. Facebook
F. Instagram
G. Twitter
H. Noticias FOX
I. MSNBC
J. CNN
K. Newsmax
L. Los programas de radio como Sean Hannity o Rush Limbaugh
M. Radio público/NPR
N. Amigos y familiares
O. YouTube
P. TikTok

IF RND_01=0 1,2,3,4
IF RND_01=1 4,3,2,1

RESPONSE OPTIONS:

1. Every day
2. Several times
3. Once
4. Never
1. Todos los días
2. Varias veces
3. Una vez
4. Nunca

[GRID; 5,4; SP]

INFOTRUST.

How much do you think political information from each of these sources can be trusted?

¿Cuánto cree usted que se puede confiar en la información política de cada una de estas fuentes?

GRID ITEMS, RANDOMIZE:

A. Local news
B. National newspapers
C. Facebook
D. Instagram
E. Twitter
F. National network TV news like ABC, CBS, or NBC
G. MSNBC
H. CNN
I. FOX News
A. Noticias locales
B. Periódicos nacionales
C. Facebook
D. Instagram
E. Twitter
F. Noticias de televisión nacional como ABC, CBS, o NBC
G. MSNBC
H. CNN
I. Noticias FOX

IF RND_02=0 1,2,3,4,5
IF RND_02=1 5,4,3,2,1
RESPONSE OPTIONS:

1. Not at all
2. A little
3. A moderate amount
4. A lot
5. A great deal
1. Nada
2. Un poco
3. Algo

4. Mucho
5. Muchísimo

INTRO_2.
The next set of questions asks about your perceptions of various people and groups.
La siguiente serie de preguntas se refiere a sus percepciones sobre varias personas y grupos.

[CAWI: HORIZONTAL SCALE; SP; LABEL ENDPOINTS 0 AND 100; 6,4]
[CATI: NUMBOXES; VALIDATION BETWEN 0 AND 100; 6,4]
FT_PEOP.
Please rate the person or group on a thermometer that runs from 0 to 100 degrees. Rating above 50 means that you feel favorable and warm toward the person or group. Rating below 50 means that you feel unfavorable and cool toward the person or group.
Por favor califique a la persona o grupo usando un termómetro que va de 0 a 100 grados. Una calificación por encima de 50 significa que tiene sentimientos favorables y positivos hacia esa persona o grupo. Una calificación por debajo de 50 significa que tiene sentimientos desfavorables y frío hacia la persona o grupo.

[CAWI: *Click on the line for the indicator to appear, then slide the indicator on the scale where it best reflects your answer.*
Haga clic en la línea para que aparezca el indicador, luego deslice el indicador por la escala para indicar dónde se refleja mejor su respuesta.]

SHOW IF RND_00=0:
A. Joe Biden [SLIDER SCALE]
B. Donald Trump [SLIDER SCALE]
C. People who support Democrats [SLIDER SCALE]
D. People who support Republicans [SLIDER SCALE]
E. Democrats who ran for office [SLIDER SCALE]
F. Republicans who ran for office [SLIDER SCALE]
A. Joe Biden [SLIDER SCALE]
B. Donald Trump [SLIDER SCALE]
C. Las personas que apoyan a los demócratas [SLIDER SCALE]
D. Las personas que apoyan a los republicanos [SLIDER SCALE]
E. Los demócratas que se postularon para el cargo [SLIDER SCALE]
F. Los republicanos que se postularon para el cargo [SLIDER SCALE]

SHOW IF RND_00=1:
B. Donald Trump [SLIDER SCALE]
A. Joe Biden [SLIDER SCALE]
D. People who support Republicans [SLIDER SCALE]
C. People who support Democrats [SLIDER SCALE]
F. Republicans who ran for office [SLIDER SCALE]
E. Democrats who ran for office [SLIDER SCALE]
B. Donald Trump [SLIDER SCALE]

A. Joe Biden [SLIDER SCALE]
D. Las personas que apoyan a los republicanos [SLIDER SCALE]
C. Las personas que apoyan a los demócratas [SLIDER SCALE]
F. Los republicanos que se postularon para el cargo [SLIDER SCALE]
E. Los demócratas que se postularon para el cargo [SLIDER SCALE]

INTRO_5.
Next, we have some questions about your opinions on U.S. government.
A continuación, tenemos algunas preguntas sobre sus opiniones sobre el gobierno de EE. UU.

[GRID, SP]
USDEMOC.
How well does the United States meet the following standards?
¿Qué tan bien cumple los Estados Unidos con las siguientes normas?

GRID ITEMS, RANDOMIZE:

A. Government does not interfere with journalists or news organizations
B. Government protects individuals' right to engage in unpopular speech or expression
C. Elections are free from foreign influence
D. All adult citizens have equal opportunity to vote
E. Elections are conducted without fraud
F. Voters are knowledgeable about candidates and issues
A. El gobierno no interfiere con los periodistas o las organizaciones de noticias
B. El gobierno protege el derecho de las personas a participar en discursos o expresiones impopulares
C. Las elecciones están libres de influencia extranjera
D. Todos los ciudadanos adultos tienen la misma oportunidad de votar
E. Las elecciones se llevan a cabo sin fraude
F. Los votantes son conocedores de los candidatos y de las cuestiones

IF RND_01=0 1,2,3,4
IF RND_01=1 4,3,2,1
RESPONSE OPTIONS:

1. The U.S. does not meet this standard
2. The U.S. partly meets this standard
3. The U.S. mostly meets this standard
4. The U.S. fully meets this standard
1. Los EE.UU. no cumplen con este estándar
2. Los EE.UU. cumplen en parte con este estándar
3. Los EE.UU. en su mayoría cumplen con este estándar
4. Los EE.UU. cumplen plenamente con este estándar

[GRID; SP; 4,4]
CONFINST.
How much confidence do you have in each of the following?
¿Cuánta confianza tiene en cada uno de los siguientes?

GRID ITEMS, RANDOMIZE:

A. Presidency/executive branch
B. Congress
C. Police
D. Supreme Court
E. Your local government

F. Your state government
G. Scientific community
H. Large corporations
A. Presidencia / poder ejecutivo
B. Congreso
C. Policía
D. Tribunal Supremo
E. Su gobierno local
F. Su gobierno estatal
G. Comunidad científica
H. Grandes corporaciones

RND_02=0 1,2,3,4,5
RND_02=1 5,4,3,2,1
RESPONSE OPTIONS:
1. None
2. A little
3. A moderate amount
4. A lot
5. A great deal
1. Nada
2. Poca
3. Una cantidad moderada
4. Mucho
5. Una gran cantidad

[GRID; 3,3; SP]
DEMATT_FEATURES.
How important is it that the United States meets the following standards?
¿Qué tan importante es que los Estados Unidos cumpla con los siguientes estándares?

GRID ITEMS, RANDOMIZE:
A. Government does not interfere with journalists or news organizations
B. Government protects individuals' right to engage in unpopular speech or expression
C. Elections are free from foreign influence
D. All adult citizens have equal opportunity to vote
E. Elections are conducted without fraud
F. Voters are knowledgeable about candidates and issues
A. Un gobierno que no interfiere con periodistas u organizaciones de noticias
B. Un gobierno que protege el derecho de las personas a participar en discursos o expresiones impopulares
C. Las elecciones libres de influencias extranjeras
D. Todos los ciudadanos adultos tienen la misma oportunidad de votar
E. Las elecciones que se llevan a cabo sin fraude
F. Votantes que conocen los candidatos y los problemas

RND_02=0 1,2,3,4,5
RND_02=1 5,4,3,2,1
RESPONSE OPTIONS:

1. Not important at all
2. Slightly important
3. Moderately important
4. Very important
5. Extremely important
1. Nada importante
2. Ligeramente importante
3. Moderadamente importante
4. Muy importante
5. Extremadamente importante

INTRO_4.
We now have some questions about COVID-19, the disease caused by the coronavirus.
Ahora tenemos algunas preguntas sobre COVID-19, la enfermedad causada por el coronavirus.

[SP]
COVIDWORRY.
How worried, if at all, are you about the risk of COVID-19?
¿Qué tan preocupado/a, si es que lo está, está por el riesgo de exposición al COVID-19?

RND_01=0 1,2,3,4
RND_01=1 4,3,2,1
RESPONSE OPTIONS

1. Very worried
2. Somewhat worried
3. Not too worried
4. Not at all worried
1. Muy preocupado/a
2. Algo preocupado/a
3. No muy preocupado/a
4. Nada preocupado/a

[MP]
COVIDEXP.
For each of the following, indicate whether or not it is something that happened to <u>you or someone in your household</u> because of the COVID-19 outbreak.
Para cada uno de los siguientes, indique si es algo que le sucedió a <u>usted o alguien en su hogar</u> debido al brote de COVID-19.

Select all that apply.
Seleccione todas las opciones que correspondan.

RESPONSE OPTIONS:
1. Tested positive for COVID-19
2. Been laid off or lost a job
3. Had to take a cut in pay due to reduced hours or demand for their work
4. None of the above [SP]
1. Probó positivo de COVID-19
2. Ha sido despedido o perdió un trabajo
3. Tuvo que aceptar un recorte salarial debido a la reducción de horas o la demanda de su trabajo
4. Ninguna de las anteriores [SP]

[SP]
VACCINE.
When a COVID-19 vaccine becomes available to you, will you get vaccinated?
Cuando una vacuna COVID-19 esté disponible para usted, ¿se vacunará?

RND_01=0 1,2,3,4
RND_01=1 4,3,2,1
RESPONSE OPTIONS:
1. Definitely will get vaccinated
2. Probably will get vaccinated
3. Probably will not get vaccinated
4. Definitely will not get vaccinated
1. Definitivamente se vacunará
2. Probablemente se vacunará
3. Probablemente no se vacunará
4. Definitivamente no se vacunará

DISP_ISSUE.
Next, we have some questions about issues facing the country.
A continuación, tenemos algunas preguntas sobre los problemas que enfrenta el país.

[SP]
PROTEST1.
Thinking about what it means to be a good citizen, how important is it to protest if you think government actions are wrong?
Pensando en lo que significa ser un buen ciudadano, ¿qué tan importante es protestar si cree que las acciones del gobierno están mal?

RND_01=0 1,2,3,4
RND_01=1 4,3,2,1
RESPONSE OPTIONS:

1. Very important
2. Somewhat important
3. Not too important
4. Not at all important
1. Muy importante
2. Algo importante
3. No es demasiado importante
4. Nada importante

[SP]
ELECTWIN.
In your opinion, which candidate won the 2020 presidential election?
En su opinión, ¿qué candidato ganó las elecciones presidenciales de 2020?

RND_03=0 1,2,3
RND_03=1 2,1,3
RESPONSE OPTIONS:

1. Joe Biden
2. Donald Trump
3. Not yet determined
1. Joe Biden
2. Donald Trump
3. Aún no se ha determinado

[SP]
POLVIOLENCE.
Suppose that a presidential candidate declares victory even though that candidate did not legitimately win the election. To what extent do you feel like violence would be justified to ensure the actual winner is president?
Supongamos que un candidato presidencial declara la victoria a pesar de que ese candidato no ganó legítimamente las elecciones. ¿Hasta qué punto cree que la violencia estaría justificada para garantizar que el verdadero ganador sea el presidente?

RND_02=0 1,2,3,4,5
RND_02=1 5,4,3,2,1
RESPONSE OPTIONS:

1. Not at all
2. A little
3. A moderate amount
4. A lot
5. A great deal

1. Nada en lo absoluto
2. Un poco
3. Una cantidad moderada
4. Mucho
5. Una gran cantidad

[GRID; SP]
IRREG2020.
How often did the following occur in the 2020 presidential election?
¿Con qué frecuencia ocurrió lo siguiente en las elecciones presidenciales de 2020?

GRID ITEMS, RANDOMIZE:

A. Registered voters were illegally prevented from voting
B. People voted illegally
A. A los votantes registrados se les impidió ilegalmente votar
B. Personas votaron ilegalmente

RESPONSE OPTIONS:

1. Often
2. Sometimes
3. Rarely
4. Never
1. A menudo
2. A veces
3. Raramente
4. Nunca

CREATE STRING DOV_IRREGA

IF IRREG2020A=1	DOV_IRREGA=often
IF IRREG2020A=2	DOV_IRREGA=sometimes
IF IRREG2020A=3	DOV_IRREGA=rarely
IF IRREG2020A=1	DOV_IRREGA=a menudo
IF IRREG2020A=2	DOV_IRREGA=a veces
IF IRREG2020A=3	DOV_IRREGA=raramente

CREATE STRING DOV_IRREGB

IF IRREG2020B=1	DOV_IRREGB=often
IF IRREG2020B=2	DOV_IRREGB=sometimes
IF IRREG2020B=3	DOV_IRREGB=rarely
IF IRREG2020B=1	DOV_IRREGB=a menudo
IF IRREG2020B=2	DOV_IRREGB=a veces
IF IRREG2020B=3	DOV_IRREGB=raramente

[SHOW IF IRREG2020A=1,2,3]
[SP]
PREVENTEFFECT2020.
You said that registered voters [INSERT DOV_IRREGA] were illegally prevented from voting in the 2020 presidential election.
Usted dijo que a los votantes registrados se les impidieron ilegalmente votar [INSERT DOV_IRREGA] en las elecciones presidenciales de 2020.

Do you think this changed who won the presidential election?
¿Cree que esto cambió quién ganó las elecciones presidenciales?

RESPONSE OPTIONS:

1. Yes
2. No
77. Not sure
1. Sí
2. No
77. No sabe

[SHOW IF IRREG2020B=1,2,3]
[SP]
ILLEGALVOTEEFFECT2020.
You said that people [INSERT DOV_IRREGB] voted illegally in the 2020 presidential election.
Usted dijo que [INSERT DOV_IRREGB] la gente votó ilegalmente en las elecciones presidenciales de 2020.

Do you think this changed who won the presidential election?
¿Cree que esto cambió quién ganó las elecciones presidenciales?

RESPONSE OPTIONS:

1. Yes
2. No
77. Not sure
1. Sí
2. No
77. No sabe

[SP]
TRUMPCONCEDE.
Do you think Donald Trump should or should not concede the election to Joe Biden?
¿Cree que el Donald Trump debería o no conceder la elección a Joe Biden?

RND_01=0 1,2,3,4
RND_01=1 4,3,2,1
RESPONSE OPTIONS:

1. Definitely should concede
2. Probably should concede
3. Probably should not concede
4. Definitely should not concede
1. Definitivamente debería conceder
2. Probablemente debería conceder
3. Probablemente no debería conceder
4. Definitivamente no debería conceder

[SP]
CONFOFFICIALS.
How much confidence do you have in the officials who oversee elections?
¿Cuánta confianza tiene en los funcionarios que supervisan las elecciones?

IF RND_02=0 1,2,3,4,5
IF RND_02=1 5,4,3,2,1
RESPONSE OPTIONS:

1. None
2. A little
3. A moderate amount
4. A lot
5. A great deal
1. Nada en lo absoluto
2. Un poco
3. Una cantidad moderada
4. Mucha
5. Una gran cantidad

[SP]
COUNTACCURATE.
In the November 2020 general election, how accurately do you think the votes were counted?
En las elecciones generales de noviembre de 2020, ¿con qué exactitud cree que se contaron los votos?

IF RND_02=0 1,2,3,4,5
IF RND_02=1 5,4,3,2,1
RESPONSE OPTIONS:

1. Not at all accurately
2. Not very accurately
3. Moderately accurately
4. Very accurately
5. Completely accurately
1. Sin ninguna exactitud
2. Poca exactitud
3. Moderada exactitud
4. Mucha exactitud
5. Total exactitud

[SP]
MAILACCURATE.
How much do you trust that votes are counted accurately when people mail in their ballots?
¿Cuánto confía en que los votos sean contados con exactitud cuando la gente envía sus boletas electorales por correo?

IF RND_02=0 1,2,3,4,5
IF RND_02=1 5,4,3,2,1
RESPONSE OPTIONS:

1. A great deal
2. A lot
3. A moderate amount
4. A little
5. Not at all
1. Una gran cantidad
2. Mucho
3. Una cantidad moderada
4. Un poco
5. Nada en lo absoluto

[GRID]
MISINFO.
Next [CAWI: you will see][CATI: I will read to you] a series of statements about the 2020 election. We'd like to know how <u>accurate</u> you think each of the statements are to the best of your knowledge.
A continuación [CAWI: verá][CATI: le leeré] una serie de declaraciones sobre las elecciones de 2020. Nos gustaría saber cuán <u>precisas</u> cree que son cada una de las declaraciones según su conocimiento.

GRID ITEMS, RANDOMIZE:

A. Election observers were prohibited from observing the vote count in numerous states.
B. Millions of fraudulent mail and absentee ballots were cast.
C. The US Postal Service failed to deliver hundreds of thousands of ballots.
D. Voting machines were manipulated to add tens of thousands of votes for Joe Biden.
E. Tens of thousands of votes were recorded from dead people.
F. Immediately after the election, a pharmaceutical company announced that a new coronavirus vaccine is more than 90% effective.
G. Donald Trump's campaign held a press conference at a landscaping company next to an adult book store.
H. Donald Trump improved his vote share among Hispanic voters in Florida compared to 2016.
I. Church bells rang in Paris to celebrate Joe Biden's victory.
J. First lady Melania Trump put out a statement in the week after the election saying this would be her final Christmas in the White House.
A. Se prohibió a los observadores electorales observar el recuento de votos en numerosos estados.
B. Se emitieron millones de votos fraudulentos por correo y de votación ausente.
C. El Servicio Postal de los Estados Unidos fracasó en enviar cientos de miles de boletas electorales.
D. Las máquinas de votación fueron manipuladas para agregar decenas de miles de votos a Joe Biden.
E. Se registraron decenas de miles de votos de personas fallecidas.
F. Inmediatamente después de las elecciones, una compañía farmacéutica anunció que una nueva vacuna contra el coronavirus tiene una efectividad superior al 90%.
G. La campaña electoral de Donald Trump celebró una conferencia de prensa en una empresa de jardinería junto a una librería para adultos.
H. Donald Trump mejoró su porcentaje de votos entre los votantes hispanos en Florida en comparación con 2016.
I. Las iglesias en París tocaron sus campanas para celebrar la victoria electoral del Joe Biden.
J. La primera dama Melania Trump emitió un comunicado la semana después de las elecciones informando que esta sería su última Navidad en la Casa Blanca.

RND_01=0 1,2,3,4
RND_01=1 4,3,2,1
RESPONSE OPTIONS:

1. Not at all accurate
2. Not very accurate
3. Somewhat accurate
4. Very accurate
1. Para nada preciso
2. No es muy preciso
3. Algo preciso
4. Muy preciso

[SHOW IF P_FB_USER=1 OR P_IG_USER=1]
INTRO_6.
Next we have some questions about your use of social media.
A continuación tenemos algunas preguntas sobre su uso de las redes sociales.

[SHOW IF P_FB_USER=1]
[SP]
FBSAT.
Overall, how satisfied are you with your Facebook experience?
En general, ¿qué tan satisfecho/a estaba con su experiencia en Facebook antes de unirse al estudio?

RND_04=0 1,2,3,4,5,6,7
RND_04=1 7,6,5,4,3,2,1
RESPONSE OPTIONS:

1. Completely satisfied
2. Very satisfied
3. Fairly satisfied
4. Neither satisfied nor dissatisfied
5. Fairly dissatisfied
6. Very dissatisfied
7. Completely dissatisfied

1. Completamente satisfecho/a
2. Muy satisfecho/a
3. Algo satisfecho/a
4. Ni satisfecho/a ni insatisfecho/a
5. Bastante insatisfecho/a
6. Muy insatisfecho/a
7. Completamente insatisfecho/a

[SHOW IF P_IG_USER=1]
[SP]
INSTSAT.
Overall, how satisfied are you with your Instagram experience?
En general, ¿qué tan satisfecho/a estaba con su experiencia en Instagram antes de unirse al estudio?

RND_04=0 1,2,3,4,5,6,7
RND_04=1 7,6,5,4,3,2,1
RESPONSE OPTIONS:

1. Completely satisfied
2. Very satisfied
3. Fairly satisfied
4. Neither satisfied nor dissatisfied
5. Fairly dissatisfied
6. Very dissatisfied
7. Completely dissatisfied

1. Completamente satisfecho/a
2. Muy satisfecho/a
3. Algo satisfecho/a
4. Ni satisfecho/a ni insatisfecho/a
5. Bastante insatisfecho/a
6. Muy insatisfecho/a
7. Completamente insatisfecho/a

[SHOW IF P_FB_USER=1]
[SP]
UNFRIEND.
In the last 90 days, have you unfriended one or more people on Facebook? [CATI: If you're not sure you can say that too.]
En los últimos 90 días, ¿ha eliminado a un o más amigo(s) en Facebook? [CATI: Si no está seguro puede decir eso también.]

CAWI RESPONSE OPTIONS:
1. Yes
2. No
77. Not sure
1. Sí
2. No
77. No estoy seguro

CATI RESPONSE OPTIONS:
1. YES
2. NO
77. NOT SURE

[SHOW IF UNFRIEND=1]
[SP]
UNFRIEND_WHO.
Thinking about the people you unfriended on Facebook, to the best of your knowledge, were any of them on the opposite side of the political spectrum? [CATI: If you're not sure you can say that too.]
Pensando en los amigos que eliminó en Facebook, según su conocimiento, ¿alguno de ellos estaba en el lado opuesto del espectro político? [CATI: Si no está seguro puede decir eso también.]

CAWI RESPONSE OPTIONS:
1. Yes
2. No
77. Not sure
1. Sí
2. No
77. No estoy seguro

CATI RESPONSE OPTIONS:

1. YES
2. NO
77. NOT SURE

[SHOW IF UNFRIEND=1]
[MP]
UNFRIEND_WHY.
What are the reasons that you unfriended that person or persons?
¿Cuáles son las razones por las que eliminó a ese amigo o esos amigos en Facebook?

Select all that apply,
Seleccione todas las opciones que correspondan.

RESPONSE OPTIONS

1. Posted too much political content
2. Posted things that you disagreed with politically
3. Posted something you found offensive
4. Were abusive or harassing
5. Some other reason [TEXTBOX]
1. Publicó demasiado contenido político
2. Publicó cosas con las que no estaba de acuerdo políticamente
3. Publicó algo que encontró ofensivo
4. Fueron abusivos o acosadores
5. Alguna otra razón

SOCMEDIAUSE.
How often do you visit or use each site or application, if at all?
¿Con qué frecuencia visita o utiliza cada sitio o aplicación, si es que lo hace?

GRID ITEMS, RANDOMIZE:

A. Facebook
B. Instagram
C. Twitter
D. Snapchat
E. YouTube
F. Parler
G. TikTok

RND_04=0 1,2,3,4,5,6,7
RND_04=1 7,6,5,4,3,2,1
RESPONSE OPTIONS:

1. Never
2. Less than monthly
3. Monthly
4. Every couple weeks
5. A few times a week
6. About once a day
7. Several times a day
1. Nunca
2. Menos de un mes
3. Mensual
4. Cada dos semanas
5. Unas cuantas veces a la semana
6. Alrededor de una vez al día
7. Varias veces al día

[SHOW IF P_W4COMP=0]DISPLAY_PRES.
Next, we have several questions about voting.
A continuación, tenemos varias preguntas sobre votación.

[SHOW IF P_W4COMP=0] [SP]
TURNOUT.
In talking to people about elections, we often find that a lot of people were not able to vote because they weren't registered, they were sick, or they just didn't have time.
Al hablar con la gente sobre las elecciones, a menudo nos encontramos con que muchas personas no pudieron votar porque no estaban registradas, estaban enfermas o simplemente no tenían tiempo.

Which of the following statements best describes you:
Cuál de las siguientes declaraciones lo/a describe mejor:

RND_01=0 1,2,3,4
RND_01=1 4,3,2,1
CAWI RESPONSE OPTIONS:

1. I did not vote in the 2020 presidential election
2. I thought about voting this time, but didn't
3. I usually vote, but didn't this time
4. I am sure I voted in the 2020 presidential election
1. No voté en las elecciones presidenciales de 2020
2. Pensé en votar esta vez, pero no lo hice
3. Normalmente voto, pero esta vez no lo hice
4. Estoy seguro de que voté en las elecciones presidenciales de 2020

RND_01=0 1,2,3,4
RND_01=1 4,3,2,1
CATI RESPONSE OPTIONS:

1. You did not vote in the 2020 presidential election
2. You thought about voting this time, but didn't
3. You usually vote, but didn't this time
4. You are sure you voted in the 2020 presidential election
1. No votó en las elecciones presidenciales de 2020
2. Pensó en votar esta vez, pero no lo hizo
3. Normalmente vota, pero esta vez no lo hizo
4. Está seguro/a de que votó en las elecciones presidenciales de 2020

[SHOW IF TURNOUT=4]
[SP]
HOWVOTED.
Which one of the following best describes how you voted?
¿Cuál de las siguientes declaraciones describe mejor cómo votó?

CAWI RESPONSE OPTIONS:

1. Definitely voted in person at a polling place <u>before</u> election day
2. Definitely voted in person at a polling place <u>on</u> election day
3. Definitely voted <u>before</u> election day by mailing in my ballot or depositing my mail ballot into a drop box
4. Definitely voted <u>on</u> election day by mailing in my ballot or depositing my mail ballot into a drop box
5. Definitely voted in some other way
77. Not completely sure whether I voted or not
1. Definitivamente voté en persona en un lugar de votación <u>antes</u> el día de la elección
2. Definitivamente voté en persona en un lugar de votación <u>en</u> el día de la elección
3. Definitivamente voté <u>antes</u> del día de la elección enviando mi boleta o depositando mi boleta en un buzón
4. Definitivamente voté <u>en</u> el día de la elección enviando mi boleta o depositando mi boleta en un buzón
5. Definitivamente voté de alguna otra manera
77. No estoy completamente seguro de si voté o no

CATI RESPONSE OPTIONS:

1. Definitely voted in person at a polling place <u>before</u> election day
2. Definitely voted in person at a polling place <u>on</u> election day
3. Definitely voted <u>before</u> election day by mailing in your ballot or depositing your mail ballot into a drop box
4. Definitely voted <u>on</u> election day by mailing in your ballot or depositing your ballot into a drop box
5. Definitely voted in some other way
77. Not completely sure whether you voted or not

1. Definitivamente votó en persona en un lugar de votación antes el día de la elección
2. Definitivamente votó en persona en un lugar de votación en el día de la elección
3. Definitivamente votó antes del día de la elección enviando su boleta o depositando su boleta en un buzón
4. Definitivamente votó en el día de la elección enviando su boleta o depositando su boleta en un buzón
5. Definitivamente votó de alguna otra manera
77. No está completamente seguro de si votó o no

[SHOW IF TURNOUT=4]
VOTE_POST.
For whom did you vote for President of the United States?
¿Por quién votó usted para Presidente de los Estados Unidos?

SHOW IF RND_00=0:
RESPONSE OPTIONS:

12. Joe Biden (Democrat)
13. Donald Trump (Republican)
14. Jo Jorgensen (Libertarian)
15. Howie Hawkins (Green)
16. Other candidate, please specify: [TEXTBOX]
17. [CAWI I][CATI You] didn't vote in this race
79. Not sure
12. Joe Biden (demócrata)
13. Donald Trump (republicano)
14. Jo Jorgensen (libertario)
15. Howie Hawkins (verde)
16. Otro candidato, por favor especifique: [TEXTBOX]
17. [CAWI Yo no voté][CATI Usted no votó] en esta elección
79. No estoy seguro

SHOW IF RND_00=1:
RESPONSE OPTIONS:

2. Donald Trump (Republican)
1. Joe Biden (Democrat)
3. Jo Jorgensen (Libertarian)
4. Howie Hawkins (Green)
5. Other candidate, please specify: [TEXTBOX]
6. [CAWI I][CATI You] didn't vote in this race
77. Not sure
2. Donald Trump (republicano)
1. Joe Biden (demócrata)
3. Jo Jorgensen (libertario)
4. Howie Hawkins (verde)
5. Otro candidato, por favor especifique: [TEXTBOX]
6. [CAWI Yo no voté][CATI Usted no votó] en esta elección
77. No estoy seguro

[SHOW IF TURNOUT=4 AND P_SCMPGN=1]
[INSERT IF S_STATE=GA]
Your state had 2 senate seats up for election in November 2020. Please let us know who you voted for in each race.
Su estado tiene 2 escaños en el Senado para las elecciones de noviembre de 2020. Por favor, díganos por quién votó en la contienda por cada uno de los escaños.

[SHOW ALL]
VOTESENATE.
For whom did you vote for <u>U.S. Senator</u> [INSERT IF S_STATE=GA for the November 2020 election]?
¿Por quién votó usted para <u>Senador de los EE.UU.</u> [INSERT IF S_STATE=GA para las elecciones de noviembre de 2020]?

RESPONSE OPTIONS, RANDOMIZE:
1. [SHOW IF P_SCANDE1 NOT BLANK] [INSERT: P_SCANDE1] [CATI: CANDIDATE NAME PRONUNCIATION INSERT P_SCPRO1]
2. [SHOW IF P_SCANDE2 NOT BLANK] [INSERT: P_SCANDE2 [CATI: CANDIDATE NAME PRONUNCIATION INSERT P_SCPRO2]
3. [SHOW IF P_SCANDE3 NOT BLANK] [INSERT: P_SCANDE3 [CATI: CANDIDATE NAME PRONUNCIATION INSERT P_SCPRO3]
4. [SHOW IF P_SCANDE4 NOT BLANK] [INSERT: P_SCANDE4 [CATI: CANDIDATE NAME PRONUNCIATION INSERT P_SCPRO4]
5. [SHOW IF P_SCANDE5 NOT BLANK] [INSERT: P_SCANDE5 [CATI: CANDIDATE NAME PRONUNCIATION INSERT P_SCPRO5]
6. [SHOW IF P_SCANDE6 NOT BLANK] [INSERT: P_SCANDE6 [CATI: CANDIDATE NAME PRONUNCIATION INSERT P_SCPRO6]
7. [SHOW IF P_SCANDE7 NOT BLANK] [INSERT: P_SCANDE7 [CATI: CANDIDATE NAME PRONUNCIATION INSERT P_SCPRO7]
8. [SHOW IF P_SCANDE8 NOT BLANK] [INSERT: P_SCANDE8 [CATI: CANDIDATE NAME PRONUNCIATION INSERT P_SCPRO8]
9. [SHOW IF P_SCANDE9 NOT BLANK] [INSERT: P_SCANDE9 [CATI: CANDIDATE NAME PRONUNCIATION INSERT P_SCPRO9]
10. [SHOW IF P_SCANDE10 NOT BLANK] [INSERT: P_SCANDE10] [CATI: CANDIDATE NAME PRONUNCIATION INSERT P_SCPRO10]
11. Other, please specify: [TEXTBOX] [ANCHOR]
12. [CAWI I][CATI You] didn't vote in this race [ANCHOR]

1. [SHOW IF P_SCANDS1 NOT BLANK] [INSERT: P_SCANDS1] [CATI: CANDIDATE NAME PRONUNCIATION INSERT P_SCPRO1]
2. [SHOW IF P_SCANDS2 NOT BLANK] [INSERT: P_SCANDS2 [CATI: CANDIDATE NAME PRONUNCIATION INSERT P_SCPRO2]
3. [SHOW IF P_SCANDS3 NOT BLANK] [INSERT: P_SCANDS3 [CATI: CANDIDATE NAME PRONUNCIATION INSERT P_SCPRO3]
4. [SHOW IF P_SCANDS4 NOT BLANK] [INSERT: P_SCANDS4 [CATI: CANDIDATE NAME PRONUNCIATION INSERT P_SCPRO4]
5. [SHOW IF P_SCANDS5 NOT BLANK] [INSERT: P_SCANDS5 [CATI: CANDIDATE NAME PRONUNCIATION INSERT P_SCPRO5]

6. [SHOW IF P_SCANDS6 NOT BLANK] [INSERT: P_SCANDS6 [CATI: CANDIDATE NAME PRONUNCIATION INSERT P_SCPRO6]
7. [SHOW IF P_SCANDS7 NOT BLANK] [INSERT: P_SCANDS7 [CATI: CANDIDATE NAME PRONUNCIATION INSERT P_SCPRO7]
8. [SHOW IF P_SCANDS8 NOT BLANK] [INSERT: P_SCANDS8 [CATI: CANDIDATE NAME PRONUNCIATION INSERT P_SCPRO8]
9. [SHOW IF P_SCANDS9 NOT BLANK] [INSERT: P_SCANDS9 [CATI: CANDIDATE NAME PRONUNCIATION INSERT P_SCPRO9]
10.[SHOW IF P_SCANDS10 NOT BLANK] [INSERT: P_SCANDS10] [CATI: CANDIDATE NAME PRONUNCIATION INSERT P_SCPRO10]
11. Otro, por favor especifique: [TEXTBOX] [ANCHOR]
12. [CAWI Yo no voté][CATI Usted no votó] en esta carrera[ANCHOR]

[INSERT IF S_STATE=GA]
[SP]
VOTESENATE2
For whom did you vote for U.S. Senator [INSERT IF S_STATE=GA for the November 2020 election]?
¿Por quién votó usted para Senador de los EE.UU. [INSERT IF S_STATE=GA para las elecciones de noviembre de 2020]?

1. [SHOW IF P_SCANDE12 NOT BLANK] [INSERT: P_SCANDE12] [CATI: CANDIDATE NAME PRONUNCIATION INSERT P_SCPRO12]
2. [SHOW IF P_SCANDE22 NOT BLANK] [INSERT: P_SCANDE22 [CATI: CANDIDATE NAME PRONUNCIATION INSERT P_SCPRO22]
3. [SHOW IF P_SCANDE32 NOT BLANK] [INSERT: P_SCANDE32 [CATI: CANDIDATE NAME PRONUNCIATION INSERT P_SCPRO32]
4. Other, please specify: [TEXTBOX]
5. [CAWI I][CATI You] didn't vote in this race
1. [SHOW IF P_SCANDS12 NOT BLANK] [INSERT: P_SCANDS12] [CATI: CANDIDATE NAME PRONUNCIATION INSERT P_SCPRO12]
2. [SHOW IF P_SCANDS22 NOT BLANK] [INSERT: P_SCANDS22 [CATI: CANDIDATE NAME PRONUNCIATION INSERT P_SCPRO22]
3. [SHOW IF P_SCANDS32 NOT BLANK] [INSERT: P_SCANDS32 [CATI: CANDIDATE NAME PRONUNCIATION INSERT P_SCPRO32]
4. Otro, por favor especifique: [TEXTBOX]
5. [CAWI Yo no voté][CATI Usted no votó] en esta carrera[ANCHOR]

[SHOW IF TURNOUT=4 AND P_GCMPGN=1]
VOTEGOV.
For whom did you vote for Governor?
¿Por quién votó usted para Gobernador?

RESPONSE OPTIONS, RANDOMIZE:

1. [SHOW IF P_GCANDE1 NOT BLANK] [INSERT: P_GCANDE1] [CATI: CANDIDATE NAME PRONUNCIATION INSERT P_GCPRO1]
2. [SHOW IF P_GCANDE2 NOT BLANK] [INSERT: P_GCANDE2] [CATI: CANDIDATE NAME PRONUNCIATION INSERT P_GCPRO2]
3. [SHOW IF P_GCANDE3 NOT BLANK] [INSERT: P_GCANDE3] [CATI: CANDIDATE NAME PRONUNCIATION INSERT P_GCPRO3]
4. [SHOW IF P_GCANDE4 NOT BLANK] [INSERT: P_GCANDE4] [CATI: CANDIDATE NAME PRONUNCIATION INSERT P_GCPRO4]
5. Other, please specify: [TEXTBOX]
6. [CAWI I][CATI You] didn't vote in this race

1. [SHOW IF P_GCANDS1 NOT BLANK] [INSERT: P_GCANDS1] [CATI: CANDIDATE NAME PRONUNCIATION INSERT P_GCPRO1]
2. [SHOW IF P_GCANDS2 NOT BLANK] [INSERT: P_GCANDS2] [CATI: CANDIDATE NAME PRONUNCIATION INSERT P_GCPRO2]
3. [SHOW IF P_GCANDS3 NOT BLANK] [INSERT: P_GCANDS3] [CATI: CANDIDATE NAME PRONUNCIATION INSERT P_GCPRO3]
4. [SHOW IF P_GCANDS4 NOT BLANK] [INSERT: P_GCANDS4] [CATI: CANDIDATE NAME PRONUNCIATION INSERT P_GCPRO4]5.
5. Otro, por favor especifique: [TEXTBOX]
6.[CAWI Yo no voté][CATI Usted no votó] en esta carrera

[SHOW IF TURNOUT=4]
VOTEHOUSE.
For whom did you vote for U.S. House?
¿Por quién votó usted para la Cámara de Representantes de los EE.UU.?

RESPONSE OPTIONS, RANDOMIZE:

1. A Democratic candidate
2. A Republican candidate
3. Other, please specify: [TEXTBOX]
4. [CAWI I][CATI You] didn't vote in this race

1. Un candidato demócrata
2. Un candidato republican
3. Otro, por favor especifique: [TEXTBOX]
4. 4. [CAWI Yo no voté][CATI Usted no votó] en esta carrera

DISPLAY_SELF.
Lastly, [CAWI: we'd][CATI: I'd] like to ask you a few questions about yourself.
Finalmente, [CAWI: nos][CATI: me] gustaría hacerle algunas preguntas sobre usted.

[SP]
EMOT.
Please tell [CAWI: us][CATI: me] how much of the time during the past 4 weeks you felt…
Por favor, [CAWI: díganos][CATI: dígame] cuánto tiempo durante las últimas 4 semanas se sintió…

GRID ITEMS, RANDOMIZE:

A. Happy
B. Depressed
C. Anxious

A. Feliz
B. Deprimido
C. Ansioso

IF RND_02=0 1,2,3,4,5
IF RND_02=1 5,4,3,2,1
RESPONSE OPTIONS:

1. All the time
2. Often
3. Sometimes
4. Rarely
5. Never
1. Todo el tiempo
2. A menudo
3. A veces
4. Raramente
5. Nunca

[SP]
CITIZENSHIP.
Which of these statements best describes you?
¿Cuál de estas afirmaciones lo describe mejor?

CAWI RESPONSE OPTIONS:

1. I am an immigrant to the USA and a naturalized citizen
2. I am an immigrant to the USA and <u>not</u> a citizen of the USA
3. I was born in the USA but at least one of my parents is an immigrant
4. My parents and I were born in the USA but at least one of my grandparents was an immigrant
5. My parents, grandparents and I were all born in the USA
1. Soy un inmigrante en los Estados Unidos y un ciudadano naturalizado
2. Soy un inmigrante en los Estados Unidos y <u>no</u> un ciudadano naturalizado
3. Nací en los Estados Unidos pero al menos uno de mis padres es un inmigrante
4. Mis padres y yo nacimos en los Estados Unidos pero al menos uno de mis abuelos era un inmigrante
5. Mis padres, mis abuelos y yo nacimos en los Estados Unidos

CATI RESPONSE OPTIONS:

1. You are an immigrant to the USA and a naturalized citizen
2. You are an immigrant to the USA and not a citizen of the USA
3. You were born in the USA but at least one of your parents is an immigrant
4. Your parents and you were born in the USA but at least one of your grandparents was an immigrant
5. Your parents, grandparents and you were all born in the USA

1. Usted es un inmigrante en los Estados Unidos y un ciudadano naturalizado
2. Usted es un inmigrante en los Estados Unidos y no un ciudadano naturalizado
3. Nació en los Estados Unidos pero al menos uno de sus padres es un inmigrante
4. Sus padres y usted nacieron en los Estados Unidos pero al menos uno de sus abuelos era un inmigrante
5. Sus padres, sus abuelos y usted nacieron en los Estados Unidos

---

[SP]
BORNAGAIN.
Would you describe yourself as a ”born again” or evangelical Christian, or not?
¿Se describiría como un cristiano "nacido de nuevo" o evangélico, o no?

CAWI RESPONSE OPTIONS:

1. Yes
2. No

1. Sí
2. No

CATI RESPONSE OPTIONS:

1. YES
2. NO

1. SÍ
2. NO

[SP]
RELFREQ.
How often do you attend religious services?
¿Con qué frecuencia asiste a servicios religiosos?

RESPONSE OPTIONS:

1. Never
2. Less than once a year
3. About once or twice a year
4. Several times a year
5. About once a month
6. 2-3 times a month
7. Nearly every week
8. Every week

9. Several times a week
1. Nunca
2. Menos de una vez al año
3. Alrededor de una o dos veces al año
4. Varias veces al año
5. Alrededor de una vez al mes
6. 2-3 veces al mes
7. Casi todas las semanas
8. Cada semana
9. Varias veces a la semana

[SP]
RELIGION.
What is your present religion, if any?
¿Cuál es su religión actual, si es que la tiene?

RESPONSE OPTIONS:
1. Protestant
2. Roman Catholic
3. Mormon
4. Eastern or Greek Orthodox
5. Jewish
6. Muslim
7. Buddhist
8. Hindu
9. Atheist
10. Agnostic
11. Nothing in particular
12. Something else, please specify:
1. Protestante
2. Católica Romana
3. Mormón
4. Ortodoxa oriental o griega
5. Judío
6. Musulmán
7. Budista
8. Hindú
9. Ateo
10. Agnóstico
11. Ninguna en particular
12. Alguna más, por favor especifique:

[SHOW IF P_CONSENTW4=MISSING]
DISPLAY_REG.
Next, we ask for your help on a different part of the November 2020 US Election study that you are a research participant in.
A continuación, le pedimos su ayuda en una investigación voluntario relacionado con una parte diferente del estudio sobre las elecciones de noviembre de 2020 en los Estados Unidos en el que usted es un participante en la investigación.
The goal of this part of the study is to develop an understanding of how people participate in elections, such as by voting or donating to political campaigns. As a result, we would like to ask you to allow NORC to collect publicly available third-party data on whether you've voted or made a political contribution, if that data is available.
El objetivo de esta parte del estudio de desarrollar una compresión de la forma en que las personas participan en las elecciones, por ejemplo, votando o haciendo donaciones a campañas políticas. Como resultado, nos gustaría pedirle que permita a NORC datos de terceros disponibles públicamente sobre si ha votado o hecho una contribución política, si esos datos están disponibles.

[SHOW IF P_CONSENTW4=MISSING]
[SP]
CONSENT_REG.<u>The Data Collected and Your Privacy If You Choose to Participate in this part of the Study</u>
<u>Los datos recopilados y su privacidad si decide participar en esta parte del estudio</u>

- NORC will collect publicly available third-party data on whether you've voted or made a political contribution, if this data is available
- NORC will share this data on your voting and donation history with Facebook and exclude data that may directly identify you such as your name
- Facebook will join the third-party data it receives from NORC with data you previously consented to sharing for the November 2020 US Election research study (such as your survey data, and/or device data, as applicable), collectively called Combined Data
- This Combined Data will only be used for research purposes and will not be used to show you ads
- This Combined Data will be shared with Facebook's academic partners and, if legally required, with the Institutional Review Board (IRB) that reviewed this study
- All access to this Combined Data will be monitored and logged by Facebook
- Once this study is over, de-identified data (i.e. data where identifiers such as your name and other information that could reasonably be linked to you are removed) will be stored and shared by Facebook for future research on elections, to validate the findings of this study, or if required by law for an IRB inquiry

- NORC crecogerá datos de terceros disponibles públicamente sobre si usted ha votado o hecho una contribución política, si estos datos están disponibles
- NORC compartirá estos datos sobre su historial de votación y donaciones con Facebook y excluirá los datos que puedan identificarlo directamente, como su nombre.
- Facebook unirá a los datos de terceros que recibe de NORC con los datos que previamente consintió en compartir para el estudio de investigación de las elecciones de noviembre de 2020 en los Estados Unidos (como los datos de su encuesta, y/o los datos del dispositivo, según corresponda), llamados colectivamente Datos Combinados
- Estos datos combinados sólo se utilizarán con fines de investigación y no se utilizarán para mostrarle anuncios
- Estos Datos Combinados se compartirán con los socios académicos de Facebook y, si se requiere legalmente, con la Junta de Revisión Institucional (IRB) que estuvo a cargo de revisó este estudio
- Todo el acceso a estos datos combinados será monitoreado y registrado por Facebook
- Una vez finalizado este estudio, los datos desidentificados (es decir, en los que se eliminan los identificadores como su nombre y otra información que podría estar razonablemente vinculada a usted) serán almacenados y compartidos por Facebook para futuras investigaciones sobre elecciones, para validar los resultados de este estudio o, si lo requiere la ley para una investigación de la IRB

You can decide to stop participating in this study at any time, for any reason, and without consequences. You may withdraw from the study by emailing [INSERT IF P_PANEL=1: support@amerispeak.org][INSERT IF P_PANEL=22: erpSurvey@norc.org][INSERT IF P_PANEL=23: erpStudy@norc.org] or calling [INSERT IF P_PANEL=1:AmeriSpeak support at (888) 326-9424][INSERT IF P_PANEL=22: toll-free (877) 839-1505] [INSERT IF P_PANEL=23: toll-free (866) 270-2602].
Puede decidir dejar de participar en este estudio en cualquier momento, por cualquier motivo y sin consecuencias. Puede retirarse del estudio enviando un correo electrónico a [INSERT IF P_PANEL=1: ayuda@amerispeak.org][INSERT IF P_PANEL=22: erpSurvey@norc.org] o llamando [INSERT IF P_PANEL=1:a la unidad de soporte de AmeriSpeak al (888) 326-9424][INSERT IF P_PANEL=22: gratis a (877) 839-1505].

Do you agree to share your information as described above?
¿Acepta compartir su información como se ha descrito anteriormente?

CAWI REPONSE OPTIONS:

1. Yes, I agree
2. No, I do not agree
1. Sí, estoy de acuerdo
2. No, no estoy de acuerdo

CATI REPONSE OPTIONS:

1. Yes, you agree
2. No, you do not agree
1. Sí, está de acuerdo
2. No, no está de acuerdo

[SHOW IF P_W4COMP=0]
[SP]
reg.
Are you now registered to vote, or are you not registered? [CATI: If you're not sure, you can say that too.]
¿Está usted registrado para votar o actualmente no está registrado? [CATI: Si no está seguro/a, puede decir eso también.]

CAWI RESPONSE OPTIONS:

5. Registered
6. Not registered
79. Not sure
5. Registrado
6. No registrado
79. No estoy seguro

CATI RESPONSE OPTIONS:

5. REGISTERED
6. NOT REGISTERED
79. NOT SURE
5. REGISTRADO
6. NO REGISTRADO
79. NO ESTOY SEGURO

[SHOW IF reg=1]
[SHOW IF P_MAILADDRESS AND P_CITY AND S_STATE AND P_ZIP NOT MISSING]
regloc1.
Where are you registered to vote?
¿Dónde está registrado para votar?

CAWI RESPONSE OPTIONS:

1. At [P_MAILADDRESS P_CITY, S_STATE P_ZIP]
2. At another address
77. Not sure
1. En [P_MAILADDRESS P_CITY, S_STATE P_ZIP]
2. En otra dirección
77. No estoy seguro

[SHOW IF regloc1=2 OR (reg=1 AND P_MAILADDRESS OR P_CITY OR S_STATE OR P_ZIP MISSING)]
regloc2.
What is the address where you are registered to vote now?
¿Cuál es la dirección donde está registrado para votar ahora?

regloc2_add. Address
regloc2_city. City

regloc2_st. State
regloc2_zip. Zip
regloc2_add. Dirección
regloc2_city. Ciudad
regloc2_st. Estado
regloc2_zip. Código postal

[SHOW IF regloc1 = 77,98,99 or regloc2_state = 98]
regstate.
In what state are you registered to vote now?
¿En qué estado está registrado para votar ahora?

THIS IS THE IG/FB ACCOUNT LINKING SECTION – SHOWN TO AMSP + ABS SAMPLE SOURCES WHO ARE FB or IG USER BASED ON PRELOADED SURVEY RESPONSES AT W2
[SHOW IF (P_W4COMP=0 OR P_RED_ERROR=1) AND CAWI AND (PANEL_TYPE=<20,22 AND (P_FB_USER=1 OR P_IG_USER=1))]
INTRO_7.
Next, we ask for your help on a related voluntary research study of how people use Facebook and Instagram to learn about current events.
A continuación, le pedimos su ayuda en un estudio de investigación voluntario sobre cómo las personas usan Facebook e Instagram para conocer temas de actualidad.

[SHOW IF (P_W4COMP=0 OR P_RED_ERROR=1) AND CAWI AND (PANEL_TPYE=1,22 AND (P_FB_USER=1 OR P_IG_USER=1))]
[SP]
CONSENT_FBIG.
[INSERT IF PANEL_TYPE<20]
<u>The Data Collected and Your Privacy If You Choose to Participate in the Study</u>
<u>Los datos recopilados y su privacidad si decide participar en el estudio</u>

- NORC will join your survey responses to publicly available third-party data like if you've voted or made a political contribution, if this data is available
- Facebook will combine this data with your activity on Facebook and Instagram from the 2020 calendar year, collectively called Combined Data
- This Combined Data will only be used for research purposes and will not be used to show you ads
- This Combined Data will be shared with Facebook, their academic partners and, if legally required, with the Institutional Review Board (IRB) that reviewed this study
- All access to this Combined Data will be monitored and logged by Facebook and NORC
- Once this study is over, de-identified data may be stored and shared by Facebook for future research on elections, to validate the findings of this study, or if required by law for an IRB inquiry

- NORC cruzará sus respuestas a la encuesta con datos de terceros disponibles públicamente, como por ejemplo si usted ha votado o hecho una contribución política, si estos datos están disponibles
- Facebook combinará estos datos con su actividad en Facebook e Instagram en el año 2020, colectivamente llamados Datos Combinados
- Estos datos combinados sólo se utilizarán con fines de investigación y no se utilizarán para mostrarle anuncios
- Estos Datos Combinados se compartirán con Facebook, sus socios académicos y, si se requiere legalmente, con la Junta de Revisión Institucional (IRB) que estuvo a cargo de revisó este estudio
- Todo el acceso a estos datos combinados será monitoreado y registrado por Facebook y NORC
- Una vez finalizado este estudio, Facebook puede almacenar y compartir datos anónimos para futuras investigaciones sobre elecciones, para validar los resultados de este estudio o, si así lo exige la ley, para una consulta del IRB

You can decide to stop participating in this study at any time, for any reason, and without consequences. You may withdraw from the study by emailing support@amerispeak.org or calling AmeriSpeak support at (888) 326-9424.
Puede decidir dejar de participar en este estudio en cualquier momento, por cualquier motivo y sin consecuencias. Puede retirarse del estudio enviando un correo electrónico a ayuda@amerispeak.org o llamando a la unidad de soporte de AmeriSpeak al (888) 326-9424.

Do you agree to share this information with Facebook?
¿Acepta compartir esta información con Facebook?

[INSERT IF PANEL_TYPE=22]
The Data Collected and Your Privacy If You Choose to Participate in the Study
Los datos recopilados y su privacidad si decide participar en el estudio

- NORC will join your survey responses to publicly available third-party data like if you've voted or made a political contribution, if this data is available
- Facebook will combine this data with your activity on Facebook and Instagram from the 2020 calendar year, collectively called Combined Data
- This Combined Data will only be used for research purposes and will not be used to show you ads
- This Combined Data will be shared with Facebook, their academic partners and, if legally required, with the Institutional Review Board (IRB) that reviewed this study
- All access to this Combined Data will be monitored and logged by Facebook and NORC
- Once this study is over, de-identified data may be stored and shared by Facebook for future research on elections, to validate the findings of this study, or if required by law for an IRB inquiry

- NORC cruzaráunirá sus respuestas a la encuesta con datos de terceros disponibles públicamente, como por ejemplo ha votado o hecho una contribución política, si estos datos están disponibles
- Facebook combinará estos datos con su actividad en Facebook e Instagram en el año 2020, colectivamente llamados Datos Combinados
- Estos datos combinados sólo se utilizarán con fines de investigación y no se utilizarán para mostrarle anuncios
- Estos datos combinados se compartirán con Facebook, sus socios académicos y, si se requiere legalmente, con la Junta de Revisión Institucional (IRB) que estuvo a cargo de revisó este estudio
- Todo el acceso a estos datos combinados será monitoreado y registrado por Facebook y NORC
- Una vez finalizado este estudio, Facebook puede almacenar y compartir datos anónimos para futuras investigaciones sobre elecciones, para validar los resultados de este estudio o, si así lo exige la ley, para una consulta del IRB

You can decide to stop participating in this study at any time, for any reason, and without consequences. You may withdraw from the study by visiting 2020erp.norc.org, by emailing erpSurvey@norc.org or by calling toll-free (877) 839-1505.
Puede decidir dejar de participar en este estudio en cualquier momento, por cualquier motivo y sin consecuencias. Puede retirarse del estudio visitando 2020erp.norc.org, enviando un correo electrónico a erpSurvey@norc.org o llamando al número gratuito (877) 839-1505.

Do you agree to share this information with Facebook?
¿Acepta compartir esta información con Facebook?

CAWI REPONSE OPTIONS:

1. Yes, I agree
2. No, I do not agree
1. Sí, estoy de acuerdo
2. No, no estoy de acuerdo

CATI REPONSE OPTIONS:

1. Yes, you agree
2. No, you do not agree
1. Sí, está de acuerdo
2. No, no está de acuerdo

[SHOW IF CONSENT_FBIG=1 AND ((P_FB_USER=1 AND P_IG_USER=0) OR (P_IG_USER=1 AND P_FB_USER=0))]
CONST2_FBIG.
Thank you. When you click “Continue” you will be taken to [INSERT IF P_FB_USER=1 AND P_IG_USER=0 Facebook][INSERT IF P_IG_USER=1 AND P_FB_USER=0 Instagram] to confirm your account. Once you confirm your account, you’ll be sent back here to complete the survey.
Gracias. Cuando haga clic en "Continuar", se le llevará a [INSERT IF P_FB_USER = 1 AND P_IG_USER = 0 Facebook] [INSERT IF P_IG_USER = 1 AND P_FB_USER = 0 Instagram] para confirmar su cuenta. Una vez que confirme su cuenta, se le enviará de regreso aquí para completar la encuesta.

REDIRECT TO FACEBOOK/INSTAGRAM, CONFIRM IDENTITY, THEN REDIRECT BACK TO THE SUREVY TO RESUME AT NEXT ITEM.

FACEBOOK
IF PANEL_TYPE=22 (ABS):
https://www.facebook.com/distance_survey/?oid=821494361720519&id1=<P_EPIN>&id2=1

IF PANEL_TYPE<20 (AmeriSpeak):
https://www.facebook.com/distance_survey/?oid=821494361720519&id1=<P_EPIN>&id2=2

INTAGRAM
IF PANEL_TYPE=22 (ABS):
https://www.instagram.com/fbsurvey/confirm_user/?survey_fbid=3422369734466790&id1=<P_EPIN>&id2=1

IF PANEL_TYPE<20 (AmeriSpeak):
https://www.instagram.com/fbsurvey/confirm_user/?survey_fbid=3422369734466790&id1=<P_EPIN>&id2=2

[SHOW IF CONSENT_FBIG=1 AND (P_FB_USER=1 AND P_IG_USER=1)]
CONSENT2_FB.
Thank you. When you click “Continue” you will go to a Facebook screen to confirm your account.
Gracias. Cuando haga clic en "Continuar", irá a una pantalla de Facebook para confirmar su cuenta.

REDIRECT TO FACEBOOK, CONFIRM IDENTITY, THEN REDIRECT BACK TO THE SUREVY TO CONSENT WITH INSTAGRAM.

FACEBOOK
IF PANEL_TYPE=22 (ABS):
https://www.facebook.com/distance_survey/?oid=821494361720519&id1=<P_EPIN>&id2=1

IF PANEL_TYPE<20 (AmeriSpeak):
https://www.facebook.com/distance_survey/?oid=821494361720519&id1=<P_EPIN>&id2=2

IF R FINISHES CLIENT SURVEY, CLIENT WILL CREATE FLAG:

IF P_FB_USER=1
AND FBSTAT=C “finished external client survey”
AND FBSTAT= (MISSING) “did not finish external client survey”
IF P_IG_USER=1
AND IGSTAT=C “finished external client survey”
AND IGSTAT= (MISSING) “did not finish external client survey”

[SHOW IF (FBSTAT=C AND (P_FB_USER=1 AND P_IG_USER=0)) OR (IGSTAT=C AND (P_IG_USER=1 AND P_FB_USER=0))] Respondent finished external client survey
RESUME1_FBIG.
Thank you for allowing Facebook to share this information. Please click "Continue" to resume the survey.
Gracias por permitir que Facebook comparta esta información. Por favor haga clic en "Continuar" para reanudar la encuesta.

[SHOW IF ((FBSTAT = MISSING AND P_FB_USER=1 AND P_IG_USER=0) OR (IGSTAT = MISSING AND P_IG_USER=1 AND P_FB_USER=0)) AND CONSENT_FBIG=1)] Respondent consented, but did not finish external survey
RESUME2_FBIG.
Please click "Continue" to resume the survey.
Por favor haga clic en "Continuar" para reanudar la encuesta.

[SHOW IF FBSTAT=C AND (P_FB_USER=1 AND P_IG_USER=1)] Respondent finished external client survey
RESUMED1_FB.
Thank you for allowing Facebook to share this information. Please click "Continue" to go to an Instagram screen to confirm your account.
Gracias por permitir que Facebook comparta esta información. Por favor haga clic en "Continuar" para ir a una pantalla de Instagram y confirmar su cuenta.

INTAGRAM
IF PANEL_TYPE=22 (ABS):
https://www.instagram.com/fbsurvey/confirm_user/?survey_fbid=3422369734466790&id1=<P_EPIN>&id2=1

IF PANEL_TYPE<20 (AmeriSpeak):
https://www.instagram.com/fbsurvey/confirm_user/?survey_fbid=3422369734466790&id1=<P_EPIN>&id2=2

[SHOW IF FBSTAT = MISSING AND CONSENT_FBIG=1 AND (P_FB_USER=1 AND P_IG_USER=1)]
Respondent consented, but did not finish external survey
RESUMED2_FB.
Please click "Continue" to go to an Instagram screen to confirm your account.
Por favor haga clic en "Continuar" para ir a una pantalla de Instagram y confirmar su cuenta.

INTAGRAM
IF PANEL_TYPE=22 (ABS):
https://www.instagram.com/fbsurvey/confirm_user/?survey_fbid=3422369734466790&id1=<P_EPIN>&id2=1

IF PANEL_TYPE<20 (AmeriSpeak):
https://www.instagram.com/fbsurvey/confirm_user/?survey_fbid=3422369734466790&id1=<P_EPIN>&id2=2

[SHOW IF IGSTAT=C AND (P_FB_USER=1 AND P_IG_USER=1)] Respondent finished external client survey
RESUMED3_FB.
Thank you for allowing Instagram to share this information. Please click “Continue” to resume the survey.
Gracias por permitir que Instagram comparta esta información. Por favor haga clic en "Continuar" para reanudar la encuesta.

[SHOW IF IGSTAT = MISSING AND CONSENT_FBIG=1 AND (P_FB_USER=1 AND P_IG_USER=1)]
Respondent consented, but did not finish external survey
RESUMED4_FB.
Please click “Continue” to resume the survey.
Por favor haga clic en "Continuar" para reanudar la encuesta.

[SHOW IF MODE=CAWI AND ((P_W4COMP=1 AND P_RED_ERROR=0,MISSING) OR (P_W4COMP=0 AND P_FB_USER=0 AND P_IG_USER=0))]
[SP]
TWITACCT.
We’re interested in learning a little more about how people use Twitter. Do you have an account on the social networking site Twitter?
Estamos interesados en aprender un poco más sobre cómo la gente usa Twitter. ¿Usted tiene una cuenta en la red social Twitter?

CAWI RESPONSE OPTIONS:
1. Yes
2. No
1. Sí
2. No

[SHOW IF TWITACCT=1]
TWITPERM.
Next, we ask for your help on another different part of the November 2020 US Election Study that you are a research participant in.
A continuación, le pedimos su ayuda en otra parte diferente del estudio de las elecciones de noviembre de 2020 en los Estados Unidos. en el que usted es un participante de la investigación.

As social media plays an increasing role in society, we would like to know who uses Twitter, and how people use it. The overarching goal of this part of the study is to develop an understanding of people’s

use of social media during the lead up to and after the 2020 US elections. As a result, we would like to ask you to share your Twitter account handle with NORC and verify that it's yours so we may look at what you have publicly posted, commented on, followed, and engaged with on Twitter.
Como los medios sociales juegan un papel cada vez más importante en la sociedad, nos gustaría saber quién usa Twitter y cómo lo usa la gente. El objetivo general de esta parte del estudio es desarrollar una comprensión del uso de los medios sociales por parte de la gente durante el período previo y posterior a las elecciones estadounidenses de 2020. Como resultado, nos gustaría pedirle que comparta su nombre de usuario de Twitter con NORC y verifique que es suya para que podamos ver lo que ha publicado, comentado, seguido y participado con públicamente en Twitter.

If you link your Twitter account, you will receive an additional [INSERT IF PANEL_TYPE<20: 5,000 AmeriPoints][INSERT IF PANEL_TYPE=22,23: $5].
Si usted conecta su cuenta de Twitter, recibirá [INSERT IF PANEL_TYPE<20: 5,000 AmeriPoints][INSERT IF PANEL_TYPE=22,23: $5] adicional.

[SHOW IF TWITACCT=1]
[SP]
TWIT_CONSENT.
<u>The Data Collected and Your Privacy If You Choose to Participate in this part of the Study</u>
Los datos recopilados y su privacidad si decide participar en esta parte del estudio

- NORC will collect data from your Twitter account that is publicly available. This will include your account information from July 1, 2020 through December 31, 2020, such as your profile description, who you follow and who follows you, the content of your tweets (including text, images, videos and web links), and background information about your tweets (such as when you tweeted, what type of device you tweeted from, and if enabled, the location the tweet was sent from)
- NORC will share your Twitter data with Facebook and exclude data that may directly identify you such as your Twitter handle or display name
- Facebook will join the Twitter data it receives from NORC with data you previously consented to sharing for the November 2020 US Election research study (such as your survey data, publicly available third-party data, your activity on Facebook and Instagram from the 2020 calendar year, and/or device data, as applicable), collectively called Combined Data
- This Combined Data will be shared with Facebook's academic partners and, if legally required, with the Institutional Review Board (IRB) that reviewed this study
- This Combined Data will only be used for research purposes and will not be used to show you ads
- All access to this Combined Data will be monitored and logged by Facebook
- Once this study is over, de-identified data (i.e. data where identifiers such as your name and other information that could reasonably be linked to you are removed) will be stored and shared by Facebook for future research on elections, to validate the findings of this study, or if required by law for an IRB inquiry
- NORC recogerá datos de su cuenta de Twitter que estén disponibles públicamente. Esto incluirá información de su cuenta desde el 1 de julio de 2020 hasta el 31 de diciembre de 2020 como la descripción de su perfil, a quién sigue y quién le sigue a usted, el contenido de sus tweets (incluyendo texto, imágenes, vídeos y enlaces web), e información de fondo sobre sus tweets

(como cuándo hizo el tweet, desde qué tipo de dispositivo lo hizo y, si está configurado, la ubicación desde la que se envió el tweet)

- NORC compartirá sus datos de Twitter con Facebook y excluirá los datos que puedan identificarlo directamente, como su nombre de usuario en Twitter o nombre de perfil
- Facebook unirá los datos de Twitter que recibe de NORC con los datos que usted puede haber consentido previamente en compartir para el estudio de investigación de las elecciones de noviembre de 2020 en los Estados Unidos (como los datos de su encuesta, los datos de terceros disponibles públicamente, su actividad en Facebook e Instagram a partir del año calendario 2020, y/o los datos del dispositivo, como corresponda), denominados colectivamente Datos Combinados
- Estos Datos Combinados serán compartidos con los socios académicos de Facebook y, si se requiere legalmente, con la Junta de Revisión Institucional (IRB) que revisó este estudio
- Estos datos combinados sólo se utilizarán para fines de investigación y no se usarán para mostrarle anuncios
- Todo acceso a estos Datos Combinados será monitoreado y registrado por Facebook
- Una vez finalizado este estudio, los datos des-identificados (es decir, los datos en los que se eliminan los identificadores como su nombre y otra información que podría estar razonablemente vinculada a usted) án serán almacenados y compartidos por Facebook para futuras investigaciones sobre las elecciones, para validar los resultados de este estudio, o si lo requiere la ley para una investigación de la IRB

You can decide to stop participating in this study at any time, for any reason, and without consequences. You may withdraw from the study by emailing [INSERT IF P_PANEL=1: support@amerispeak.org][INSERT IF P_PANEL=22: erpSurvey@norc.org][INSERT IF P_PANEL=23: erpStudy@norc.org] or calling [INSERT IF P_PANEL=1:AmeriSpeak support at (888) 326-9424][INSERT IF P_PANEL=22: toll-free (877) 839-1505] [INSERT IF P_PANEL=23: toll-free (866) 270-2602]. If you have questions about your rights as a research participant, please contact the NORC IRB at 1-866-309-0542 or send an email to irb@norc.org.
Puede decidir dejar de participar en este estudio en cualquier momento, por cualquier motivo y sin consecuencias. Puede retirarse del estudio enviando un correo electrónico a [INSERT IF P_PANEL=1: ayuda@amerispeak.org][INSERT IF P_PANEL=22: erpSurvey@norc.org][INSERT IF P_PANEL=23: erpStudy@norc.org] o llamando [INSERT IF P_PANEL=1:a la unidad de soporte de AmeriSpeak al (888) 326-9424][INSERT IF P_PANEL=22: gratis a (877) 839-1505][INSERT IF P_PANEL=23: gratis a (866) 270-2602]. Si tiene preguntas sobre sus derechos como participante en una investigación, por favor contacte al NORC IRB al 1-866-309-0542 o envíe un correo electrónico a irb@norc.org.

Do you agree to share this information as described above?
¿Acepta compartir esta información como se ha descrito anteriormente?

CAWI REPONSE OPTIONS:

1. Yes, I agree
2. No, I do not agree

1. Sí, estoy de acuerdo
2. No, no estoy de acuerdo

[SHOW IF TWIT_CONSENT =1]
TWITPERM_2.

Thank you. When you click “Continue” you will be taken to Twitter to confirm your account.
Gracias. Cuando haga clic en "Continuar", se le llevará a Twitter para confirmar su cuenta.

Once on Twitter, you will be asked to enter your account name and authorize the app. If you have multiple Twitter accounts please enter the account you use most frequently for personal reasons.
Una vez en Twitter, se le pedirá que introduzca su nombre de cuenta y que autorice la aplicación. Si tiene varias cuentas de Twitter por favor introduzca la cuenta que utiliza con más frecuencia por razones personales.

Once you confirm your account, you’ll be sent back here to complete the survey.
Una vez que confirme su cuenta, se le enviará de regreso aquí para completar la encuesta.

If you decide you do not want to confirm your account and chose “Cancel” on the next screen, you will need to choose to “Return to 2020 Election Research Project” (see image below) in order to return to this survey and let us know how you would like to receive your incentives.
Si decide que no quiere confirmar su cuenta y elige "Cancelar" en la siguiente pantalla, tendrá que elegir “Volver a 2020 Election Research Project” (ver imagen abajo) para volver a esta encuesta y hacernos saber cómo le gustaría recibir sus incentivos.

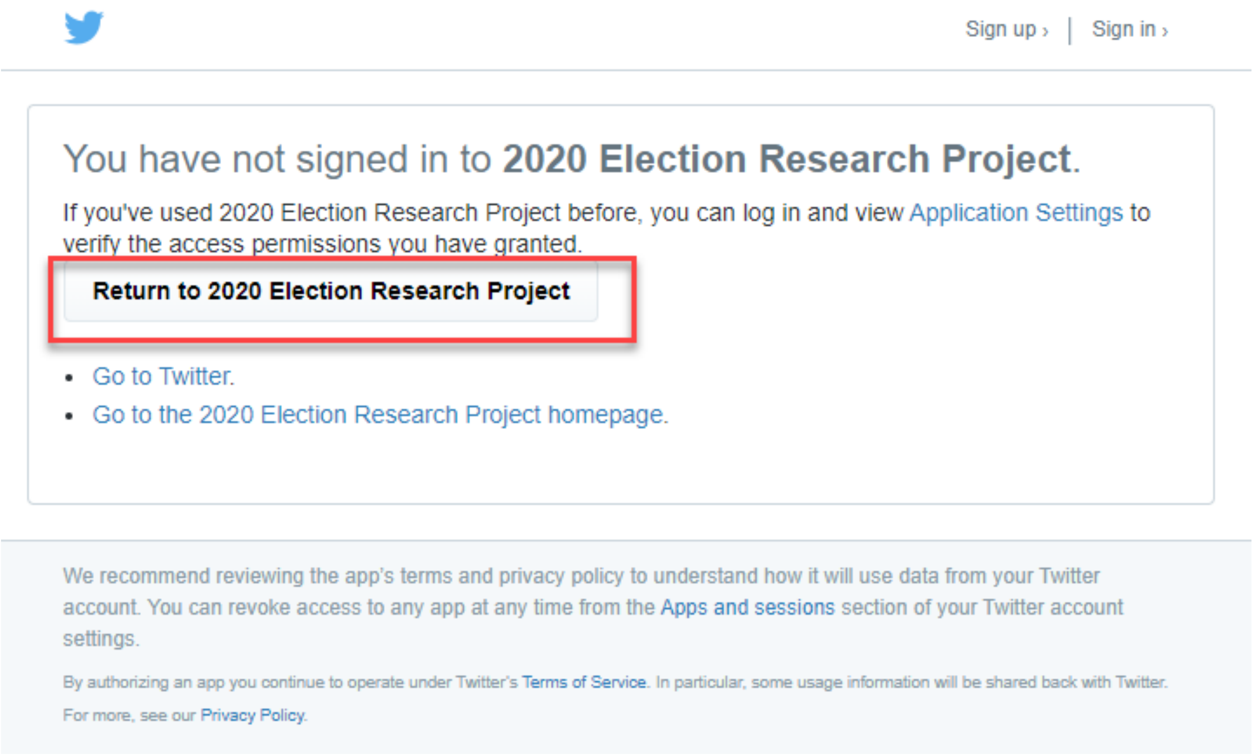

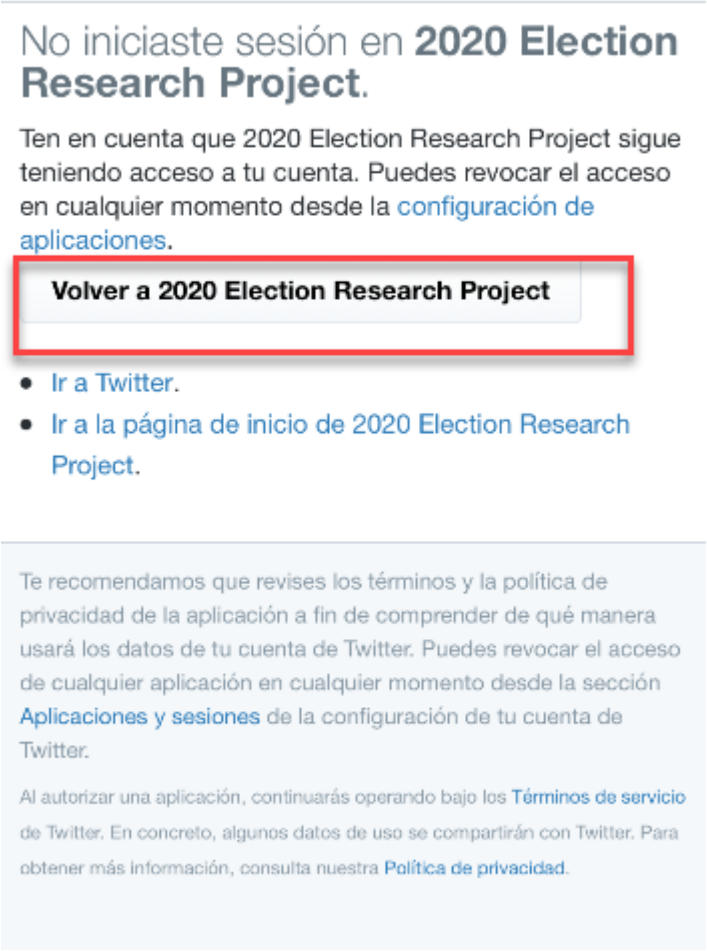

REDIRECT TO TWITTER, CONFIRM IDENTITY, THEN REDIRECT BACK TO THE SUREVY TO RESUME AT NEXT ITEM.

TWITTER
https://erpauth.norc.org/twitter/authenticate?st=<TOKEN>&p=<PIN>

AFTER R FINISHES TWITTER AUTHORIZATION, THEY WILL BE REDIRECTED BACK TO THE SURVEY AND THE TSAT VARIABLE WILL BE PASSED AS FOLLOWS:

IF TSTAT=1 “success”
IF TSTAT= 2, (MISSING) “fail”

[SHOW IF TSTAT=1] Respondent finished twitter authorization
RESUME1_TWIT.
Thank you for verifying your Twitter account name. Please click “Continue” to resume the survey.
Gracias por verificar el nombre de su cuenta de Twitter. Por favor haga clic en "Continuar" para reanudar la encuesta.

[SHOW IF ((TSTAT = 2,MISSING AND TWITPERM =1)] Respondent consented, but did not finish twitter authorization
RESUME2_TWIT.
What is the username for the account you use most frequently for personal reasons?
¿Cuál es su nombre de usuario para la cuenta que utiliza con más frecuencia por razones personales?

Twitter usernames must have a maximum of 15 characters (A-Z, a-z, 0-9, underscore), no word spaces. Please do not include the @ character.
Los nombres de usuario de Twitter deben tenerun máximo de 15 caracteres (A-Z, a-z, 0-9, guión bajo), sin espacios de palabras. Por favor, no incluya el carácter @.

Remember that all your answers will be kept confidential and used only for research purposes.
Recuerde que todas sus respuestas se mantendrán de forma confidencial y se usarán sólo con fines de investigación.

END.
Those are all the questions we have. The survey is now complete. Thank you!
Esas fueron todas las preguntas. La encuesta ya está completa. ¡Gracias!

[SPACE]
[IF P_SAMPLE_GRP=3,4] You may now reactivate your [INSERT IF P_SAMPLE_GRP=3: Facebook][INSERT IF P_SAMPLE_GRP=4: Instagram] account.
[IF P_SAMPLE_GRP=3,4] Ahora puede reactivar su cuenta de [INSERT IF P_SAMPLE_GRP=3: Facebook][INSERT IF P_SAMPLE_GRP=4: Instagram].

[IF PANEL_TYPE<20: [IF TSTAT=1 OR TVALID=1 To thank you for confirming your Twitter username, we've added 5,000 AmeriPoints to your total reward.] We will add [INCENTWCOMMA] AmeriPoints to your AmeriPoints balance for completing the survey today.
[IF PANEL_TYPE<20 AND P_W3COMP=1 AND P_W4COMP=1: And since you completed all 4 Election Research Project surveys, you will also receive 15,000 bonus AmeriPoints.]
If you have any questions at all for us, you can email us at support@AmeriSpeak.org or call us toll-free at **888-326-942**4. [CATI: Let me repeat that again: email us at support@AmeriSpeak.org or call us at **888-326-9424**.] Thank you for participating in our new AmeriSpeak survey!
[IF PANEL_TYPE<20 [IF TSTAT=1 OR TVALID=1 Para agradecerle la confirmación de su nombre de usuario de Twitter, hemos añadido 5.000 AmeriPointsa su premio total.] Agregaremos [INCENTWCOMMA] AmeriPoints a su saldo de AmeriPoints por completar la encuesta hoy.
[IF PANEL_TYPE<20 AND P_W3COMP=1 AND P_W4COMP=1: Y ya que ha completado las 4 encuestas del Proyecto de Investigación Electoral, también recibirá 15.000 AmeriPoints de bonificación.]
Si tiene alguna pregunta, puede enviarnos un correo electrónico a ayuda@AmeriSpeak.org o llamarnos al número gratuito **888-326-9424**. [CATI: Permítame repetirlo nuevamente: envíenos un correo electrónico a ayuda@AmeriSpeak.org o llámenos al **888-326-9424**.] ¡Gracias por participar en nuestra nueva encuesta AmeriSpeak!]

[CAWI: Please click Continue below to submit your answers.]
[CAWI: Por favor haga clic en Continuar a continuación para enviar sus respuestas.]

4.12 References

54. Kevin Coe, Kate Kenski, and Stephen A. Rains. Online and uncivil? patterns and determinants of incivility in newspaper website comments. Journal of Communication, 64(4):658–679, Aug 2014.

55. Yoav Benjamini, Abba M. Krieger, and Daniel Yekutieli. Adaptive linear step-up procedures that control the false discovery rate. Biometrika, 93(3):491–507, Sep 2006.

56. Michael L Anderson. Multiple inference and gender differences in the effects of early intervention: A reevaluation of the Abecedarian, Perry Preschool, and Early Training Projects. Journal of the American statistical Association, 103(484):1481–1495, 2008.

57. Mark J. van der Laan and Alexander R. Luedtke. Targeted learning of an optimal dynamic treatment, and statistical inference for its mean outcome. Unpublished manuscript. Downloaded October 22, 2021 from https://core.ac.uk/download/pdf/61322095.pdf, 2014.

58. Alexander R Luedtke and Mark J van der Laan. Super-learning of an optimal dynamic treatment rule. The international journal of biostatistics, 12(1):305–332, 2016.

59. Edward H Kennedy. Optimal doubly robust estimation of heterogeneous causal effects. Unpublished manuscript. Downloaded October 22, 2021 from https://arxiv.org/abs/2004.14497, 2020.

60. James M Robins, Andrea Rotnitzky, and Lue Ping Zhao. Analysis of semiparametric regression models for repeated outcomes in the presence of missing data. Journal of the American Statistical Association, 90(429):106–121, 1995.

61. Mark J Van der Laan, MJ Laan, and James M Robins. Unified methods for censored longitudinal data and causality. Springer Science & Business Media, 2003.

62. Anastasios A Tsiatis. Semiparametric theory and missing data. Springer, 2006.

63. Anastasios A Tsiatis, Marie Davidian, Min Zhang, and Xiaomin Lu. Covariate adjustment for two-sample treatment comparisons in randomized clinical trials: A principled yet flexible approach. Statistics in medicine, 27(23):4658–4677, 2008.

64. Victor Chernozhukov, Denis Chetverikov, Mert Demirer, Esther Duflo, Christian Hansen, Whitney Newey, and James Robins. Double/debiased machine learning for treatment and structural parameters. Econometrics Journal, 21(1):C1–C68, 2018.

65. Edward H Kennedy, Zongming Ma, Matthew D McHugh, and Dylan S Small. Nonparametric methods for doubly robust estimation of continuous treatment effects. Journal of the Royal Statistical Society: Series B (Statistical Methodology), 79(4):1229–1245, 2017.

66. Jianqing Fan and Irene Gijbels. Local polynomial modelling and its applications. Routledge, 1996.

67. Trevor Hastie, Robert Tibshirani, and Jerome Friedman. The elements of statistical learning. Springer-Verlag, 2001.

68. Qingyuan Zhao and Trevor Hastie. Causal interpretations of black-box models. Journal of business & economic statistics, 2019.

69. Brian D Williamson, Peter B Gilbert, Noah R Simon, and Marco Carone. A general framework for inference on algorithm-agnostic variable importance. Journal of the American Statistical Association, 2021.

70. José Hernández-Orallo. ROC curves for regression. Pattern Recognition, 46(12):3395–3411, 2013.

71. Justin Esarey and Jane Lawrence Sumner. Marginal effects in interaction models: Determining and controlling the false positive rate. Comparative Political Studies, 51(9):1144–1176, 2018.

72. David E Broockman. Approaches to studying policy representation. Legislative Studies Quarterly, 41(1):181–215, 2016.

73. Jeffrey B Lewis, Keith Poole, Howard Rosenthal, Adam Boche, Aaron Rudkin, and Luke Sonnet. Voteview: Congressional roll-call votes database. See https://voteview.com/(accessed 4 April 2023), 2023.

74. Alexandra Siegel, Evgenii Nikitin, Pablo Barbera, Joanna Sterling, Bethany Pullen, Richard Bonneau, Jonathan Nagler, and Joshua Tucker. Trumping hate on Twitter? online hate speech and white nationalist rhetoric in the 2016 US election campaign and its aftermath. Quarterly Journal of Political Science, 2021.

75. Federico Bianchi, Stefanie Anja Hills, Patricia Rossini, Dirk Hovy, Rebekah Tromble, and Nava Tintarev. 'It's not just hate': A multi-dimensional perspective on detecting harmful speech online. arXiv preprint arXiv:2210.15870, 2022.

76. Bertie Vidgen, Dong Nguyen, Helen Margetts, Patricia Rossini, and Rebekah Tromble. Introducing CAD: The contextual abuse dataset. In Proceedings of the 2021 Conference of the North American Chapter of the Association for Computational Linguistics: Human Language Technologies, pages 2289–2303, 2021.

77. Kevin Coe, Kate Kenski, and Stephen A Rains. Online and uncivil? Patterns and determinants of incivility in newspaper website comments. Journal of Communication, 64(4):658–679, 2014.

78. Patrícia Rossini. Beyond incivility: Understanding patterns of uncivil and intolerant discourse in online political talk. Communication Research, 49(3):399–425, 2022.

79. Sam Davidson, Qiusi Sun, and Magdalena Wojcieszak. Developing a new classifier for automated identification of incivility in social media. In Proceedings of the fourth workshop on online abuse and harms, pages 95–101, 2020.

80. Yannis Theocharis, Pablo Barberá, Zoltán Fazekas, and Sebastian Adrian Popa. The dynamics of political incivility on Twitter. Sage Open, 10(2):2158244020919447, 2020.

81. Graeme Blair, Jasper Cooper, Alexander Coppock, and Macartan Humphreys. Declaring and diagnosing research designs. American Political Science Review, 113(3):838–859, 2019.

82. Simon Bate and Natasha A Karp. A common control group-optimising the experiment design to maximise sensitivity. PloS one, 9(12):e114872, 2014.

83. Hunt Allcott, Luca Braghieri, Sarah Eichmeyer, and Matthew Gentzkow. The welfare effects of social media. American Economic Review, 110(3):629–76, 2020.
