# Supplementary material for: Like-minded sources on Facebook are prevalent but not polarizing
Source: Nature. 2023 Jul 27;620(7972):137–44. doi: 10.1038/s41586-023-06297-w (PMC10396953; doi:10.1038/s41586-023-06297-w)
Supplement: Supplementary file 2 — Reporting Summary [file 41586_2023_6297_MOESM2_ESM.pdf]

nature portfolio

Corresponding author(s): Brendan Nyhan

Last updated by author(s): 04/28/2023

Reporting Summary

Nature Portfolio wishes to improve the reproducibility of the work that we publish. This form provides structure for consistency and transparency in reporting. For further information on Nature Portfolio policies, see our Editorial Policies and the Editorial Policy Checklist.

Statistics

For all statistical analyses, confirm that the following items are present in the figure legend, table legend, main text, or Methods section.

n/a	Confirmed	
☐	☒	The exact sample size (*n*) for each experimental group/condition, given as a discrete number and unit of measurement
☐	☒	A statement on whether measurements were taken from distinct samples or whether the same sample was measured repeatedly
☐	☒	The statistical test(s) used AND whether they are one- or two-sided<br>*Only common tests should be described solely by name; describe more complex techniques in the Methods section.*
☐	☒	A description of all covariates tested
☐	☒	A description of any assumptions or corrections, such as tests of normality and adjustment for multiple comparisons
☐	☒	A full description of the statistical parameters including central tendency (e.g. means) or other basic estimates (e.g. regression coefficient) AND variation (e.g. standard deviation) or associated estimates of uncertainty (e.g. confidence intervals)
☐	☒	For null hypothesis testing, the test statistic (e.g. *F*, *t*, *r*) with confidence intervals, effect sizes, degrees of freedom and *P* value noted<br>*Give P values as exact values whenever suitable.*
☒	☐	For Bayesian analysis, information on the choice of priors and Markov chain Monte Carlo settings
☒	☐	For hierarchical and complex designs, identification of the appropriate level for tests and full reporting of outcomes
☐	☒	Estimates of effect sizes (e.g. Cohen's *d*, Pearson's *r*), indicating how they were calculated

Our web collection on statistics for biologists contains articles on many of the points above.

Software and code

Policy information about availability of computer code

Data collection	Data collection was carried out by Meta and NORC, an independent survey research organization at the University of Chicago. Meta recruited most participants and collected on-platform data. NORC carried out all surveys associated with the project, recruited additional survey panelists, collected all supplemental data outside of the Facebook/Instagram on-platform data, and removed any direct identifiers before linking to the survey data and sharing with the research team.<br><br>On-platform behavioral data were collected via Meta's internal systems for logging user behavior. Survey data were collected by NORC using their existing survey infrastructure. To collect the passive measurement data, NORC partnered with two vendors: MDI Global and RealityMine. Users who consented to passive data tracking were asked to install an app and use a virtual private network (VPN) on their mobile or desktop devices to collect data about the number of visits and time spent on different web domains as well as usage and time spent on apps on their mobile device. The app was developed by MDI Global and the VPN was developed and maintained by RealityMine. Both firms collected the passive tracking data and sanitized, truncated, and/or categorized the URLs to minimize the risk of sharing any additional personally identifiable information (PII).
Data analysis	Analysis code from this study is archived in the Social Media Archive (SOMAR) at ICPSR (https://socialmediaarchive.org) and made available in the ICPSR virtual data enclave for university IRB-approved research on elections or to validate the findings of this study per the data availability statement above. The data in this study was analyzed using R (version 4.1.1), which was executed via R notebooks on JupyterLab (3.2.3). The analysis code imports several R packages available on CRAN, including dplyr (1.0.10), ggplot2 (3.4.0), xtable (1.8-4), aws.s3 (0.3.22), glmnet (4.1.2), SuperLearner (2.0-28), margins (0.3.26), and estimatr (1.0.0).

For manuscripts utilizing custom algorithms or software that are central to the research but not yet described in published literature, software must be made available to editors and reviewers. We strongly encourage code deposition in a community repository (e.g. GitHub). See the Nature Portfolio guidelines for submitting code & software for further information.

Data

Policy information about availability of data

All manuscripts must include a data availability statement. This statement should provide the following information, where applicable:

- Accession codes, unique identifiers, or web links for publicly available datasets
- A description of any restrictions on data availability
- For clinical datasets or third party data, please ensure that the statement adheres to our policy

De-identified data from this project (Meta Platforms, Inc. Facebook Intervention Experiment Participants. Inter-university Consortium for Political and Social Research [distributor], 2023-07-27. https:// doi.org/10.3886/9wct-2d24; Meta Platforms, Inc. Exposure to and Engagement with Facebook Posts. Inter-university Consortium for Political and Social Research [distributor], 2023-07-27. https://doi. org/10.3886/9sqy-ny89; Meta Platforms, Inc. Ideological Alignment of Users in Facebook Networks. Inter-university Consortium for Political and Social Research [distributor], 2023-07-27. https://doi. org/10.3886/nvh0-jh41; Meta Platforms, Inc. Facebook User Attributes. Inter-university Consortium for Political and Social Research [distribu- tor], 2023-07-27. https://doi.org/10.3886/vecn-ze56; Stroud, Natalie J., Tucker, Joshua A., NORC at the University of Chicago, and Meta Plat- forms, Inc. US 2020 FIES NORC Data Files. Inter-university Consortium for Political and Social Research [distributor], 2023-07-27. https://doi. org/10.3886/0d26-d856) is available under controlled access from the Social Media Archive (SOMAR) at the University of Michigan's Inter-university Consortium for Political and Social Research (ICPSR). The data can be accessed via ICPSR's virtual data enclave for university IRB-approved research on elections or to validate the findings of this study. ICPSR will accept and vet all applications for data access. Data access is controlled to protect the privacy of the study participants and to be consistent with the consent form signed by study participants where they were told that their data would be used for "future research on elections, to validate the findings of this study, or if required by law for an IRB inquiry." Requests for data can be made via the SOMAR website (https://socialmediaarchive.org); inquiries can be directed to SOMAR staff at somar-help@umich.edu. ICPSR staff will respond to requests for data within 2-4 weeks of submission. To access the data, the home institution of the academic making the request must complete ICPSR's Restricted Data Agreement.

The categorization methods described in section S6.2 rely on two open-source labeled datasets: a list of slurs sourced from Hatebase (hatebase.org) and the Racial Slur Database (rsdb.org) that was compiled by Siegel et al. (2021) and two sets of social media posts annotated by human coders by whether they are perceived as uncivil or not (Theocharis et al. 2020; Davidson et al. 2020). We describe these in more detail in Section 4.3.2 of the SI, "Other classifiers and categorization methods."

Human research participants

Policy information about studies involving human research participants and Sex and Gender in Research.

Reporting on sex and gender	We confirm that we do not use the terms gender or sex in the main text. Several analyses in the SI employ gender, which is measured via survey self-report (male, female, and other). Gender was determined based on survey self-reports. Informed consent was provided prior to collecting survey data.<br><br>For our main findings, gender is included as a candidate covariate (included covariates were selected via lasso). In addition, we conduct several analyses that examine how the effects of the treatment on exposure to different types of content vary by gender (as well as a variety of other subgroups). These analyses are available in Section S3.9.5 of the SI, "HTE analysis for exploratory moderators."
Population characteristics	Our experimental sample is 73.3% white (2.2% Asian or Pacific Islander, 6.7% Black, Non-Hispanic, 12% Hispanic, 5.7% Other), 57.3% female (41.9% male and 0.8% Other), and relatively highly educated (50.7% have a college degree. With regard to self-reported party identification, the sample is Democratic-leaning (54.1% self-identify as Democrats or lean Democrat, 33.5% self-identify as Republicans or lean Republican, and 12.4% are Independents leaning toward neither party). With regard to self-reported ideology, 41.2% were very or somewhat liberal, 33.4% "middle of the road" and 25.3% were somewhat or very conservative.
Recruitment	We summarize the recruitment strategy below (it is briefly described in the main text as well). Further details are provided in Section S4.6 in the SI. At the top of their Facebook feed, randomly selected participants saw a recruitment message asking them if they would like to share their opinion. Those clicking "Start Survey" were directed to a consent form. Participants gave their consent to participate using an IRB-approved consent form that outlined the study procedure, benefits and risks, and compensation.<br><br>Our analyses show that participants use Facebook more frequently than the general Facebook population and are exposed to more politically like-minded content (the phenomenon of interest), including like-minded civic and news content, than are other Facebook users. To address potential self-selection bias, our treatment effect estimates on attitudes apply survey weights created to reflect the population of adult monthly active Facebook users who were eligible for recruitment.<br><br>We also note that randomization into the treatment and control condition was successful, with no statistically significant differences between the groups on 25 out of 26 characteristics (see Table S5 in the SI). We also provide evidence there showing no indication of differential attrition across waves by treatment status and quartile of pre-treatment exposure to content from like-minded sources (see Table S6). As such, attrition bias should not impact the results.
Ethics oversight	We have complied with all relevant ethical regulations. The overall project was reviewed and approved by the NORC IRB. Academic researchers worked with their respective university IRBs to ensure compliance with human subjects research regulations in analyzing data collected by NORC and Meta and authoring papers based on those findings. The research team also received ethical guidance from the independent firm Ethical Resolve to inform study designs. More detailed information is provided in Sections S1.2 and S4.9 of the SI.

All participants provided informed consent before taking part (see SI Section S4.6 for recruitment and consent materials). Participants were given the option to withdraw from the study while the experiment was ongoing as well as to withdraw their data at any time up until their survey responses were disconnected from any identifying information in February 2023. We also implemented a stopping rule, inspired by clinical trials, which stated that we would terminate the intervention before the election if we detected it was generating changes in specific variables related to individual welfare that were much larger than expected. More details are available in SI Section S1.2.

None of the academic researchers received compensation from Meta for their participation in the project. The analyses were preregistered at the Open Science Foundation. The lead authors retained final discretion over everything reported in this paper. Meta publicly agreed that there would be no pre-publication approval of papers for publication on the basis of their findings. See SI Section S4.8 for more details about the Meta-academic collaboration.

Note that full information on the approval of the study protocol must also be provided in the manuscript.

Field-specific reporting

Please select the one below that is the best fit for your research. If you are not sure, read the appropriate sections before making your selection.

☐ Life sciences ☒ Behavioural & social sciences ☐ Ecological, evolutionary & environmental sciences

For a reference copy of the document with all sections, see nature.com/documents/nr-reporting-summary-flat.pdf

Behavioural & social sciences study design

All studies must disclose on these points even when the disclosure is negative.

Study description

We rely on an over-time experimental design. Respondents were assigned to treatment or control with equal probability using block randomization. The News Feed of participants in the control condition was not systematically altered. For participants assigned to treatment, we downranked content from friends, Groups, and Pages who were predicted to share the participant's estimated political leaning. The details on the design are presented in the Design section of the main text, as well as in Section S1 in the SI, “Materials and Methods.” The data are quantitative.

Research sample

Participants in the experiment consisted of U.S. Facebook users age 18 and over who agreed to participate in a study of social media and politics and completed both baseline survey waves. They were recruited via survey invitations placed at the top of their feeds and remunerated for their participation (details on sampling are provided in Section S8 of the Supplementary Information). The sampling frames included all Facebook monthly active U.S.-based users 18 years of age or older eligible to receive general surveys on a given platform (these represent a random set of users from the overall Facebook populations) as of August 17, 2020. Participants were asked to confirm they were over 18 years of age and lived in the United States as part of the recruitment process. Platform-wide statistics were provided as aggregate data for U.S.-based users 18 years of age or older who were active at least once per month, a standard social media measure often known as monthly active users/people (MAP/MAU). This sample represents the subset of adults (18+ years old) in the 231 million people who accessed Facebook every month during this period.

Sampling strategy

Below we summarize our sampling strategy. Further details are available in Section S8 of the SI, “Sampling, strata definitions, randomization, and power analyses.”

The sampling approach was designed to achieve specific sample targets across different stages of the study. The sample targets were chosen to achieve desired minimum detectable effect sizes (MDEs) across different subgroups. The sampling frames included all Facebook monthly active U.S.-based users 18 years of age or older eligible to receive general surveys on a given platform (these represent a random set of users from the overall Facebook populations) as of August 17, 2020. The sample stratification took into account the following variables: number of days a user was active on a given platform, a user’s predicted census region, whether the user is predicted to live in a battleground state, a user’s predicted ideology, and the census ethnic/racial composition in the zip code in which a user is predicted to live. Sampling probabilities were computed to achieve specific sample distributions for the set of demographics encoded in the stratification step across each of the samples of interest. The sampling probabilities took into account (a) differential nonresponse across different demographics (see section S10) and (b) the desired sample size across the different studies. The frame was adjusted as we reviewed the incoming data (see section S8.3). We designed our sampling approach with the goal of recruiting the minimum number of respondents required to detect meaningful effect sizes (see SI Section S8.4, “Power calculations,” for more detail).

Data collection

Data was collected from participants on their own devices (e.g., mobile phones and computers). The survey vendor we used (NORC) was blind to the experimental condition of each subject as well as our hypotheses.

Timing

Data collection started on August 31, 2020. Two surveys were fielded pre-treatment: Wave 1 (August 31-September 12) and Wave 2 (September 8-23). The treatment ran from September 24-December 23. During the treatment period, three more surveys were administered: Wave 3 (October 9-23), Wave 4 (November 4-18), and Wave 5 (December 9-23). This process is outlined in the “Field experiment among consenting U.S. Facebook users section of the main manuscript.”

Data exclusions	Data from 25 users (0.1% of the sample) for whom no classifier prediction for ideology was available were excluded from the analyses because it would not be possible to determine whether certain sources of online content were congenial or cross-cutting for those participants. Details on this exclusion are available in SI Section S1.3, “Classifiers.” This exclusion criteria was preregistered.
Non-participation	We detail information about recruitment and response rates for the collaboration in the Supplementary Information S9.4. In total, 75,318 participants were randomized into one of the experimental conditions within the collaboration. Of these, 8 (0.01%) withdrew from the study after completing a post-treatment wave, and 1,369 (1.8%) deleted or deactivated their Facebook account since the study was completed. Data from these participants are not included in the analyses in this paper. This information is detailed in S2.1.3 of the SI, “Deleted Accounts and Study Withdrawals.”
Randomization	Respondents were randomly assigned to treatment or control with probabilities that maximized statistical power using block randomization. A combination of survey-based pre-treatment outcomes and Facebook data were used to define the blocks in the sample of interest. The full details are available in Section S9.3 of the SI, “Randomization.”

Reporting for specific materials, systems and methods

We require information from authors about some types of materials, experimental systems and methods used in many studies. Here, indicate whether each material, system or method listed is relevant to your study. If you are not sure if a list item applies to your research, read the appropriate section before selecting a response.

Materials & experimental systems

n/a	Involved in the study
☒	☐ Antibodies
☒	☐ Eukaryotic cell lines
☒	☐ Palaeontology and archaeology
☒	☐ Animals and other organisms
☒	☐ Clinical data
☒	☐ Dual use research of concern

Methods

n/a	Involved in the study
☒	☐ ChIP-seq
☒	☐ Flow cytometry
☒	☐ MRI-based neuroimaging
